# Synthesis of Gb_3_ Glycosphingolipids with Labeled Head Groups: Distribution in Phase‐Separated Giant Unilamellar Vesicles

**DOI:** 10.1002/anie.201910148

**Published:** 2019-10-21

**Authors:** Jeremias Sibold, Katharina Kettelhoit, Loan Vuong, Fangyuan Liu, Daniel B. Werz, Claudia Steinem

**Affiliations:** ^1^ Georg-August-Universität Göttingen Institute of Organic and Biomolecular Chemistry Tammannstr. 2 37077 Göttingen Germany; ^2^ Technische Universität Braunschweig Institute of Organic Chemistry Hagenring 30 38106 Braunschweig Germany; ^3^ Max Planck Institute for Dynamics and Self Organization Am Faßberg 17 37077 Göttingen Germany

**Keywords:** carbohydrates, fatty acids, fluorescence, membranes, toxins

## Abstract

The receptor lipid Gb_3_ is responsible for the specific internalization of Shiga toxin (STx) into cells. The head group of Gb_3_ defines the specificity of STx binding, and the backbone with different fatty acids is expected to influence its localization within membranes impacting membrane organization and protein internalization. To investigate this influence, a set of Gb_3_ glycosphingolipids labeled with a BODIPY fluorophore attached to the head group was synthesized. C_24_ fatty acids, saturated, unsaturated, α‐hydroxylated derivatives, and a combination thereof, were attached to the sphingosine backbone. The synthetic Gb_3_ glycosphingolipids were reconstituted into coexisting liquid‐ordered (*l*_o_)/liquid‐disordered (*l*_d_) giant unilamellar vesicles (GUVs), and STx binding was verified by fluorescence microscopy. Gb_3_ with the C_24:0_ fatty acid partitioned mostly in the *l*_o_ phase, while the unsaturated C_24:1_ fatty acid distributes more into the *l*_d_ phase. The α‐hydroxylation does not influence its partitioning.

## Graphical abstract

**Making the invisible visible**: Shiga toxin B subunits (STxB) bind to liquid‐ordered (*l*_o_) domains in model membranes, implying that its receptor Gb_3_ is localized in these *l*_o_ domains. The synthetic access to Gb_3_ glycosphingolipids with labeled head groups allows quantification of their partitioning in coexisting *l*_o_/*l*_d_ (liquid‐disordered) phase giant unilamellar vesicles prior to STxB binding. The data clearly indicate an impact of the fatty acid (un)saturation and α‐hydroxylation on the partitioning between phases.

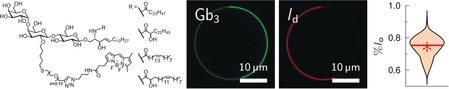

## Introduction

The eukaryotic plasma membrane of animals is a heterogeneous structure with a plethora of different lipids. The main lipid components are glycerophospholipids, sterols, and sphingolipids.^1^ Among them, glycosphingolipids serve a particular role. They are found in the outer leaflet of the plasma membrane and are discussed to reside preferentially in so‐called raft domains, which are enriched in sphingomyelin (SM) and cholesterol (Chol).^2^, ^3^, ^4^ Their size, chemical composition, and physical characteristics are tightly associated with their signal processing capabilities.^5^ Raft domains are supposed to have diameters of 10–200 nm and are highly dynamic structures.^3^, ^6^ This combination of smallness and dynamics bears the major challenge in visualizing raft domains in cellular membranes.^4^ Hence, two approaches have been pursued within the last decades to shed some light on the structure and function of these domains. On the one hand, detergent‐resistant membranes were extracted from cells and their composition analyzed, however they turned out to be prone to artefacts.^7^ On the other hand, artificial membranes with lipid compositions resembling the outer leaflet of the plasma membrane were reconstituted, which separate into a liquid‐disordered (*l*_d_) and a liquid‐ordered (*l*_o_) phase.^8^ Typical lipid compositions comprise a low‐melting glycerophospholipid, a high‐melting glycerophospholipid or SM, and Chol.^9^ The *l*_d_ phase has loose lateral lipid packing, acyl chains with gtg kinks, and fast lateral diffusion. In contrast, the *l*_o_ phase is characterized by a tighter lipid packing and a higher degree of order, but still rather fast lateral diffusion.^10^ However, the size and physical properties of *l*_o_ domains formed in artificial membranes are very different from those found in the plasma membrane. This difference becomes obvious if comparing, for example, the physicochemical properties of coexisting *l*_o_/*l*_d_ phase‐separated GUVs with those of phase‐separated cell‐derived membranes termed giant plasma membrane vesicles (GPMVs).^11^ Despite this difference between the natural and artificial membrane systems, artificial coexisting *l*_o_/*l*_d_ membranes have been frequently used to analyze the partitioning of receptor lipids and proteins,^12^ such as bacterial toxins, in the different phases.^13^

Bacterial toxins are known to bind to specific glycosphingolipids embedded in the outer leaflet of the plasma membrane. Cholera toxin (CTx) produced by *Vibrio cholerae* and Shiga toxin (STx) produced by *Shigella dysenteriae* and by enterohemorrhagic strains of *Escherichia coli*, both belonging to the class of AB_5_ toxins,^14^ bind specifically to monosialotetrahexosylganglioside (G_M1_)^15^ and globotriaosyl ceramide (Gb_3_),^16^, ^17^ respectively. While the head groups of the glycosphingolipids indeed define the specificity of protein binding, not much attention has been drawn to the variability of the ceramide backbone harboring different fatty acids. In various cell types (human colon Caco‐2, HCT‐8 epithelial cells, human endothelial cell lines, primary human umbilical vein endothelial cells, primary human endothelial cells of the brain and the kidney,^18^ and references therein), a conserved repertoire of Gb_3_ species was found carrying saturated C_16:0_, C_22:0_, or C_24:0_ fatty acids as well as the unsaturated C_24:1_ fatty acid. Results of Lingwood and co‐workers^19^ suggest that the pathogenic outcome of Shiga toxin producing *E. coli* (STEC) infections is related to the different Gb_3_ species. To gather more molecular information, artificial membranes doped with Gb_3_ were employed. In coexisting *l*_o_/*l*_d_ supported lipid membranes, Gb_3_ species differing in their fatty acid gave rise to a different phase behavior before and after binding of the B subunits of STx (STxB) as well as differences in the protein organization on the membrane surface.^20^, ^21^ In giant unilamellar vesicles (GUVs), Gb_3_ species with an unsaturated acyl chain caused the formation of tubular invaginations upon STxB binding, in contrast to Gb_3_ with a saturated acyl chain.^22^ In all these studies, it became evident that STxB binds exclusively to the *l*_o_ phase, which also implies that the receptor Gb_3_ is localized in the *l*_o_ phase after protein binding. However, it remains unclear how Gb_3_ is distributed in coexisting *l*_o_/*l*_d_ membranes prior protein binding.

To get access to this information, an approach based on fluorescently labeled Gb_3_ molecules can be pursued. However, it turned out that, if a fluorescent label is attached to the fatty acid position to ensure that the STxB interaction with the head group is not influenced by the fluorophore, binding of STxB is greatly altered.^23^ If a fatty acid labeled Gb_3_ is reconstituted into *l*_o_/*l*_d_ phase‐separated GUVs, the protein binds to the *l*_d_ phase and not to the *l*_o_ phase as known from membranes containing naturally occurring Gb_3_.

To date, only a few examples are found in the literature where synthetic routes towards glycosphingolipids with labeled head groups have been described.^24^ Here, we decided on a new strategy in line with approaches pursued for G_M1_ and G_M3_ 25 and focused on head group labeled Gb_3_. The idea is to develop fluorescently labeled Gb_3_ glycosphingolipids without altering its binding properties to STxB. We attached a fluorophore via an oligoethylene glycol spacer to the 2′‐OH group of the middle galactose of the Gb_3_ head group, which is not involved in STxB binding as deduced from crystal structure analysis^17^ and binding studies of different trisaccarides.^26^ This approach in turn allows us to alter the fatty acid of the Gb_3_ molecules.

## Results and Discussion

**Scheme 1:**
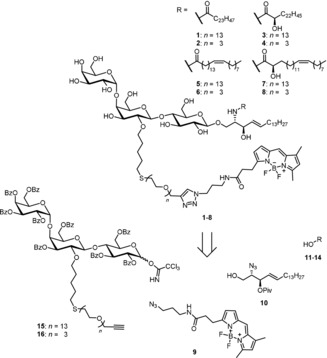
Retrosynthetic analysis of head group labeled Gb_3_PEG_*n*_R derivatives (*n=*3,13, R=different fatty acids C_24:0_H, C_24:0_OH, C_24:1_H and C_24:1_OH).

We synthesized a set of Gb_3_ sphingolipids as depicted in Scheme 1. Altogether eight different glycosphingolipids were synthesized and they consist of the globotriaose head group with two different oligoethylene glycol (PEG) linkers, to which a BODIPY fluorophore was attached and the sphingosine. Saturated, unsaturated, α‐hydroxylated derivatives, and a combination thereof were prepared, all based on a C_24_ fatty acid. C_24_ fatty acids were chosen as they are the major constituent (>50 %) found in natural Gb_3_ mixtures such as toxin insensitive erythrocytes,^27^ HeLa‐cells,^28^ and HEp‐2 cells.^29^

To access the head group labeled Gb_3_ derivatives with different fatty acids and PEG linker lengths we designed a modular convergent synthesis in which a variation of the fatty acid and the fluorophore is possible with minimal synthetic effort (Scheme 1). In contrast to semisynthetic approaches, a convergent total synthesis ensures the highly defined nature of the obtained material, which was crucial for our biophysical experiments. The retrosynthetic analysis of the desired structures **1**–**8** led to four different components. The commercially available BODIPY dye **9** should be attached to the carbohydrate head group in the last step of the synthesis by a Huisgen cycloaddition (click chemistry). The sphingosine core should be introduced as the azido sphingosine **10**. The azide serves as a masked amine which undergoes amide coupling with the four selected fatty acids (**11**–**14**) with a C_24_ backbone. Assembling the globotriose building blocks **15** and **16**, in which the 2‐hydroxy group of the middle galactose was modified with the PEG linker and the reducing end was activated for the glycosylation reaction with **10**, would be the most challenging endeavor during this synthesis. Monosaccharide building blocks with carefully chosen patterns of temporary and permanent protecting groups had to be synthesized starting from the simple monosaccharides d‐glucose and d‐galactose.

**Scheme 2:**
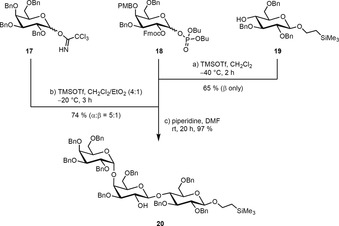
Assembly of the Gb_3_ trisaccharide.

Naturally occurring Gb_3_ molecules carry 24 carbon long fatty acids, either saturated or monounsaturated.^30^ The galactosyl trichloroacetimidate **17**, galactosyl phosphate **18**, and glucoside **19** were identified as suitable precursors to build up the trisaccharide (Scheme 2). They were prepared according to literature procedures.^31^ The union of **18** and **19** under Lewis‐acidic conditions utilizing TMSOTf as a promoter afforded the respective (1→4)‐linked disaccharide. Perfect β‐selectivity was observed because of the neighboring‐group participation of the Fmoc group of **18**. During the course of the reaction the *para*‐methoxybenzyl group of the galactose was cleaved,^32^ yielding the lactose acceptor for the second glycosylation step with **17** under Lewis‐acidic conditions without any additional deprotection step. The desired α‐configured product was isolated as the main product when diethyl ether was used as a cosolvent. Subsequent removal of the Fmoc protecting group with piperidine led to the trisaccharide **20**.

**Scheme 3:**
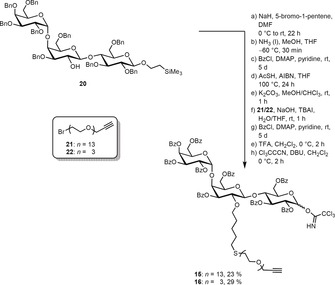
Synthesis of the trichloroacetimidates **15** and **16** with two different PEG linkers.

The trisaccharide **20** was then equipped with a pentenyl chain in the position where the fluorophore needs to be attached (Scheme 3). In the next step the substrate was subjected to Birch conditions to remove all benzyl protecting groups. Despite the strongly reducing conditions, the anomeric CH_2_CH_2_TMS group and the pentenyl handle stayed intact. Deprotection was followed by DMAP‐mediated benzoylation. In contrast to benzyl groups, benzoyl esters have the advantage that they can be easily removed at the end of the synthetic route without affecting the double bond in the lipid part of the glycosphingolipid.^21^ To attach the PEG linker, the double bond of the pentenyl handle was first transformed into a thioester with tioacetic acid under radical conditions. This species was hydrolyzed under basic conditions and the emerging highly nucleophilic thiol was subsequently reacted with the PEG bromides **21** (13 ethylene glycol units) and **22** (3 ethylene glycol units). To ensure a full protection of all hydroxy groups, the benzoylation step was repeated. Finally, the anomeric protecting group was removed with trifluoroacetic acid and the reducing end was converted into the corresponding trichloroacetimidates **15** and **16**.

**Scheme 4:**
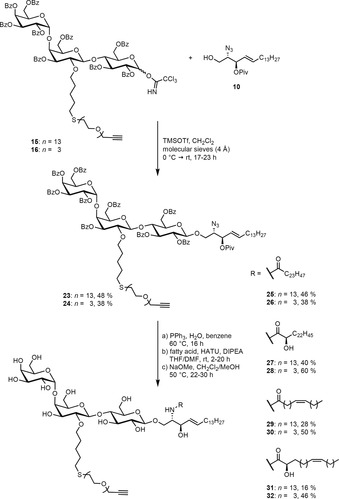
Gb_3_ glycosphingolipid assembly.

**Scheme 5:**
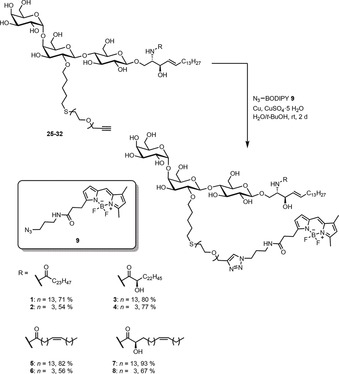
Huisgen cycloaddition of the BODIPY derivative **9** with the glycosphingolipids **25**–**32**.

To build up the glycolipid, the trichloroacetimidates were reacted with the protected azidosphingosine **10**,^33^ which was synthesized starting from the chiral pool compound l‐serine (for detailed information see the Supporting Information), in a glycosylation reaction utilizing TMSOTf as the Lewis acid to afford **23** and **24** in moderate yields (Scheme 4). Comparing experiments with globotriaosyl trichloroacetimidates devoid of the PEG modification indicated that the Lewis‐basic linker might hamper this very sensitive glycosylation step. Staudinger reduction of the azides and direct coupling with the fatty acids **11**–**14**,^21^, ^34^ without isolating the intermediary amines, afforded the PEG‐modified glycosphingolipids **25**–**32**. Global deprotection under Zemplén conditions set the stage for the final step of the synthesis. The commercially available BODIPY dye **9** was introduced into the glycosphingolipids by coupling its azide unit with the alkyne moiety of the PEG linker under mild copper(I)‐catalyzed conditions (Scheme 5). In total, eight different fluorescently labeled glycosphingolipids (**1**–**8**), varying in the PEG linker length and the acyl chain of the fatty acid, were obtained. The linker length (*n*) is either 3 or 13 oligoethylene glycol groups. The fatty acid (C_m:Δ_) is either saturated (C_24:0_) or unsaturated (C_24:1_). Hydroxylation at the α‐position is indicated by OH, and non‐hydroxylation is indicated by H.

Starting with the saturated C_24_ fatty acid and a PEG spacer composed of 13 oligoethylene glycol units, we prepared GUVs composed of the well‐known raft mixture 1,2‐dioleoyl‐*sn*‐glycero‐3‐phosphocholine (DOPC)/SM‐porc/Chol labeled with 5 mol % **1** and 0.25 mol % Texas Red‐DHPE (39.75/35/20/5/0.25) to address the question of whether STxB is indeed still capable of binding to the head group modified Gb_3_ and whether it binds to the *l*_o_ phase as expected.

**Figure 1:**
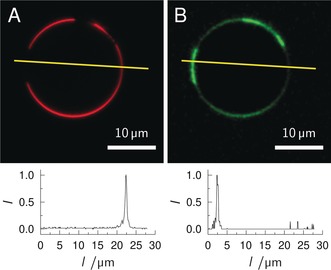
Confocal images of a phase‐separated GUV composed of DOPC/SM‐porc/Chol/**1**/Texas Red‐DHPE (39.75/35/20/5/0.25) in an aqueous solution of STxB‐Cy5 (500 nm, monomer). A) Texas Red‐DHPE fluorescence (red). B) STxB‐Cy5 fluorescence (green). The yellow lines indicate the position of the fluorescence intensity profiles shown below the images.

Figure 1 shows representative confocal images of a GUV in a 500 nm STxB‐Cy5 (monomer) solution. Texas Red‐DHPE partitions preferentially in the *l*_d_ phase, visualizing the coexisting *l*_o_/*l*_d_ membrane (Figure 1 A). The fluorescence image of STxB‐Cy5 shows that STxB binds to the GUV and that it binds to the *l*_o_ phase (Figure 1 B). This result confirms our hypothesis that the fluorescent label at the 2′‐OH position does not greatly interfere with the binding properties of STxB and is suited to investigate the partition of different Gb_3_ species as a function of the fatty acid in coexisting *l*_o_/*l*_d_ membranes.

**Figure 2:**
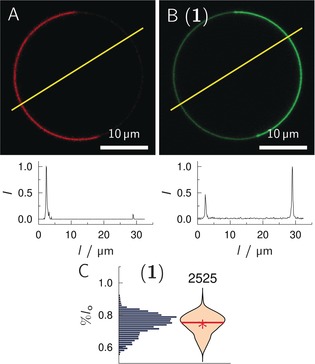
Confocal images of a phase‐separated GUV composed of DOPC/SM‐porc/Chol/**1**/Dy731‐DOPE (39/39/20/1/1). A) Dy731‐DOPE fluorescence (red). B) **1** fluorescence (green). The yellow lines indicate where the fluorescence intensity profiles (bottom images) were obtained. From the intensity profiles %*l*_o_=68.2 % was calculated for **1**. C) Histogram and corresponding violin plot obtained from 60 GUVs (number of line profiles atop) with the composition as in (A/B). The red solid line indicates the median value, the red star the mean value.

To quantitatively compare the phase partitioning among the different Gb_3_ species, we used the fluorophore Dy731‐DOPE as *l*_d_ marker^23^ to guarantee that the fluorescence of the BODIPY labeled Gb_3_ does not spectrally overlap with the absorption of the *l*_d_ marker. Moreover, the concentration of Gb_3_ was reduced from 5 to 1 mol % to ensure that self‐quenching of the BODIPY fluorophore is minimized (see Figure S1 in the Supporting Information). GUVs composed of DOPC/SM‐porc/Chol/Gb_3_/Dy731 (39/39/20/1/1) were prepared. As it is known that the composition of GUVs obtained by electroformation is rather heterogeneous,^35^ at least two independent GUV preparations with about 30 individual GUVs each were analyzed. Confocal *z*‐stack images were measured for each GUV and line profiles were taken from each slice, where phase separation was visible. An example of fluorescence images of a *l*_o_/*l*_d_ coexisting GUV together with the line profile is shown in Figure 2. The fluorophore Dy731‐DOPE indicates the *l*_d_ phase (Figure 2 A).^23^ From the BODIPY fluorescence intensity (Figure 2 B), the preferential localization of **1** is visible. To quantify the partition of **1**, the BODIPY intensity of the *l*_d_ phase (*I*(*l*_d_)) and of the *l*_o_ phase (*I*(*l*_o_)) as obtained from the corresponding line profile was determined and the *l*_o_ distribution (%*l*_o_) was calculated [Eq. (1)]:^23^

(1) $$\%l_{o}=\;\frac{I\left(l_{o}\right)}{I\left(l_{o}\right)+I\left(l_{d}\right)}$$

Several tens of line profiles were taken from each GUV. All %*l*_o_ values were cast into a histogram (Figure 2 C). Data obtained in this manner are presented as violin plots throughout the manuscript.

**Figure 3:**
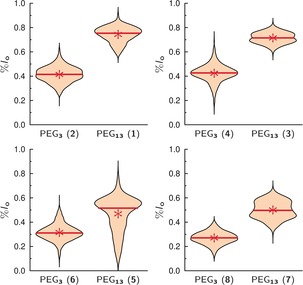
*l*_o_ distribution of different Gb_3_ species in GUVs composed of DOPC/SM‐porc/Chol/Gb_3_/Dy731 (39/39/20/1/1). The partition of Gb_3_ sphingolipids with the short PEG linker (PEG_3_) was compared with those carrying the long PEG linker (PEG_13_). The mean values are given as a red star, while the red solid lines show the median value (Table 1).

There is increasing evidence that the size of the linker attached to the head group of a lipid alters the phase behavior of the fluorescently labeled lipid.^36^, ^37^ To investigate whether the linker length, that is, the number of ethylene glycol units, influences the partition of the Gb_3_ sphingolipids in phase‐separated GUVs, we synthesized Gb_3_ molecules differing in their fatty acid with either 13 ethylene glycol units (PEG_13_) or 3 (PEG_3_). Independent of the fatty acid, the same trend is observed (Figure 3). All Gb_3_ sphingolipids with PEG_13_ partition more in the *l*_o_ phase than the corresponding Gb_3_ species with PEG_3_.

The mean values are summarized in Table 1. The difference between the *l*_o_ distribution of PEG_13_Gb_3_ species and PEG_3_Gb_3_ species lies between 0.15 and 0.33 (Table 2, ΔPEG). Such altered partitioning of a lipid as a function of linker length, to which a fluorophore has been attached, was also observed by Honigmann et al.^36^ They reported on a fluorophore that was either directly connected to the lipid 1,2‐distearoyl‐*sn*‐glycero‐3‐phosphoethanolamine (DSPE) or connected by a PEG‐linker with 45 ethylene glycol units, and was reconstituted into supported lipid membranes composed of 1,2‐diphytanoyl‐*sn*‐glycero‐3‐phosphocholine (DPhPC)/1,2‐dipalmitoyl‐*sn*‐glycero‐3‐phosphocholine (DPPC)/Chol. A fluorescence analysis of the partition clearly showed that the fluorescent lipid lacking the PEG‐linker was preferentially localized in the *l*_d_ phase, while that with the PEG‐linker partitioned into the *l*_o_ phase. Similarly, Momin et al.^38^ and Bordovsky et al.^37^ found that an increase in length of the hydrophilic PEG linker at the head group of lipids that are expected to be localized in the *l*_o_ phase of coexisting *l*_o_/*l*_d_ membranes is required to favor their partitioning in the *l*_o_ phase. This observation is explained by the notion that the fluorophore itself is partially hydrophobic and might be also bulky. It changes the packing parameter of the lipid. If the fluorophore is directly connected to the lipid or attached by a short linker, the size of the lipid's head group is expanded and the lipid is more conically shaped, favoring the *l*_d_ phase.^39^ For the slightly hydrophobic but small BODIPY fluorophore used in our study, a hydrophilic PEG spacer of suitable length is required to mitigate interactions with the membrane. Momin et al.^38^ found a linker with 10 ethylene glycol units to be sufficient to decouple the fluorophore from the membrane.^39^ In our study, a 13‐unit long linker decoupled the fluorophore from the membrane interface with the result that **1**, which is expected to at least preferentially partition into the *l*_o_ phase, indeed has a *l*_o_ distribution of almost 0.75. From these results, we conclude that the Gb_3_ species with PEG_13_ are better suited to report on the natural partition of Gb_3_ than those with PEG_3_. Thus, the experiments in which we compare the influence of unsaturation and hydroxylation of the fatty acid of Gb_3_ are all performed with the PEG_13_ species. The corresponding results with the PEG_3_ linker can be found in the Supporting Information (see Figures S2 and S3).

**Table 1:** Mean values of the *l*_o_ distributions (%*l*_o_) for the different Gb_3_ sphingolipids in GUVs composed of DOPC/SM‐porc/Chol/Gb_3_/Dy731 (39/39/20/1/1).

No.	Gb_3_	%*l*_o_ (*N*)
1	Gb_3_PEG_13_C_24:0_H	0.74±0.07 (2525)
2	Gb_3_PEG_3_C_24:0_H	0.41±0.07 (2516)
3	Gb_3_PEG_13_C_24:0_OH	0.71±0.05 (3064)
4	Gb_3_PEG_3_C_24:0_OH	0.42±0.08 (2273)
5	Gb_3_PEG_13_C_24:1_H	0.47±0.15 (1654)
6	Gb_3_PEG_3_C_24:1_H	0.32±0.07 (2351)
7	Gb_3_PEG_13_C_24:1_OH	0.50±0.08 (2377)
8	Gb_3_PEG_3_C_24:1_OH	0.27±0.06 (2701)

The errors are the standard deviation of the mean. *N*=number of line profiles.

**Table 2:** Differences in the mean values dependent on the functional group.

ΔPEG		ΔC24		ΔOH	
1‐2:	0.33±0.14	1‐5:	0.27±0.22	1‐3:	0.03±0.12
3‐4:	0.29±0.13	3‐7:	0.21±0.13	5‐7:	0.03±0.23
5‐6:	0.15±0.22	2‐6:	0.09±0.14	2‐4:	−0.01±0.15
7‐8:	0.23±0.14	4‐8:	0.15±0.14	6‐8:	0.05±0.13

ΔPEG=%*l*_o_ (PEG_13_)—%*l*_o_ (PEG_3_); ΔC24=%*l*_o_ (C_24:0_)—%*l*_o_ (C_24:1_); ΔOH=%*l*_o_ (H)—%*l*_o_ (OH).

**Figure 4:**
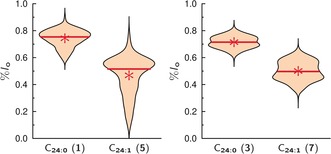
*l*_o_ distribution of different Gb_3_ species with a PEG_13_ linker in GUVs composed of DOPC/SM‐porc/Chol/Gb_3_/Dy731 (39/39/20/1/1). The partition of the Gb_3_ species harboring a saturated fatty acid (C_24:0_) were compared with that with an attached unsaturated fatty acid (C_24:1_). The mean values are given as a red star, while the red lines show the median value (Table 1).

We investigated the influence of the fatty acid saturation on the partition behavior of Gb_3_ (Figure 4). The results show that introducing a fatty acid with a *cis*‐double bond redistributes the Gb_3_ sphingolipid in the *l*_d_ phase, and can be rationalized by the increased space requirement of the Gb_3_ species with the C_24:1_ fatty acid. The differences between the *l*_o_ distribution of **1**/**5** and **3**/**7** harboring the PEG_13_ linker are significant and range between 0.21 and 0.27 (Table 2, ΔC24).

Björkqvist et al.^40^ investigated different glycosphingolipids as well as sphingomyelins and found by differential scanning calorimetry (DSC) that the phase‐transition temperature was decreased by about 20 K for all sphingolipids harboring the C_24:1_ fatty acid compared to the corresponding C_24:0_ sphingolipids, demonstrating their different packing behavior. The ability to pack tightly with ordered acyl chains in case of a saturated fatty acid^41^ is a requirement for membrane lipids to partition into *l*_o_ domains and they concluded that the C_24:1_ sphingolipids are less likely to partition into the *l*_o_ phase. Fluorescence quenching experiments revealed that sphingolipids with a C_24:0_ fatty acid form *l*_o_ domains in multicomponent membranes composed of either the sphingolipid or mixed with palmitoyl sphingomyelin.^40^ This behavior was also found by Mate et al.,^42^ who reported that sphingomyelin with the C_24:0_ fatty acid reconstituted into a DOPC/Chol membrane leads to visible phase separation into an *l*_o_ and *l*_d_ phase, while the sphingomyelin with the C_24:1_ fatty results only in one lipid phase.

These results support our notion that the packing of the unsaturated Gb_3_ species disfavors its partition in the *l*_o_ phase. Similar to our in vitro results, Legros et al.^43^ found in primary human blood brain barrier endothelial cells that Gb_3_ with C_24:1_ fatty acids resides more strongly in non‐detergent‐resistant membranes compared to Gb_3_ with C_24:0_ fatty acids.

**Figure 5:**
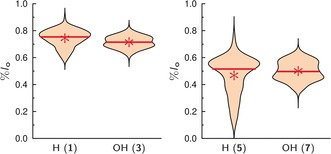
*l*_o_ distribution of different Gb_3_ species with a PEG_13_ linker in GUVs composed of DOPC/SM‐porc/Chol/Gb_3_/Dy731 (39/39/20/1/1). The partition of the Gb_3_ sphingolipids, which are nonhydroxylated in the α‐position (H) is compared with that carrying an α‐hydroxylation (OH). The mean values are given as red stars, while the red solid lines are the median values (Table 1).

In nature, about 50 % of the Gb_3_ sphingolipids are decorated with an OH group in the α‐position of the fatty acid, raising the question, whether this OH group alters the Gb_3_ partition. The results (Figure 5) clearly indicate that the OH group in the α‐position does not influence its distribution. The differences of %*l*_o_ for **1**/**3** and **5**/**7** are in the range of −0.03–0.03 and are not significant (Table 2, ΔOH).

Monolayer experiments on galactosyl ceramide (GalCer), harboring either an α‐hydroxylated or nonhydroxylated C_24:0_ fatty acid on a Langmuir trough, suggest that the α‐hydroxylation does not change the area per lipid at 30 mN m^−1^,^44^ a surface pressure that reflects the packing density of bilayers.^45^ Using ^2^H NMR spectroscopy, Morrow and co‐workers^41^, ^46^ also demonstrated that the order parameter of the fatty acids of GalCer embedded in a POPC/Chol membrane and the orientation of the head group does not change considerably.

This report is in line with our observation that the OH group does not significantly alter the partitioning of the Gb_3_ species in phase‐separated GUVs. However, in a previous study, we found that the 2‐OH group influences the fraction of *l*_o_ phase in phase‐separated supported lipid bilayers.^21^ In the case of the hydroxylated C_24:0_ fatty acid, the *l*_o_ fraction was smaller than that of the nonhydroxylated species. Slotte and co‐workers^47^ showed that the 2‐OH group increases the hydration in the membrane interface and decreases the affinity of a sphingolipid for sterols. The same was found by Lingwood et al.^48^ and Yahi et al.^49^ and implies that the recruitment of Chol into the *l*_o_ phase by hydroxylated Gb_3_ is reduced compared to the nonhydroxylated species, leading to a smaller *l*_o_ fraction, while the amount of Gb_3_ in the *l*_o_ fraction is the same.

**Figure 6:**
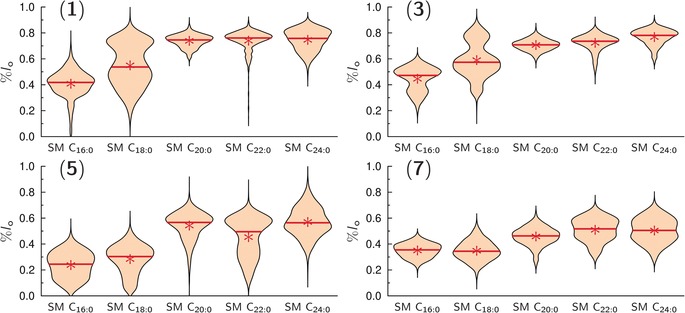
*l*_o_ distribution of different Gb_3_ species with a PEG_13_ linker composed of DOPC/SM/Chol/Gb_3_/Dy731 (39/39/20/1/1). The partition of four different Gb_3_ sphingolipids in *l*_o_/*l*_d_ phase‐separated GUVs with different sphingomyelin species are shown: SM C_16:0_ (palmitoyl SM), SM C_18:0_ (stearoyl SM), SM C_20:0_ (arachidoyl SM), SM C_22:0_ (behenoyl SM) and SM C_24:0_ (lignoceroyl SM). The mean values are given as red stars and the solid red lines represent the median value(Table 3).

Our results clearly demonstrate that the fatty acid of Gb_3_ influences its partitioning into the *l*_o_ phase. One reason might be found in the interaction of the Gb_3_ fatty acid with the fatty acid of SM, which we next analyzed. To investigate this aspect in more detail, we replaced the SM mixture isolated from pigs with synthetic pure SM. Exchanging a sphingomyelin mixture with sphingomyelins with a defined fatty acid is known to alter the phase separation behavior of ternary mixtures.^50^ Five different SM species with a saturated fatty acid of varying length were chosen, namely palmitoyl SM (C_16:0_), stearoyl SM (C_18:0_), arachidoyl SM (C_20:0_), behenoyl SM (C_22:0_), and lignoceroyl SM (C_24:0_), and the *l*_o_ distribution of each Gb_3_ species in these membranes was determined (Figure 6, Table 3).

**Table 3:** Mean values of the *l*_o_ distributions (%*l*_o_) for the different Gb_3_ sphingolipids with the PEG_13_ linker in GUVs composed of DOPC/SM/Chol/Gb_3_/Dy731 (39/39/20/1/1) varying in the SM species.

Gb_3_	%*l*_o_(SM C_16:0_) (*N*)	%*l*_o_(SM C_18:0_) (*N*)	%*l*_o_(SM C_20:0_) (*N*)	%*l*_o_(SM C_22:0_) (*N*)	%*l*_o_(SM C_24:0_) (*N*)
1	0.41±0.11 (2392)	0.55±0.17 (2986)	0.74±0.05 (2397)	0.74±0.09 (2077)	0.75±0.09 (1707)
3	0.45±0.10 (3232)	0.59±0.14 (3396)	0.71±0.05 (2414)	0.72±0.07 (2893)	0.77±0.07 (2509)
5	0.24±0.10 (2035)	0.28±0.11 (1845)	0.54±0.11 (2482)	0.45±0.15 (2759)	0.57±0.12 (1730)
7	0.35±0.06 (1814)	0.34±0.08 (2465)	0.46±0.08 (2648)	0.51±0.09 (2266)	0.50±0.10 (2491)

SM C_16:0_ (palmitoyl SM), SM C_18:0_ (stearoyl SM), SM C_20:0_ (arachidoyl SM), SM C_22:0_ (behenoyl SM), and SM C_24:0_ (lignoceroyl SM). The errors are the standard deviation of the mean. *N*=number of line profiles.

The fatty‐acid chain length also determines the length difference between the two hydrophobic chains, which increases with an increase in fatty‐acid chain length. This mismatch results in interdigitation of both leaflets,^51^ which was—for fatty acids with a length of more than 20 carbon atoms—not only observed in the gel phase but also in the liquid‐crystalline phase.^52^, ^53^ Interdigitation was also reported for glycosphingolipids carrying a C_24_ fatty acid.^53^, ^54^ Hence, it is likely that the Gb_3_ species under investigation preferentially partition into the *l*_o_ phase if SM interdigitates. Interdigitation of SM in the liquid‐crystalline phase occurs for C_20_ fatty acids and longer, in agreement with our observation that the partition in the *l*_o_ phase is increased for SM species with C_20_ fatty acids or longer.

However, the *l*_o_ phase consists not only of SM but also of Chol owing to its better solubility in SM membranes than in PC membranes.^55^, ^56^, ^57^ Chol is best soluble in SM C_16:0_.^56^, ^57^ If the solubility of Chol in the *l*_o_ phase greatly influenced the Gb_3_ distribution in the *l*_o_ phase, the opposite trend would have been observed. This trend was not found and agrees with the idea that the interaction of Gb_3_ with Chol is less important than the one with SM.

## Conclusion

G_M1_ and Gb_3_ detection by fluorescently labeled Cholera toxin B subunits (CTxB) and Shiga toxin B subunits (STxB), respectively is a well‐established tool for monitoring *l*_o_ membrane domains^58^ and implies that these glycosphingolipids are localized in the *l*_o_ phase. However, as each CTxB and STxB pentamer can recruit a maximum of 5 (CTx) or 15 (STx) receptor lipids, the glycosphingolipid partitioning in coexisting *l*_o_/*l*_d_ membranes after protein binding does not necessarily reflect the situation prior protein binding. Hence, to be able to quantify the partitioning of Gb_3_ in phase coexisting *l*_o_/*l*_d_ membranes by means of fluorescence readout, chemical access to fluorescently labeled pure Gb_3_ molecules is required. The approach of synthesizing head group labeled glycosphingolipids enables one to address the question how the fatty acid of a glycosphingolipid influences its distribution in *l*_o_/*l*_d_ phase‐separated membranes, a question that has been hardly addressed because most of the glycosphingolipids are not available in chemically pure form. Our results clearly demonstrate that the fatty acid (un)saturation significantly shifts the Gb_3_ molecules from the *l*_o_ phase (C_24:0_) to the *l*_d_ phase (C_24:1_). As STxB exclusively binds to Gb_3_ in the *l*_o_ phase, the amount of redistributed Gb_3_ and probably also other *l*_o_ phase lipids thus depends on the fatty acid of Gb_3_. However, the α‐hydroxylation does not alter the partition of Gb_3_, even though it has been shown that the OH group of Chol can form a hydrogen bond only to the nonhydroxylated fatty acid. Instead, the length match of the fatty acids of SM and Gb_3_ appear to play a more decisive role in determining where the Gb_3_ glycosphingolipids are preferentially localized. As the combination of the attached fatty acids of SM and Gb_3_ considerably impacts the distribution of the Gb_3_ glycosphingolipids, it is conceivable that the overall recruitment of lipids and thus the Shiga toxin induced membrane reorganization that eventually leads to the invagination of the protein into the host cell, is strongly influenced by the fatty acid composition of Gb_3_.

## Conflict of interest

The authors declare no conflict of interest.

## Supporting information

As a service to our authors and readers, this journal provides supporting information supplied by the authors. Such materials are peer reviewed and may be re‐organized for online delivery, but are not copy‐edited or typeset. Technical support issues arising from supporting information (other than missing files) should be addressed to the authors.

Supplementary
