# Supplementary material for: Synthesis of Gb3 Glycosphingolipids with Labeled Head Groups: Distribution in Phase‐Separated Giant Unilamellar Vesicles
Source: Angew Chem Int Ed Engl. 2019 Oct 21;58(49):17805–13. doi: 10.1002/anie.201910148 (PMC6899692; doi:10.1002/anie.201910148)
Supplement: Supplementary file 1 — Supplementary [file ANIE-58-17805-s001.pdf]

Angewandte Chemie
Eine Zeitschrift der Gesellschaft Deutscher Chemiker

Supporting Information

Synthesis of Gb_3 Glycosphingolipids with Labeled Head Groups: Distribution in Phase-Separated Giant Unilamellar Vesicles

Jeremias Sibold[+], Katharina Kettelhoit[+], Loan Vuong, Fangyuan Liu, Daniel B. Werz, and Claudia Steinem**

anie_201910148_sm_miscellaneous_information.pdf

Table of Contents

General experimental (chemical synthesis) S2

General procedures S3

Synthesis of fluorescently labeled Gb_3 species S4

Synthesis of azidosphingosine **10** S47

NMR spectra S48

Materials S123

Preparation of giant unilamellar vesicles (GUVs) S123

Fluorescence microscopy of GUVs S123

Self-quenching of BODIPY labeled Gb_3 .... S123

l_o distribution of different Gb_3 species with a PEG_3 linker (SM-porc) S124

l_o distribution of different Gb_3 species with a PEG_3 linker (chemically defined SM) S125

References S126

General experimental (chemical synthesis)

Air and moisture sensitive reactions were carried out in oven-dried or flame-dried glassware, septum-capped under atmospheric pressure or argon. The solvents were dried by standard procedures and distilled prior to use. If necessary, solvents were degassed via freeze-pump-thaw technique. Commercially available compounds were used without further purification unless otherwise stated. Dye **9** was purchased from Lumiprobe/Germany and PEG linker **23** from Broadpharm/USA. Proton (^{1}H), carbon (^{13}C) and fluorine (^{19}F) NMR spectra were recorded on a 300, 400, 500 or 600 MHz instrument using the residual signals from tetramethyl silane (TMS) δ = 0.00 ppm as internal references for ^{1}H and ^{13}C chemical shifts, respectively. Mass spectrometry was carried out on a FT-ICR instrument. IR spectra were measured on an ATR spectrometer. Optical rotations were obtained using a common polarimeter. Dialysis was performed in deionized water using cellulose ester tubing with a molecular weight cut-off of 100-500 g/mol. Gel permeation HPLC was performed in chloroform using a recycling type system.

General procedure 1 (GP1): Synthesis of trichloroacetimidates

To a solution of the fully protected sugar in dry dichloromethane was added trifluoroacetic acid at 0 °C. The reaction was stopped by adding dichloromethane and water. The organic layer was washed with diluted aq. $NaHCO_3$ solution (3×) and sat. aq. NaCl solution until pH 7 was reached, dried over Na_2SO_4, filtered and the solvent was removed under reduced pressure. The reducing sugar was dissolved in dry dichloromethane, the solution was cooled to 0 °C and trichloroacetonitrile and DBU were added. The reaction mixture was stirred at 0 °C until completion of the reaction and all volatiles were removed under reduced pressure.

General procedure 2 (GP2): Glycosylation with azidosphingosine 10

The trichloroacetimidate and alcohol **10** were azeotroped with toluene (3×), dried in high vacuum for at least 1 h and dissolved in dry dichloromethane. Molecular sieves (3 Å) were added and the mixture was stirred at ambient temperature for 20 min. Afterwards, TMSOTf was added dropwise at 0 °C and the reaction mixture was allowed to slowly warm to ambient temperature. The reaction was quenched by adding pyridine, the molecular sieves were filtered off through cotton and the solvent was removed under reduced pressure.

General procedure 3 (GP3): *Staudinger* reduction/acylation

To a solution of the azide in benzene were added triphenylphosphine and water and the suspension was heated to 60 °C (oil bath temperature). The solvents were removed under reduced pressure, the residue was azeotroped with toluene (3×) and dried in high vacuum for at least 1 h. The amine and the fatty acid were dissolved in dry THF and DIPEA was added. Afterwards, a solution of HATU in dry DMF was added dropwise at ambient temperature. The reaction was quenched by adding ethyl acetate and sat. aq. NaCl solution. The organic layer was washed with sat. aq. NaCl solution (2×) and the combined aqueous phases were reextracted with ethyl acetate. The combined organic layers were dried over Na_2SO_4, filtered and concentrated under reduced pressure.

General procedure 4 (GP4): *Zemplén* deprotection

To a solution of the protected carbohydrate in methanol/dichloromethane (3:1) a solution of sodium methoxide (5.4 M in MeOH) was added at ambient temperature until a pH value >12 was reached. The reaction was stirred at ambient temperature or at 50 °C, respectively, then neutralized with Amberlite®, filtered and concentrated in vacuo.

General procedure 5 (GP5): *Huisgen* cycloaddition

Glycolipid, dye **9** and Cu were added to a solution of water and *t*-butanol, an aqueous solution of $CuSO_4 \cdot 5\,H_2O$ was added, and the reaction mixture was stirred for 2 d at ambient temperature. The suspension was filtered, and the solvent was removed under reduced pressure.

Synthesis of fluorescently labeled Gb3 species

2-(Trimethylsilyl)ethyl-3,6-di-*O*-benzyl-2-*O*-(9-fluorenylmethoxycarbonyl)-β-D-galactopyranosyl-(1→4)-2,3,6-tri-*O*-benzyl-β-D-glucopyranoside (33)

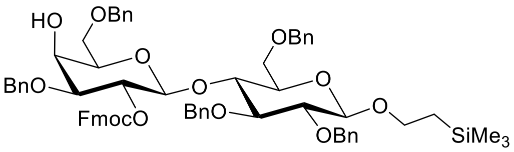

Glucopyranoside **19**[1] (100 mg, 0.182 mmol, 1.00 eq.) and galactosyl phosphate **18**[2] (195 mg, 0.218 mmol, 1.20 eq.) were azeotroped with toluene (3× 3.0 mL) and dried in high vacuo for 1 h. This mixture was dissolved in anhydrous dichloromethane (3.0 mL) and cooled to −40 °C. Then, TMSOTf (39.5 µL, 48.5 mg, 0.218 mmol, 1.20 eq.) was added dropwise. The reaction mixture was at −40 °C for 2 h. Afterwards the reaction was quenched by the addition of pyridine (0.5 mL), and the solvents were removed under reduced pressure. Column chromatography on silica gel (*n*-pentane/EtOAc, 8:1 → 5:1 → 4:1) afforded disaccharide **33** (132 mg, 0.118 mmol, 65 %) as a colorless foam.

$[\alpha]_D^{23}$ = +20.7 (*c* 1.55, $CHCl_3$).

^{1}H-NMR (600 MHz, $CDCl_3$): *δ* (ppm) = 0.04 (s, 9 H), 0.98–1.10 (m, 2 H), 2.35 (s, 1 H), 3.17 (ddd, *J* = 9.9, 3.7, 1.6 Hz, 1 H), 3.20–3.26 (m, 2 H), 3.33–3.39 (m, 2 H), 3.48–3.55 (m, 2 H), 3.57–3.63 (m, 2 H), 3.65 (dd, *J* = 11.1, 3.8 Hz, 1 H), 3.88 (dd, *J* = 9.9, 8.9 Hz, 1 H), 3.97–3.99 (m, 1 H), 4.02 (ddd, *J* = 11.0, 9.5, 6.8 Hz, 1 H), 4.21 (t, *J* = 6.5 Hz, 1 H), 4.30–4.34 (m, 2 H), 4.34 (d, *J* = 12.0 Hz, 1 H), 4.38 (d, *J* = 12.0 Hz, 1 H), 4.44 (d, *J* = 8.0 Hz, 1 H), 4.49 (dd, *J*= 10.8, 6.5 Hz, 1 H), 4.49 (d, *J* = 11.8 Hz, 1 H), 4.55 (dd, *J* = 10.6, 6.5 Hz, 1 H), 4.59 (d, *J* = 12.1 Hz, 1 H), 4.62 (d, *J* = 11.8 Hz, 1 H), 4.70 (d, *J* = 11.1 Hz, 1 H), 4.73 (d, *J* = 10.9 Hz, 1 H), 4.86–4.93 (m, 2 H), 4.93 (d, *J* = 10.9 Hz, 1 H), 7.13–7.35 (m, 27 H), 7.34–7.44 (m, 2 H), 7.59–7.61 (m, 2 H), 7.77 (ddt, *J* = 7.6, 4.9, 0.9 Hz, 2 H).

^{13}C-NMR (151 MHz, $CDCl_3$): *δ* (ppm) = −1.4, 18.5, 46.9, 65.5, 67.4, 67.9, 67.9, 69.4, 71.4, 72.8, 73.1, 73.5, 74.6, 74.9, 75.4, 75.8, 77.1, 78.8, 81.9, 82.8, 100.3, 103.1, 120.1, 120.1, 124.8, 124.9, 127.1, 127.1, 127.4, 127.5, 127.5, 127.6, 127.7, 127.8, 127.8, 127.9, 128.0, 128.0, 128.0, 128.2, 128.2, 128.4, 128.5, 137.4, 138.1, 138.3, 138.7, 139.2, 141.3, 141.4, 143.3, 143.5, 154.5.

IR (ATR): $\tilde{\nu}$ (cm^{-1}) = 3064, 3030, 2951, 2872, 1754, 1452, 1363, 1248, 1070, 986, 858, 836, 733, 696.

MS (ESI): *m*/*z* (%) = 1137.5 (100) $[M+Na]^+$. **HRMS** (ESI): *m*/*z* calculated for: $C_{87}H_{74}O_{13}Si$ $[M+Na]^+$: 1137.4791, found: 1137.4793.

2-(Trimethylsilyl)ethyl-2,4,3,6-tetra-*O*-benzyl-α-D-galactopyranosyl-(1→4)-3,6-di-*O*-benzyl-2-*O*-(9-fluorenylmethoxycarbonyl)-β-D-galactopyranosyl-(1→4)-2,3,6-tri-*O*-benzyl-β-D-glucopyranoside (34)

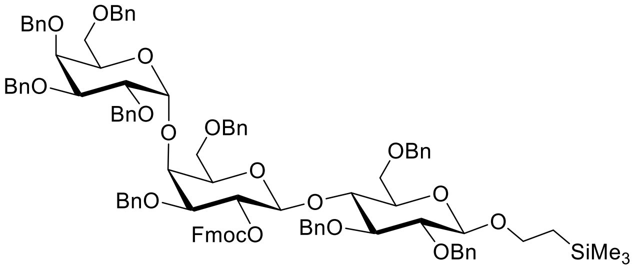

Disaccharide **33** (1.55 g, 1.39 mmol, 1.00 eq.) and trichloroacetimidate **17**[3] (1.24 g, 1.81 mmol, 1.30 eq.) were azeotroped with toluene (3× 20 mL) and dried in high vacuo for 1 h. The mixture was dissolved in anhydrous dichloromethane (44 mL) and diethyl ether (11 mL) and was cooled to −20 °C. TMSOTf (38.0 µL, 46.5 mg, 0.209 mmol, 15.0 mol%) was added. The reaction mixture was stirred at this temperature for 3 h. The reaction was stopped by the addition of pyridine (1.0 mL). The solvents were removed under reduced pressure. Column chromatography on silica gel (*n*-pentane/EtOAc, 8:1 → 6:1 → 5:1) afforded the target compound **34** (1.69 g, 1.03 mmol, 74 %) and the β-linked diastereomer (338 mg, 0.206 mmol, 15 %) as colorless oils.

$[\alpha]_D^{24}$ = +26.2 (*c* 1.09, $CHCl_3$).

^{1}H-NMR (600 MHz, $CDCl_3$): *δ* (ppm) = 0.03 (s, 9 H), 0.99–1.09 (m, 2 H), 3.07 (dd, *J* = 8.9, 4.7 Hz, 1 H), 3.19–3.28 (m, 3 H), 3.33–3.40 (m, 2 H), 3.50 (t, *J* = 8.9 Hz, 1 H), 3.55 (t, *J* = 9.2 Hz, 1 H), 3.57–3.65 (m, 2 H), 3.73 (dd, *J* = 11.0, 4.0 Hz, 1 H), 3.90 (t, *J* = 9.2 Hz, 1 H), 3.97 (d, *J* = 11.7 Hz, 1 H), 4.00–4.06 (m, 2 H), 4.05–4.11 (m, 3 H), 4.11–4.18 (m, 3 H), 4.18–4.23 (m, 2 H), 4.29–4.38 (m, 3 H), 4.38–4.46 (m, 3 H), 4.45–4.52 (m, 2 H), 4.53–4.61 (m, 3 H), 4.68 (d, *J* = 11.0 Hz, 1 H), 4.70–4.79 (m, 4 H), 4.82 (d, *J* = 11.1 Hz, 1 H), 4.87 (d, *J* = 11.0 Hz, 1 H), 4.96–5.07 (m, 3 H), 7.08–7.23 (m, 29 H), 7.22–7.33 (m, 16 H), 7.34–7.44 (m, 4 H), 7.54–7.58 (m, 2 H), 7.76–7.80 (m, 2 H).

^{13}C-NMR (151 MHz, $CDCl_3$): *δ* (ppm) = −1.4, 18.5, 46.8, 67.2, 67.4, 67.6, 68.1, 69.0, 69.4, 71.6, 72.4, 72.9, 72.9, 73.1, 73.4, 73.7, 74.3, 74.7, 74.8, 74.9, 74.9, 75.3, 75.8, 76.2, 77.6, 79.5, 79.6, 81.9, 82.7, 100.8, 101.2, 103.1, 120.1, 120.1, 124.9, 125.0, 127.1, 127.2, 127.2, 127.2, 127.3, 127.4, 127.5, 127.5, 127.6, 127.7, 127.7, 127.9, 128.0, 128.0, 128.1, 128.1, 128.1, 128.1, 128.2, 128.2, 128.4, 138.1, 138.3, 138.3, 138.4, 138.7, 138.7, 138.7, 139.0, 139.1, 141.3, 141.4, 143.3, 143.5, 154.5.

IR (ATR): $\tilde{\nu}$ (cm^{-1}) = 3373, 3030, 2949, 2870, 1756, 1730, 1695, 1496, 1384, 1252, 1091, 1048, 833, 734, 695, 619.

MS (ESI): *m*/*z* (%) = 841.9 (100) $[M+2Na]^{2+}$, 1660.7 (81) $[M+Na]^+$. **HRMS** (ESI): *m*/*z* calculated for: $C_{101}H_{108}O_{18}Si$ $[M+Na]^+$: 1659.7197, found: 1659.7195.

2-(Trimethylsilyl)ethyl-2,4,3,6-tetra-*O*-benzyl-α-D-galactopyranosyl-(1→4)-3,6-di-*O*-benzyl-β-D-galactopyranosyl-(1→4)-2,3,6-tri-*O*-benzyl-β-D-glucopyranoside (20)

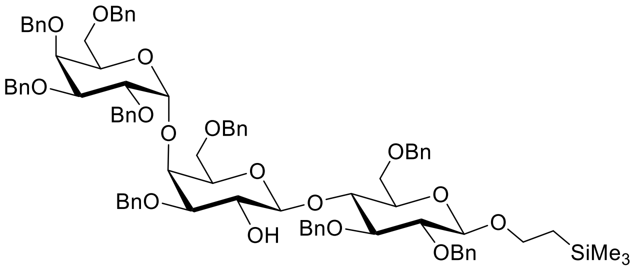

To a solution of fully protected trisaccharide **34** (862 mg, 0.526 mmol, 1.00 eq.) in anhydrous DMF (10.5 mL) was added piperidine (4.15 mL, 3.58 g, 42.1 mmol, 80.0 eq.) at ambient temperature. The reaction mixture was stirred for 20 h. The solvent was removed under reduced pressure. Column chromatography on silica gel (*n*-pentane/EtOAc, 10:1 → 4:1 → 3:1) afforded the target compound **20** (719 mg, 0.508 mmol, 97 %) as colorless foam.

$[\alpha]_D^{23}$ = +41.7 (*c* 0.71, $CHCl_3$).

^{1}H-NMR (600 MHz, $CDCl_3$): *δ* (ppm) = 0.03 (s, 9 H), 1.03 (m, 2 H), 3.14–3.23 (m, 4 H), 3.25 (dd, *J* = 8.5, 6.1 Hz, 1 H), 3.38 (dd, *J* = 9.1, 7.9 Hz, 1 H), 3.47–3.53 (m, 2 H), 3.60 (ddd, *J* = 10.8, 9.5, 5.9 Hz, 1 H), 3.65 (t, *J* = 9.1 Hz, 1 H), 3.75 (dd, *J* = 10.0, 7.6 Hz, 1 H), 3.84 (dd, *J* = 11.5, 1.9 Hz, 1 H), 3.94–4.03 (m, 5 H), 4.03–4.08 (m, 3 H), 4.08–4.14 (m, 3 H), 4.37 (d, *J* = 7.9 Hz, 1 H), 4.35–4.41 (m, 1 H), 4.45 (d, *J* = 12.7 Hz, 1 H), 4.48 (d, *J* = 11.2 Hz, 1 H), 4.56 (d, *J* = 7.6 Hz, 1 H), 4.56–4.63 (m, 3 H), 4.65 (d, *J* = 11.0 Hz, 1 H), 4.68 (d, *J* = 12.1 Hz, 1 H), 4.68 (d, *J* = 12.0 Hz, 1 H), 4.77 (d, *J* = 12.0 Hz, 1 H), 4.78 (d, *J* = 12.7 Hz, 1 H), 4.84–4.90 (m, 3 H), 4.97 (d, *J* = 11.4 Hz, 1 H), 4.99 (d, *J* = 3.3 Hz, 1 H), 7.05–7.40 (m, 45 H).

^{13}C-NMR (151 MHz, $CDCl_3$): *δ* (ppm) = −1.4, 18.5, 67.3, 67.5, 67.8, 68.7, 69.2, 71.8, 72.4, 72.9, 73.1, 73.4, 73.6, 73.8, 73.8, 74.5, 74.9, 74.9, 76.4, 77.4, 79.3, 80.8, 82.3, 83.4, 100.7, 103.3, 104.0, 127.1, 127.2, 127.2, 127.2, 127.3, 127.4, 127.4, 127.5, 127.5, 127.6, 127.6, 127.8, 127.9, 128.0, 128.1, 128.1, 128.2, 128.2, 128.2, 128.2, 128.3, 128.3, 128.3, 128.4, 138.0, 138.1, 138.2, 138.5, 138.5, 138.7, 138.8, 139.0, 139.2.

IR (ATR): $\tilde{\nu}$ (cm^{-1}) = 3478, 3063, 2922, 2870, 1496, 1453, 1361, 1248, 1208, 1086, 1052, 910, 858, 733, 695.

MS (ESI): *m*/*z* (%) = 730.3 (85) $[M+2Na]^{2+}$, 1438.7 (100) $[M+Na]^+$. **HRMS** (ESI): *m*/*z* calculated for: $C_{86}H_{98}SiO_{16}$ $[M+Na]^+$: 1437.6516, found: 1437.6516.

2-(Trimethylsilyl)ethyl-2,4,3,6-tetra-*O*-benzyl-α-D-galactopyranosyl-(1→4)-3,6-di-*O*-benzyl-2-*O*-pent-4-ene-1-yl-β-D-galactopyranosyl-(1→4)-2,3,6-tri-*O*-benzyl-β-D-glucopyranoside (35)

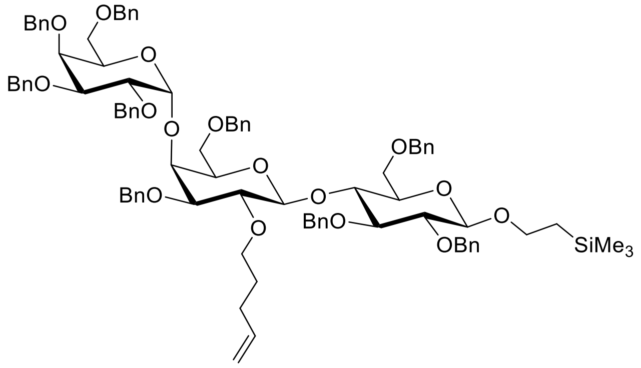

Sodium hydride (60 % in mineral oil, 178 mg, 4.45 mmol, 5.00 eq.) was washed with anhydrous cyclohexane (2× 10 mL). A solution of trisaccharide **20** (1.26 g, 0.890 mmol, 1.00 eq.) in anhydrous DMF (37.0 mL) which had been cooled to 0 °C was added. After 30 min 5-bromo-1-pentene (527 µL, 663 mg, 4.45 mmol, 5.00 eq.) was added dropwise at 0 °C, a tiny amount of tetrabutylammonium iodide was added and the reaction mixture was stirred for 22 h at ambient temperature. The reaction was quenched by the addition of ethyl acetate (100 mL) and water (100 mL); the organic phase was washed with brine (2× 100 mL), dried over Na_2SO_4, filtered and concentrated in vacuo. The crude product was purified by column chromatography on silica gel (*n*-pentane/EtOAc, 5:1 → 3:1) and the target compound **35** (1.17 g, 0.788 mmol, 89 %) was obtained as a colorless oil.

$[\alpha]_D^{23}$ = +35.2 (*c* 0.91, $CHCl_3$).

^{1}H-NMR (600 MHz, $CDCl_3$): *δ* (ppm) = 0.02 (s, 9 H), 0.93–1.08 (m, 2 H), 1.59–1.68 (m, 2 H), 1.99–2.11 (m, 2 H), 3.13 (dd, *J* = 8.3, 4.6 Hz, 1 H), 3.20 (dd, *J* = 10.0, 2.8 Hz, 1 H), 3.28 (dd, *J* = 8.4, 5.2 Hz, 1 H), 3.36 (dd, *J* = 9.3, 7.9 Hz, 1 H), 3.37–3.42 (m, 2 H), 3.44–3.51 (m, 2 H), 3.51–3.61 (m, 2 H), 3.69 (dt, *J* = 9.2, 6.8 Hz, 1 H), 3.75 (dt, *J* = 8.9, 6.8 Hz, 1 H), 3.84–3.93 (m, 3 H), 3.93–4.02 (m, 4 H), 4.02–4.06 (m, 2 H), 4.10 (d, *J* = 11.5 Hz, 1 H), 4.11–4.18 (m, 1 H), 4.22 (d, *J* = 11.8 Hz, 1H), 4.26 (d, *J* = 11.8 Hz, 1 H), 4.33 (dd, *J* = 9.3, 4.6 Hz, 1 H), 4.37 (d, *J* = 7.9 Hz, 1 H), 4.39 (d, *J* = 7.7 Hz, 1 H), 4.43 (d, *J* = 11.5 Hz, 1 H), 4.43–4.51 (m, 4 H), 4.67 (d, *J* = 12.0 Hz, 1 H), 4.68 (d, *J* = 11.0 Hz, 1 H), 4.69–4.77 (m, 4 H), 4.84 (d, *J* = 11.3 Hz, 1 H), 4.87 (d, *J* = 11.0 Hz, 1 H), 4.89–4.95 (m, 1 H), 5.00 (ddd, *J* = 17.2, 3.4, 1.6 Hz, 1 H), 5.03 (d, *J* = 3.4 Hz, 1 H), 5.06 (d, *J* = 11.0 Hz, 1 H), 5.73–5.83 (m, 1 H), 7.03–7.42 (m, 45 H).

^{13}C-NMR (151 MHz, $CDCl_3$): *δ* (ppm) = −1.6, 18.3, 29.4, 29.5, 30.1, 67.2, 67.5, 67.6, 68.2, 69.1, 71.9, 72.2, 72.7, 72.8, 72.9, 73.0, 73.4, 74.6, 74.6, 74.7, 74.8, 75.0, 75.1, 76.4, 77.0, 77.0, 79.1, 79.5, 81.4, 81.6, 82.5, 100.5, 102.7, 102.9, 114.4, 126.9, 127.1, 127.1, 127.2, 127.2, 127.2, 127.2, 127.3, 127.3, 127.4, 127.6, 127.9, 127.9, 127.9, 127.9, 127.9, 128.0, 128.0, 128.0, 128.0, 128.1, 128.1, 128.2, 137.9, 138.2, 138.2, 138.3, 138.5, 138.6, 138.6, 138.7, 138.9.

IR (ATR): $\tilde{\nu}$ (cm^{-1}) = 3063, 2921, 2868, 1496, 1453, 1361, 1091, 1072, 1051, 859, 836, 732, 696.

MS (ESI): *m*/*z* (%) = 764.9 (67) [M+2Na]$^{2+}$, 1506.7 (100) [M+Na]$^{+}$. **HRMS** (ESI): *m*/*z* calculated for: $C_{91}H_{106}O_{16}Si$ [M+Na]$^{+}$: 1505.7142, found: 1505.7143.

2-(Trimethylsilyl)ethyl-2,4,3,6-tetra-*O*-benzoyl-α-D-galactopyranosyl-(1→4)-3,6-di-*O*-benzoyl-2-*O*-pent-4-ene-1-yl-β-D-galactopyranosyl-(1→4)-2,3,6-tri-*O*-benzoyl-β-D-glucopyranoside (36)

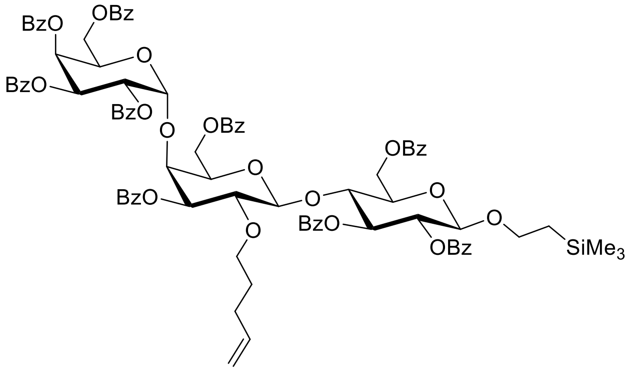

Into a three-necked round bottom flask equipped with dry ice reflux condenser and stirring bar covered with glass ammonia was condensed at −60 °C. Tiny pieces of sodium (*ACROS*, 99.8 %) which had been washed before with *n*-pentane were added until the blue color of the solution remained. By careful and slow warming the pre-dried ammonia was transferred (evaporation and condensation) into the reaction vessel and cooled to −60 °C. Again, tiny pieces of sodium were added until the blue color remained. Then a solution of trisaccharide **35** (85.0 mg, 57.3 μmol, 1.00 eq.) in anhydrous THF (12.0 mL) was added dropwise. In the case of disappearing color more sodium was added. After 15 min methanol was added dropwise until the blue color disappeared. After that sodium was added again (until the blue color remained); the reaction was stopped by the slow addition of methanol (several mL). The cooling bath and the dry ice cooling funnel were removed. The reaction was first placed in an ice bath; after most of the ammonia was evaporated, the ice bath was substituted by warm water to remove all traces of ammonia. The solution was neutralized with Amberlite® and filtered. Amberlite® was carefully washed with methanol. The solvents were removed under reduced pressure and the crude product was azeotroped with toluene (3× 5 mL) and dried in high vacuo for at least 1 h.

The deprotected trisaccharide was dissolved in anhydrous pyridine (1.2 mL), benzoyl chloride (99.2 μL, 121 mg, 0.860 mmol, 15.0 eq.) and DMAP (63.1 mg, 0.516 mmol, 9.00 eq.) were added at ambient temperature. After 24 h another portion benzoyl chloride (99.2 μL, 121 mg, 0.860 mmol, 15.0 eq.) was added and the reaction mixture was stirred for another 4 d. The reaction was stopped by adding EtOAc (25 mL) and brine (25 mL). The aqueous phase was extracted with EtOAc (25 mL), the combined organic phases were dried over Na_2SO_4, filtered and concentrated in vacuo. Gel permeation HPLC followed by subsequent purification by

column chromatography on silica gel (*n*-pentane/EtOAc, 3:1) yielded the target compound **36** (77.5 mg, 48.1 mmol, 84 %) as a colorless oil.

$[\alpha]_D^{24}$ = +60.0 (*c* 1.06, $CHCl_3$).

^{1}H-NMR (600 MHz, $CDCl_3$): *δ* (ppm) = −0.11 (s, 9 H), 0.79–0.93 (m, 2 H), 1.31–1.43 (m, 2 H), 1.67–1.77 (m, 1 H), 1.78–1.88 (m, 1 H), 3.49–3.55 (m, 1 H), 3.54–3.59 (m, 2 H), 3.65 (dd, *J* = 10.3, 7.5 Hz, 1 H), 3.69–3.74 (m, 1 H), 3.95 (ddd, *J* = 10.6, 9.6, 5.7 Hz, 1 H), 3.99 (ddd, *J* = 9.6, 5.4, 2.1 Hz, 1 H), 4.05–4.15 (m, 3 H), 4.18 (d, *J* = 3.0 Hz, 1 H), 4.24 (t, *J* = 9.6 Hz, 1 H), 4.40 (dd, *J* = 11.1, 5.6 Hz, 1 H), 4.57 (d, *J* = 7.5 Hz, 1 H), 4.59 (dd, *J* = 11.9, 5.4 Hz, 1 H), 4.58 (dd, *J* = 9.6, 7.0 Hz, 1 H), 4.63–4.69 (m_c, 1 H), 4.68–4.74 (m, 1 H), 4.75 (d, *J* = 7.9 Hz, 1 H), 4.92 (dd, *J* = 11.9, 2.1 Hz, 1 H), 5.04 (dd, *J* = 10.3, 3.0 Hz, 1 H), 5.42 (dd, *J* = 9.5, 7.9 Hz, 1 H), 5.46 (d, *J* = 3.6 Hz, 1 H), 5.45–5.55 (m, 1 H), 5.65 (dd, *J* = 10.9, 3.6 Hz, 1 H), 5.79 (t, *J* = 9.6 Hz, 1 H), 5.83 (dd, *J* = 10.9, 3.4 Hz, 1 H), 6.02 (dd, *J* = 3.4, 1.6 Hz, 1 H), 7.18–7.25 (m, 2 H), 7.30–7.45 (m, 18 H), 7.45–7.55 (m, 6 H), 7.71–7.85 (m, 6 H), 7.91–8.15 (m, 13 H)

^{13}C-NMR (75 MHz, $CDCl_3$): *δ* (ppm) = −1.4, 18.0, 29.3, 29.9, 61.5, 61.9, 63.0, 67.5, 67.7, 68.1, 69.2, 70.1, 72.4, 72.5, 73.1, 73.3, 73.6, 75.2, 75.5, 77.4, 77.7, 98.1, 100.5, 103.1, 114.6, 128.4, 128.5, 128.5, 128.5, 128.7, 128.8, 129.3, 129.3, 129.4, 129.5, 129.6, 129.7, 129.7, 129.8, 129.9, 130.0, 130.0, 130.0, 130.0, 133.1, 133.2, 133.2, 133.3, 133.3, 133.6, 133.7, 138.1, 165.3, 165.3, 165.3, 165.4, 165.7, 165.8, 166.0, 166.2.

IR (ATR): $\tilde{\nu}$ (cm^{-1}) = 2952, 2926, 2871, 1723, 1602, 1451, 1315, 1264, 1091, 1066, 1026, 856, 837, 706.

MS (ESI): *m*/*z* (%) = 827.8 (40) $[M+2Na]^{2+}$, 1632.5 (100) $[M+Na]^+$. **HRMS** (ESI): *m*/*z* calculated for: $C_{91}H_{88}O_{25}Si$ $[M+Na]^+$: 1631.5276, found: 1631.5276.

2-(Trimethylsilyl)ethyl-2,4,3,6-tetra-*O*-benzoyl-α-D-galactopyranosyl-(1→4)-3,6-di-*O*-benzoyl-2-*O*-(5-thioacetyl)pent-1-yl-β-D-galactopyranosyl-(1→4)-2,3,6-tri-*O*-benzoyl-β-D-glucopyranoside (37)

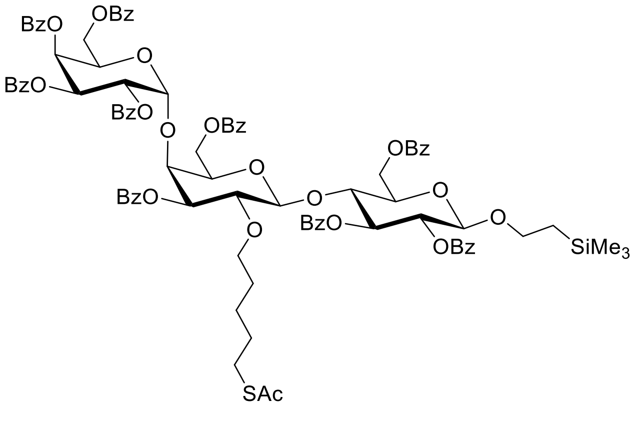

Trisaccharide **36** (99.0 mg, 61.4 µmol, 1.00 eq.) was dissolved in degassed THF (20 mL). Thioacetic acid (43.2 µL, 46.7 mg, 0.614 mmol, 10.0 eq.) and a tiny amount of recrystallized

AIBN were added. The reaction mixture was heated to reflux in a preheated oil bath (100 °C). After 6 h again a tiny amount of AIBN and thioacetic acid (43.2 µL, 46.7 mg, 0.614 mmol, 10.0 eq.) were added. In total the mixture was stirred for 24 h under reflux. Afterwards, the solvent was removed under reduced pressure. Column chromatography on silica gel (*n*-pentane/EtOAc, 3:1 → 2:1) afforded the target compound **37** (91.7 mg, 54.3 µmol, 88 %) as a colorless foam.

$[\boldsymbol{\alpha}]_{\mathbf{D}}^{\mathbf{21}}$ = +44.0 (*c* 1.18, $CHCl_3$).

^{1}H-NMR (600 MHz, $CDCl_3$): *δ* (ppm) = −0.11 (s, 9 H), 0.80–0.93 (m, 3 H), 1.15–1.37 (m, 5 H), 2.26 (s, 3 H), 2.46–2.58 (m, 2 H), 3.44 (dt, *J* = 9.2, 6.9 Hz, 1 H), 3.52–3.59 (m, 2 H), 3.62 (dd, *J* = 10.3, 7.5 Hz, 1 H), 3.70 (dt, *J* = 9.2, 6.1 Hz, 1 H), 3.95 (ddd, *J* = 10.7, 9.7, 5.6 Hz, 1 H), 4.00 (ddd, *J* = 9.6, 5.3, 2.2 Hz, 1 H), 4.06–4.18 (m, 4 H), 4.25 (t, *J* = 9.6 Hz, 1 H), 4.44 (dd, *J* = 11.2, 5.6 Hz, 1 H), 4.56 (d, *J* = 7.5 Hz, 1 H), 4.56 (dd, *J* = 11.9, 5.3 Hz, 1 H), 4.65 (ddd, *J* = 7.3, 5.6, 1.5 Hz, 1 H), 4.76 (d, *J* = 7.8 Hz, 1 H), 4.91 (dd, *J* = 11.9, 2.2 Hz, 1 H), 5.05 (dd, *J* = 10.3, 2.9 Hz, 1 H), 5.42 (dd, *J* = 9.5, 7.8 Hz, 1 H), 5.47 (d, *J* = 3.5 Hz, 1 H), 5.64 (dd, *J* = 10.9, 3.5 Hz, 1 H), 5.79 (t, *J* = 9.6 Hz, 1 H), 5.83 (dd, *J* = 10.9, 3.4 Hz, 1 H), 6.01 (dd, *J* = 3.4, 1.5 Hz, 1 H), 7.19–7.26 (m, 3 H), 7.31–7.46 (m, 18 H), 7.47–7.55 (m, 5 H), 7.73–7.87 (m, 6 H), 7.92–8.15 (m, 13 H).

^{13}C-NMR (151 MHz, $CDCl_3$): *δ* (ppm) = −1.5, 17.9, 25.2, 28.8, 29.1, 29.5, 30.6, 61.4, 61.8, 62.9, 67.4, 67.6, 67.9, 69.1, 69.9, 72.3, 72.4, 73.0, 73.4, 73.5, 74.8, 75.3, 76.5, 77.5, 97.9, 100.4, 102.9, 128.3, 128.4, 128.4, 128.4, 128.4, 128.5, 128.6, 128.7, 129.1, 129.2, 129.2, 129.4, 129.5, 129.6, 129.6, 129.6, 129.7, 129.8, 129.8, 129.9, 129.9, 133.0, 133.1, 133.1, 133.2, 133.2, 133.5, 133.6, 165.2, 165.2, 165.6, 165.9, 195.8.

IR (ATR): $\tilde{\nu}$ (cm^{-1}) = 3066, 2949, 2869, 1722, 1688, 1602, 1451, 1261, 1091, 1066, 1025, 855, 837, 705.

MS (ESI): *m*/*z* (%) = 865.8 (61) $[M+2Na]^{2+}$, 1708.5 (100) $[M+Na]^+$. **HRMS** (ESI): *m*/*z* calculated for: $C_{93}H_{92}O_{26}SiS$ $[M+Na]^+$: 1707.5259, found: 1707.5271.

2-(Trimethylsilyl)ethyl-2,4,3,6-tetra-*O*-benzoyl-α-D-galactopyranosyl-(1→4)-3,6-di-*O*-benzoyl-2-*O*-(9,12,15,18,21,24,27,30,33,36,39,42,45-tridecaoxa-6-thiaoctatetracont-47-yne-1-yl)-β-D-galactopyranosyl-(1→4)-2,3,6-tri-*O*-benzoyl-β-D-glucopyranoside (38)

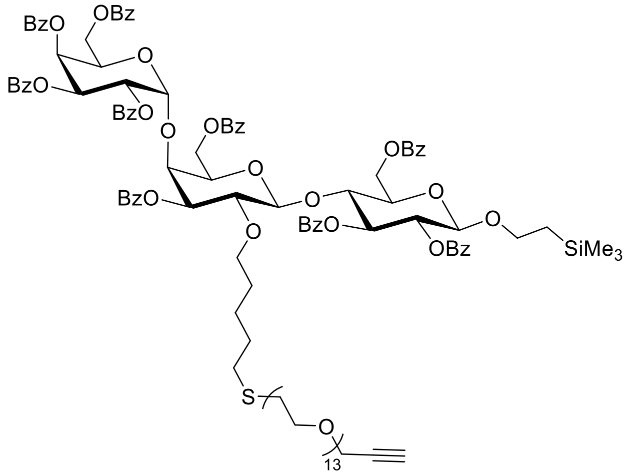

To a solution of thioester **37** (156 mg, 92.5 µmol, 1.00 eq.) in a mixture of degassed methanol (3.7 mL) and chloroform (1.2 mL) K_2CO_3 (12.8 mg, 92.5 µmol, 1.00 eq.) was added. The reaction mixture was stirred at ambient temperature for 1 h. The solution was neutralized by the addition of Amberlite®, filtered and concentrated in vacuo. The residue was taken up in water (5.8 mL) and a solution of bromide **21** (76.8 mg, 0.111 mmol, 1.20 eq.) in THF (11.6 mL) was added. Sodium hydroxide (0.5 M in H_2O, 185 µL, 92.5 µmol, 1.00 Äq) and a tiny amount of tetrabutylammonium iodide were added. The reaction mixture was stirred for 2 d at ambient temperature. The reaction mixture was neutralized by addition of Amberlite®, filtered and the solvents were removed under reduced pressure. The resulting product was azeotroped with toluene (3× 2.5 mL) and dried in high vacuo for 1 h. Pyridine (1.9 mL), benzoyl chloride (160 µL, 195 mg, 1.39 mmol, 15.0 eq.) and DMAP (56.6 mg, 0.463 mmol, 5.00 eq.) were added and the reaction mixture was stirred at ambient temperature for 1 d. Then again benzoyl chloride (160 µL, 195 mg, 1.39 mmol, 15.0 eq.) was added and the reaction mixture was stirred for further 4 d at ambient temperature. The reaction was stopped by the addition of ethyl acetate (50 mL) and water (50 mL). The organic phase was washed with brine (50 mL), dried over Na_2SO_4, filtered and concentrated in vacuo. Gel permeation HPLC and subsequent column chromatography on silica gel (*n*-pentane/EtOAc, 2:1 → CH_2Cl_2/MeOH, 30:1 → 20:1) afforded the target compound **38** (103 mg, 45.7 mmol, 49 %) as a colorless oil.

$[\alpha]_D^{21}$ = +38.9 (*c* 0.85, $CHCl_3$).

^{1}H-NMR (600 MHz, $CDCl_3$): *δ* (ppm) = −0.11 (s, 9 H), 0.79–0.93 (m, 3 H), 1.15–1.33 (m, 5 H), 2.11–2.22 (m, 2 H), 2.44 (t, *J* = 2.4 Hz, 1 H), 2.57 (t, *J* = 7.2 Hz, 2 H), 3.40–3.73 (m, 55 H), 3.91–3.99 (m, 1 H), 4.00 (ddd, *J* = 10.0, 5.3, 2.1 Hz, 1 H), 4.05–4.14 (m, 3 H), 4.16 (d, *J* = 2.9 Hz, 1 H), 4.20 (d, *J* = 2.4 Hz, 2 H), 4.24 (t, *J* = 9.5 Hz, 1 H), 4.44 (dd, *J* = 11.2, 5.6 Hz, 1 H), 4.51–4.59 (m, 2 H), 4.65 (t, *J* = 6.7 Hz, 1 H), 4.75 (d, *J* = 7.8 Hz, 1 H), 4.88–4.93 (m, 1 H), 5.05 (dd, *J* = 10.4, 2.8 Hz, 1 H), 5.42 (dd, *J* = 9.7, 7.8 Hz, 1 H), 5.47 (d, *J* = 3.5 Hz, 1 H), 5.64 (dd, *J* = 10.8,

3.4 Hz, 1 H), 5.79 (t, *J* = 9.4 Hz, 1 H), 5.82 (dd, *J* = 10.9, 3.4 Hz, 1 H), 6.01 (d, *J* = 3.7 Hz, 1 H), 7.17–7.25 (m, 4 H), 7.31–7.60 (m, 23 H), 7.73–7.86 (m, 6 H), 7.90–8.14 (m, 12 H).

^{13}C-NMR (151 MHz, $CDCl_3$): *δ* (ppm) = −1.5, 17.9, 25.3, 29.5, 29.7, 31.2, 32.1, 58.4, 61.4, 61.8, 62.9, 67.4, 67.6, 67.9, 69.1, 69.1, 69.9, 70.2, 70.4, 70.6, 70.8, 72.2, 72.4, 73.0, 73.4, 73.7, 74.6, 74.8, 75.3, 76.5, 77.6, 79.7, 97.9, 100.4, 103.0, 128.3, 128.4, 128.4, 128.4, 128.5, 128.6, 128.7, 129.1, 129.2, 129.2, 129.3, 129.5, 129.6, 129.6, 129.6, 129.8, 129.8, 129.9, 129.9, 133.0, 133.1, 133.1, 133.2, 133.2, 133.5, 133.7, 165.2, 165.2, 165.2, 165.3, 165.6, 165.7, 165.8, 165.9, 166.1.

IR (ATR): $\tilde{\nu}$ (cm^{-1}) = 2868, 1723, 1601, 1451, 1264, 1251, 1091, 1067, 1026, 855, 838, 708.

MS (ESI): *m*/*z* (%) = 1149.9 (100) $[M+2Na]^{2+}$, 2276.9 (84) $[M+Na]^+$. **HRMS** (ESI): *m*/*z* calculated for: $C_{120}H_{144}O_{38}SiS$ $[M+Na]^+$: 2275.8718, found: 2275.8722.

2-(Trimethylsilyl)ethyl-2,4,3,6-tetra-*O*-benzoyl-α-D-galactopyranosyl-(1→4)-3,6-di-*O*-benzoyl-2-*O*-(9,12,15-trioxa-6-thiaoctadec-17-yne)-1-yl-β-D-galactopyranosyl-(1→4)-2,3,6-tri-*O*-benzoyl-β-D-glucopyranoside (39)

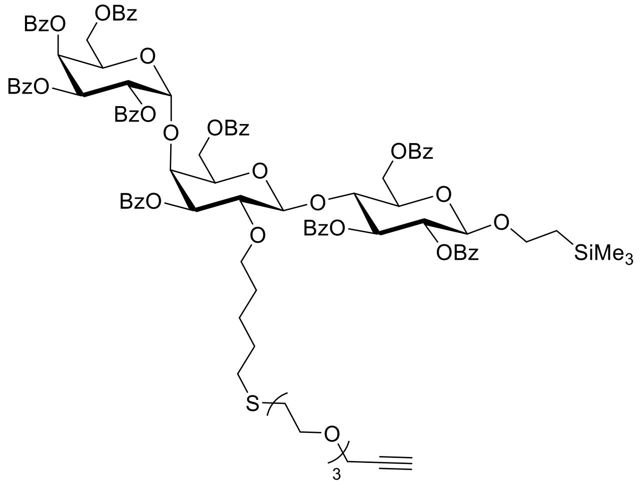

To a solution of thioester **37** (128 mg, 75.9 µmol, 1.00 eq.) in a mixture of degassed methanol (5.7 mL) and chloroform (1.9 mL) was added K_2CO_3 (10.5 mg, 75.9 µmol, 1.00 eq.). The reaction mixture was stirred at ambient temperature for 1 h. The solution was neutralized by the addition of Amberlite®, filtered and concentrated in vacuo. The residue was taken up in water (4.7 mL) and a solution of bromide **22** (26.6 mg, 0.106 mmol, 1.40 eq.) in THF (9.5 mL) was added. Sodium hydroxide (0.5 M, 152 µL, 75.9 µmol, 1.00 eq.) and a tiny amount of tetrabutylammonium iodide were added. The reaction mixture was stirred for 2 d at ambient temperature. The mixture was neutralized by addition of Amberlite®, filtered and the solvents were removed under reduced pressure. The resulting product was azeotroped with toluene (3× 2.5 mL) and dried in high vacuo for 1 h. Pyridine (1.5 mL), benzoyl chloride (133 µL, 160 mg, 1.14 mmol, 15.0 eq.) and DMAP (83.5 mg, 0.683 mmol, 9.00 eq.) were added and the reaction mixture was stirred at ambient temperature for 1 d. Then again benzoyl chloride (133 µL, 160 mg, 1.14 mmol, 15.0 eq.) was added and the reaction mixture was stirred for further 4 d at ambient temperature. The reaction was stopped by the addition of ethyl acetate (50 mL) and

water (50 mL). The organic phase was washed with brine (50 mL), dried over Na_2SO_4, filtered and concentrated in vacuo. Gel permeation HPLC and subsequent column chromatography (SiO_2, *n*-pentane/EtOAc, 2:1) afforded target compound **39** (69.2 mg, 38.1 mmol, 50 %) as a colorless oil.

$[\alpha]_D^{23}$ = +47.0 (*c* 1.00, $CHCl_3$).

^{1}H-NMR (600 MHz, $CDCl_3$): *δ* (ppm) = −0.11 (s, 9 H), 0.79–0.89 (m, 1 H), 0.85–0.95 (m, 1 H), 0.94–1.04 (m, 1 H), 1.04–1.17 (m, 1 H), 1.17–1.32 (m, 4 H), 2.10–2.24 (m, 2 H), 2.43 (t, *J* = 2.4 Hz, 1 H), 2.53–2.61 (m_c, 2 H), 3.38–3.49 (m, 1 H), 3.51–3.74 (m, 14 H), 3.95 (ddd, *J* = 10.7, 9.6, 5.7 Hz, 1 H), 4.00 (ddd, *J* = 9.8, 5.3, 2.1 Hz, 1 H), 4.06–4.18 (m, 4 H), 4.19 (d, *J* = 2.4 Hz, 2 H), 4.25 (t, *J* = 9.5 Hz, 1 H), 4.45 (dd, *J* = 11.2, 5.7 Hz, 1 H), 4.53–4.60 (m, 2 H), 4.62–4.69 (m_c, 1 H), 4.75 (d, *J* = 7.8 Hz, 1 H), 4.91 (dd, *J* = 11.9, 2.2 Hz, 1 H), 5.06 (dd, *J* = 10.3, 2.9 Hz, 1 H), 5.42 (dd, *J* = 9.7, 7.9 Hz, 1 H), 5.47 (d, *J* = 3.5 Hz, 1 H), 5.65 (dd, *J* = 10.8, 3.5 Hz, 1 H), 5.75–5.86 (m, 2 H), 6.01 (dd, *J* = 3.4, 1.5 Hz, 1 H), 7.17–7.26 (m, 3 H), 7.32–7.46 (m, 18 H), 7.48–7.61 (m, 6 H), 7.74–7.87 (m, 6 H), 7.91–8.14 (m, 12 H).

^{13}C-NMR (126 MHz, $CDCl_3$): *δ* (ppm) = −1.5, 17.9, 25.3, 29.5, 29.7, 31.2, 32.1, 58.4, 61.4, 61.8, 62.8, 67.4, 67.6, 67.9, 69.0, 69.1, 69.9, 70.2, 70.4, 70.6, 70.8, 72.2, 72.4, 73.0, 73.4, 73.7, 74.5, 74.8, 75.3, 76.5, 77.6, 79.6, 97.9, 100.4, 103.0, 128.2, 128.3, 128.4, 128.4, 128.4, 128.6, 128.7, 129.1, 129.2, 129.2, 129.3, 129.4, 129.5, 129.6, 129.6, 129.7, 129.8, 129.8, 133.0, 133.1, 133.1, 133.1, 133.2, 133.4, 133.6, 165.1, 165.2, 165.2, 165.3, 165.5, 165.7, 165.8, 165.8, 166.1.

IR (ATR): $\tilde{\nu}$ (cm^{-1}) = 3066, 2947, 2867, 1722, 1602, 1584, 1451, 1352, 1261, 1091, 1065, 1026, 838, 706.

MS (ESI): *m*/*z* (%) = 929.8 (68) $[M+2Na]^{2+}$, 1835.6 (100) $[M+Na]^+$. **HRMS** (ESI): *m*/*z* calculated for: $C_{100}H_{104}O_{28}SSi$ $[M+Na]^+$: 1835.6096, found: 1835.6096.

2,4,3,6-Tetra-*O*-benzoyl-α-D-galactopyranosyl-(1→4)-3,6-di-*O*-benzoyl-2-*O*-(9,12,15,18,21,24,27,30,33,36,39,42,45-tridecaoxa-6-thiaoctatetracont-47-yne-1-yl)-β-D-galactopyranosyl-(1→4)-2,3,6-tri-*O*-benzoyl-β-D-glucopyranosyl trichloroacetimidate (15)

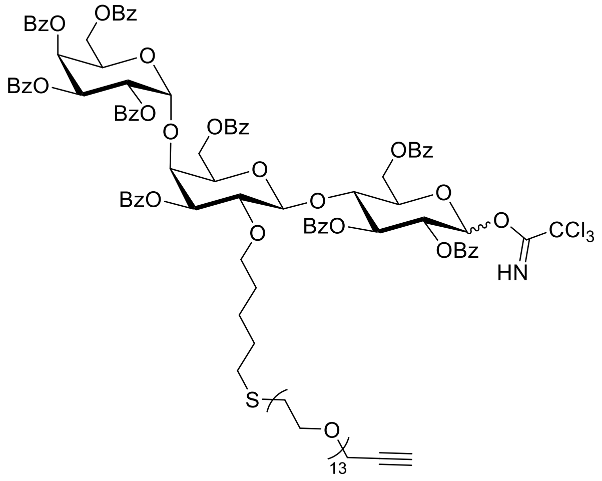

The anomeric hydroxyl group of carbohydrate **38** was deprotected according to general procedure **GP1** using anhydrous dichloromethane (4.4 mL) and TFA (2.2 mL). The reaction was stopped after 2 h. The crude hemiacetal was reacted with trichloroacetonitrile (88.2 µL, 127 mg, 0.878 mmol, 20.0 eq.) and DBU (8.00 µL, 8.12 mg, 52.7 µmol, 1.20 eq.) in anhydrous dichloromethane (4.4 mL) for 2 h. Gel permeation HPLC afforded trichloroacetimidate **15** (74.0 mg, 32.2 µmol, 73 %) as a yellow oil.

^{1}H-NMR (600 MHz, $CDCl_3$): δ (ppm) = 0.76–0.90 (m), 1.12–1.34 (m), 2.05–2.27 (m), 2.44 (t, J = 2.4 Hz), 2.56 (t, J = 7.2 Hz), 3.26–3.46 (m), 3.48–3.75 (m), 4.05 (dd, J = 11.3, 6.7 Hz), 4.10–4.18 (m), 4.20 (d, J = 2.4 Hz), 4.34–4.41 (m), 4.41 (dd, J = 11.3, 6.1 Hz), 4.51 (dd, J = 10.8, 4.1 Hz), 4.55–4.63 (m), 4.86–4.93 (m), 5.06 (dd, J = 10.3, 2.9 Hz), 5.47 (d, J = 3.5 Hz), 5.50 (ddd, J = 10.2, 3.7, 1.0 Hz), 5.63 (dd, J = 10.8, 3.5 Hz), 5.76 (dd, J = 10.9, 3.4 Hz), 5.89–5.96 (m), 6.19–6.28 (m), 6.76 (d, J = 3.7 Hz), 7.18–7.25 (m), 7.33–7.61 (m), 7.69–7.87 (m), 7.92–8.16 (m), 8.59 (s), 8.60 (s).

^{13}C-NMR (151 MHz, $CDCl_3$): δ (ppm) = 25.0, 25.4, 29.0, 29.6, 29.8, 29.9, 31.3, 32.2, 38.4, 58.5, 61.4, 61.4, 62.3, 62.3, 67.9, 68.0, 69.2, 70.0, 70.4, 70.4, 70.5, 70.7, 70.7, 70.8, 70.9, 70.9, 70.9, 71.9, 72.6, 73.7, 73.8, 74.7, 75.1, 75.7, 75.8, 77.6, 79.8, 90.9, 93.2, 97.9, 97.9, 103.4, 103.4, 103.5, 128.4, 128.5, 128.6, 128.6, 128.7, 128.7, 128.8, 128.9, 129.2, 129.3, 129.5, 129.6, 129.7, 129.7, 129.8, 129.9, 130.0, 130.1, 130.1, 133.3, 133.3, 133.3, 133.6, 133.6, 133.7, 133.8, 133.9, 160.6, 165.2, 165.3, 165.3, 165.7, 165.7, 165.9, 166.2.

IR (ATR): $\tilde{\nu}$ (cm^{-1}) = 2919, 2869, 1723, 1451, 1263, 1092, 1067, 1026, 840, 798, 752, 708, 668, 640.

MS (ESI): *m*/*z* (%) = 1170.8 (100) [M+2Na]$^{2+}$, 2318.7 (14) [M+Na]$^+$. **HRMS** (ESI): *m*/*z* calculated for: $C_{117}H_{132}{}^{35}Cl_3NO_{38}S$ [M+Na]$^+$: 2318.7106, found: 2318.7091.

2,4,3,6-Tetra-*O*-benzoyl-α-D-galactopyranosyl-(1→4)-3,6-di-*O*-benzoyl-2-*O*-(9,12,15-trioxa-6-thiaoctadec-17-yne-1-yl)-β-D-galactopyranosyl-(1→4)-2,3,6-tri-*O*-benzoyl-β-D-glucopyranosyl trichloroacetimidate (16)

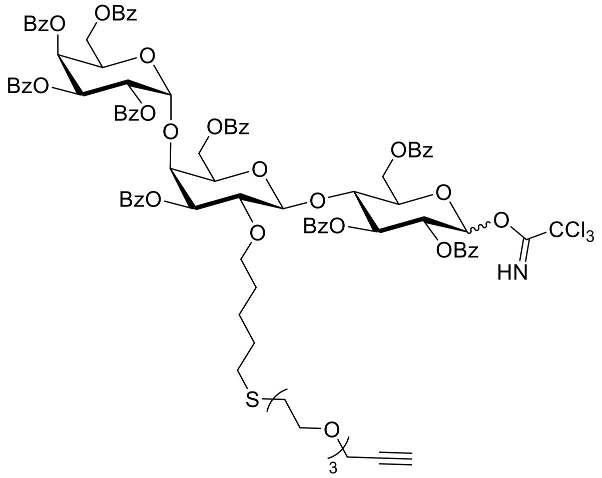

The anomeric hydroxyl group of carbohydrate **39** (63.4 mg, 34.9 µmol, 1.00 eq.) was deprotected according to general procedure **GP1** using anhydrous dichloromethane (3.5 mL) and TFA (1.7 mL). The reaction was stopped after 3 h. The crude hemiacetal was reacted with trichloroacetonitrile (70.1 µL, 101 mg, 0.698 mmol, 20.0 eq.) and DBU (6.25 µL, 6.38 mg, 52.7 µmol, 1.20 eq.) in anhydrous dichloromethane (3.5 mL) for 2 h. Gel permeation HPLC afforded trichloroacetimidate **16** (59.0 mg, 31.8 µmol, 91 %) as a yellow oil.

^{1}H-NMR (600 MHz, $CDCl_3$): δ (ppm) = 0.75–0.92 (m), 0.92–1.00 (m), 1.01–1.13 (m), 1.14–1.38 (m), 2.04–2.23 (m), 2.38–2.46 (m), 2.52–2.61 (m), 3.37–3.43 (m), 3.49–3.73 (m), 4.05 (dd, *J* = 11.3, 6.8 Hz), 4.11–4.19 (m), 4.19 (dd, *J* = 2.4 Hz), 4.35–4.44 (m), 4.51 (ddd, *J* = 10.1, 3.9, 1.7 Hz), 4.56–4.65 (m), 4.87–4.93 (m), 5.06 (dd, *J* = 10.3, 2.8 Hz), 5.47 (d, *J* = 3.6 Hz), 5.50 (dd, *J* = 10.3, 3.7 Hz), 5.60–5.66 (m), 5.77 (dd, *J* = 10.8, 3.4 Hz), 5.91–5.96 (m), 6.20–6.26 (m), 6.76 (d, *J* = 3.7 Hz), 7.12–7.25 (m), 7.33–7.60 (m), 7.72–7.86 (m), 7.91–8.18 (m), 8.53 (s), 8.60 (s).

^{13}C-NMR (151 MHz, $CDCl_3$): δ (ppm) = 25.0, 29.2, 29.5, 31.0, 31.8, 58.2, 61.1, 61.9, 62.0, 67.6, 67.6, 68.9, 69.6, 70.0, 70.2, 70.4, 70.6, 71.5, 72.2, 73.4, 74.3, 74.8, 75.4, 77.0, 79.4, 90.6, 92.9, 97.6, 103.1, 128.1, 128.2, 128.2, 128.2, 128.3, 128.3, 128.4, 128.5, 128.9, 128.9, 129.1, 129.2, 129.3, 129.4, 129.4, 129.6, 129.6, 129.7, 129.7, 132.9, 133.0, 133.0, 133.2, 133.3, 133.3, 133.5, 164.9, 165.0, 165.0, 165.3, 165.3, 165.5, 165.6, 165.8.

IR (ATR): $\tilde{\nu}$ (cm^{-1}) = 2926, 2864, 1722, 1674, 1601, 1451, 1316, 1261, 1091, 1066, 1024, 797, 753, 707.

MS (ESI): *m*/*z* (%) = 950.7 (83) $[M+2Na]^{2+}$, 1878.4 (100) $[M+Na]^+$. **HRMS** (ESI): *m*/*z* calculated for: $C_{97}H_{92}{}^{35}Cl_3NO_{28}S$ $[M+Na]^+$: 1878.4484, found: 1878.4486.

_O_-(2,4,3,6-Tetra-_O_-benzoyl-α-D-galactopyranosyl)-(1→4)-(3,6-di-_O_-benzoyl-2-_O_-(9,12,15,18,21,24,27,30,33,36,39,42,45-tridecaoxa-6-thiaoctatetracont-47-yne-1-yl)-β-D-galactopyranosyl)-(1→4)-2,3,6-tri-_O_-benzoyl-β-D-glucopyranosyl-(1→1)-(2_S_,3_R_,4_E_)-2-azido-3-_O_-pivaloyl-4-octadecene-1,3-diol (23)

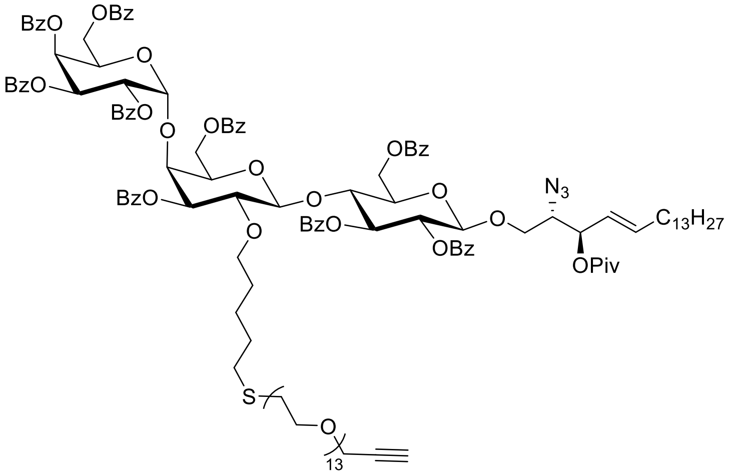

The glycosylation of trichloroacetimidate **15** (36.0 mg, 15.7 µmol, 1.00 eq.) with azidosphingosine **10** (12.8 mg, 31.3 µmol, 2.00 eq.) was performed according to general procedure **GP2** in anhydrous dichloromethane (1.6 mL). TMSOTf (0.1 M in CH_2Cl_2, 31.3 µL, 3.13 µmol, 20.0 mol%) was added and the reaction mixture was stirred for 23 h. Gel permeation HPLC afforded the target compound **23** (19.1 mg, 7.50 µmol, 48 %) as a yellow oil.

$[\alpha]_D^{22}$ = +34.4 (*c* 0.90, $CHCl_3$).

^{1}H-NMR (600 MHz, $CDCl_3$): *δ* (ppm) = 0.78–0.99 (m, 4 H), 0.88 (t, *J* = 7.0 Hz, 3 H), 1.13 (s, 9 H), 1.15–1.33 (m, 28 H), 1.83–1.90 (m, 2 H), 2.10–2.20 (m, 2 H), 2.44 (t, *J* = 2.4 Hz, 1 H), 2.56 (t, *J* = 7.2 Hz, 2 H), 3.39–3.84 (m, 53 H), 4.00 (ddd, *J* = 10.0, 4.9, 2.2 Hz, 1 H), 4.06–4.16 (m, 3 H), 4.16 (d, *J* = 2.9 Hz, 1 H), 4.20 (d, *J* = 2.4 Hz, 2 H), 4.25–4.31 (m_c, 1 H), 4.43 (dd, *J* = 11.2, 5.6 Hz, 1 H), 4.54–4.60 (m, 2 H), 4.62–4.67 (m, 1 H), 4.75 (d, *J* = 7.7 Hz, 1 H), 4.88–4.92 (m, 1 H), 5.05 (dd, *J* = 10.3, 2.9 Hz, 1 H), 5.23 (dd, *J* = 7.9, 4.2 Hz, 1 H), 5.26–5.33 (m, 1 H), 5.43 (dd, *J* = 9.5, 7.7 Hz, 1 H), 5.47 (d, *J* = 3.5 Hz, 1 H), 5.59 (dt, *J* = 14.3, 6.9 Hz, 1 H), 5.64 (dd, *J* = 10.9, 3.4 Hz, 1 H), 5.79 (t, *J* = 9.3 Hz, 1 H), 5.83 (dd, *J* = 10.9, 3.4 Hz, 1 H), 6.01 (d, *J* = 2.1 Hz, 1 H), 7.18–7.25 (m, 3 H), 7.31–7.60 (m, 24 H), 7.73–7.84 (m, 6 H), 7.93–8.13 (m, 12 H).

^{13}C-NMR (151 MHz, $CDCl_3$): *δ* (ppm) = 13.9, 22.5, 25.1, 26.8, 28.4, 28.8, 29.1, 29.2, 29.3, 29.4, 29.4, 29.4, 29.5, 31.0, 31.7, 31.8, 32.0, 38.6, 58.2, 60.2, 61.1, 61.5, 62.4, 63.1, 67.4, 67.7, 67.8, 68.8, 68.9, 69.7, 70.0, 70.2, 70.3, 70.4, 70.5, 71.7, 72.2, 72.5, 73.3, 73.4, 73.5, 73.6, 74.4, 74.6, 75.0, 75.9, 77.3, 79.4, 97.7, 100.4, 102.8, 122.5, 128.0, 128.1, 128.1, 128.1, 128.2, 128.3, 128.4, 128.5, 128.9, 128.9, 129.0, 129.1, 129.1, 129.2, 129.2, 129.3, 129.4, 129.4, 129.5, 129.5, 129.6, 132.9, 132.9, 133.0, 133.1, 133.2, 133.4, 138.0, 164.8, 165.0, 165.0, 165.3, 165.5, 165.6, 165.6, 165.9, 176.4.

IR (ATR): $\tilde{\nu}$ (cm^{-1}) = 2924, 2858, 2102, 1724, 1602, 1452, 1315, 1263, 1092, 1067, 1027, 847, 754, 708.

MS (ESI): *m*/*z* (%) = 1295.1 (100) $[M+2Na]^{2+}$, 2567.1 (29) $[M+Na]^{+}$. **HRMS** (ESI): *m*/*z* calculated for: $C_{138}H_{173}N_3O_{40}S$ $[M+Na]^{+}$: 2567.1208, found: 2567.1189.

_O_-(2,4,3,6-Tetra-_O_-benzoyl-α-D-galactopyranosyl)-(1→4)-(3,6-di-_O_-benzoyl-2-_O_-(9,12,15-trioxa-6-thiaoctadec-17-yne-1-yl)-β-D-galactopyranosyl)-(1→4)-2,3,6-tri-_O_-benzoyl-β-D-glucopyranosyl-(1→1)-(2_S_,3_R_,4_E_)-2-azido-3-_O_-pivaloyl-4-octadecene-1,3-diol (24)

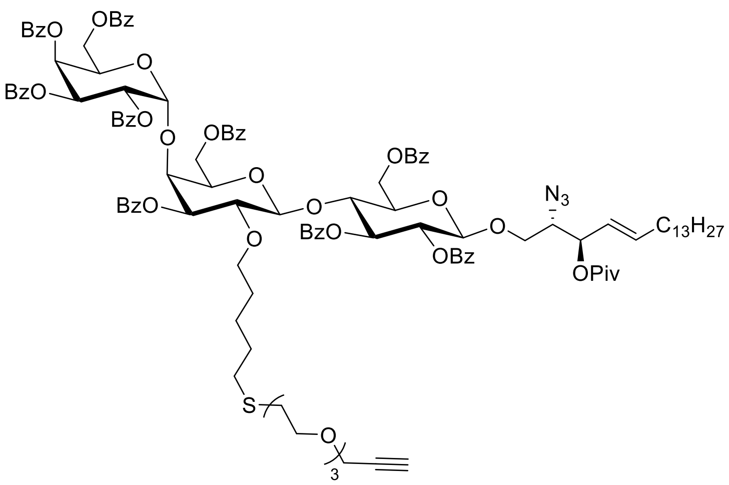

The glycosylation of trichloroacetimidate **16** (122 mg, 65.7 µmol, 1.00 eq.) with azido-sphingosine **10** (53.7 mg, 0.131 mmol, 2.00 eq.) was performed according to general procedure **GP2** in anhydrous dichloromethane (6.6 mL) with TMSOTf (2.39 µL, 2.91 mg, 13.1 µmol, 20.0 mol%) in 15 h. Column chromatography on silica gel (*n*-pentane/EtOAc, 3:1 → 2:1 → 1:1) afforded target compound **24** (52.9 mg, 25.1 µmol, 38 %) as a colorless oil.

$[\alpha]_D^{22}$ = +30.0 (*c* 1.57, $CHCl_3$).

^{1}H-NMR (600 MHz, $CDCl_3$): *δ* (ppm) = 0.79–0.93 (m, 2 H), 0.88 (t, *J* = 7.0 Hz, 3 H), 0.92–1.04 (m, 2 H), 1.13 (s, 9 H), 1.15–1.30 (m, 25 H), 1.83–1.90 (m, 2 H), 2.11–2.22 (m, 2 H), 2.41–2.45 (m, 1 H), 2.57 (t, *J* = 7.1 Hz, 2 H), 3.40–3.47 (m, 1 H), 3.51–3.77 (m, 14 H), 3.78–3.87 (m, 1 H), 3.96–4.05 (m, 1 H), 4.05–4.18 (m, 4 H), 4.19 (d, *J* = 2.2 Hz, 2 H), 4.25–4.32 (m_c, 1 H), 4.43 (dd, *J* = 11.2, 5.6 Hz, 1 H), 4.53–4.60 (m, 2 H), 4.62–4.68 (m_c, 1 H), 4.75 (d, *J* = 8.0 Hz, 1 H), 4.90 (dd, *J* = 11.9, 2.1 Hz, 1 H), 5.05 (dd, *J* = 10.4, 2.9 Hz, 1 H), 5.22 (dd, *J* = 8.0, 4.0 Hz, 1 H), 5.26–5.33 (m, 1 H), 5.43 (dd, *J* = 9.6, 7.5 Hz, 1 H), 5.47 (d, *J* = 3.6 Hz, 1 H), 5.55–5.63 (m, 1 H), 5.64 (dd, *J* = 11.0, 3.5 Hz, 1 H), 5.79 (t, *J* = 9.3 Hz, 1 H), 5.83 (dd, *J* = 10.9 , 3.3 Hz, 1 H), 6.00–6.03 (m, 1 H), 7.15–7.25 (m, 5 H), 7.32–7.47 (m, 16 H), 7.48–7.61 (m, 6 H), 7.73–7.87 (m, 6 H), 7.93–8.13 (m, 12 H).

^{13}C-NMR (151 MHz, $CDCl_3$): *δ* (ppm) = 13.9, 22.5, 25.1, 26.8, 28.4, 28.8, 29.1, 29.2, 29.3, 29.4, 29.4, 29.5, 29.5, 31.0, 31.7, 31.8, 32.0, 38.6, 58.2, 61.1, 61.6, 62.4, 63.1, 67.4, 67.7, 67.8, 68.8,

68.9, 69.7, 70.0, 70.2, 70.4, 70.6, 71.7, 72.2, 72.6, 73.3, 73.5, 73.6, 74.3, 74.6, 75.1, 75.9, 77.3, 79.4, 97.7, 100.4, 102.8, 122.5, 128.0, 128.1, 128.1, 128.2, 128.2, 128.3, 128.4, 128.5, 128.9, 129.0, 129.0, 129.1, 129.2, 129.2, 129.3, 129.3, 129.4, 129.4, 129.5, 129.6, 132.9, 132.9, 133.0, 133.1, 133.2, 133.5, 138.0, 164.8, 165.0, 165.0, 165.3, 165.5, 165.6, 165.6, 165.9, 176.5.

IR (ATR): $\tilde{\nu}$ (cm^{-1}) = 2925, 2855, 2102, 1723, 1602, 1452, 1316, 1263, 1091, 1066, 1027, 972, 851, 829, 707.

MS (ESI): *m*/*z* (%) = 1074.9 (58) [M+2Na]$^{2+}$, 2126.9 (100) [M+Na]$^{+}$. **HRMS** (ESI): *m*/*z* calculated for: $C_{118}H_{133}N_3O_{30}S$ [M+Na]$^{+}$:2126.8587, found:2126.8592.

O-(2,4,3,6-Tetra-O-benzoyl-α-D-galactopyranosyl)-(1→4)-(3,6-di-O-benzoyl-2-O-(9,12,15,18,21,24,27,30,33,36,39,42,45-tridecaoxa-6-thiaoctatetracont-47-yne-1-yl)-β-D-galactopyranosyl)-(1→4)-2,3,6-tri-O-benzoyl-β-D-glucopyranosyl-(1→1)-(2S,3R,4E)-3-O-pivaloyl-2-(tetracosanamido)-4-octadecene-1,3-diol (40)

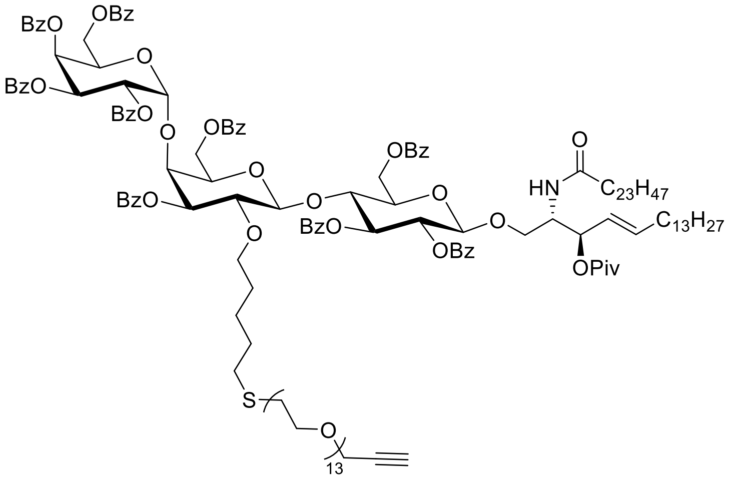

The reduction of azide **23** (19.1 mg, 7.50 µmol, 1.00 eq.) was performed according to general procedure **GP3** with triphenylphosphine (4.93 mg, 18.8 µmol, 2.50 eq.) in benzene (1.5 mL) and water (6.0 µL). The resulting amine was dissolved in THF (0.5 mL) and was reacted with lignoceric acid (**11**) (3.59 mg, 9.75 µmol, 1.30 eq.), DIPEA (1.70 µL, 1.26 mg, 9.75 µmol, 1.30 eq.) and a solution of HATU (3.71 mg, 9.75 µmol, 1.30 eq.) in DMF (0.25 mL). The reaction mixture was stirred for 2 h. Gel permeation HPLC afforded glycolipid **40** (13.5 mg, 4.70 µmol, 63 %) as a colorless oil.

$[\alpha]_D^{26}$ = +38.8 (*c* 0.60, $CHCl_3$).

^{1}H-NMR (600 MHz, $CDCl_3$): *δ* (ppm) = 0.75–0.92 (m, 10 H), 1.06 (s, 9 H), 1.13–1.44 (m, 55 H), 1.59–1.71 (m, 2 H), 1.88–1.98 (m, 2 H), 2.05–2.21 (m, 2 H), 2.44 (t, *J* = 2.4 Hz, 1 H), 2.56 (t, *J* = 7.1 Hz, 2 H), 3.38–3.45 (m, 1 H), 3.47 (dd, *J* = 9.7, 3.7 Hz, 1 H), 3.50–3.57 (m, 4 H), 3.57–3.74 (m, 61 H), 3.76 (t, *J* = 5.9 Hz, 1 H), 3.97 (ddd, *J* = 9.6, 4.6, 2.0 Hz, 1 H), 4.06 (dd, *J* = 8.5, 6.1 Hz, 1 H), 4.08–4.19 (m, 3 H), 4.21 (d, *J* = 2.2 Hz, 2 H), 4.25 (t, *J* = 9.5 Hz, 1 H), 4.47 (dd,

J = 11.2, 5.7 Hz, 1 H), 4.52–4.58 (m, 2 H), 4.58–4.64 (m, 1 H), 4.67 (d, *J* = 7.9 Hz, 1 H), 4.85–4.88 (m, 1 H), 5.05 (dd, *J* = 10.3, 2.9 Hz, 1 H), 5.16 (t, *J* = 7.4 Hz, 1 H), 5.26–5.33 (m, 1 H), 5.37 (dd, *J* = 9.7, 7.8 Hz, 1 H), 5.46–5.51 (m, 2 H), 5.62 (dd, *J* = 10.8, 3.6 Hz, 1 H), 5.65–5.73 (m, 1 H), 5.77–5.83 (m, 2 H), 5.98 (dd, *J* = 3.4, 1.5 Hz, 1 H), 7.11–7.26 (m, 6 H), 7.31–7.62 (m, 21 H), 7.72–7.87 (m, 6 H), 7.90–8.15 (m, 12 H).

^{13}C-NMR (151 MHz, $CDCl_3$): δ (ppm) = 14.1, 15.1, 22.7, 25.3, 25.5, 26.9, 29.0, 29.2, 29.3, 29.4, 29.4, 29.5, 29.5, 29.6, 29.6, 29.7, 29.7, 29.7, 29.7, 29.7, 31.2, 31.9, 32.0, 32.2, 36.4, 38.6, 42.8, 50.2, 58.4, 61.4, 61.8, 62.6, 66.6, 67.6, 67.8, 69.0, 69.1, 69.7, 70.0, 70.2, 70.2, 70.3, 70.3, 70.5, 70.7, 71.3, 72.4, 72.5, 73.0, 73.5, 73.7, 74.6, 74.8, 75.3, 76.0, 77.6, 79.6, 97.8, 101.1, 103.0, 125.0, 128.3, 128.4, 128.4, 128.5, 128.5, 128.6, 128.7, 129.1, 129.1, 129.2, 129.2, 129.3, 129.4, 129.5, 129.5, 129.6, 129.6, 129.7, 129.8, 129.9, 129.9, 133.1, 133.2, 133.4, 133.7, 136.8, 165.1, 165.2, 165.2, 165.6, 165.7, 165.8, 165.8, 166.1, 172.5, 176.8.

IR (ATR): $\tilde{\nu}$ (cm^{-1}) = 2923, 2854, 1725, 1679, 1602, 1453, 1263, 1095, 1068, 1029, 845, 804, 711.

MS (ESI): *m*/*z* (%) = 1457.2 (13) $[M+2Na]^{2+}$, 2891.5 (100) $[M+Na]^{+}$. **HRMS** (ESI): *m*/*z* calculated for: $C_{162}H_{221}NO_{41}S$ $[M+Na]^{+}$: 2891.4852, found: 2891.4839.

O-(2,4,3,6-Tetra-*O*-benzoyl-α-D-galactopyranosyl)-(1→4)-(3,6-di-*O*-benzoyl-2-*O*-(9,12,15-trioxa-6-thiaoctadec-17-yne-1-yl)-β-D-galactopyranosyl)-(1→4)-2,3,6-tri-*O*-benzoyl-β-D-glucopyranosyl-(1→1)-(2*S*,3*R*,4*E*)-3-*O*-pivaloyl-2-(tetracosanamido)-4-octadecene-1,3-diol (41)

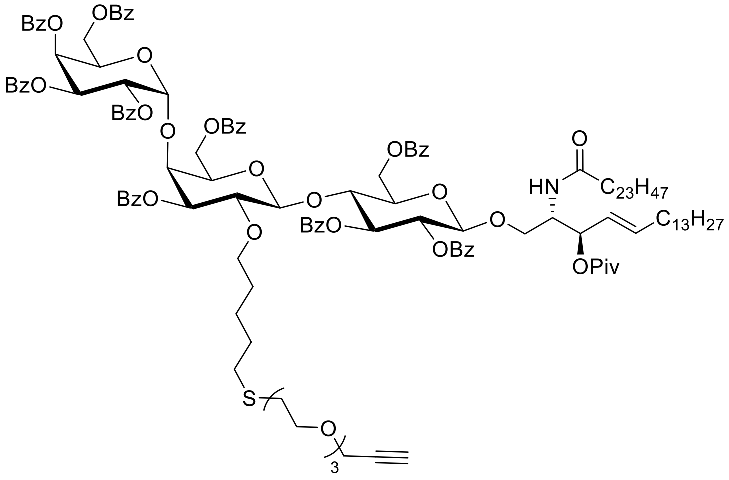

The reduction of azide **24** (30.0 mg, 14.2 µmol, 1.00 eq.) was performed according to general procedure **GP3** with triphenylphosphine (9.34 mg, 35.6 µmol, 2.50 eq.) in benzene (2.8 mL) and water (3 drops). The resulting amine was dissolved in THF (1.4 mL) and was reacted with lignoceric acid (**11**) (6.82 mg, 18.5 µmol, 1.30 eq.), DIPEA (3.22 µL, 2.39 mg, 18.5 µmol, 1.30 eq.) and a solution of HATU (7.03 mg, 18.5 µmol, 1.30 eq.) in DMF (0.70 mL). The reaction

mixture was stirred for 20 h. Gel permeation HPLC afforded glycolipid **41** (13.6 mg, 5.60 μmol, 39 %) as a colorless oil.

$[\alpha]_D^{20}$ = +34.8 (*c* 1.36, $CHCl_3$).

^{1}H-NMR (600 MHz, $CDCl_3$): *δ* (ppm) = 0.74–0.95 (m, 8 H), 0.93–1.01 (m, 2 H), 1.06 (s, 9 H), 1.11–1.38 (m, 65 H), 1.61–1.70 (m, 2 H), 1.90–1.97 (m, 2 H), 2.10–2.21 (m, 2 H), 2.43 (t, *J* = 2.3 Hz, 1 H), 2.57 (t, *J* = 7.1 Hz, 2 H), 3.34–3.46 (m, 1 H), 3.50–3.73 (m, 14 H), 3.94–4.00 (m, 1 H), 4.02–4.18 (m, 6 H), 4.19 (d, *J* = 2.3 Hz, 2 H), 4.22–4.30 (m, 2 H), 4.43–4.49 (m_c, 1 H), 4.51–4.59 (m, 2 H), 4.59–4.64 (m, 1 H), 4.67 (d, *J* = 7.8 Hz, 1 H), 4.87 (dd, *J* = 12.1, 2.1 Hz, 1 H), 5.05 (dd, *J* = 10.4, 2.8 Hz, 1 H), 5.14–5.20 (m_c, 1 H), 5.29 (ddd, *J* = 15.4 Hz, 7.3, 1.3 Hz, 1 H), 5.37 (dd, *J* = 9.8, 7.8 Hz, 1 H), 5.45–5.51 (m, 2 H), 5.62 (dd, *J* = 10.8, 3.4 Hz, 1 H), 5.65–5.74 (m, 1 H), 5.75–5.84 (m, 2 H), 5.98–6.00 (m, 1 H), 7.16–7.26 (m, 4 H), 7.32–7.47 (m, 17 H), 7.48–7.62 (m, 6 H), 7.73–7.83 (m, 6 H), 7.92–8.13 (m, 12 H).

^{13}C-NMR (151 MHz, $CDCl_3$): *δ* (ppm) = 14.1, 22.7, 25.3, 25.5, 26.9, 29.0, 29.2, 29.4, 29.4, 29.5, 29.5, 29.6, 29.7, 29.7, 29.7, 29.7, 31.2, 31.9, 32.1, 32.2, 36.4, 38.7, 50.2, 58.4, 61.4, 61.8, 62.6, 67.6, 67.9, 69.1, 69.1, 70.0, 70.2, 70.4, 70.6, 70.8, 72.4, 72.5, 72.5, 73.0, 73.5, 73.7, 74.6, 74.8, 75.3, 76.0, 77.6, 79.6, 97.9, 101.1, 103.0, 125.0, 128.3, 128.4, 128.4, 128.5, 128.5, 128.6, 128.7, 129.1, 129.2, 129.3, 129.4, 129.5, 129.5, 129.6, 129.6, 129.8, 129.8, 129.9, 129.9, 133.1, 133.1, 133.2, 133.3, 133.4, 133.5, 133.7, 136.8, 165.1, 165.2, 165.2, 165.6, 165.7, 165.8, 165.8, 166.1, 172.4, 176.8.

IR (ATR): $\tilde{\nu}$ (cm^{-1}) = 2923, 2853, 1723, 1681, 1601, 1451, 1264, 1091, 1066, 1026, 844, 801, 754, 707.

MS (ESI): *m*/*z* (%) = 1237.1 (100) $[M+2Na]^{2+}$, 2451.2 (55) $[M+Na]^+$. **HRMS** (ESI): *m*/*z* calculated for: $C_{142}H_{181}NO_{31}S$ $[M+Na]^+$: 2451.2242, found: 2451.2206.

O-(2,4,3,6-Tetra-O-benzoyl-α-D-galactopyranosyl-(1→4)-(3,6-di-O-benzoyl-2-O-(9,12,15,18,21,24,27,30,33,36,39,42,45-tridecaoxa-6-thiaoctatetracont-47-yne-1-yl)-β-D-galactopyranosyl)-(1→4)-2,3,6-tri-O-benzoyl-β-D-glucopyranosyl-(1→1)-(2S,3R,4E,2′R)-2-(2′-benzoyloxytetracosanamido)-3-O-pivaloyl-4-octadecene-1,3-diol (42)

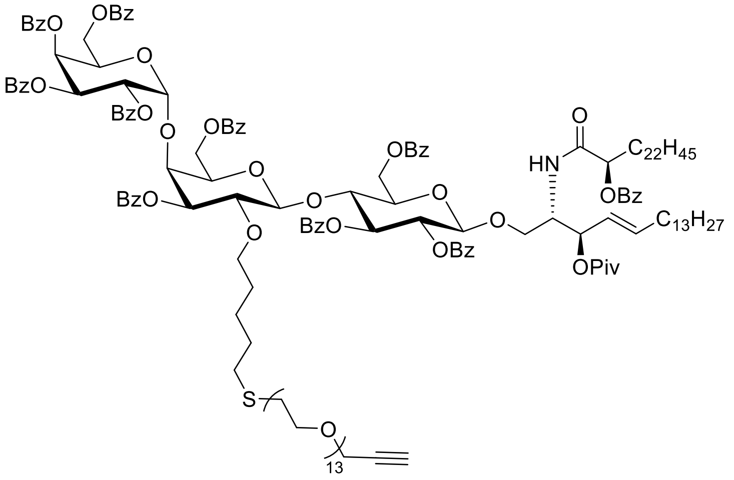

The reduction of azide **23** (23.0 mg, 9.03 µmol, 1.00 eq.) was performed according to general procedure **GP3** using triphenylphosphine (5.93 mg, 22.6 µmol, 2.50 eq.) in benzene (1.8 mL) and water (3 drops). The amine was dissolved in THF (0.90 mL) and hydroxylated acid **12**[4] (5.72 mg, 11.7 µmol, 1.30 eq.), DIPEA (2.04 µL, 1.51 mg, 11.7 µmol, 1.30 eq.) and a solution of HATU (4.45 mg, 11.7 µmol, 1.30 eq.) in DMF (0.45 mL) were added. The reaction mixture was stirred for 16 h. Column chromatography on silica gel (CH_2Cl_2/MeOH, 40:1 → 15:1), followed by gel permeation HPLC afforded glycolipid **42** (10.3 mg, 3.44 µmol, 38 %) as a colorless oil.

$[\alpha]_D^{20}$ = +38.9 (*c* 0.60, $CHCl_3$).

^{1}H-NMR (500 MHz, $CDCl_3$): *δ* (ppm) = 0.77–0.94 (m, 10 H), 0.89–0.99 (m, 2 H), 1.04 (s, 9 H), 1.10–1.40 (m, 71 H), 1.69–1.88 (m, 3 H), 2.07–2.20 (m, 2 H), 2.44 (t, *J* = 2.4 Hz, 1 H), 2.52–2.59 (m_c, 2 H), 3.35–3.74 (m, 47 H), 3.86–3.93 (m, 1 H), 3.94 (dd, *J* = 10.2, 4.3 Hz, 1 H), 4.02–4.19 (m, 5 H), 4.20 (d, *J* = 2.4 Hz, 2 H), 4.27–4.35 (m, 1 H), 4.44 (dd, *J* = 11.1, 5.6 Hz, 1 H), 4.50 (dd, *J* = 12.1, 4.7 Hz, 1 H), 4.54 (d, *J* = 7.5 Hz, 1 H), 4.59–4.66 (m, 1 H), 4.68 (d, *J* = 7.7 Hz, 1 H), 4.78–4.84 (m, 1 H), 5.04 (dd, *J* = 10.2, 2.9 Hz, 1 H), 5.12–5.19 (m, 1 H), 5.23–5.35 (m, 3 H), 5.47 (d, *J* = 3.6 Hz, 1 H), 5.58 (dd, *J* = 14.2, 6.9 Hz 1 H), 5.63 (dd, *J* = 10.9, 3.4 Hz, 1 H), 5.69–5.76 (m_c, 1 H), 5.81 (dd, *J* = 10.8, 3.4 Hz, 1 H), 5.96–6.02 (m, 1 H), 6.37 (d, *J* = 8.8 Hz, 1 H), 7.17–7.25 (m, 4 H), 7.28–7.62 (m, 26 H), 7.71–7.90 (m, 8 H), 7.93–8.14 (m, 12 H).

^{13}C-NMR (151 MHz, $CDCl_3$): *δ* (ppm) = 14.1, 22.7, 24.7, 25.3, 26.9, 26.9, 28.8, 29.2, 29.4, 29.4, 29.4, 29.5, 29.5, 29.6, 29.7, 29.7, 29.7, 31.2, 31.5, 31.9, 32.0, 32.2, 38.7, 50.8, 58.4, 61.2, 61.8, 62.6, 67.1, 67.6, 67.9, 69.0, 69.1, 69.9, 70.2, 70.4, 70.5, 70.8, 72.0, 72.4, 72.8, 73.0, 73.5, 73.7, 74.5, 74.6, 74.8, 75.2, 76.1, 77.5, 79.7, 97.9, 100.6, 103.1, 124.3, 128.3, 128.3, 128.4, 128.4, 128.5, 128.6, 128.6, 128.7, 129.1, 129.2, 129.2, 129.2, 129.3, 129.4, 129.4, 129.5, 129.6, 129.6, 129.7, 129.7, 129.8, 129.9, 133.1, 133.1, 133.3, 133.4, 133.5, 133.5, 133.7, 136.6, 165.0, 165.1, 165.2, 165.5, 165.7, 165.8, 166.1, 169.3, 176.9.

IR (ATR): $\tilde{\nu}$ (cm^{-1}) = 2923, 2855, 1725, 1602, 1516, 1453, 1352, 1315, 1263, 1095, 1068, 1028, 846, 803, 710.

MS (ESI): *m*/*z* (%) = 1019.2 (33) $[M+3Na]^{3+}$, 1517.2 (100) $[M+2Na]^{2+}$, 3011.5 (8) $[M+Na]^{+}$. **HRMS** (ESI): *m*/*z* calculated for: $C_{169}H_{225}NO_{43}S$ $[M+Na]^{+}$: 3011.5063, found: 3011.5004.

***O*-(2,4,3,6-Tetra-*O*-benzoyl-α-D-galactopyranosyl)-(1→4)-(3,6-di-*O*-benzoyl-2-*O*-(9,12,15-trioxa-6-thiaoctadec-17-yne-1-yl)-β-D-galactopyranosyl)-(1→4)-2,3,6-tri-*O*-benzoyl-β-D-glucopyranosyl-(1→1)-(2*S*,3*R*,4*E*,2′*R*)-2-(2′-benzoyloxytetracosanamido)-3-*O*-pivaloyl-4-octadecene-1,3-diol (43)**

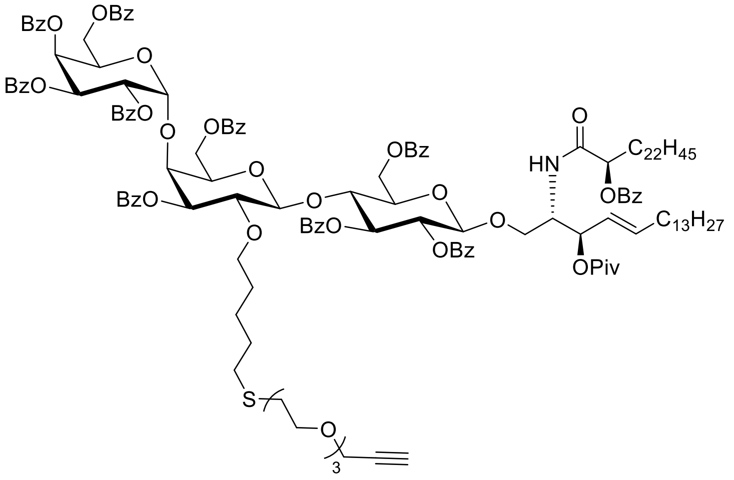

The reduction of azide **24** (20.0 mg, 9.50 µmol, 1.00 eq.) was performed according to general procedure **GP3** with triphenylphosphine (6.24 mg, 23.8 µmol, 2.50 eq.) in benzene (1.9 mL) and water (3 drops). The resulting amine was dissolved in THF (0.95 mL) and was reacted with fatty acid **12**[4] (6.06 mg, 12.4 µmol, 1.30 eq.), DIPEA (2.16 µL, 1.60 mg, 12.4 µmol, 1.30 eq.) and a solution of HATU (4.71 mg, 12.4 µmol, 1.30 eq.) in DMF (0.50 mL). The reaction mixture was stirred for 16 h at ambient temperature. Column chromatography on silica gel (*n*-pentane/EtOAc, 3:1 → 2:1) afforded glycolipid **43** (17.0 mg, 6.67 µmol, 70 %) as a colorless oil.

$[\boldsymbol{\alpha}]_{\mathbf{D}}^{\mathbf{23}}$ = +31.5 (*c* 1.67, $CHCl_3$).

^{1}H-NMR (600 MHz, $CDCl_3$): *δ* (ppm) = 0.72–1.00 (m, 9 H), 1.05 (s, 9 H), 1.09–1.32 (m, 64 H), 1.74–1.87 (m, 4 H), 2.06–2.21 (m, 2 H), 2.41–2.45 (m_c, 1 H), 2.56 (t, *J* = 7.1 Hz, 2 H), 3.38–3.47 (m, 1 H), 3.49–3.74 (m, 14 H), 3.90 (ddd, *J* = 9.9, 4.7, 2.1 Hz, 1 H), 3.94 (dd, *J* = 10.4, 4.4 Hz, 1 H), 4.00–4.19 (m, 5 H), 4.19 (d, *J* = 2.4 Hz, 2 H), 4.26–4.36 (m, 2 H), 4.43 (dd, *J* = 11.1, 5.6 Hz, 1 H), 4.50 (dd, *J* = 12.0, 4.7 Hz, 1 H), 4.54 (d, *J* = 7.5 Hz, 1 H), 4.61–4.66 (m_c, 1 H), 4.69 (d, *J* = 7.7 Hz, 1 H), 4.77–4.84 (m, 1 H), 5.05 (dd, *J* = 10.3, 2.8 Hz, 1 H), 5.16 (t, *J* = 6.0 Hz, 1 H), 5.24–5.34 (m, 3 H), 5.47 (d, *J* = 3.6 Hz, 1 H), 5.53–5.62 (m, 1 H), 5.63 (dd, *J* = 10.8, 3.5 Hz, 1 H), 5.73 (t, *J* = 9.4 Hz, 1 H), 5.82 (dd, *J* = 10.9, 3.4 Hz, 1 H), 5.99–6.01 (m, 1 H), 6.37 (d, *J* = 8.7 Hz, 1 H), 7.15–7.25 (m, 4 H), 7.29–7.63 (m, 26 H), 7.72–7.82 (m, 6 H), 7.84–8.15 (m, 14 H).

^{13}C-NMR (151 MHz, $CDCl_3$): *δ* (ppm) = 14.1, 22.7, 24.7, 25.3, 26.9, 28.8, 29.2, 29.4, 29.4, 29.4, 29.5, 29.5, 29.6, 29.7, 29.7, 29.7, 29.7, 31.2, 31.5, 31.9, 32.0, 32.2, 38.7, 50.8, 58.4, 61.2, 61.8, 62.6, 67.1, 67.6, 67.9, 69.0, 69.1, 69.9, 70.2, 70.4, 70.6, 70.8, 72.0, 72.4, 72.8, 73.0, 73.5, 73.7, 74.6, 74.6, 74.8, 75.2, 76.1, 77.5, 79.6, 97.9, 100.6, 103.1, 124.3, 128.3, 128.3, 128.4, 128.4, 128.5, 128.6, 128.6, 128.7, 129.1, 129.2, 129.2, 129.2, 129.3, 129.4, 129.4, 129.5, 129.6, 129.7, 129.7, 129.8, 129.9, 133.1, 133.1, 133.1, 133.2, 133.4, 133.5, 133.5, 133.7, 136.6, 165.0, 165.1, 165.2, 165.5, 165.7, 165.8, 166.1, 169.3, 176.9.

IR (ATR): $\tilde{\nu}$ (cm^{-1}) = 2924, 2853, 1724, 1602, 1452, 1316, 1264, 1093, 1067, 1027, 754, 707.

MS (ESI): *m*/*z* (%) = 1297.1 (95) [M+2Na]$^{2+}$, 2571.2 (100) [M+Na]$^{+}$. **HRMS** (ESI): *m*/*z* calculated for: $C_{149}H_{185}NO_{33}S$ [M+Na]$^{+}$: 2571.2442, found: 2571.2446.

***O*-(2,4,3,6-Tetra-*O*-benzoyl-α-D-galactopyranosyl)-(1→4)-(3,6-di-*O*-benzoyl-2-*O*-(9,12,15,18,21,24,27,30,33,36,39,42,45-tridecaoxa-6-thiaoctatetracont-47-yne-1-yl)-β-D-galactopyranosyl)-(1→4)-2,3,6-tri-*O*-benzoyl-β-D-glucopyranosyl-(1→1)-(2*S*,3*R*,4*E*,15′*Z*)-3-*O*-pivaloyl-2-(15′-tetracosenamido)-4-octadecen-1,3-diol (44)**

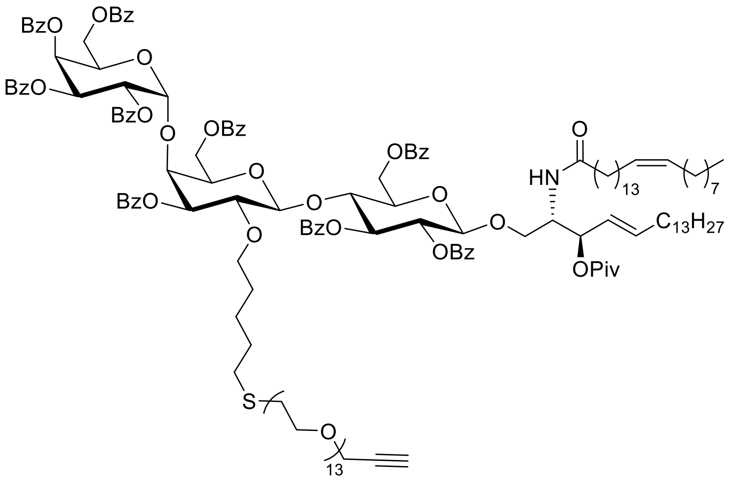

The reduction of azide **23** (25.6 mg, 10.1 µmol, 1.00 eq.) was performed according to general procedure **GP3** with triphenylphosphine (6.58 mg, 25.1 µmol, 2.50 eq.) in benzene (2.0 mL) and water (7.0 µL). The amine was dissolved in THF (0.50 mL) and nervonic acid (**13**) (4.80 mg, 13.1 µmol, 1.30 eq.), DIPEA (2.28 µL, 1.69 mg, 13.1 µmol, 1.30 eq.) and a solution of HATU (4.98 mg, 13.1 µmol, 1.30 eq.) in DMF (0.30 mL) were added and the reaction mixture was stirred for 3 h. Gel permeation HPLC afforded glycolipid **44** (11.5 mg, 4.01 µmol, 40 %) as a colorless oil.

$[\alpha]_{\mathrm{D}}^{27}$ = +25.0 (*c* 1.00, $CHCl_3$).

^{1}H-NMR (600 MHz, $CDCl_3$): *δ* (ppm) = 0.77–0.92 (m, 9 H), 1.06 (s, 9 H), 1.13–1.34 (m, 63 H), 1.89–1.98 (m, 2 H), 1.97–2.05 (m, 4 H), 2.08–2.20 (m, 2 H), 2.42–2.47 (m, 1 H), 2.51–2.62 (m, 2 H), 3.39–3.85 (m, 55 H), 4.02–4.19 (m, 5 H), 4.19–4.22 (m, 2 H), 4.23–4.32 (m, 2 H), 4.47 (dd,

J = 11.1, 5.6 Hz, 1 H), 4.51–4.59 (m, 2 H), 4.61 (t, *J* = 6.6 Hz, 1 H), 4.64–4.71 (m, 1 H), 4.83–4.90 (m_c, 1 H), 5.05 (dd, *J* = 10.2, 2.8 Hz 1 H), 5.13–5.20 (m_c, 1 H), 5.29 (dd, *J* = 15.5, 7.4 Hz, 1 H), 5.32–5.41 (m, 3 H), 5.43–5.52 (m, 2 H), 5.62 (dd, *J* = 10.8, 3.5 Hz, 1 H), 5.65–5.74 (m_c, 1 H), 5.80 (dd, *J* = 10.8, 3.3 Hz, 1 H), 5.98 (dd, *J* = 3.5, 1.4 Hz, 1 H), 7.12–7.26 (m, 3 H), 7.32–7.61 (m, 24 H), 7.73–7.86 (m, 6 H), 7.93–8.14 (m, 12 H).

^{13}C-NMR (151 MHz, $CDCl_3$): δ (ppm) = 14.1, 22.7, 25.3, 25.5, 26.9, 27.2, 27.2, 29.0, 29.2, 29.3, 29.4, 29.4, 29.5, 29.6, 29.6, 29.7, 29.7, 29.8, 29.8, 31.2, 31.9, 31.9, 32.1, 32.2, 36.4, 38.6, 50.2, 58.5, 61.4, 61.8, 62.6, 67.6, 67.9, 69.1, 70.0, 70.2, 70.3, 70.4, 70.4, 70.5, 70.8, 72.4, 72.5, 73.0, 73.7, 74.8, 75.3, 76.0, 77.3, 79.6, 97.8, 101.1, 103.0, 125.0, 128.2, 128.3, 128.4, 128.4, 128.5, 128.5, 128.6, 128.7, 129.1, 129.2 ,129.2, 129.3, 129.4, 129.5, 129.5, 129.6, 129.6, 129.8, 129.8, 129.9, 133.1, 133.2, 133.3, 133.4, 133.5, 133.7, 136.8, 165.1, 165.2, 165.2, 165.6, 165.7, 165.8, 165.8, 166.1, 172.4, 176.8.

IR (ATR): $\tilde{\nu}$ (cm^{-1}) = 2923, 2855, 1725, 1677, 1452, 1265, 1093, 1067, 1028, 843, 754, 709.

MS (ESI): *m*/*z* (%) = 1456.2 (100) $[M+2Na]^{2+}$, 2889.5 (31) $[M+Na]^+$. **HRMS** (ESI): *m*/*z* calculated for: $C_{162}H_{219}NO_{41}S$ $[M+Na]^+$: 2889.4695, found: 2889.4629.

O-(2,4,3,6-Tetra-O-benzoyl-α-D-galactopyranosyl)-(1→4)-(3,6-di-O-benzoyl-2-O-(9,12,15-trioxa-6-thiaoctadec-17-yne-1-yl)-β-D-galactopyranosyl)-(1→4)-2,3,6-tri-O-benzoyl-β-D-glucopyranosyl-(1→1)-(2S,3R,4E,15′Z)-3-O-pivaloyl-2-(15′-tetracosenamido)-4-octadecene-1,3-diol (45)

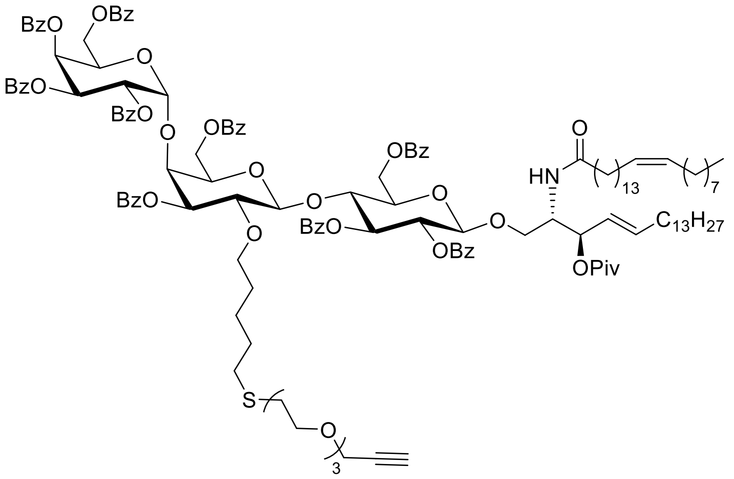

The reduction of azide **24** (16.0 mg, 7.60 µmol, 1.00 eq.) was performed according to general procedure **GP3** with triphenylphosphine (4.98 mg, 19.0 µmol, 2.50 eq.) in benzene (1.5 mL) and water (3 drops). The resulting amine was dissolved in THF (0.80 mL) and was reacted with nervonic acid (**13**) (3.62 mg, 9.88 µmol, 1.30 eq.), DIPEA (1.72 µL, 1.28 mg, 9.88 µmol, 1.30 eq.) and a solution of HATU (3.76 mg, 9.88 µmol, 1.30 eq.) in DMF (0.40 mL). The reaction mixture was stirred for 17 h at ambient temperature. Column chromatography on silica gel

(*n*-pentane/EtOAc, 3:1 → 2:1) afforded glycolipid **45** (11.2 mg, 4.61 µmol, 61 %) as a colorless oil.

$[\alpha]_D^{22}$ = +33.4 (*c* 0.58, $CHCl_3$).

^{1}H-NMR (600 MHz, $CDCl_3$): *δ* (ppm) = 0.77–0.91 (m, 8 H), 0.91–1.01 (m, 2 H), 1.06 (s, 9 H), 1.10–1.41 (m, 56 H), 1.47–1.72 (m, 4 H), 1.88–1.97 (m_c, 2 H), 1.97–2.06 (m, 4 H), 2.09–2.20 (m, 2 H), 2.43 (t, *J* = 2.4 Hz, 1 H), 2.57 (t, *J* = 7.1 Hz, 2 H), 3.36–3.46 (m, 1 H), 3.51–3.74 (m, 14 H), 3.93–4.00 (m, 1 H), 4.03–4.18 (m, 5 H), 4.20 (d, *J* = 2.4 Hz, 2 H), 4.22–4.31 (m, 2 H), 4.46 (dd, *J* = 11.2, 5.6 Hz, 1 H), 4.49–4.58 (m, 2 H), 4.61 (t, *J* = 6.5 Hz, 1 H), 4.67 (d, *J* = 7.8 Hz, 1 H), 4.84–4.89 (m, 1 H), 5.05 (dd, *J* = 10.3, 2.9 Hz, 1 H), 5.17 (t, *J* = 7.5 Hz, 1 H), 5.29 (dd, *J* = 15.6, 7.4 Hz, 1 H), 5.32–5.41 (m, 3 H), 5.45–5.52 (m, 2 H), 5.62 (dd, *J* = 10.8, 3.5 Hz, 1 H), 5.65–5.74 (m_c, 1 H), 5.78–5.84 (m, 2 H), 5.97–6.02 (m, 1 H), 7.13–7.25 (m, 4 H), 7.32–7.48 (m, 16 H), 7.48–7.64 (m, 7 H), 7.73–7.84 (m, 6 H), 7.92–8.14 (m, 12 H).

^{13}C-NMR (151 MHz, $CDCl_3$): *δ* (ppm) = 14.1, 22.7, 22.7, 25.3, 25.5, 26.9, 27.2, 27.2, 29.0, 29.2, 29.3, 29.4, 29.4, 29.4, 29.5, 29.5, 29.5, 29.6, 29.6, 29.7, 29.7, 29.7, 29.8, 29.8, 31.2, 31.9, 31.9, 32.1, 32.2, 36.4, 38.7, 50.2, 58.4, 61.4, 61.8, 62.6, 67.6, 67.8, 67.9, 69.1, 69.1, 70.0, 70.2, 70.4, 70.6, 70.8, 72.4, 72.5, 72.5, 73.0, 73.5, 73.7, 74.6, 74.8, 75.3, 76.0, 77.6, 79.6, 97.9, 101.1, 103.0, 125.0, 128.3, 128.4, 128.4, 128.5, 128.5, 128.6, 128.7, 129.1, 129.2, 129.2, 129.3, 129.4, 129.5, 129.5, 129.6, 129.6, 129.7, 129.8, 129.8, 129.9, 129.9, 133.1, 133.1, 133.2, 133.3, 133.5, 133.7, 136.8, 165.1, 165.2, 165.2, 165.6, 165.7, 165.8, 165.8, 166.1, 172.5, 176.8.

IR (ATR): $\tilde{\nu}$ (cm^{-1}) = 2924, 2853, 1724, 1679, 1602, 1452, 1316, 1264, 1093, 1067, 1027, 851, 802, 708.

MS (ESI): *m*/*z* (%) = 1236.1 (100) $[M+2Na]^{2+}$, 2449.2 (59) $[M+Na]^+$. **HRMS** (ESI): *m*/*z* calculated for: $C_{142}H_{179}NO_{31}S$ $[M+Na]^+$: 2449.2074, found: 2449.2067.

O-(2,4,3,6-Tetra-O-benzoyl-α-D-galactopyranosyl)-(1→4)-(3,6-di-O-benzoyl-2-O-(9,12,15,18,21,24,27,30,33,36,39,42,45-tridecaoxa-6-thiaoctatetracont-47-yne-1-yl)-β-D-galactopyranosyl)-(1→4)-2,3,6-tri-O-benzoyl-β-D-glucopyranosyl-(1→1)-(2S,3R,4E,2′R,15′E)-2-(2′-hydroxy-15′-tetracosenamido)-3-O-pivaloyl-4-octadecene-1,3-diol (46)

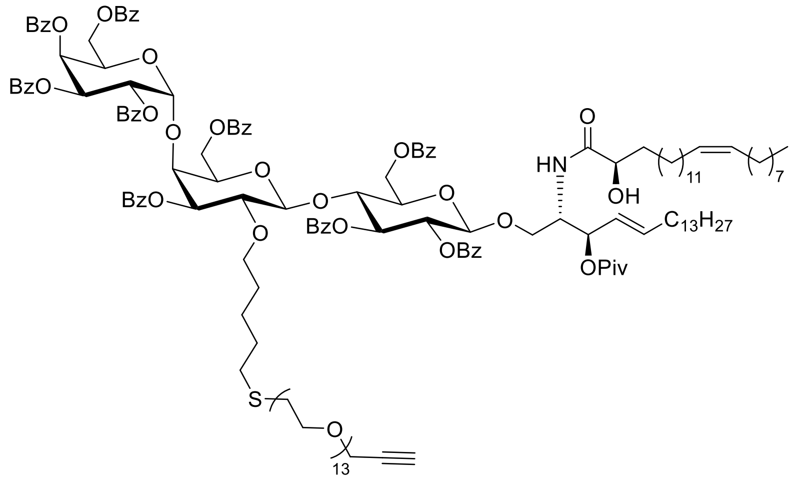

The reduction of azide **23** (25.0 mg, 9.82 µmol, 1.00 eq.) was performed according to general procedure **GP3** using triphenylphosphine (5.93 mg, 24.5 µmol, 2.50 eq.) in benzene (2.0 mL) and water (3 drops). The amine was dissolved in THF (1.0 mL) and fatty acid **14**[5] (5.62 mg, 14.7 µmol, 1.30 eq.), DIPEA (2.56 µL, 1.90 mg, 14.7 µmol, 1.30 eq.) and a solution of HATU (5.59 mg, 14.7 µmol, 1.30 eq.) in DMF (0.50 mL) were added. The reaction mixture was stirred for 17 h. Gel permeation HPLC, followed by column chromatography (SiO_2, CH_2Cl_2/MeOH, 40:1 → 30:1) and again gel permeation HPLC afforded glycolipid **46** (4.40 mg, 1.53 µmol, 16 %) as a colorless oil.

$[\alpha]_D^{25}$ = +34.3 (*c* 0.80, $CHCl_3$).

¹H-NMR (500 MHz, $CDCl_3$): *δ* (ppm) = 0.82–0.93 (m, 9 H), 1.05 (s, 9 H), 1.20–1.31 (m, 67 H), 1.84–1.95 (m, 2 H), 1.97–2.05 (m, 4 H), 2.09–2.19 (m, 1 H), 2.44 (t, *J* = 2.4 Hz, 1 H), 2.48 (d, *J* = 4.7 Hz, 1 H), 2.56 (t, *J* = 7.2 Hz, 2 H), 3.38–3.47 (m, 1 H), 3.53 (t, *J* = 7.2 Hz, 2 H), 3.56–3.74 (m, 46 H), 3.74–3.82 (m, 1 H), 3.92–4.00 (m, 2 H), 4.05–4.18 (m, 4 H), 4.20 (d, *J* = 2.4 Hz, 2 H), 4.20–4.29 (m, 1 H), 4.46 (dd, *J* = 11.2, 5.7 Hz, 1 H), 4.53 (dd, *J* = 12.1, 4.3 Hz, 1 H), 4.58 (d, *J* = 7.5 Hz, 1 H), 4.58–4.65 (m, 1 H), 4.74 (d, *J* = 7.8 Hz, 1 H), 4.89–4.96 (m_c, 1 H), 5.06 (dd, *J* = 10.3, 2.9 Hz, 1 H), 5.14–5.21 (m_c, 1 H), 5.27 (dd, *J* = 15.4, 7.4 Hz, 1 H), 5.33–5.40 (m, 3 H), 5.48 (d, *J* = 3.5 Hz, 1 H), 5.57–5.69 (m, 2 H), 5.78 (t, *J* = 8.8 Hz, 1 H), 5.81 (dd, *J* = 10.3, 2.9 Hz, 1 H), 5.98 (dd, *J* = 3.5, 1.5 Hz, 1 H), 6.74 (d, *J* = 9.4 Hz, 1 H), 7.19–7.26 (m, 4 H), 7.32–7.63 (m, 23 H), 7.74–7.83 (m, 6 H), 7.91–8.12 (m, 12 H)

¹³C-NMR (151 MHz, $CDCl_3$): *δ* (ppm) = 14.1, 22.7, 25.0, 25.3, 26.9, 27.2, 27.2, 28.9, 29.2, 29.3, 29.4, 29.4, 29.5, 29.5, 29.5, 29.6, 29.6, 29.7, 29.7, 29.8, 29.8, 31.2, 31.9, 31.9, 32.1, 32.2, 34.5, 38.7, 50.0, 58.4, 61.4, 61.8, 62.4, 66.6, 67.6, 67.9, 69.0, 69.1, 70.0, 70.2, 70.4, 70.5, 70.6, 70.8, 72.0, 72.4, 72.4, 72.6, 73.0, 73.6, 73.7, 74.6, 74.8, 75.2, 75.9, 77.6, 79.6, 97.8, 100.2, 103.0,

124.6, 128.3, 128.4, 128.4, 128.5, 128.6, 128.7, 129.1, 129.2, 129.3, 129.4, 129.5, 129.5, 129.6, 129.6, 129.8, 129.9, 129.9, 133.1, 133.1, 133.2, 133.3, 133.4, 133.5, 133.7, 137.0, 165.2, 165.2, 165.6, 165.7, 165.8, 166.0, 166.1, 173.6, 176.8.

IR (ATR): $\tilde{\nu}$ (cm^{-1}) = 2924, 2856, 1728, 1455, 1353, 1315, 1270, 1102, 1037, 1031, 845, 713.

MS (ESI): *m/z* (%) = 984.1 (30) [M+3Na]$^{3+}$, 1464.7 (100) [M+2Na]$^{2+}$, 2906.5 (2) [M+Na]$^{+}$. **HRMS** (ESI): *m/z* calculated for: $C_{162}H_{219}NO_{42}S$ [M+Na]$^{+}$: 2906.4678, found: 2906.4626.

_O_-(2,4,3,6-Tetra-_O_-benzoyl-α-D-galactopyranosyl)-(1→4)-(3,6-di-_O_-benzoyl-2-_O_-(9,12,15-trioxa-6-thiaoctadec-17-yne-1-yl)-β-D-galactopyranosyl)-(1→4)-2,3,6-tri-_O_-benzoyl-β-D-glucopyranosyl-(1→1)-(2_S_,3_R_,4_E_,2′_R_,15′_E_)-2-(2′-hydroxy-15′-tetracosenamido)-3-_O_-pivaloyl-4-octadecene-1,3-diol (47)

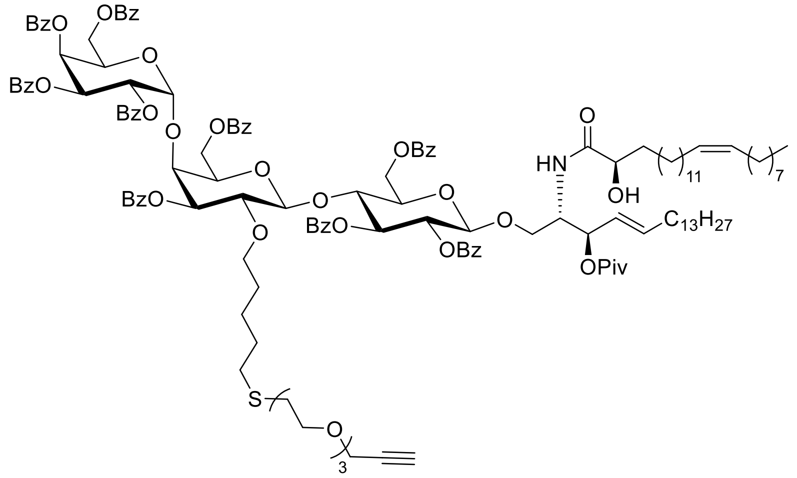

The reduction of azide **24** (22.0 mg, 10.4 µmol, 1.00 eq.) was performed according to general procedure **GP3** with triphenylphosphine (6.85 mg, 26.1 µmol, 2.50 eq.) in benzene (5.2 mL) and water (5 drops). The resulting amine was dissolved in THF (1.1 mL) and was reacted with fatty acid **14**[5] (5.17 mg, 13.5 µmol, 1.30 eq.), DIPEA (2.35 µL, 1.75 mg, 13.5 µmol, 1.30 eq.) and a solution of HATU (5.13 mg, 13.5 µmol, 1.30 eq.) in DMF (0.55 mL). The reaction mixture was stirred for 16 h at ambient temperature. Column chromatography on silica gel (*n*-pentane/EtOAc, 3:1 → 2:1) afforded glycolipid **47** (11.7 mg, 4.79 µmol, 46 %) as a colorless oil.

$[\alpha]_D^{28}$ = +37.4 (*c* 1.17, $CHCl_3$).

^{1}H-NMR (600 MHz, $CDCl_3$): *δ* (ppm) = 0.80–0.91 (m, 8 H), 0.93–1.00 (m, 2 H), 1.05 (s, 9 H), 1.11–1.41 (m, 59 H), 1.87–1.94 (m, 2 H), 1.98–2.04 (m, 4 H), 2.09–2.23 (m, 2 H), 2.43 (t, *J* = 2.4 Hz, 1 H), 2.48 (d, *J* = 4.6 Hz, 1 H), 2.57 (t, *J* = 7.1 Hz, 2 H), 3.43 (dd, *J* = 15.9, 6.9 Hz, 1 H), 3.54 (t, *J* = 7.1 Hz, 2 H), 3.56–3.72 (m, 11 H), 3.75–3.81 (m, 1 H), 3.92–4.00 (m, 2 H), 4.05–4.18 (m, 4 H), 4.19 (d, *J* = 2.4 Hz, 2 H), 4.20–4.30 (m, 2 H), 4.45 (dd, *J* = 11.2, 5.7 Hz, 1 H), 4.53 (dd, *J* = 12.1, 4.4 Hz, 1 H), 4.58 (d, *J* = 7.5 Hz, 1 H), 4.59–4.64 (m_c, 1 H), 4.74 (d, *J* = 7.8 Hz, 1 H), 4.90–4.96 (m, 1 H), 5.07 (dd, *J* = 10.3, 2.9 Hz, 1 H), 5.18 (t, *J* = 7.3 Hz, 1 H),

5.27 (dd, *J* = 15.3, 7.4 Hz, 1 H), 5.32–5.39 (m, 3 H), 5.48 (d, *J* = 3.5 Hz, 1 H), 5.60–5.69 (m, 2 H), 5.78 (t, *J* = 9.4 Hz, 1 H), 5.81 (dd, *J* = 10.9, 3.5 Hz, 1 H), 5.99 (dd, *J* = 3.5, 1.4 Hz, 1 H), 6.75 (d, *J* = 9.4 Hz, 1 H), 7.16–7.25 (m, 5 H), 7.32–7.47 (m, 16 H), 7.47–7.60 (m, 6 H), 7.74–7.85 (m, 6 H), 7.92–8.15 (m, 12 H).

^{13}C-NMR (151 MHz, $CDCl_3$): *δ* (ppm) = 14.1, 22.7, 22.7, 25.0, 25.3, 26.9, 27.2, 27.2, 28.8, 29.2, 29.3, 29.4, 29.4, 29.5, 29.5, 29.6, 29.6, 29.7, 29.7, 29.8, 29.8, 31.2, 31.9, 31.9, 32.1, 32.2, 34.5, 38.7, 50.0, 58.4, 61.4, 61.8, 62.4, 66.5, 67.6, 67.9, 69.0, 69.1, 70.0, 70.2, 70.4, 70.6, 70.8, 72.0, 72.4, 72.4, 72.6, 73.0, 73.6, 73.7, 74.6, 74.8, 75.2, 75.9, 77.6, 79.6, 97.8, 100.2, 103.0, 124.6, 128.3, 128.4, 128.4, 128.5, 128.6, 128.7, 129.1, 129.2, 129.3, 129.4, 129.5, 129.5, 129.6, 129.6, 129.8, 129.9, 129.9, 133.1, 133.1, 133.2, 133.3, 133.4, 133.5, 133.7, 137.0, 165.2, 165.2, 165.6, 165.7, 165.8, 166.0, 166.1, 173.6, 176.8.

IR (ATR): $\tilde{\nu}$ (cm^{-1}) = 2925, 2855, 1728, 1676, 1454, 1316, 1270, 1100, 1070, 1005, 712.

MS (ESI): *m*/*z* (%) = 1244.1 (100) $[M+2Na]^{2+}$, 2465.2 (83) $[M+Na]^+$. **HRMS** (ESI): *m*/*z* calculated for: $C_{142}H_{179}NO_{32}S$ $[M+Na]^+$: 2465.2023, found: 2465.2007.

O-(α-D-Galactopyranosyl)-(1→4)-(2-*O*-(9,12,15,18,21,24,27,30,33,36,39,42,45-tridecaoxa-6-thiaoctatetracont-47-yne-1-yl)-β-D-galactopyranosyl)-(1→4)-β-D-glucopyranosyl-(1→1)-(2*S*,3*R*,4*E*)-2-(tetracosanamido)-4-octadecene-1,3-diol (25)

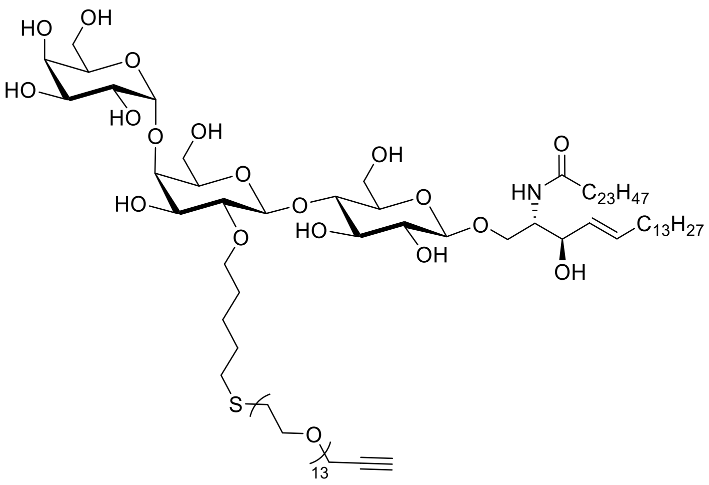

The deprotection of glycolipid **40** (14.0 mg, 4.88 µmol, 1.00 eq.) was performed according to general procedure **GP4** in methanol (0.75 mL) and dichloromethane (0.25 mL) for 24 h at 50 °C. After a dialysis of 2 d with 5 L of H_2O and subsequent lyophilization glycolipid **25** (6.60 mg, 3.57 µmol, 73 %) was obtained as a colorless powder.

$[\alpha]_D^{21}$ = +20.4 (*c* 0.54, $CHCl_3$/MeOH/H_2O, 1.6:1:0.2).

^{1}H-NMR (600 MHz, $CDCl_3$/MeOD/D_2O, 1.6:1.0:0.2): *δ* (ppm) = 0.76–0.94 (m, 8 H), 1.02–1.47 (m, 60 H), 1.55–1.65 (m, 6 H), 1.68–1.90 (m, 3 H), 1.98–2.06 (m, 3 H), 2.17 (t, *J* = 7.6 Hz, 2 H),

2.19–2.25 (m, 1 H), 2.56 (t, J = 7.3 Hz, 2 H), 2.61–2.66 (m, 1 H), 2.69–2.75 (m_c, 2 H), 2.80–2.86 (m_c, 1 H), 3.23–3.29 (m, 1 H), 3.36–3.42 (m, 2 H), 3.50–3.60 (m, 3 H), 3.57–3.91 (m, 53 H), 3.95–4.01 (m, 3 H), 4.08 (t, J = 8.1 Hz, 1 H), 4.15–4.21 (m, 1 H), 4.22 (d, J = 2.3 Hz, 2 H), 4.27 (t, J = 6.1 Hz, 1 H), 4.44 (d, J = 7.7 Hz, 1 H), 4.94 (d, J = 3.2 Hz, 1 H), 5.44 (dd, J = 15.4, 7.7 Hz, 1 H), 5.66–5.75 (m, 1 H).

Further signals were covered by the signals of the solvents.

^{13}C-NMR (151 MHz, $CDCl_3$/MeOD/D_2O, 1.6:1.0:0.2): δ (ppm) = 14.4, 23.1, 23.1, 25.6, 26.4, 29.7, 29.8, 29.8, 29.9, 29.9, 29.9, 30.0, 30.0, 30.1, 30.1, 30.2, 31.6, 32.3, 32.4, 32.7, 32.9, 36.9, 53.6, 58.7, 60.8, 62.0, 64.1, 67.1, 69.3, 70.0, 70.1, 70.1, 70.5, 70.5, 70.6, 70.6, 70.7, 71.1, 71.8, 72.2, 73.5, 73.7, 73.9, 75.0, 75.4, 75.5, 75.6, 79.4, 79.7, 80.3, 101.6, 103.2, 103.6, 129.7, 135.1, 175.1.

IR (ATR): $\tilde{\nu}$ (cm^{-1}) = 3343, 2918, 2850, 1639, 1546, 1464, 1355, 1296, 1257, 1076, 1028, 808, 719.

MS (ESI): m/z (%) = 947.1 (100) $[M+2Na]^{2+}$, 1871.2 (11) $[M+Na]^+$. **HRMS** (ESI): m/z calculated for: $C_{94}H_{177}NO_{31}S$ $[M+Na]^+$: 1871.1918, found: 1871.1925.

_O_-(α-D-Galactopyranosyl)-(1→4)-(2-_O_-(9,12,15-trioxa-6-thiaoctadec-17-yne-1-yl)-β-D-galactopyranosyl)-(1→4)-β-D-glucopyranosyl-(1→1)-(2_S_,3_R_,4_E_)-2-(tetracosanamido)-4-octadecene-1,3-diol (26)

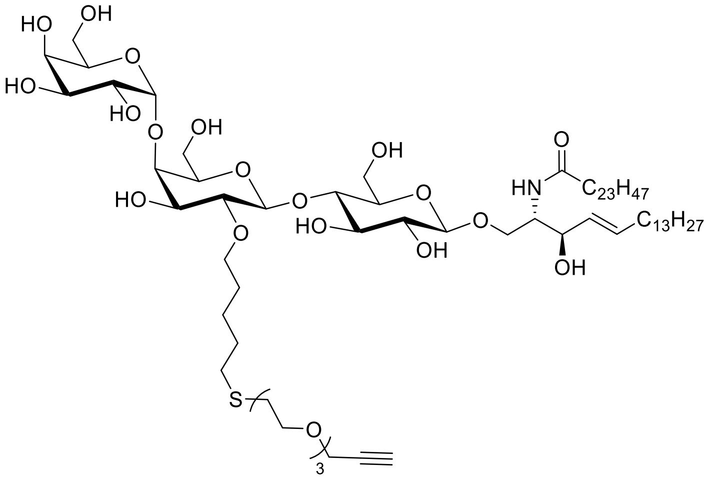

The deprotection of glycolipid **41** (12.5 mg, 5.14 µmol, 1.00 eq.) was performed according to general procedure **GP4** in methanol (2.0 mL) and dichloromethane (0.60 mL) in 24 h at 50 °C. After dialysis (2 d with 5 L H_2O) and subsequent lyophilization deprotected glycolipid **26** (7.60 mg, 5.04 µmol, 98 %) was obtained as a colorless powder.

$[\alpha]_D^{21}$ = +23.5 (c 0.60, $CHCl_3$/MeOH/H_2O, 1.6:1.0:0.2).

^{1}H-NMR (600 MHz, $CDCl_3$/MeOD/D_2O, 1.6:1.0:0.2): δ (ppm) = 0.89 (t, J = 6.9 Hz, 6 H), 1.11–1.64 (m, 64 H), 1.96–2.07 (m, 2 H), 2.17 (t, J = 7.6 Hz, 2 H), 2.57 (t, J = 7.3 Hz, 2 H), 2.63 (t, J = 2.4 Hz, 1 H), 2.72 (t, J = 6.9 Hz, 2 H), 3.26 (dd, J = 10.0, 7.7 Hz, 1 H), 3.35–3.41 (m, 1 H), 3.52–3.59 (m, 2 H), 3.60–3.75 (m, 14 H), 3.76–3.91 (m, 7 H), 3.94–4.01 (m, 3 H), 4.05–4.11 (m_c, 1 H), 4.15–4.23 (m, 1 H), 4.22 (d, J = 2.4 Hz, 2 H), 4.24–4.30 (m_c, 1 H), 4.32 (d, J = 7.8 Hz, 1 H), 4.44 (d, J = 7.8 Hz, 1 H), 4.94 (d, J = 3.2 Hz, 1 H), 5.44 (dd, J = 15.4, 7.7 Hz, 1 H), 5.66–5.74 (m_c, 1 H).

Further signals were covered by signals of the solvent mixture.

^{13}C-NMR (151 MHz, $CDCl_3$/MeOD/D_2O, 1.6:1.0:0.2): δ (ppm) = 14.5, 14.5, 23.2, 23.3, 25.7, 26.6, 29.9, 29.9, 30.0, 30.0, 30.1, 30.2, 30.2, 30.2, 30.3, 30.3, 30.4, 31.8, 32.5, 32.5, 32.8, 33.0, 37.1, 53.7, 58.9, 60.4, 61.0, 62.1, 69.3, 69.5, 69.6, 70.2, 70.3, 70.7, 70.8, 70.9, 71.3, 72.0, 72.4, 73.6, 73.8, 74.1, 75.2, 75.6, 75.7, 75.8, 78.8, 79.5, 79.9, 80.5, 101.7, 103.4, 103.8, 129.8, 135.3, 175.3.

IR (ATR): $\tilde{\nu}$ (cm^{-1}) = 3335, 2918, 2850, 1638, 1549, 1465, 1373, 1292, 1256, 1076, 1027, 719.

MS (ESI): m/z (%) = 727.0 (80) $[M+2Na]^{2+}$, 1430.9 (100) $[M+Na]^+$. **HRMS** (ESI): m/z calculated for: $C_{74}H_{137}NO_{21}S$ $[M+Na]^+$: 1430.9296, found: 1430.9298.

***O*-(α-D-Galactopyranosyl)-(1→4)-(2-*O*-(9,12,15,18,21,24,27,30,33,36,39,42,45-tridecaoxa-6-thiaoctatetracont-47-yne-1-yl)-β-D-galactopyranosyl)-(1→4)-β-D-glucopyranosyl-(1→1)-(2*S*,3*R*,4*E*,2′*R*)-2-(2′-hydroxytetracosanamido)-4-octadecene-1,3-diol (27)**

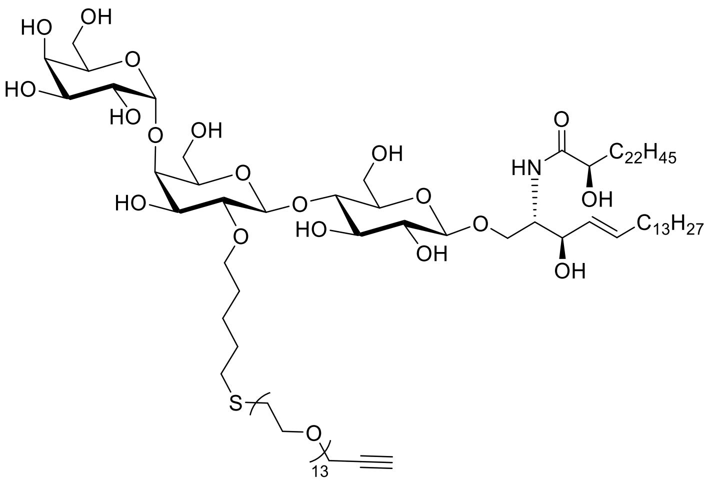

The deprotection of glycolipid **42** (14.0 mg, 4.68 µmol, 1.00 eq.) was performed according to general procedure **GP4** in methanol (1.8 mL) and dichloromethane (0.60 mL) for 30 h at 50 °C. After dialysis (2 d with 5 L of H_2O) and subsequent lyophilization glycolipid **27** (6.50 mg, 3.48 µmol, 74 %) was obtained as a colorless powder.

$[\alpha]_D^{24}$ = +19.5 (c 0.65, $CHCl_3$/MeOH/H_2O, 1.6:1.0:0.2).

^{1}H-NMR (600 MHz, $CDCl_3$/MeOD/D_2O, 1.6:1.0:0.2): δ (ppm) = 0.82–1.01 (m, 9 H), 1.07–1.52 (m, 66 H), 1.52–1.65 (m, 7 H), 1.66–1.76 (m, 2 H), 1.98–2.07 (m, 3 H), 2.54–2.60 (m_c, 2 H), 2.63 (t, *J* = 2.4 Hz, 1 H), 2.72 (t, *J* = 6.9 Hz, 2 H), 3.26 (dd, *J* = 10.0, 7.7 Hz, 1 H), 3.29–3.32 (m_c, 1 H), 3.39 (dt, *J* = 9.7, 3.0 Hz, 1 H), 3.51–3.59 (m, 2 H), 3.59–3.78 (m, 47 H), 3.79–3.93 (m, 5 H), 3.94–4.02 (m, 3 H), 4.03 (dd, *J* = 7.9, 3.9 Hz, 1 H), 4.06–4.12 (m, 2 H), 4.22 (d, *J* = 2.4 Hz, 2 H), 4.25–4.29 (m_c, 1 H), 4.33 (d, *J* = 7.9 Hz, 1 H), 4.44 (d, *J* = 7.8 Hz, 1 H), 4.93 (d, *J* = 3.0 Hz, 1 H), 5.43 (dd, *J* = 15.4, 7.5 Hz, 1 H), 5.69–5.77 (m_c, 1 H).

Further signals were covered by signals of the solvent mixture.

^{13}C-NMR (151 MHz, $CDCl_3$/MeOD/D_2O, 1.6:1.0:0.2): δ (ppm) = 14.6, 14.6, 23.3, 23.3, 25.8, 25.8, 29.8, 29.9, 30.0, 30.1, 30.2, 30.2, 30.3, 30.3, 30.4, 31.8, 32.5, 32.5, 32.9, 33.1, 35.2, 53.7, 58.9, 60.4, 61.0, 62.1, 69.2, 69.5, 70.3, 70.7, 70.7, 70.9, 70.9, 71.2, 72.0, 72.4, 72.7, 73.7, 74.1, 75.2, 75.6, 75.7, 75.8, 78.7, 79.5, 79.9, 80.5, 101.7, 103.4, 103.8, 129.4, 135.4, 176.6.

IR (ATR): $\tilde{\nu}$ (cm^{-1}) = 3379, 2918, 2851, 1726, 1639, 1540, 1464, 1351, 1256, 1083, 1030, 804.

MS (ESI): *m*/*z* (%) = 644.4 (29) $[M+3Na]^{3+}$, 955.1 (100) $[M+2Na]^{2+}$, 1888.2 (7) $[M+Na]^+$. **HRMS** (ESI): *m*/*z* calculated for: $C_{94}H_{177}NO_{32}S$ $[M+Na]^+$: 1888.1900, found: 1888.1906.

_O_-(α-D-Galactopyranosyl)-(1→4)-(2-_O_-(9,12,15-trioxa-6-thiaoctadec-17-yne-1-yl)-β-D-galactopyranosyl)-(1→4)-β-D-glucopyranosyl-(1→1)-(2_S_,3_R_,4_E_,2′_R_)-2-(2′-hydroxy-tetracosanamido)-4-octadecene-1,3-diol (28)

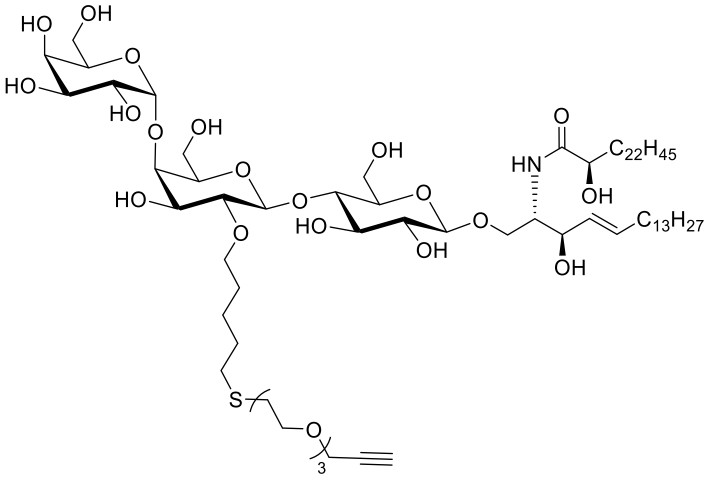

The deprotection of glycolipid **43** (17.5 mg, 6.86 µmol, 1.00 eq.) was performed according to general procedure **GP4** in methanol (2.6 mL) and dichloromethane (0.90 mL) in 22 h at 50 °C. After dialysis (2 d, 5 L H_2O) and subsequent lyophilization deprotected glycolipid **28** (7.00 mg, 4.91 µmol, 72 %) was obtained as colorless powder.

$[\alpha]_D^{25}$ = +24.4 (*c* 0.77, $CHCl_3$/MeOH/H_2O, 1.6:1.0:0.2).

^{1}H-NMR (600 MHz, $CDCl_3$/MeOD/D_2O, 1.6:1.0:0.2): δ (ppm) = 0.86–0.93 (m, 6 H), 1.14–1.47 (m, 66 H), 1.52–1.65 (m, 4 H), 1.67–1.78 (m, 2 H), 1.97–2.05 (m, 2 H), 2.54–2.60 (m_c, 2 H), 2.64 (t, *J* = 2.4 Hz, 1 H), 2.72 (t, *J* = 6.9 Hz, 2 H), 3.26 (dd, *J* = 10.0, 7.7 Hz, 1 H), 3.31 (dd, *J* = 9.3, 8.0 Hz, 1 H), 3.36–3.42 (m_c, 1 H), 3.52–3.58 (m_c, 1 H), 3.60–3.76 (m, 16 H), 3.78 (dd, *J* = 11.6, 5.3 Hz, 1 H), 3.81–3.92 (m, 5 H), 3.95–4.02 (m, 3 H), 4.03 (dd, *J* = 7.9, 3.9 Hz, 1 H), 4.06–4.11 (m, 2 H), 4.22 (d, *J* = 2.4 Hz, 2 H), 4.26–4.29 (m_c, 1 H), 4.33 (d, *J* = 7.8 Hz, 1 H), 4.44 (d, *J* = 7.7 Hz, 1 H), 4.93 (d, *J* = 2.2 Hz, 1 H), 5.43 (dd, *J* = 15.4, 7.5 Hz, 1 H), 5.69–5.77 (m_c, 1 H).

^{13}C-NMR (151 MHz, $CDCl_3$/MeOD/D_2O, 1.6:1.0:0.2): δ (ppm) = 14.6, 23.3, 23.3, 25.8, 25.8, 29.8, 30.0, 30.0, 30.1, 30.2, 30.3, 30.3, 30.4, 30.4, 30.5, 31.8, 32.6, 32.6, 32.9, 33.1, 35.2, 53.7, 58.9, 60.5, 61.0, 62.1, 69.2, 69.6, 69.6, 70.3, 70.7, 70.8, 71.0, 71.3, 72.0, 72.4, 72.7, 73.7, 74.1, 75.2, 75.7, 75.7, 75.9, 78.8, 79.5, 79.9, 80.6, 101.8, 103.4, 103.8, 129.5, 135.5, 176.7.

IR (ATR): $\tilde{\nu}$ (cm^{-1}) = 3365, 2919, 2851, 1646, 1535, 1464, 1353, 1295, 1257, 1078, 1029, 807, 718.

MS (ESI): *m*/*z* (%) = 1446.9 (100) $[M+Na]^+$. **HRMS** (ESI): *m*/*z* calculated for: $C_{74}H_{137}NO_{22}S$ $[M+Na]^+$: 1446.9245, found: 1446.9246.

_O_-(α-D-Galactopyranosyl)-(1→4)-(2-_O_-(9,12,15,18,21,24,27,30,33,36,39,42,45-tridecaoxa-6-thiaoctatetracont-47-yne-1-yl)-β-D-galactopyranosyl)-(1→4)-β-D-glucopyranosyl-(1→1)-(2_S_,3_R_,4_E_,15′_Z_)-2-(15′-tetracosenamido)-4-octadecene-1,3-diol (29)

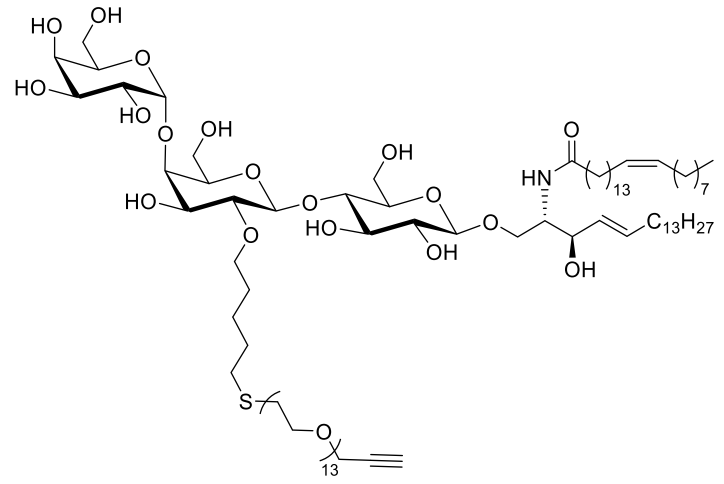

The deprotection of glycolipid **44** (10.9 mg, 3.80 µmol, 1.00 eq.) was performed according to general procedure **GP4** in methanol (0.60 mL) and dichloromethane (0.20 mL) for 28 h at 50 °C. After a dialysis of 2 d with 5 L of H_2O and subsequent lyophilization deprotected glycolipid **29** (7.00 mg, 3.79 µmol, quant.) was isolated as a colorless powder.

$[\alpha]_D^{24}$ = +22.7 (*c* 0.70, $CHCl_3$/MeOH/H_2O, 1.6:1.0:0.2).

^{1}H-NMR (600 MHz, $CDCl_3$/MeOD/D_2O, 1.6:1.0:0.2): *δ* (ppm) = 0.83–0.95 (m, 8 H), 1.10–1.49 (m, 54 H), 1.52–1.66 (m, 8 H), 1.91–2.09 (m, 6 H), 2.18 (t, *J* = 7.6 Hz, 2 H), 2.57 (t, *J* = 7.3 Hz, 2 H), 2.66 (d, *J* = 2.4 Hz, 1 H), 2.72 (t, *J* = 6.9 Hz, 2 H), 3.26 (dd, *J* = 10.0, 7.7 Hz, 1 H), 3.53–3.92 (m, 55 H), 3.95–4.00 (m, 3 H), 4.05–4.11 (m_c, 1 H), 4.19–4.24 (m, 1 H), 4.22 (d, *J* = 2.4 Hz, 2 H), 4.27 (t, *J* = 6.1 Hz, 1 H), 4.32 (d, *J* = 7.9 Hz, 1 H), 4.44 (d, *J* = 7.7 Hz, 1 H), 4.95 (d, *J* = 3.4 Hz, 1 H), 5.31–5.40 (m_c, 2 H), 5.44 (dd, *J* = 15.3, 7.8 Hz, 1 H), 5.66–5.74 (m_c, 1 H).

Further signals were covered by signals of the solvent mixture.

^{13}C-NMR (151 MHz, $CDCl_3$/MeOD/D_2O, 1.6:1:0.2): *δ* (ppm) = 14.5, 23.3, 23.3, 25.8, 26.7, 27.8, 29.9, 29.9, 29.9, 30.0, 30.1, 30.1, 30.1, 30.2, 30.2, 30.2, 30.3, 30.3, 30.3, 31.8, 32.5, 32.5, 32.9, 33.0, 37.1, 53.8, 58.9, 60.5, 61.0, 62.2, 69.4, 69.5, 69.6, 70.3, 70.7, 70.7, 70.9, 70.9, 71.3, 72.1, 72.4, 73.8, 73.9, 74.1, 75.2, 75.6, 75.7, 75.8, 79.0, 79.7, 79.9, 80.6, 101.9, 103.4, 103.9, 130.0, 130.5, 135.3, 175.3.

IR (ATR): $\tilde{\nu}$ (cm^{-1}) = 3383, 2921, 2853, 2603, 2496, 1644, 1548, 1467, 1446, 1398, 1352, 1250, 1080, 1032, 806.

MS (ESI): *m*/*z* (%) = 638.4 (33) $[M+3Na]^{3+}$, 946.1 (100) $[M+2Na]^{2+}$, 1870.2 (9) $[M+Na]^+$. **HRMS** (ESI): *m*/*z* calculated for: $C_{94}H_{175}NO_{31}S$ $[M+Na]^+$: 1869.1761, found: 1869.1768.

***O*-(α-D-Galactopyranosyl)-(1→4)-(2-*O*-(9,12,15-trioxa-6-thiaoctadec-17-yne-1-yl)-β-D-galactopyranosyl)-(1→4)-β-D-glucopyranosyl-(1→1)-(2*S*,3*R*,4*E*,15′*Z*)-2-(15′-tetra-cosenamido)-4-octadecene-1,3-diol (30)**

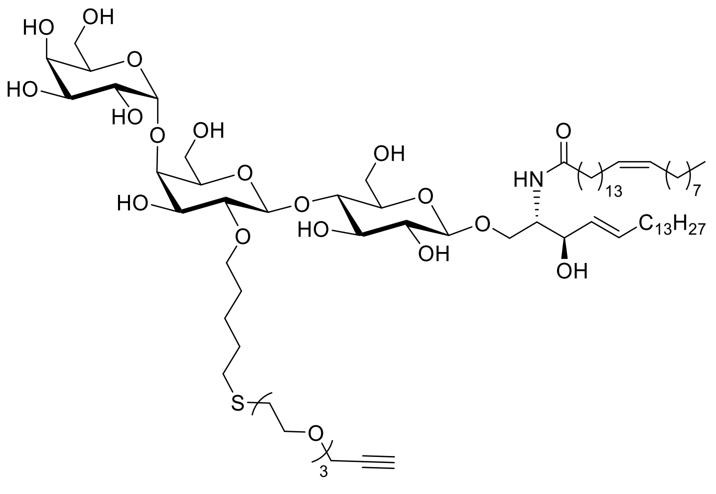

The deprotection of glycolipid **45** (17.4 mg, 7.17 µmol, 1.00 eq.) was performed according to general procedure **GP4** in methanol (2.7 mL) and dichloromethane (0.90 mL) in 24 h at 50 °C. After dialysis (2 d, 5 L H_2O) and subsequent lyophilization the unprotected glycolipid **30** (10.0 mg, 7.10 µmol, 99 %) was obtained as a colorless powder.

$[\alpha]_D^{23}$ = +18.2 (*c* 0.66, $CHCl_3$/MeOH/H_2O, 1.6:1:0.2).

^{1}H-NMR (600 MHz, $CDCl_3$/MeOD/D_2O, 1.6:1.0:0.2): δ (ppm) = 0.89 (t, J = 6.9 Hz, 6 H), 1.19–1.44 (m, 54 H), 1.50–1.66 (m, 6 H), 1.95–2.07 (m, 6 H), 2.17 (t, J = 7.6 Hz, 2 H), 2.57 (t, J = 7.3 Hz, 2 H), 2.64 (d, J = 2.3 Hz, 1 H), 2.72 (t, J = 6.9 Hz, 2 H), 3.26 (dd, J = 10.0, 7.7 Hz, 1 H), 3.36–3.41 (m, 1 H), 3.53–3.59 (m, 2 H), 3.60–3.76 (m, 14 H), 3.78–3.93 (m, 6 H), 3.92–4.01 (m, 3 H), 4.08 (t, J = 8.1 Hz, 1 H), 4.16–4.21 (m, 1 H), 4.22 (d, J = 2.4 Hz, 2 H), 4.24–4.30 (m_c, 1 H), 4.32 (d, J = 7.8 Hz, 1 H), 4.44 (d, J = 7.7 Hz, 1 H), 4.94 (d, J = 2.9 Hz, 1 H), 5.31–5.40 (m, 2 H), 5.44 (dd, J = 15.3, 7.8 Hz, 1 H), 5.65–5.74 (m, 1 H).

Further signals were covered by signals of the solvent mixture.

^{13}C-NMR (126 MHz, $CDCl_3$/MeOD/D_2O, 1.6:1.0:0.2): δ (ppm) = 14.5, 23.3, 25.7, 26.6, 27.8, 27.8, 29.9, 29.9, 30.0, 30.1, 30.2, 30.2, 30.2, 30.3, 30.3, 30.4, 30.4, 31.8, 32.5, 32.5, 32.9, 33.0, 37.1, 53.8, 58.9, 60.4, 61.0, 62.1, 69.4, 69.6, 70.3, 70.7, 70.8, 71.3, 72.0, 72.4, 73.7, 73.8, 74.1, 75.2, 75.6, 75.7, 75.8, 78.8, 79.5, 79.9, 80.5, 101.8, 103.4, 103.8, 129.9, 130.5, 135.3, 175.3.

IR (ATR): $\tilde{\nu}$ (cm^{-1}) = 3348, 3314, 2921, 2852, 2160, 2027, 1975, 1711, 1644, 1547, 1462, 1354, 1078, 1029, 807.

MS (ESI): *m*/*z* (%) = 725.5 (37) $[M+2Na]^{2+}$, 1428.9 (100) $[M+Na]^+$. **HRMS** (ESI): *m*/*z* calculated for: $C_{74}H_{135}NO_{21}S$ $[M+Na]^+$: 1428.9140, found: 1428.9142.

_O_-(α-D-Galactopyranosyl)-(1→4)-(2-_O_-(9,12,15,18,21,24,27,30,33,36,39,42,45-tridecaoxa-6-thiaoctatetracont-47-yne-1-yl)-β-D-galactopyranosyl)-(1→4)-β-D-glucopyranosyl-(1→1)-(2_S_,3_R_,4_E_,2′_R_,15′_E_)-2-(2′-hydroxy-15′-tetracosenamido)-4-octadecene-1,3-diol (31)

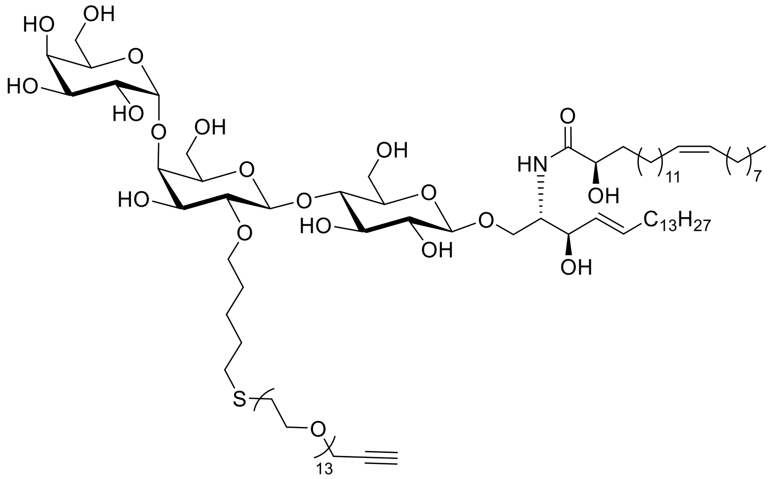

The deprotection of glycolipid **46** (9.15 mg, 3.17 µmol, 1.00 eq.) was performed according to general procedure **GP4** in methanol (1.0 mL) and dichloromethane (0.35 mL) at 50 °C in 24 h. After dialysis (2 d, 5 L H_2O) and subsequent lyophilization glycolipid **31** (5.90 mg, 3.17 µmol, quant.) was obtained as a colorless powder.

$[\alpha]_D^{22}$ = +19.5 (*c* 0.59, $CHCl_3$/MeOH/H_2O, 1.6:1.0:0.2).

^{1}H-NMR (600 MHz, $CDCl_3$/MeOD/D_2O, 1.6:1.0:0.2): δ (ppm) = 0.76–0.96 (m, 9 H), 1.07–1.47 (m, 58 H), 1.51–1.66 (m, 8 H), 1.67–1.75 (m, 2 H), 1.95–2.08 (m, 7 H), 2.57 (t, J = 7.3 Hz, 2 H), 2.63 (t, J = 2.4 Hz, 1 H), 2.72 (t, J = 6.9 Hz, 2 H), 3.23–3.29 (m_c, 1 H), 3.28–3.34 (m_c, 1 H), 3.39 (dt, J = 10.0, 3.1 Hz, 1 H), 3.52–3.58 (m, 2 H), 3.58–3.82 (m, 40 H), 3.81–3.93 (m, 5 H), 3.95–4.02 (m, 3 H), 4.03 (dd, J = 7.9, 3.9 Hz, 1 H), 4.06–4.13 (m, 2 H), 4.22 (d, J = 2.4 Hz, 2 H), 4.27 (dd, J = 7.0, 5.3 Hz, 1 H), 4.33 (d, J = 7.9 Hz, 1 H), 4.44 (d, J = 7.7 Hz, 1 H), 4.93 (d, J = 3.1 Hz, 1 H), 5.26–5.40 (m_c, 2 H), 5.43 (dd, J = 15.3, 7.5 Hz, 1 H), 5.69–5.78 (m_c, 1 H).

Further signals were covered by signals of the solvent mixture.

^{13}C-NMR (151 MHz, $CDCl_3$/MeOD/D_2O, 1.6:1.0:0.2): δ (ppm) = 14.5, 14.6, 14.6, 23.3, 23.3, 25.8, 25.8, 26.3, 27.8, 27.8, 29.8, 29.8, 29.9, 29.9, 29.9, 29.9, 30.0, 30.1, 30.1, 30.2, 30.2, 30.3, 30.3, 30.3, 30.4, 30.4, 30.5, 30.5, 31.8, 32.5, 32.6, 32.9, 33.1, 35.2, 36.3, 49.6, 53.7, 58.9, 60.4, 61.0, 62.1, 69.2, 69.5, 70.3, 70.7, 70.7, 70.8, 70.9, 70.9, 71.2, 72.0, 72.4, 72.7, 73.7, 74.1, 75.2, 75.6, 75.7, 75.9, 78.8, 79.5, 79.9, 80.5, 101.8, 103.4, 103.8, 129.5, 130.4, 130.5, 130.5, 130.6, 135.4, 176.6.

IR (ATR): $\tilde{\nu}$ (cm^{-1}) = 3373, 2919, 2853, 2025, 1972, 1737, 1642, 1537, 1461, 1297, 1083, 1033, 805.

MS (ESI): m/z (%) = 644.1 (30) $[M+3Na]^{3+}$, 954.6 (100) $[M+2Na]^{2+}$, 1886.2 (6) $[M+Na]^+$. **HRMS** (ESI): m/z calculated for: $C_{94}H_{175}NO_{32}S$ $[M+Na]^+$: 1886.1744, found: 1886.1756.

_O_-(α-D-Galactopyranosyl)-(1→4)-(2-_O_-(9,12,15-trioxa-6-thiaoctadec-17-yne-1-yl)-β-D-galactopyranosyl)-(1→4)-β-D-glucopyranosyl-(1→1)-(2_S_,3_R_,4_E_,2′_R_,15′_E_)-2-(2′-hydroxy-15′-tetracosenamido)-4-octadecene-1,3-diol (32)

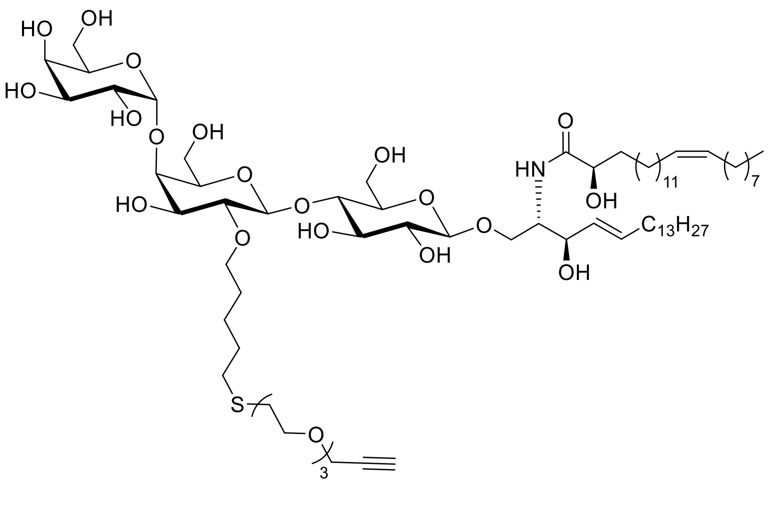

The deprotection of glycolipid **47** (11.6 mg, 4.75 µmol, 1.00 eq.) was performed according to general procedure **GP4** in methanol (1.8 mL) and dichloromethane (0.60 mL) in 24 h at 50 °C. After dialysis (2 d, 5 L H_2O) and subsequent lyophilization the deprotected glycolipid **32** (6.70 mg, 4.71 µmol, 99 %) was obtained as a colorless powder.

$[\alpha]_D^{32}$ = +24.0 (*c* 0.73, $CHCl_3/MeOH/H_2O$, 1.6:1.0:0.2).

^{1}H-NMR (600 MHz, $CDCl_3/MeOD/D_2O$, 1.6:1.0:0.2): *δ* (ppm) = 0.89 (t, *J* = 6.9 Hz, 6 H), 1.04–1.48 (m, 56 H), 1.50–1.67 (m, 6 H), 1.68–1.77 (m, 2 H), 1.95–2.08 (m, 6 H), 2.57 (t, *J* = 7.3 Hz, 2 H), 2.63–2.67 (m, 1 H), 2.72 (t, *J* = 6.9 Hz, 2 H), 3.26 (dd, *J* = 10.0, 7.7 Hz, 1 H), 3.28–3.34 (m_c, 1 H), 3.36–3.42 (m, 1 H), 3.52–3.58 (m_c, 1 H), 3.59–3.76 (m, 16 H), 3.77 (dd, *J* = 9.1, 4.3 Hz, 1 H), 3.80–3.94 (m, 5 H), 3.95–4.01 (m, 3 H), 4.03 (dd, *J* = 7.9, 3.8 Hz, 1 H), 4.06–4.12 (m, 2 H), 4.22 (d, *J* = 2.4 Hz, 2 H), 4.27 (t, *J* = 6.2 Hz, 1 H), 4.33 (d, *J* = 7.9 Hz, 1 H), 4.44 (d, *J* = 7.8 Hz, 1 H), 4.94 (d, *J* = 3.1 Hz, 1 H), 5.31–5.40 (m, 2 H), 5.43 (dd, *J* = 15.3, 7.6 Hz, 1 H), 5.68–5.77 (m_c, 1 H).

Further signals were covered by signals of the solvent mixture.

^{13}C-NMR (151 MHz, $CDCl_3/MeOD/D_2O$, 1.6:1.0:0.2): *δ* (ppm) = 14.5, 23.3, 23.3, 25.7, 25.8, 27.8, 27.8, 29.8, 29.9, 29.9, 29.9, 30.0, 30.1, 30.1, 30.2, 30.2, 30.3, 30.3, 30.3, 30.4, 30.4, 31.8, 32.5, 32.5, 32.9, 33.0, 35.2, 53.7, 58.9, 60.4, 61.0, 62.1, 64.2, 69.2, 69.5, 69.6, 70.3, 70.3, 70.7, 70.8, 70.8, 71.0, 71.0, 71.3, 72.0, 72.4, 72.7, 73.7, 73.7, 74.1, 75.2, 75.6, 75.7, 75.8, 75.8, 78.7, 79.5, 79.9, 80.5, 101.7, 103.4, 103.8, 129.4, 130.5, 135.4, 176.7.

IR (ATR): $\tilde{\nu}$ (cm^{-1}) = 3371, 2920, 2852, 2327, 2116, 1644, 1535, 1461, 1355, 1298, 1254, 1079, 1030, 809.

MS (ESI): *m*/*z* (%) = 1444.9 (100) $[M+Na]^+$. **HRMS** (ESI): *m*/*z* calculated for: $C_{74}H_{135}O_{22}NS$ $[M+Na]^+$: 1444.9089, found: 1444.9092.

_O_-(α-D-Galactopyranosyl)-(1→4)-(2-_O_-(46-(1-(3-BodipyFL-amidopropyl)-1_H_-1,2,3-triazol-4-yl)-9,12,15,18,21,24,27,30,33,36,39,42,45-tridecaoxa-6-thiahexatetracontan-1-yl)-β-D-galactopyranosyl)-(1→4)-β-D-glucopyranosyl-(1→1)-(2_S_,3_R_,4_E_)-2-(tetracosanamido)-4-octadecene-1,3-diol (1)

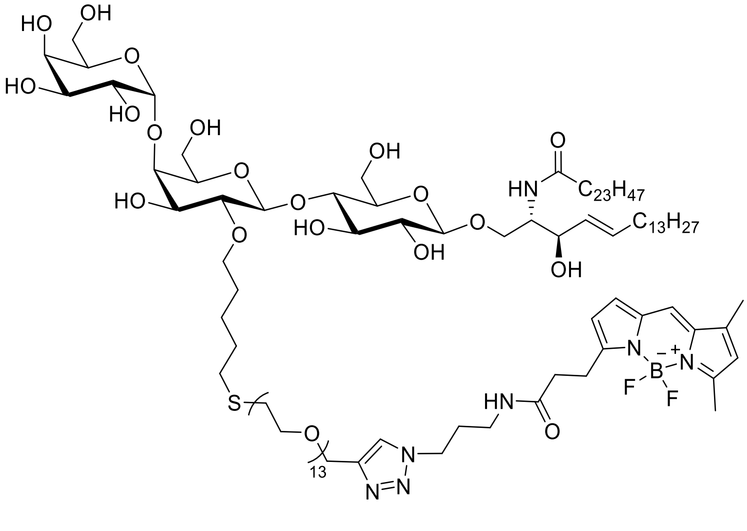

The cycloaddition of glycolipid **25** (7.00 mg, 3.78 µmol, 1.00 eq.) with dye **9** (1.70 mg, 4.54 µmol, 1.20 eq.) was performed according to general procedure **GP5** with copper powder (0.960 mg, 15.1 µmol, 4.00 eq.) and $CuSO_4 \cdot 5\ H_2O$ (0.1 M in H_2O, 7.56 µL, 0.756 µmol, 20 mol%) in water (90.0 µL) and *tert*-butanol (90.0 µL). The crude product was washed with *n*-hexane for several times to remove all plasticizers. After a dialysis of 5 d (10 L of H_2O) and subsequent lyophilization glycolipid **1** (6.00 mg, 2.70 µmol, 71 %) was isolated as a red powder.

$[\alpha]_D^{23}$ = +23.8 (*c* 0.60, $CHCl_3$/MeOH/H_2O, 1.6:1.0:0.2).

^{1}H-NMR (600 MHz, $CDCl_3$/MeOD/D_2O, 1.6:1.0:0.2): δ (ppm) = 0.73–0.97 (m, 8 H), 1.05–1.47 (m, 58 H), 1.50–1.65 (m, 8 H), 1.68–1.88 (m, 4 H), 1.97–2.05 (m, 3 H), 2.05–2.11 (m_c, 2 H), 2.14–2.20 (m_c, 2 H), 2.19–2.24 (m, 1 H), 2.29 (s, 3 H), 2.56 (s, 3 H), 2.54–2.59 (m, 1 H), 2.65 (t, *J* = 7.7 Hz, 2 H), 2.69–2.75 (m_c, 2 H), 2.79–2.86 (m, 1 H), 2.92–2.98 (m, 1 H), 3.03–3.11 (m, 1 H), 3.21 (t, *J* = 6.5 Hz, 2 H), 3.23–3.30 (m, 3 H), 3.35–3.43 (m, 2 H), 3.49–3.87 (m, 52 H), 3.95–4.01 (m, 3 H), 4.05–4.10 (m, 2 H), 4.16–4.23 (m, 2 H), 4.24–4.30 (m, 1 H), 4.94 (s, 1 H), 5.40–5.49 (m_c, 1 H), 5.66–5.75 (m_c, 1 H), 6.18 (s, 1 H), 6.33 (d, *J* = 4.0 Hz, 1 H), 6.95 (d, *J* = 4.0 Hz, 1 H), 7.21 (s, 1 H), 7.87 (s, 1 H).

Further signals were covered by signals of the solvent mixture.

^{13}C-NMR (151 MHz, $CDCl_3$/MeOD/D_2O, 1.6:1.0:0.2): δ (ppm) = 11.7, 14.6, 15.3, 23.3, 25.3, 25.7, 26.6, 27.8, 29.9, 29.9, 30.0, 30.0, 30.1, 30.2, 30.3, 30.3, 30.4, 30.4, 31.8, 35.8, 36.8, 37.1, 52.5, 53.7, 61.0, 62.0, 62.1, 64.2, 64.7, 69.4, 69.5, 70.1, 70.3, 70.7, 70.8, 71.0, 71.2, 72.0, 72.4, 73.7, 73.8, 74.1, 75.2, 75.6, 75.8, 78.8, 79.5, 80.5, 101.8, 103.4, 103.8, 117.5, 121.3, 124.8, 129.0, 129.8, 134.0, 135.3, 135.9, 145.2, 157.5, 161.2, 174.1, 175.3.

^{19}F-NMR (471 MHz, $CDCl_3$/MeOD/D_2O, 1.6:1.0:0.2): δ (ppm) = −143.1 (q, *J* = 32.9 Hz).

IR (ATR): $\tilde{\nu}$ (cm^{-1}) = 3306, 2920, 2852, 2160, 1734, 1647, 1605, 1462, 1450, 1251, 1080, 1031, 803.

MS (ESI): *m*/*z* (%) = 1134.7 (100) $[M+2Na]^{2+}$, 2246.4 (5) $[M+Na]^+$. **HRMS** (ESI): *m*/*z* calculated for: $C_{111}H_{198}O_{32}SBF_2N_7$ $[M+Na]^+$: 2246.3789, found: 2246.3776.

_O_-(α-D-Galactopyranosyl)-(1→4)-(2-_O_-(16-(1-(3-BodipyFL-amidopropyl)-1_H_-1,2,3-triazol-4-yl)-9,12,15-trioxa-6-thiahexandecan-1-yl)-β-D-galactopyranosyl)-(1→4)-β-D-glucopyranosyl-(1→1)-(2_S_,3_R_,4_E_)-2-(tetracosanamido)-4-octadecene-1,3-diol (2)

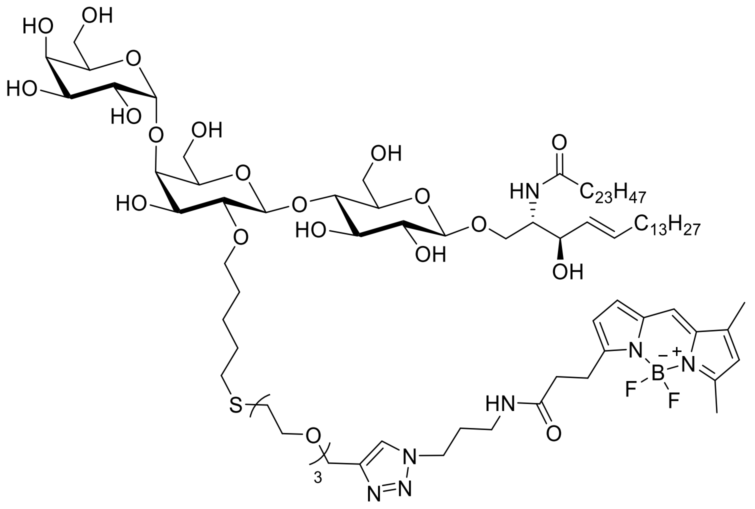

The cycloaddition of glycolipid **26** (8.20 mg, 5.82 µmol, 1.00 eq.) with dye **9** (2.61 mg, 6.98 µmol, 1.20 eq.) was performed according to general procedure **GP5** using copper powder (1.48 mg, 23.3 µmol, 4.00 eq.) and $CuSO_4 \cdot 5\,H_2O$ (0.1 M in H_2O, 11.6 µL, 1.16 µmol, 20 mol%) in water (140 µL) and *tert*-butanol (140 µL). The dialysis (5 d, 12.5 L H_2O) and the subsequent lyophilization were carried out twice to remove all traces of unreacted dye. Glycolipid **2** (5.60 mg, 3.14 µmol, 54 %) was isolated as a red powder.

$[\alpha]_D^{23}$ = +99.4 (*c* 0.90, $CHCl_3$/MeOH/H_2O, 1.6:1.0:0.2).

^{1}H-NMR (500 MHz, $CDCl_3$/MeOD/D_2O, 1.6:1.0:0.2): *δ* (ppm) = 0.78–0.95 (m, 8 H), 1.06–1.48 (m, 64 H), 1.50–1.66 (m, 6 H), 1.97–2.05 (m, 3 H), 2.08 (t, *J* = 6.7 Hz, 2 H), 2.13–2.20 (m_c, 2 H), 2.29 (s, 3 H), 2.55 (s, 3 H), 2.59–2.68 (m, 3 H), 2.71–2.85 (m, 2 H), 3.15–3.29 (m, 4 H), 3.34–3.42 (m, 1 H), 3.51–3.76 (m, 16 H), 3.78–3.92 (m, 6 H), 3.94–4.00 (m, 3 H), 4.04–4.11 (m_c, 1 H), 4.20 (dd, *J* = 9.9, 3.9 Hz, 1 H), 4.23–4.28 (m, 1 H), 4.89–4.98 (m_c, 1 H), 5.44 (dd, *J* = 15.3, 7.7 Hz, 1 H), 5.65–5.75 (m_c, 1 H), 6.18 (s, 1 H), 6.33 (d, *J* = 4.1 Hz, 1 H), 6.95 (d, *J* = 4.1 Hz, 1 H), 7.22 (s, 1 H), 7.92 (s, 1 H).

^{13}C-NMR (126 MHz, $CDCl_3$/MeOD/D_2O, 1.6:1.0:0.2): *δ* (ppm) = 11.6, 14.5, 15.2, 23.2, 25.2, 25.7, 26.6, 27.7, 29.0, 29.9, 29.9, 30.0, 30.0, 30.2, 30.2, 30.2, 30.3, 30.3, 32.5, 32.5, 33.0, 35.7, 35.9, 36.8, 37.0, 37.3, 53.7, 60.4, 61.0, 62.1, 69.3, 69.5, 70.2, 70.2, 70.7, 70.9, 70.9, 72.1, 72.3, 73.6, 73.8, 74.0, 75.2, 75.5, 75.8, 78.9, 79.6, 80.5, 101.8, 103.4, 103.8, 117.5, 121.2, 124.8, 129.0, 129.8, 135.3, 135.9, 145.2, 157.4, 161.2, 174.1, 175.3.

^{19}F-NMR (471 MHz, $CDCl_3$/MeOD/D_2O, 1.6:1.0:0.2): *δ* (ppm) = −143.1 (m).

IR (ATR): $\tilde{\nu}$ (cm^{-1}) = 3395, 2920, 2852, 2162, 2030, 1734, 1646, 1606, 1534, 1372, 1250, 1056, 803.

MS (ESI): *m*/*z* (%) = 914.1 (100) $[M+2Na]^{2+}$, 1805.1 (31) $[M+Na]^{+}$. **HRMS** (ESI): *m*/*z* calculated for: $C_{91}H_{158}BF_2O_{22}N_7S$ $[M+Na]^{+}$: 1805.1148, found: 1805.1145.

_O_-(α-D-galactopyranosyl)-(1→4)-(2-_O_-(46-(1-(3-BodipyFL-amidopropyl)-1_H_-1,2,3-triazol-4-yl)-9,12,15,18,21,24,27,30,33,36,39,42,45-tridecaoxa-6-thiahexatetracontan-1-yl)-β-D-galactopyranosyl)-(1→4)-β-D-glucopyranosyl-(1→1)-(2_S_,3_R_,4_E_,2′_R_)-2-(2′-hydroxy-tetracosanamido)-4-octadecene-1,3-diol (3)

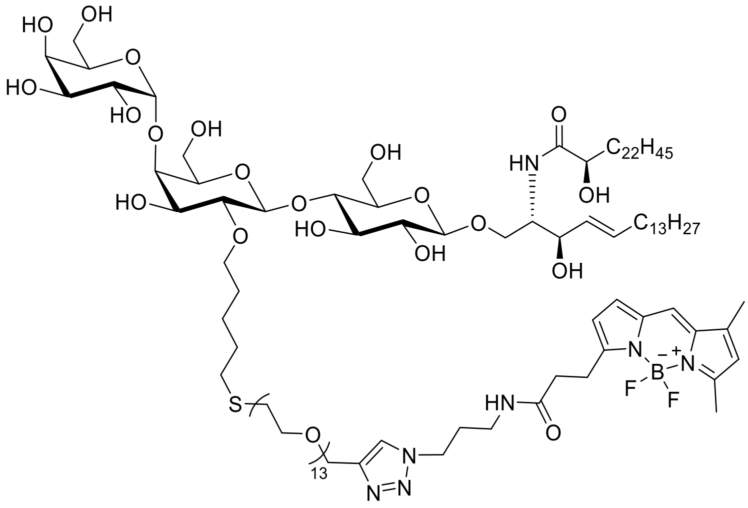

The cycloaddition of glycolipid **27** (7.00 mg, 3.75 µmol, 1.00 eq.) with dye **9** (1.62 mg, 4.32 µmol, 1.15 eq.) was performed according to **GP5** using copper powder (0.953 mg, 15.0 µmol, 4.00 eq.) and $CuSO_4 \cdot 5\ H_2O$ (0.1 M in H_2O, 7.50 µL, 0.750 µmol, 20 mol%) in water (90.0 µL) and *tert*-butanol (90.0 µL). The dialysis (5 d, 12.5 L H_2O) and the subsequent lyophilization were carried out twice to remove all traces of unreacted dye. Glycolipid **3** (6.70 mg, 2.99 µmol, 80 %) was isolated as a red powder.

$[\alpha]_D^{26}$ = +25.4 (*c* 0.60, $CHCl_3$/MeOH/H_2O, 1.6:1.0:0.2).

^{1}H-NMR (600 MHz, $CDCl_3$/MeOD/D_2O, 1.6:1.0:0.2): *δ* (ppm) = 0.73–0.84 (m, 14 H), 1.09–1.34 (m, 72 H), 1.47–1.56 (m, 6 H), 1.90–1.95 (m, 3 H), 1.95–2.02 (m, 2 H), 2.09–2.14 (m, 1 H), 2.19 (s, 3 H), 2.46 (s, 3 H), 2.55 (t, *J* = 7.6 Hz, 2 H), 2.62 (t, *J* = 6.9 Hz, 2 H), 2.70–2.77 (m, 1 H), 2.81–2.88 (m, 1 H), 2.93–3.01 (m, 1 H), 3.09–3.14 (m_c, 2 H), 3.13–3.19 (m_c, 2 H), 3.21 (dd, *J* = 9.4, 7.9 Hz, 1 H), 3.42–3.76 (m, 42 H), 3.79–3.92 (m, 3 H), 3.93 (dd, *J* = 8.0, 3.7 Hz, 1 H), 3.95–4.03 (m, 2 H), 4.17 (t, *J* = 6.2 Hz, 1 H), 4.21–4.25 (m, 1 H), 4.25 (d, *J* = 7.3 Hz, 1 H), 4.34 (d, *J* = 7.9 Hz, 1 H), 4.83 (d, *J* = 2.9 Hz, 1 H), 5.24–5.27 (m, 1H), 5.33 (dd, *J* = 15.3, 7.5 Hz, 1 H), 5.56–5.67 (m, 1 H), 6.09 (s, 1 H), 6.24 (d, *J* = 4.0 Hz, 1 H), 6.86 (d, *J* = 4.0 Hz, 1 H), 7.12 (s, 1 H), 7.75 (s, 1 H).

Further signals were covered by signals of the solvent mixture.

^{13}C-NMR (151 MHz, $CDCl_3$/MeOD/D_2O, 1.6:1.0:0.2): *δ* (ppm) = 11.7, 14.5, 15.3, 23.3, 25.3, 25.3, 25.8, 26.3, 27.8, 29.8, 29.9, 29.9, 29.9, 30.0, 30.1, 30.1, 30.2, 30.2, 30.3, 30.3, 30.4, 31.8,

32.5, 32.9, 33.0, 35.2, 35.8, 36.3, 36.8, 53.7, 55.1, 60.4, 61.0, 61.6, 62.1, 62.9, 64.2, 64.7, 69.2, 69.6, 70.2, 70.3, 70.7, 70.9, 71.3, 72.0, 72.4, 72.7, 73.7, 74.1, 75.2, 75.6, 75.9, 78.8, 79.5, 80.5, 101.8, 103.4, 103.8, 117.5, 121.3, 124.6, 124.8, 125.0, 129.0, 129.5, 130.4, 130.6, 134.0, 135.4, 135.9, 145.2, 157.5, 161.2, 174.1, 176.6.

^{19}F-NMR (471 MHz, $CDCl_3$/MeOD/D_2O, 1.6:1.0:0.2): *δ* (ppm) = −143.6 (q, *J* = 33.3 Hz).

IR (ATR): $\tilde{\nu}$ (cm^{-1}) = 3363, 2920, 2852, 2161, 2024, 1974, 1739, 1645, 1462, 1361, 1251, 1079, 801.

MS (ESI): *m*/*z* (%) = 1142.7 (100) $[M+2Na]^{2+}$, 2261.4 (6) $[M+Na]^{+}$. **HRMS** (ESI): *m*/*z* calculated for: $C_{111}H_{198}O_{33}SBF_2N_7$ $[M+Na]^{+}$: 2261.3717, found: 2261.3707.

***O*-(α-D-Galactopyranosyl)-(1→4)-(2-*O*-(16-(1-(3-BodipyFL-amidopropyl)-1*H*-1,2,3-triazol-4-yl)-9,12,15-trioxa-6-thiahexandecan-1-yl)-β-D-galactopyranosyl)-(1→4)-β-D-glucopyranosyl-(1→1)-(2*S*,3*R*,4*E*,2′*R*)-2-(2′-hydroxytetracosanamido)-4-octadecene-1,3-diol (4)**

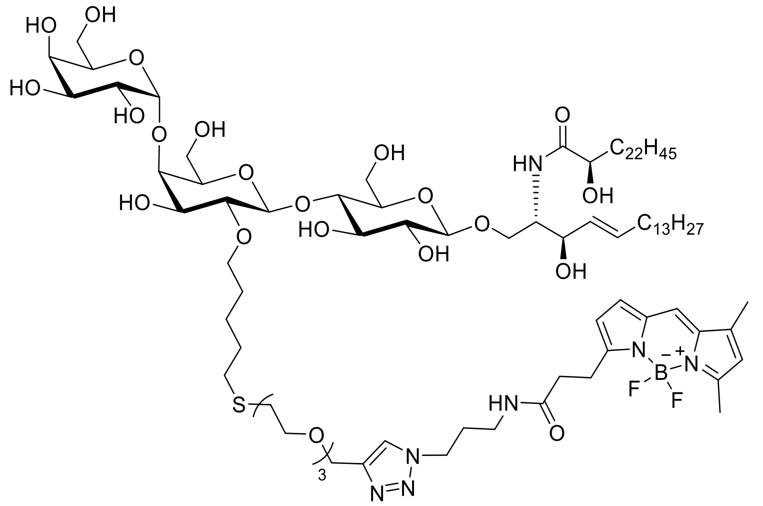

The cycloaddition of glycolipid **28** (8.00 mg, 5.61 µmol, 1.00 eq.) with dye **9** (2.52 mg, 6.74 µmol, 1.20 eq.) was performed according to general procedure **GP5** using copper powder (1.42 mg, 22.4 µmol, 4.00 eq.) and $CuSO_4{\cdot}5\ H_2O$ (0.1 M in H_2O, 11.2 µL, 1.12 µmol, 20 mol%) in water (135 µL) and *tert*-butanol (135 µL). The dialysis (5 d, 12.5 L H_2O) and the subsequent lyophilization were carried out twice to remove all traces of unreacted dye. Glycolipid **4** (7.80 mg, 4.34 µmol, 77 %) was isolated as a red powder.

$[\alpha]_D^{27}$ **=** +29.5 (*c* 0.56, $CHCl_3$/MeOH/H_2O, 1.6:1.0:0.2).

^{1}H-NMR (500 MHz, $CDCl_3$/MeOD/D_2O, 1.6:1.0:0.2): *δ* (ppm) = 0.81–0.96 (m, 10 H), 1.14–1.49 (m, 66 H), 1.47–1.66 (m, 6 H), 1.95–2.10 (m, 5 H), 2.29 (s, 3 H), 2.49–2.59 (m, 4 H), 2.65 (t, *J* = 7.6 Hz, 2 H), 2.71 (t, *J* = 6.9 Hz, 2 H), 3.18–3.33 (m, 5 H), 3.35–3.42 (m, 1 H), 3.50–3.59 (m, 2 H), 3.59–3.82 (m, 17 H), 3.80–3.92 (m, 5 H), 3.94–4.03 (m, 3 H), 4.03 (dd, *J* = 7.9, 3.9 Hz,

1 H), 4.05–4.13 (m, 2 H), 4.22–4.30 (m_c, 1 H), 4.93 (d, *J* = 2.6 Hz, 1 H), 5.43 (dd, *J* = 15.6, 7.7 Hz, 1 H), 5.68–5.77 (m_c, 1 H), 6.18 (s, 1 H), 6.33 (d, *J* = 4.0 Hz, 1 H), 6.95 (d, *J* = 4.0 Hz, 1 H), 7.22 (s, 1 H), 7.85 (s, 1 H).

Further signals were covered by signals of the solvent mixture.

^{13}C-NMR (151 MHz, $CDCl_3$/MeOD/D_2O, 1.6:1.0:0.2): *δ* (ppm) = 14.6, 15.3, 23.3, 25.3, 25.8, 25.8, 29.8, 30.0, 30.0, 30.1, 30.2, 30.3, 30.3, 30.4, 30.4, 30.5, 31.8, 32.6, 32.6, 32.9, 33.1, 35.3, 35.8, 36.9, 53.7, 60.5, 61.0, 62.1, 64.7, 69.2, 69.6, 70.2, 70.3, 70.7, 70.9, 71.0, 71.3, 72.1, 72.4, 72.7, 73.7, 74.1, 75.2, 75.7, 75.9, 78.8, 79.5, 80.6, 101.8, 103.4, 103.8, 117.5, 121.3, 124.9, 129.1, 129.5, 134.0, 135.5, 136.0, 145.3, 157.5, 161.2, 174.2, 176.7.

^{19}F-NMR (471 MHz, $CDCl_3$/MeOD/D_2O, 1.6:1.0:0.2): *δ* (ppm) = −143.1 (q, *J* = 33.1 Hz).

IR (ATR): $\tilde{\nu}$ (cm^{-1}) = 3351, 2919, 2851, 2160, 2028, 1735, 1647, 1605, 1537, 1464, 1367, 1253, 1078, 1031, 802.

MS (ESI): *m*/*z* (%) = 922.0 (100) $[M+2Na]^{2+}$, 1821.1 (29) $[M+Na]^+$. **HRMS** (ESI): *m*/*z* calculated for: $C_{91}H_{158}O_{23}BF_2N_7S$ $[M+Na]^+$: 1821.1096, found: 1821.1083.

_O_-(α-D-Galactopyranosyl)-(1→4)-(2-_O_-(46-(1-(3-BodipyFL-amidopropyl)-1_H_-1,2,3-triazol-4-yl)-9,12,15,18,21,24,27,30,33,36,39,42,45-tridecaoxa-6-thiahexatetracontan-1-yl)-β-D-galactopyranosyl)-(1→4)-β-D-glucopyranosyl-(1→1)-(2_S_,3_R_,4_E_,15′_Z_)-2-(15′-tetracosenamido)-4-octadecene-1,3-diol (5)

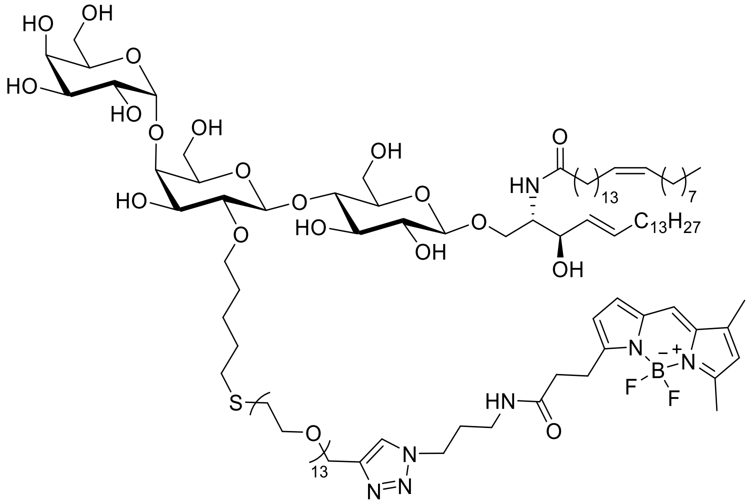

The cycloaddition of glycolipid **29** (7.70 mg, 4.17 µmol, 1.00 eq.) with dye **9** (1.87 mg, 5.00 µmol, 1.20 eq.) was performed according to general procedure **GP5** using copper powder (1.06 mg, 16.7 µmol, 4.00 eq.) and $CuSO_4{\cdot}5\,H_2O$ (0.1 M in H_2O, 8.34 µL, 0.834 µmol, 20 mol%) in water (100 µL) and *tert*-butanol (100 µL). The dialysis (2 d, 5 L of H_2O and 5 d, 12.5 L of H_2O) and the subsequent lyophilization were carried out twice to remove all traces of unreacted dye. Glycolipid **5** (7.60 mg, 3.42 µmol, 82 %) was isolated as a red powder.

$[\alpha]_D^{23}$ = +20.6 (*c* 0.82, $CHCl_3$/MeOH/H_2O, 1.6:1.0:0.2).

^{1}H-NMR (600 MHz, $CDCl_3$/MeOD/D_2O, 1.6:1.0:0.2): *δ* (ppm) = 0.78–0.95 (m, 9 H), 1.09–1.47 (m, 55 H), 1.50–1.68 (m, 8 H), 1.73–1.89 (m, 3 H), 1.99–2.06 (m, 7 H), 2.06–2.12 (m, 2 H), 2.14–2.20 (m, 2 H), 2.29 (s, 3 H), 2.56 (s, 3 H), 2.52–2.60 (m, 1 H), 2.62–2.68 (m_c, 2 H), 2.70–2.75 (m_c, 2 H), 2.80–2.86 (m, 1 H), 2.91–2.97 (m, 1 H), 3.04–3.11 (m, 1 H), 3.18–3.30 (m, 5 H), 3.36–3.43 (m, 2 H), 3.41–3.92 (m, 50 H), 3.96–4.01 (m, 3 H), 4.04–4.11 (m_c, 2 H), 4.22 (dd, *J* = 9.8, 3.9 Hz, 1 H), 4.24–4.29 (m, 1 H), 4.32 (d, *J* = 7.8 Hz, 1 H), 4.33–4.39 (m, 1 H), 4.95 (d, *J* = 3.0 Hz, 1 H), 5.31–5.40 (m, 2 H), 5.44 (dd, *J* = 15.3, 7.8 Hz, 1 H), 5.64–5.77 (m_c, 1 H), 6.19 (s, 1 H), 6.34 (d, *J* = 4.0 Hz, 1 H), 6.96 (d, *J* = 4.0 Hz, 1 H), 7.24 (s, 1 H), 7.99 (s, 1 H).

Further signals were covered by signals of the solvent mixture.

^{13}C-NMR (151 MHz, $CDCl_3$/MeOD/D_2O, 1.6:1.0:0.2): *δ* (ppm) = 15.2, 18.1, 18.8, 26.8, 28.8, 29.3, 30.2, 31.3, 33.4, 33.5, 33.5, 33.6, 33.7, 33.7, 33.7, 33.8, 33.8, 33.9, 34.0, 35.4, 36.1, 36.1, 36.4, 36.6, 39.3, 40.4, 40.6, 57.3, 60.8, 64.0, 64.5, 65.2, 65.8, 67.8, 68.4, 72.9, 73.2, 73.8, 73.9, 74.3, 74.4, 74.8, 75.7, 75.9, 76.7, 77.3, 77.5, 77.6, 78.8, 79.1, 79.4, 82.6, 83.3, 84.1, 105.5, 107.0, 107.5, 121.0, 124.8, 128.4, 132.6, 133.5, 134.1, 137.6, 138.8, 139.5, 148.8, 161.1, 164.7, 177.7, 178.9.

^{19}F-NMR (471 MHz, $CDCl_3$/MeOD/D_2O, 1.6:1.0:0.2): *δ* (ppm) = −143.1 (q, *J* = 32.9 Hz).

IR (ATR): $\tilde{\nu}$ (cm^{-1}) = 3356, 2921, 2853, 2160, 2029, 1975, 1727, 1648, 1605, 1535, 1411, 1251, 1081, 1033, 804.

MS (ESI): *m*/*z* (%) = 1133.2 (100) $[M+2Na]^{2+}$, 2243.4 (6) $[M+Na]^+$. **HRMS** (ESI): *m*/*z* calculated for: $C_{111}H_{196}O_{32}SBF_2N_7$ $[M+Na]^+$: 2243.3616, found: 2243.3612.

O-(α-D-Galactopyranosyl)-(1→4)-(2-O-(16-(1-(3-BodipyFL-amidopropyl)-1H-1,2,3-triazol-4-yl)-9,12,15-trioxa-6-thiahexandecan-1-yl)-β-D-galactopyranosyl)-(1→4)-β-D-glucopyranosyl-(1→1)-(2S,3R,4E,15′Z)-2-(15′-tetracosenamido)-4-octadecene-1,3-diol (6)

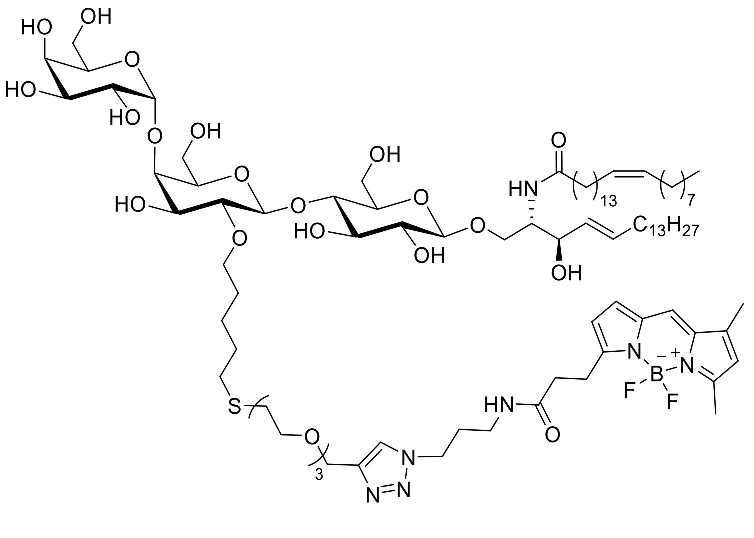

The cycloaddition of glycolipid **30** (10.6 mg, 7.53 µmol, 1.00 eq.) with dye **9** (3.10 mg, 8.28 µmol, 1.10 eq.) was performed according to general procedure **GP5** using copper powder (1.91 mg, 30.1 µmol, 4.00 eq.) and $CuSO_4 \cdot 5\,H_2O$ (0.1 M in H_2O, 15.1 µL, 1.51 µmol, 20 mol%) in water (180 µL) and *tert*-butanol (180 µL). The dialysis (5 d, 12.5 L H_2O) and the subsequent lyophilization were carried out twice to remove all traces of unreacted dye. Glycolipid **6** (7.50 mg, 4.21 µmol, 56 %) was isolated as a red powder.

$[\alpha]_D^{27}$ = +29.4 (*c* 0.60, $CHCl_3$/MeOH/H_2O, 1.6:1.0:0.2).

^{1}H-NMR (600 MHz, $CDCl_3$/MeOD/D_2O, 1.6:1.0:0.2): *δ* (ppm) = 0.78–0.98 (m, 9 H), 1.11–1.47 (m, 54 H), 1.49–1.67 (m, 7 H), 2.05–2.11 (m, 6 H), 2.05–2.11 (m_c, 2 H), 2.14–2.20 (m_c, 2 H), 2.29 (s, 3 H), 2.55 (s, 3 H), 2.56–2.62 (m_c, 2 H), 2.65 (t, *J* = 7.6 Hz, 2 H), 2.74 (t, *J* = 6.7 Hz, 2 H), 3.18–3.29 (m, 4 H), 3.39 (td, *J* = 5.5, 2.8 Hz, 1 H), 3.52–3.59 (m, 2 H), 3.59–3.79 (m, 14 H), 3.78–3.92 (m, 6 H), 3.94–4.01 (m, 3 H), 4.03–4.10 (m_c, 1 H), 4.20 (dd, *J* = 10.0, 4.0 Hz, 1 H), 4.27 (t, *J* = 6.1 Hz, 1 H), 4.93 (d, *J* = 2.8 Hz, 1 H), 5.30–5.40 (m_c, 2 H), 5.44 (dd, *J* = 15.3, 7.8 Hz, 1 H), 5.66–5.74 (m_c, 1 H), 6.19 (s, 1 H), 6.34 (d, *J* = 4.0 Hz, 1 H), 6.96 (d, *J* = 4.0 Hz, 1 H), 7.22 (s, 1 H), 7.89 (s, 1 H).

Further signals were covered by signals of the solvent mixture.

^{13}C-NMR (151 MHz, $CDCl_3$/MeOD/D_2O, 1.6:1.0:0.2): *δ* (ppm) = 11.7, 14.6, 15.3, 23.3, 23.3, 25.3, 25.7, 26.7, 27.8, 27.8, 29.9, 29.9, 30.0, 30.1, 30.2, 30.3, 30.3, 30.3, 30.3, 30.4, 30.4, 32.5, 32.6, 33.1, 35.8, 36.8, 37.1, 53.8, 60.4, 61.0, 62.1, 64.6, 69.4, 69.6, 70.2, 70.3, 70.7, 70.9, 70.9, 71.0, 72.1, 72.4, 73.7, 73.9, 74.1, 75.2, 75.6, 75.8, 78.9, 79.6, 80.5, 101.8, 103.4, 103.8, 117.5, 121.3, 124.8, 129.0, 129.9, 130.5, 134.0, 135.4, 136.0, 145.3, 157.5, 161.2, 174.2, 175.3.

^{19}F-NMR (471 MHz, $CDCl_3$/MeOD/D_2O, 1.6:1.0:0.2): *δ* (ppm) = −143.1 (q, *J* = 32.9 Hz).

IR (ATR): $\tilde{\nu}$ (cm^{-1}) = 3358, 3340, 2920, 2852, 2160, 2029, 1976, 1738, 1646, 1606, 1540, 1462, 1363, 1250, 1078, 1054, 803.

MS (ESI): *m*/*z* (%) = 913.0 (100) $[M+2Na]^{2+}$, 1803.1 (22) $[M+Na]^+$. **HRMS** (ESI): *m*/*z* calculated for: $C_{91}H_{156}O_{22}BF_2N_7S$ $[M+Na]^+$: 1803.0992, found: 1803.0999.

***O*-(α-D-Galactopyranosyl)-(1→4)-(2-*O*-(46-(1-(3-BodipyFL-amidopropyl)-1*H*-1,2,3-triazol-4-yl)-9,12,15,18,21,24,27,30,33,36,39,42,45-tridecaoxa-6-thiahexatetracontan-1-yl)-β-D-galactopyranosyl)-(1→4)-β-D-glucopyranosyl-(1→1)-(2*S*,3*R*,4*E*,2′*R*,15′*E*)-2-(2′-hydroxy-15′-tetracosenamido)-4-octadecene-1,3-diol (7)**

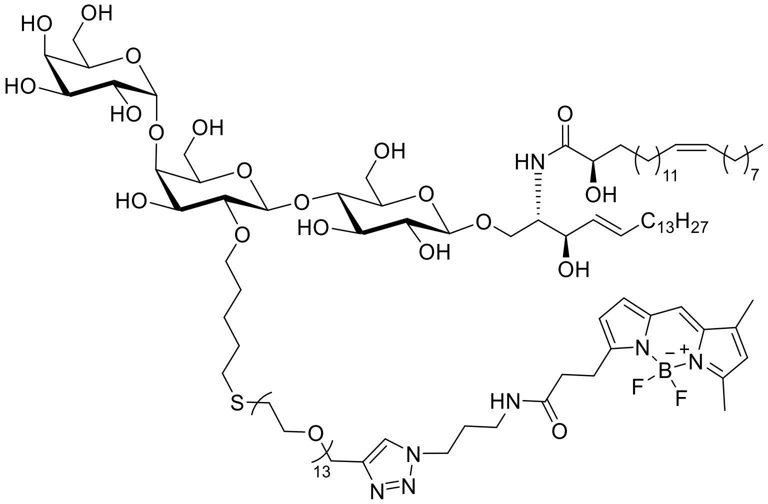

The cycloaddition of glycolipid **31** (5.90 mg, 3.17 μmol, 1.00 eq.) with dye **9** (1.42 mg, 3.80 μmol, 1.20 eq.) was performed according to general procedure **GP5** with copper powder (0.806 mg, 12.7 μmol, 4.00 eq.) and $CuSO_4 \cdot 5\ H_2O$ (0.1 M in H_2O, 6.34 μL, 0.634 μmol, 20.0 mol%) in water (80.0 μL) and *tert*-butanol (80.0 μL). The dialysis (5 d, 12.5 L H_2O) and the subsequent lyophilization were carried out twice to remove all traces of unreacted dye. Glycolipid **7** (6.60 mg, 2.95 μmol, 93 %) was isolated as a red powder.

$[\alpha]_D^{23}$ = +30.6 (*c* 0.66, $CHCl_3$/MeOH/H_2O, 1.6:1.0:0.2).

^{1}H-NMR (500 MHz, $CDCl_3$/MeOD/D_2O, 1.6:1.0:0.2): *δ* (ppm) = 0.73–1.02 (m, 12 H), 1.22–1.35 (m, 64 H), 1.55–1.66 (m, 6 H), 1.91–2.11 (m, 8 H), 2.14–2.19 (m, 1 H), 2.21 (t, *J* = 7.7 Hz, 1 H), 2.29 (s, 3 H), 2.56 (s, 3 H), 2.56–2.60 (m, 1 H), 2.65 (t, *J* = 7.6 Hz, 2 H), 2.72 (t, *J* = 6.9 Hz, 2 H), 2.79–2.87 (m, 1 H), 2.90–2.99 (m, 1 H), 3.01–3.11 (m, 1 H), 3.18–3.29 (m, 4 H), 3.48–3.92 (m, 50 H), 3.94–4.07 (m, 4 H), 4.04–4.14 (m, 2 H), 4.93 (s, 1 H), 5.25–5.38 (m, 2 H), 5.37–5.49 (m_c, 1 H), 5.67–5.78 (m, 1 H), 6.19 (s, 1 H), 6.33 (d, *J* = 4.1 Hz, 1 H), 6.95 (d, *J* = 4.1 Hz, 1 H), 7.22 (s, 1 H), 7.86 (s, 1 H).

Further signals were covered by signals of the solvent mixture.

^{13}C-NMR (126 MHz, $CDCl_3$/MeOD/D_2O, 1.6:1.0:0.2): *δ* (ppm) = 11.7, 14.5, 15.3, 23.2, 25.2, 25.7, 25.8, 26.3, 27.8, 29.8, 29.9, 30.0, 30.1, 30.1, 30.2, 30.3, 30.4, 30.4, 31.8, 32.5, 32.5, 32.9, 33.0, 35.2, 35.8, 36.3, 36.8, 52.5, 53.7, 60.5, 61.0, 62.2, 64.6, 69.2, 69.6, 70.1, 70.3, 70.6, 70.8, 71.2, 72.1, 72.3, 72.3, 72.7, 73.7, 74.0, 75.2, 75.6, 75.8, 78.8, 79.3, 79.5, 80.5, 101.8, 103.4, 103.8, 117.5, 121.3, 124.8, 129.0, 129.5, 130.3, 130.4, 130.5, 130.6, 134.0, 135.4, 135.9, 145.2, 157.5, 161.2, 174.1, 176.6.

^{19}F-NMR (471 MHz, $CDCl_3$/MeOD/D_2O, 1.6:1.0:0.2): *δ* (ppm) = −143.1 (q, *J* = 33.1 Hz).

IR (ATR): $\tilde{\nu}$ (cm^{-1}) = 3317, 2921, 2853, 2161, 2026, 1975, 1727, 1629, 1606, 1459, 1255, 1074, 801, 666.

MS (ESI): *m/z* (%) = 768.8 (26) $[M+3Na]^{3+}$, 1141.7 (100) $[M+2Na]^{2+}$, 2260.4 (3) $[M+Na]^{+}$. **HRMS** (ESI): *m/z* calculated for: $C_{111}H_{196}BF_2N_7O_{33}S$ $[M+Na]^{+}$: 2260.3582, found: 2260.3580.

***O*-(α-D-Galactopyranosyl)-(1→4)-(2-*O*-(16-(1-(3-BodipyFL-amidopropyl)-1*H*-1,2,3-triazol-4-yl)-9,12,15-trioxa-6-thiahexandecan-1-yl)-β-D-galactopyranosyl)-(1→4)-β-D-glucopyranosyl-(1→1)-(2*S*,3*R*,4*E*,2′*R*,15′*E*)-2-(2′-hydroxy-15′-tetracosenamido)-4-octadecene-1,3-diol (8)**

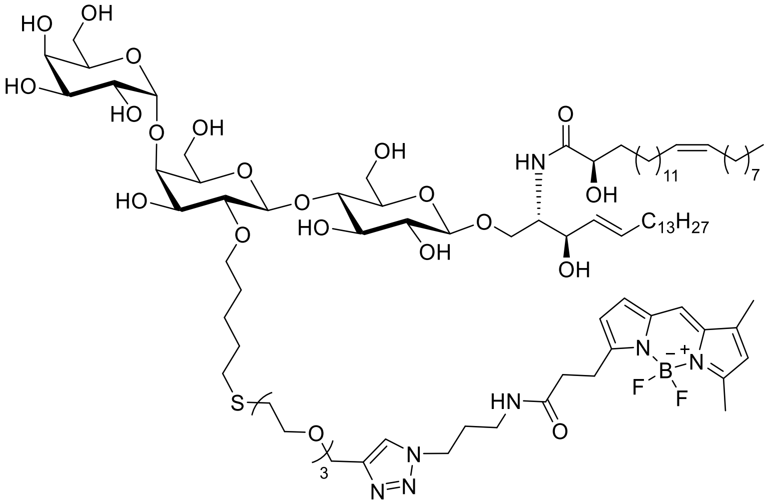

The cycloaddition of glycolipid **32** (8.30 mg, 5.83 µmol, 1.00 eq.) with dye **9** (2.62 mg, 7.00 µmol, 1.20 eq.) was performed according to general procedure **GP5** using copper powder (1.48 mg, 23.3 µmol, 4.00 eq.) and $CuSO_4{\cdot}5\ H_2O$ (0.1 M in H_2O, 11.7 µL, 1.17 µmol, 20 mol%) in water (140 µL) and *tert*-butanol (140 µL). The dialysis (5 d, 12.5 L H_2O) and the subsequent lyophilization were carried out twice to remove all traces of unreacted dye. Glycolipid **8** (7.00 mg, 3.90 µmol, 67 %) was isolated as a red powder.

$[\alpha]_{D}^{23}$ = +40.1 (*c* 0.70, $CHCl_3$/MeOH/H_2O, 1.6:1.0:0.2).

¹H-NMR (600 MHz, $CDCl_3$/MeOD/D_2O, 1.6:1.0:0.2): *δ* (ppm) = 0.71–1.04 (m, 8 H), 1.08–1.46 (m, 56 H), 1.51–1.67 (m, 6 H), 1.67–1.77 (m, 2 H), 1.99–2.06 (m, 6 H), 2.04–2.12 (m, 2 H), 2.29 (s, 3 H), 2.55 (s, 3 H), 2.58–2.63 (m_c, 1 H), 2.65 (t, *J* = 7.6 Hz, 1 H), 2.72–2.78 (m_c, 1 H), 2.90–2.97 (m, 1 H), 3.06 (dd, *J* = 14.5, 7.7 Hz, 1 H), 3.15–3.30 (m, 4 H), 3.37–3.45 (m, 2 H), 3.52–3.58 (m, 2 H), 3.57–3.78 (m, 14 H), 3.81–3.93 (m, 5 H), 3.93–4.00 (m, 3 H), 4.03 (dd, *J* = 8.0, 3.7 Hz, 1 H), 4.06–4.14 (m, 2 H), 4.24–4.29 (m, 1 H), 4.93 (d, *J* = 2.7 Hz, 1 H), 5.31–5.40 (m, 2 H), 5.43 (dd, *J* = 15.3, 7.6 Hz, 1 H), 5.65–5.77 (m_c, 1 H), 6.19 (s, 1 H), 6.34 (d, *J* = 4.0 Hz, 1 H), 6.96 (d, *J* = 4.0 Hz, 1 H), 7.22 (s, 1 H), 7.91 (s, 1 H).

Further signals were covered by signals of the solvent mixture.

^{13}C-NMR (151 MHz, $CDCl_3$/MeOD/D_2O, 1.6:1.0:0.2): δ (ppm) = 11.7, 14.5, 15.2, 23.2, 23.3, 25.2, 25.7, 25.8, 27.8, 27.8, 29.8, 29.9, 29.9, 29.9, 30.0, 30.1, 30.1, 30.2, 30.3, 30.3, 30.4, 30.4, 32.5, 32.5, 33.0, 35.2, 35.8, 36.8, 52.6, 53.7, 60.4, 61.0, 62.1, 64.2, 69.2, 69.6, 70.2, 70.3, 70.7, 70.8, 70.9, 70.9, 71.0, 72.1, 72.3, 72.7, 73.7, 74.0, 75.2, 75.6, 75.8, 78.8, 79.5, 80.5, 101.8, 103.4, 103.8, 117.5, 121.3, 124.8, 129.0, 129.5, 130.5, 134.0, 135.4, 135.9, 145.2, 157.4, 161.2, 174.2, 176.6.

^{19}F-NMR (471 MHz, $CDCl_3$/MeOD/D_2O, 1.6:1.0:0.2): δ (ppm) = −143.1 (q, J = 33.1 Hz).

IR (ATR): $\tilde{\nu}$ (cm^{-1}) = 3350, 2920, 2852, 1738, 1648, 1605, 1536, 1360, 1252, 1077, 1034, 802.

MS (ESI): m/z (%) = 921.0 (100) $[M+2Na]^{2+}$, 1819.1 (16) $[M+Na]^{+}$. **HRMS** (ESI): m/z calculated for: $C_{91}H_{156}O_{23}BF_2N_7S$ $[M+Na]^{+}$: 1819.0939, found: 1819.0944.

Synthesis of Azidosphingosine 10

Protected azidosphingosine **10** was synthesized via a combination of different literature known transformations.[6] Starting point of this sequence shown below was Garner's aldehyde (**48**),[7] which was synthesized from enantiomerically pure amino acid L-serine.

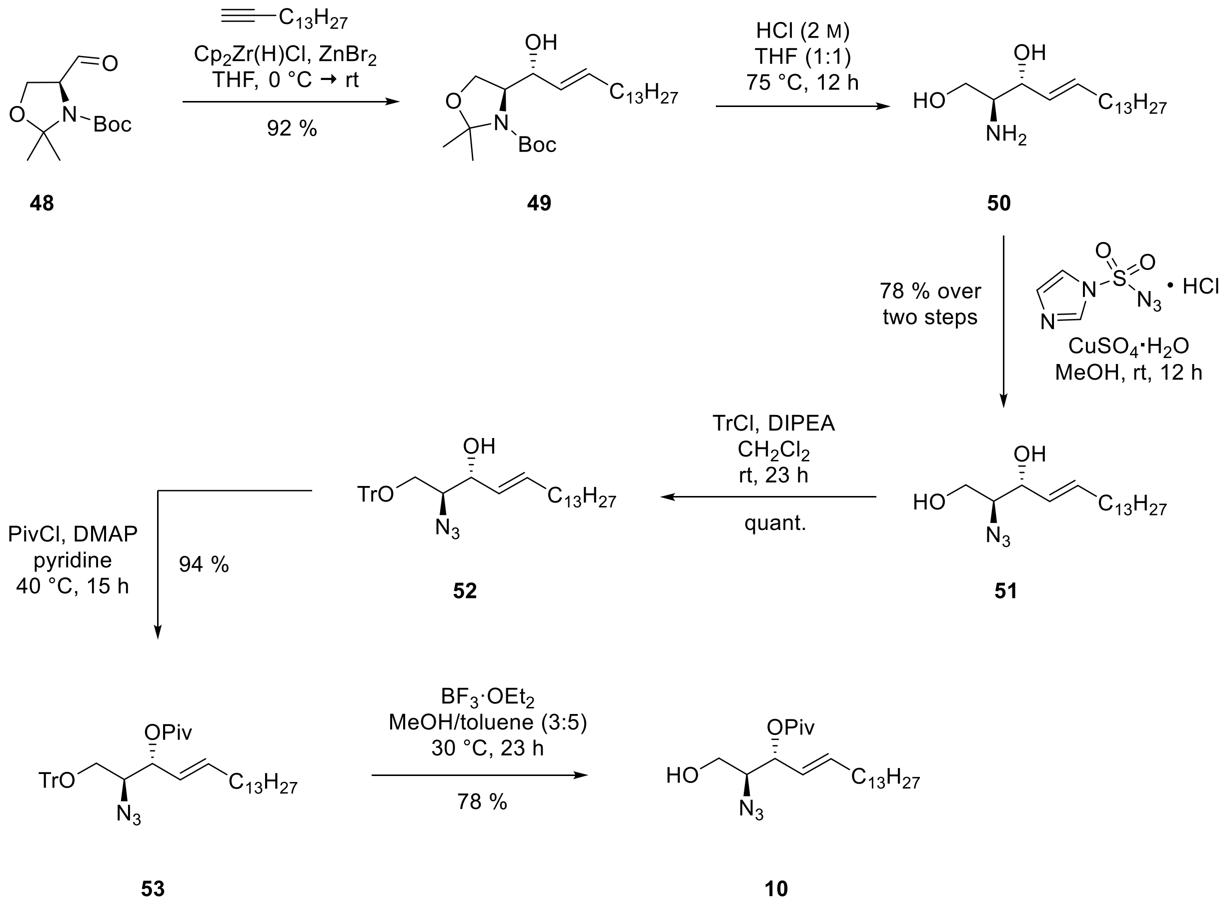

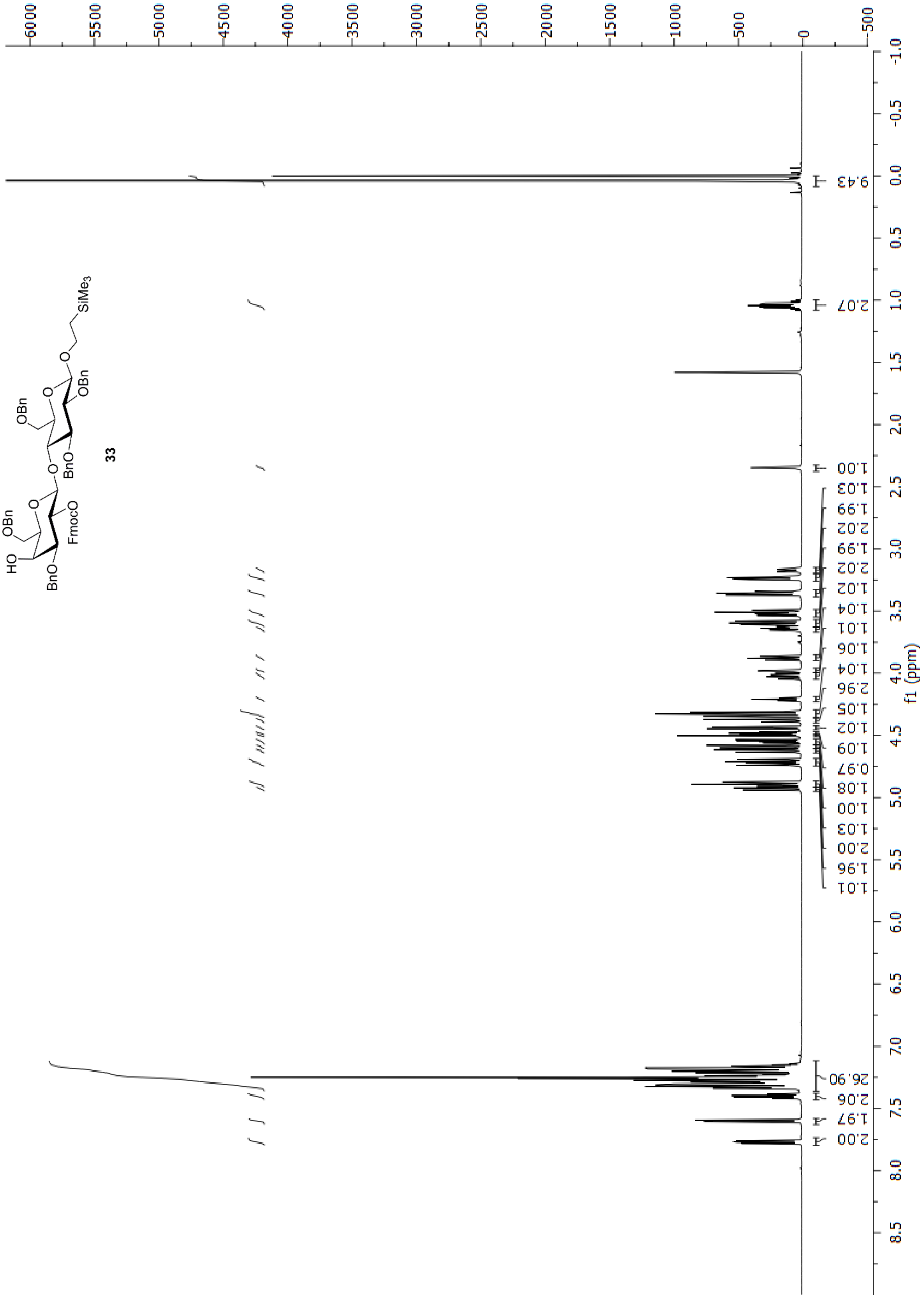
SiMe3
OBn
OBn
HO
BnO
FmocO
BnO
33
f1 (ppm)

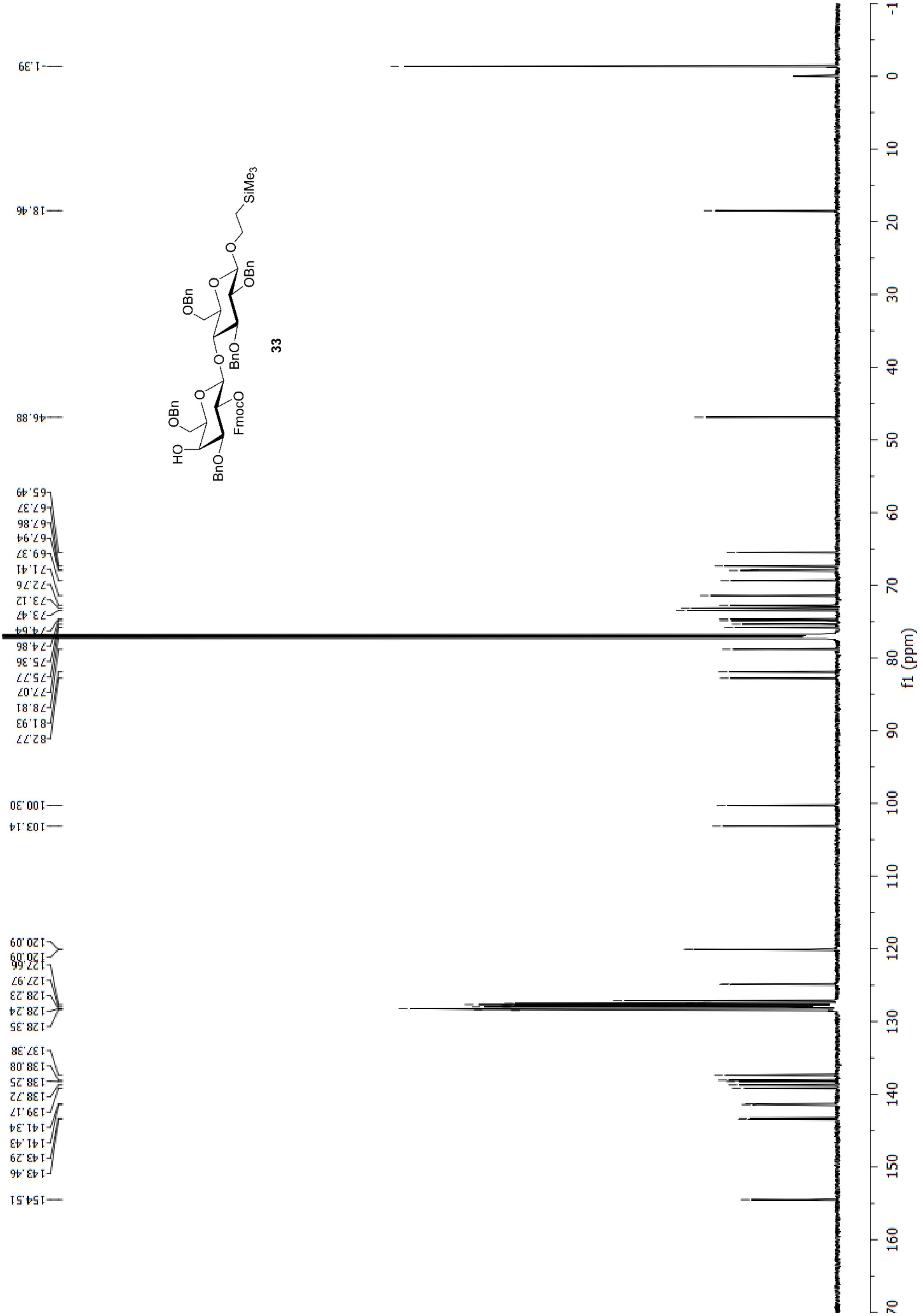
-1.39
18.46
46.88
65.49
67.37
67.86
67.94
69.37
71.41
72.76
73.12
73.47
74.64
74.86
75.36
75.77
77.07
78.81
81.93
82.77
100.30
103.14
120.09
120.09
127.66
127.97
128.23
128.24
128.35
137.38
138.08
138.25
138.72
139.17
141.34
141.43
143.29
143.46
154.51
SiMe3
OBn
BnO
HO
FmocO
33
f1 (ppm)

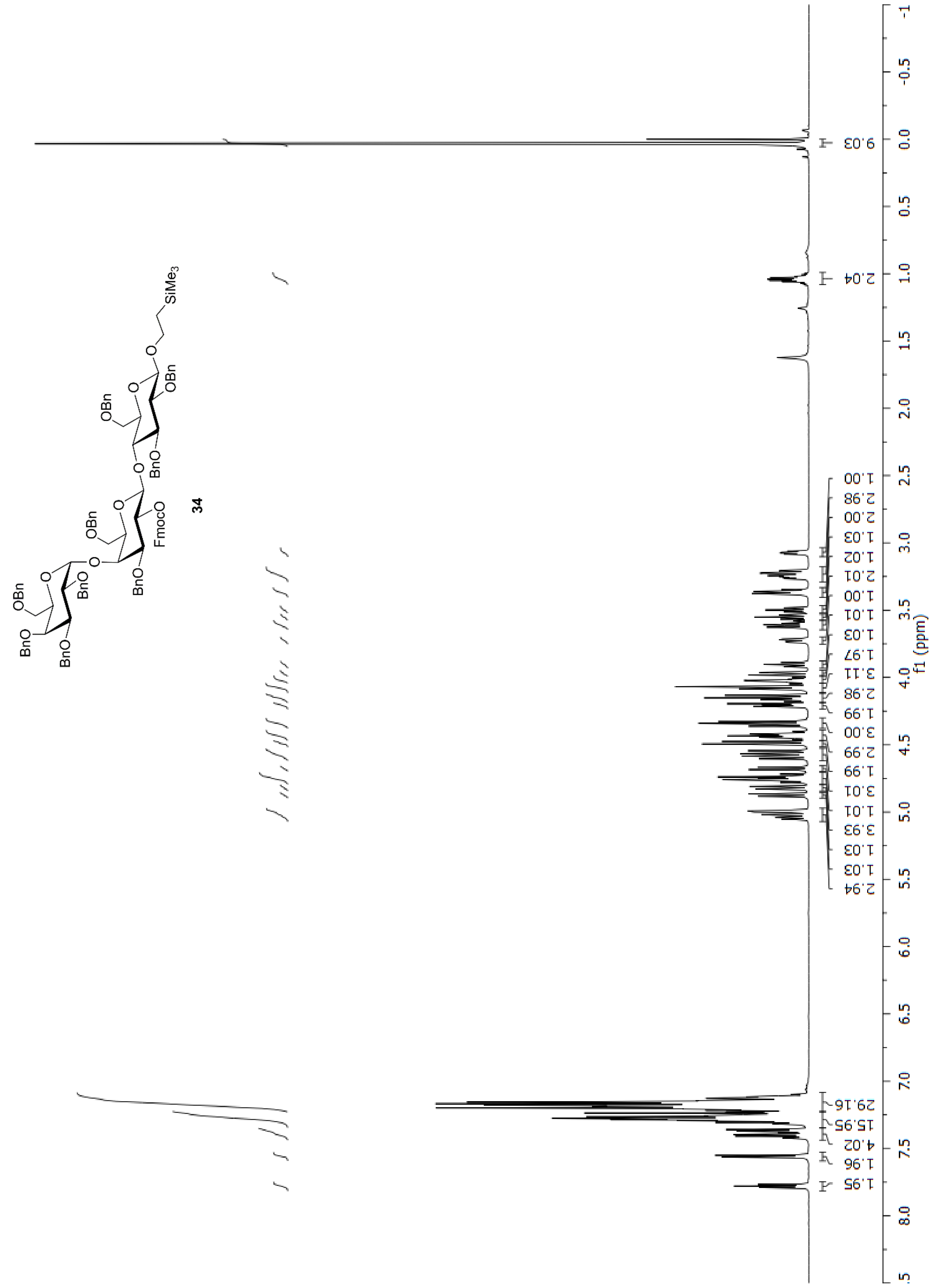

34

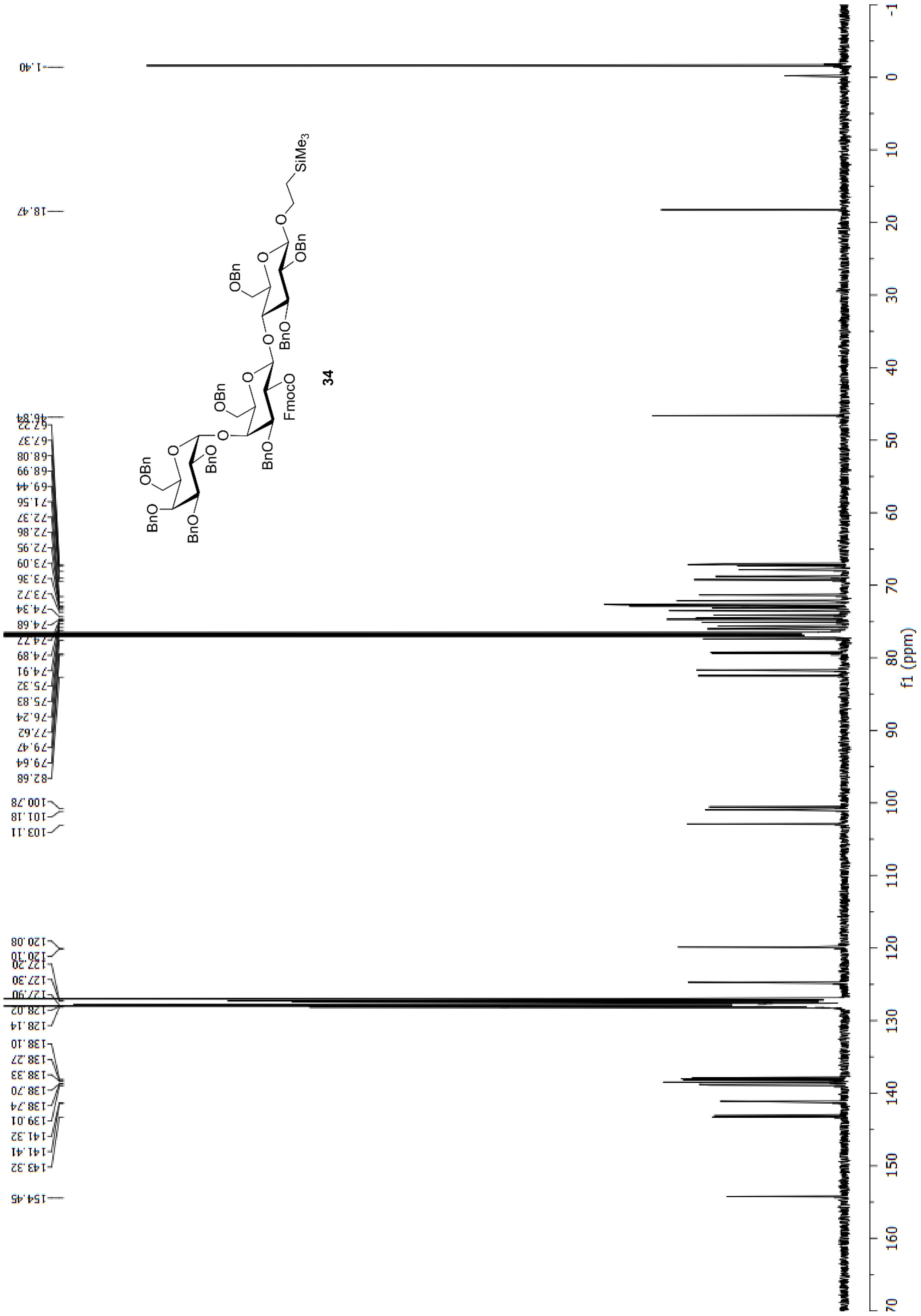
34
f1 (ppm)

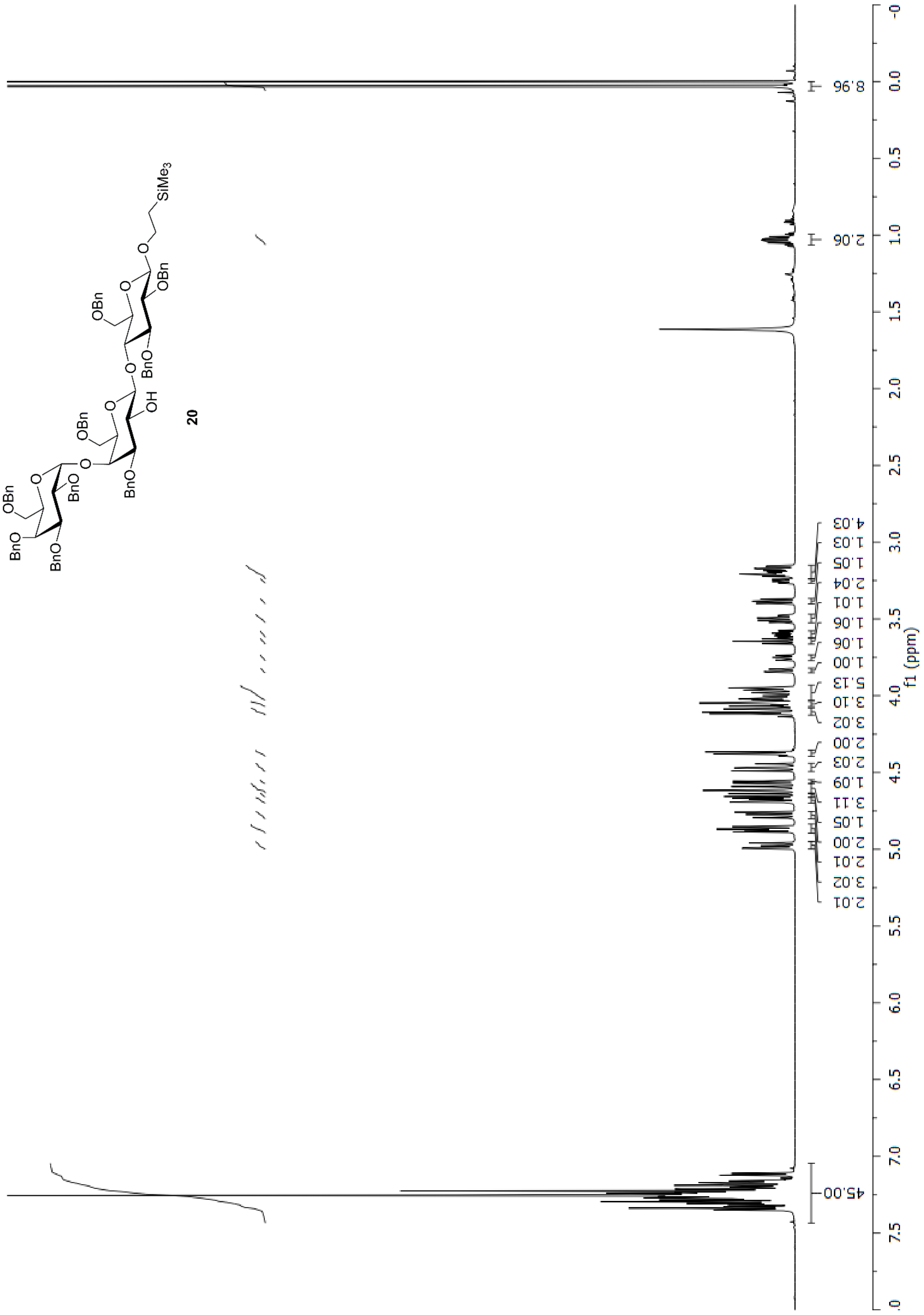
SiMe3
OBn
BnO
OH
20
f1 (ppm)

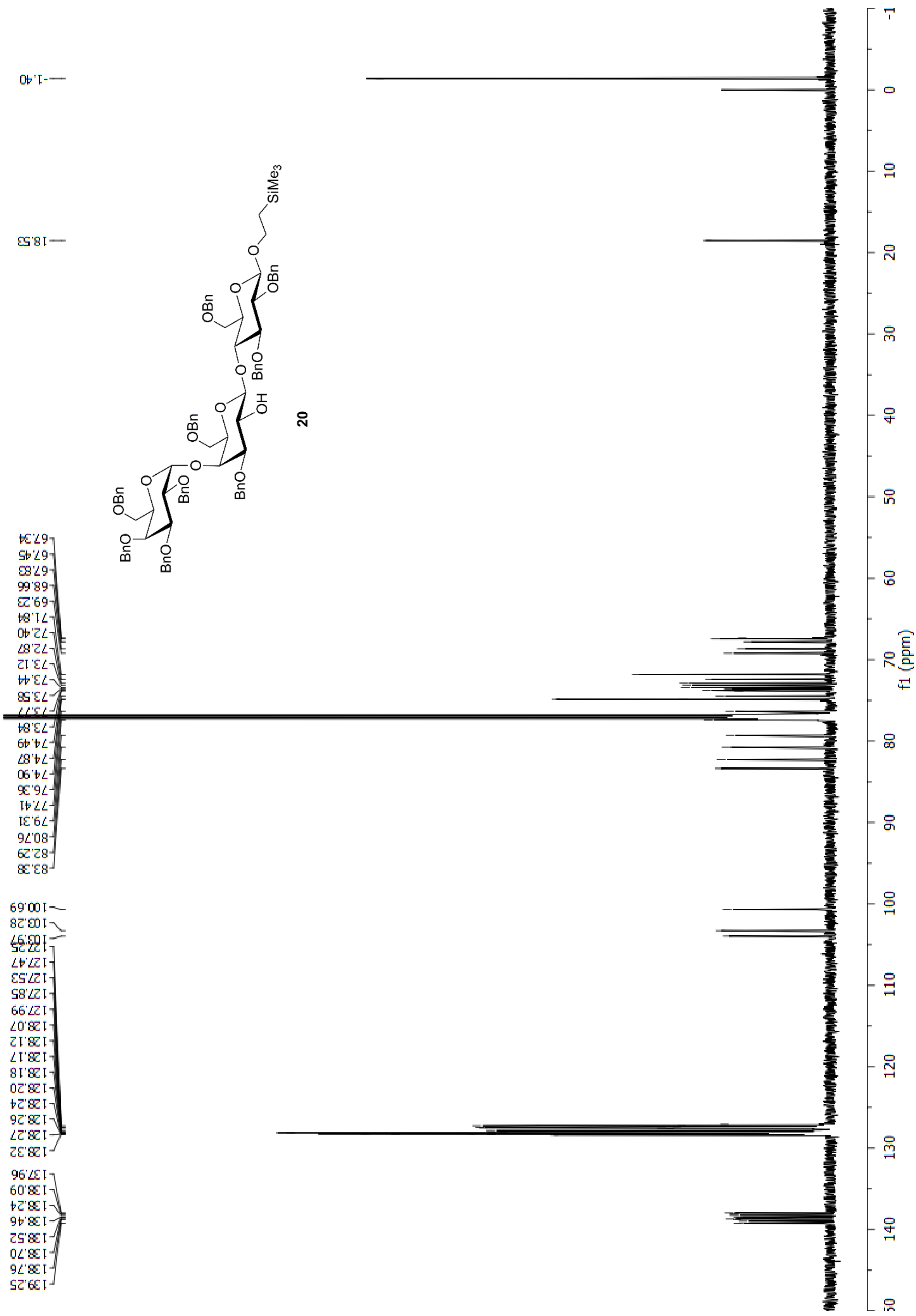
-1.40
18.53
67.34
67.45
67.83
68.66
69.23
71.84
72.40
72.87
73.12
73.44
73.58
73.77
73.84
74.49
74.87
74.90
76.36
77.41
79.31
80.76
82.29
83.38
100.69
103.28
103.97
127.35
127.47
127.53
127.85
127.99
128.07
128.12
128.17
128.18
128.20
128.24
128.26
128.27
128.32
137.96
138.09
138.24
138.46
138.52
138.70
138.76
139.25
BnO
BnO
OBn
BnO
OBn
BnO
OH
BnO
OBn
OBn
SiMe3
20
f1 (ppm)

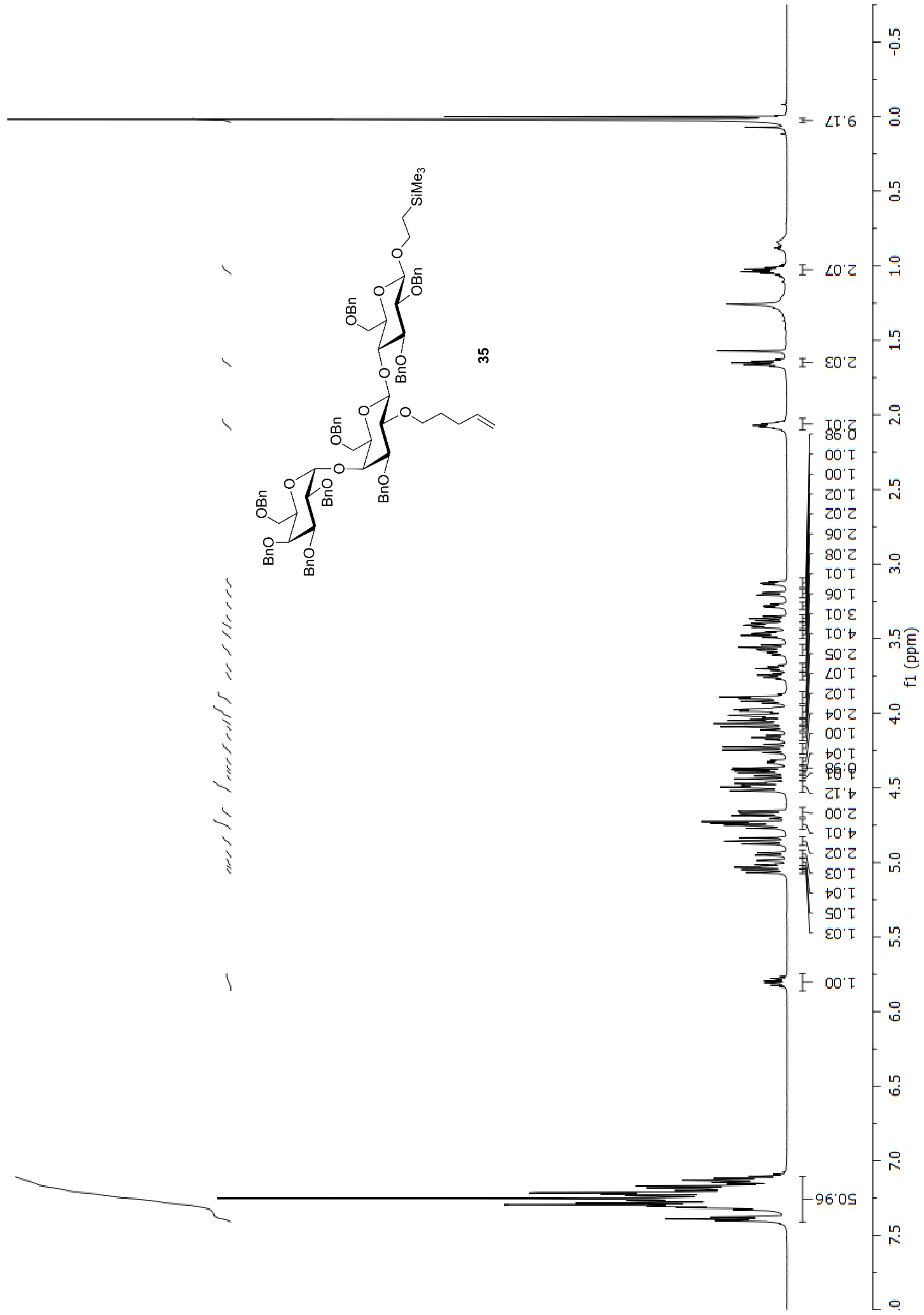
35
SiMe3
OBn
BnO
f1 (ppm)

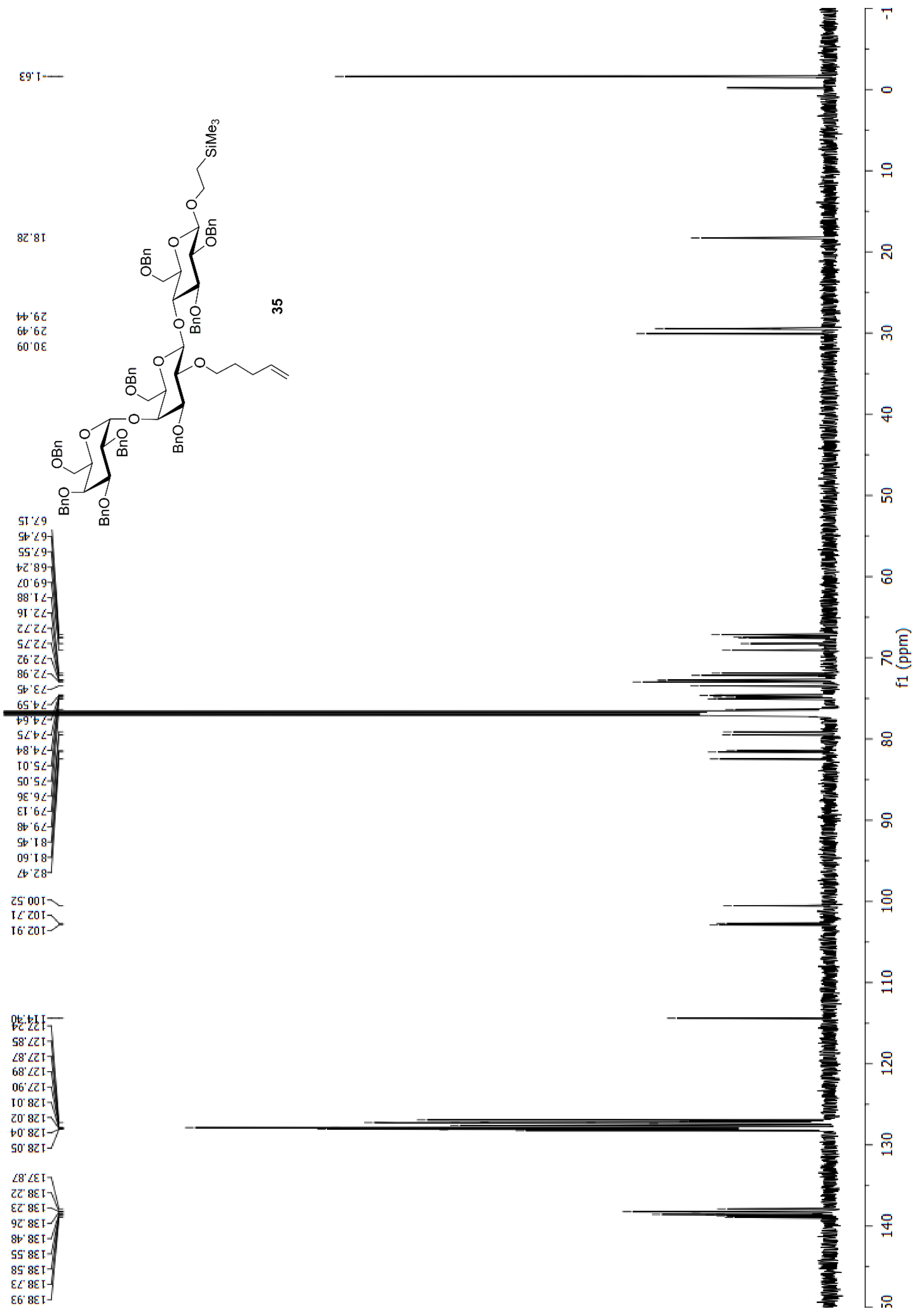
-1.63
18.28
29.44
29.49
30.09
67.15
67.45
67.55
68.24
69.07
71.88
72.16
72.72
72.75
72.92
72.98
73.45
74.59
74.64
74.75
74.84
75.01
75.05
76.36
79.13
79.48
81.45
81.60
82.47
100.52
102.71
102.91
114.90
127.24
127.85
127.87
127.89
127.90
128.01
128.02
128.04
128.05
137.87
138.22
138.23
138.26
138.48
138.55
138.58
138.73
138.93
SiMe3
OBn
BnO
35
f1 (ppm)

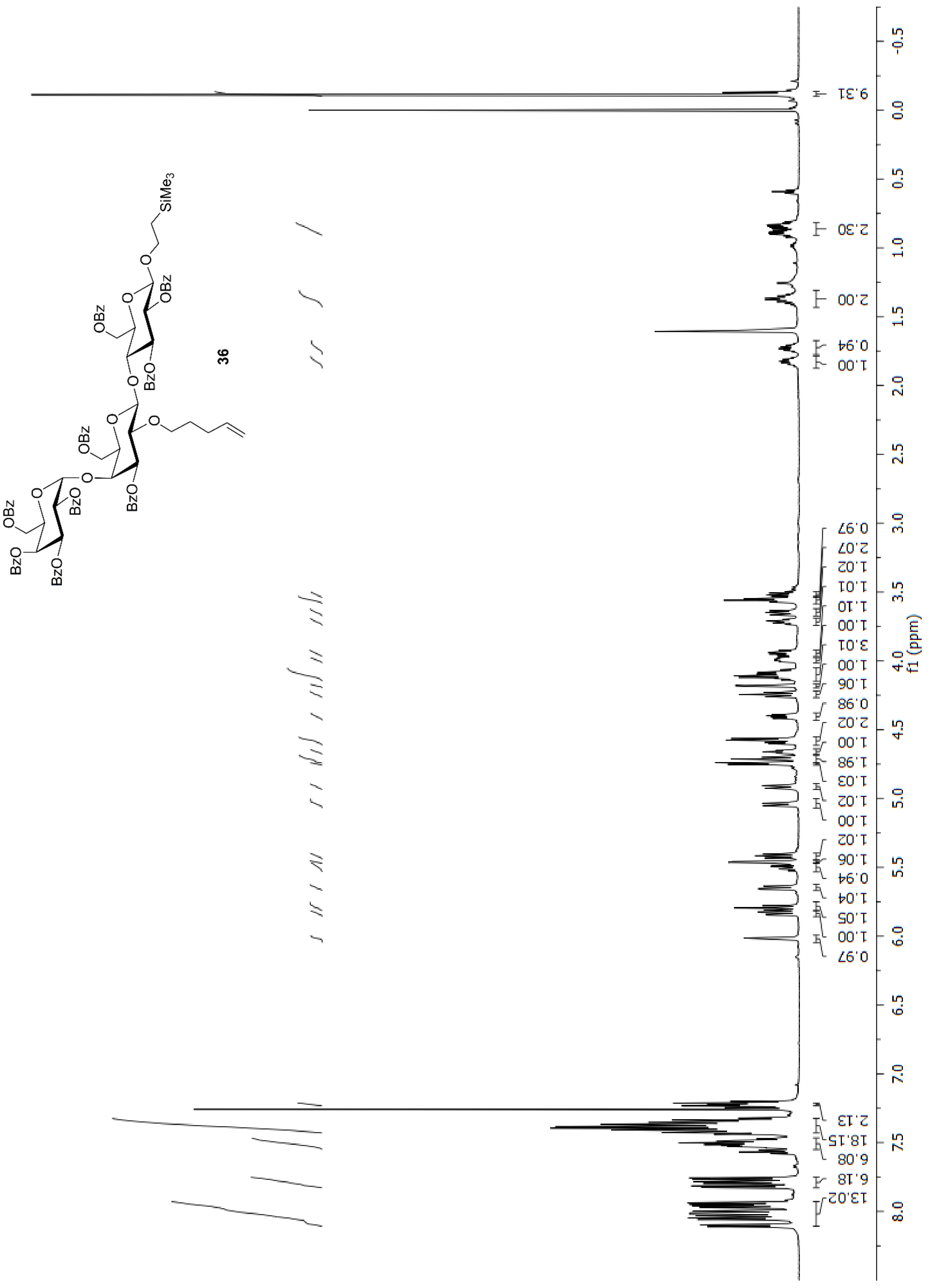
36
f1 (ppm)

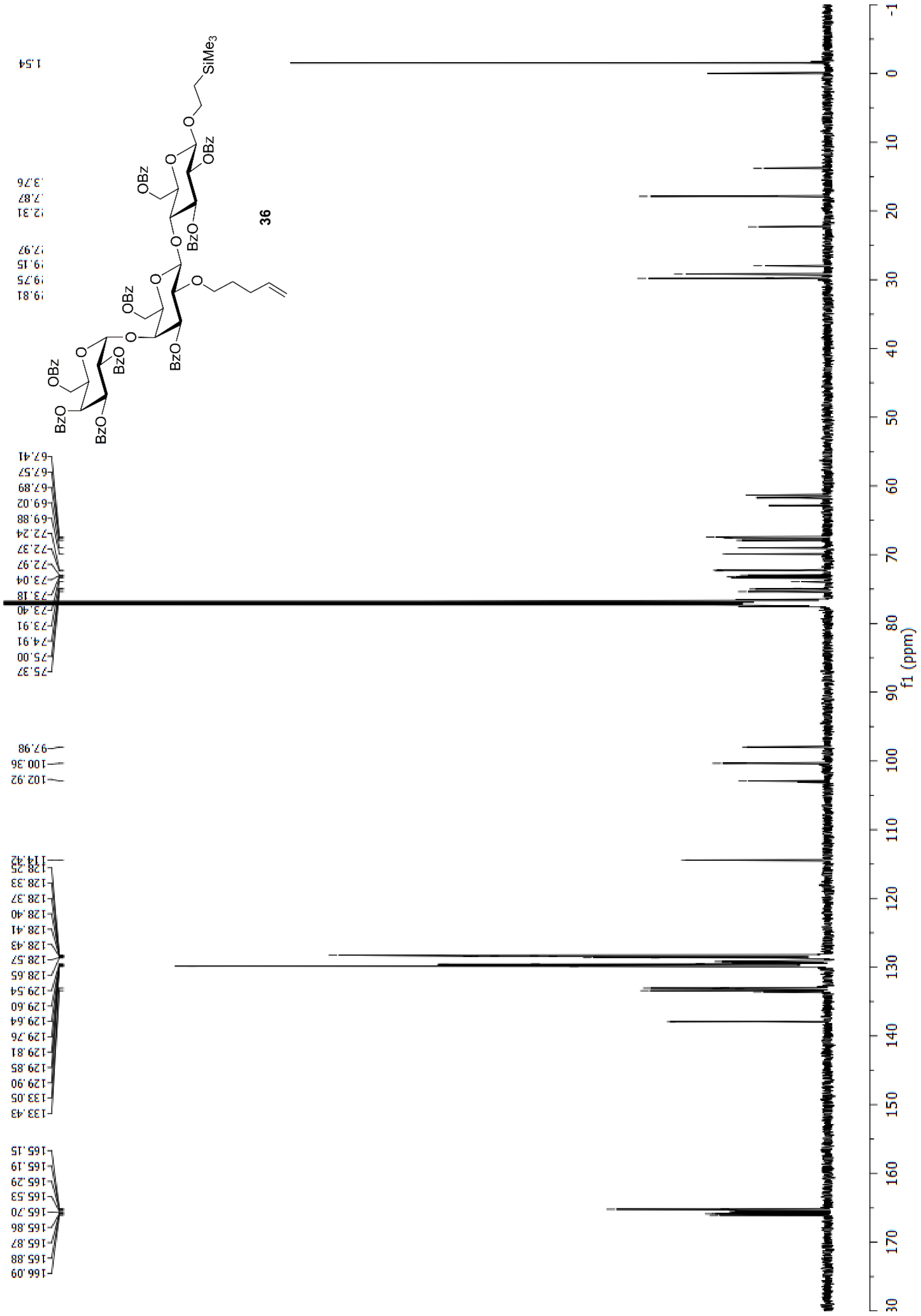
SiMe3
OBz
BzO
1.54
67.41
67.57
67.89
69.02
69.88
72.24
72.37
72.97
73.04
73.18
73.40
73.91
74.91
75.00
75.37
97.98
100.36
102.92
114.42
128.25
128.33
128.37
128.40
128.41
128.43
128.57
128.65
129.54
129.60
129.64
129.76
129.81
129.85
129.90
133.05
133.43
165.15
165.19
165.29
165.53
165.70
165.86
165.87
165.88
166.09
f1 (ppm)

36

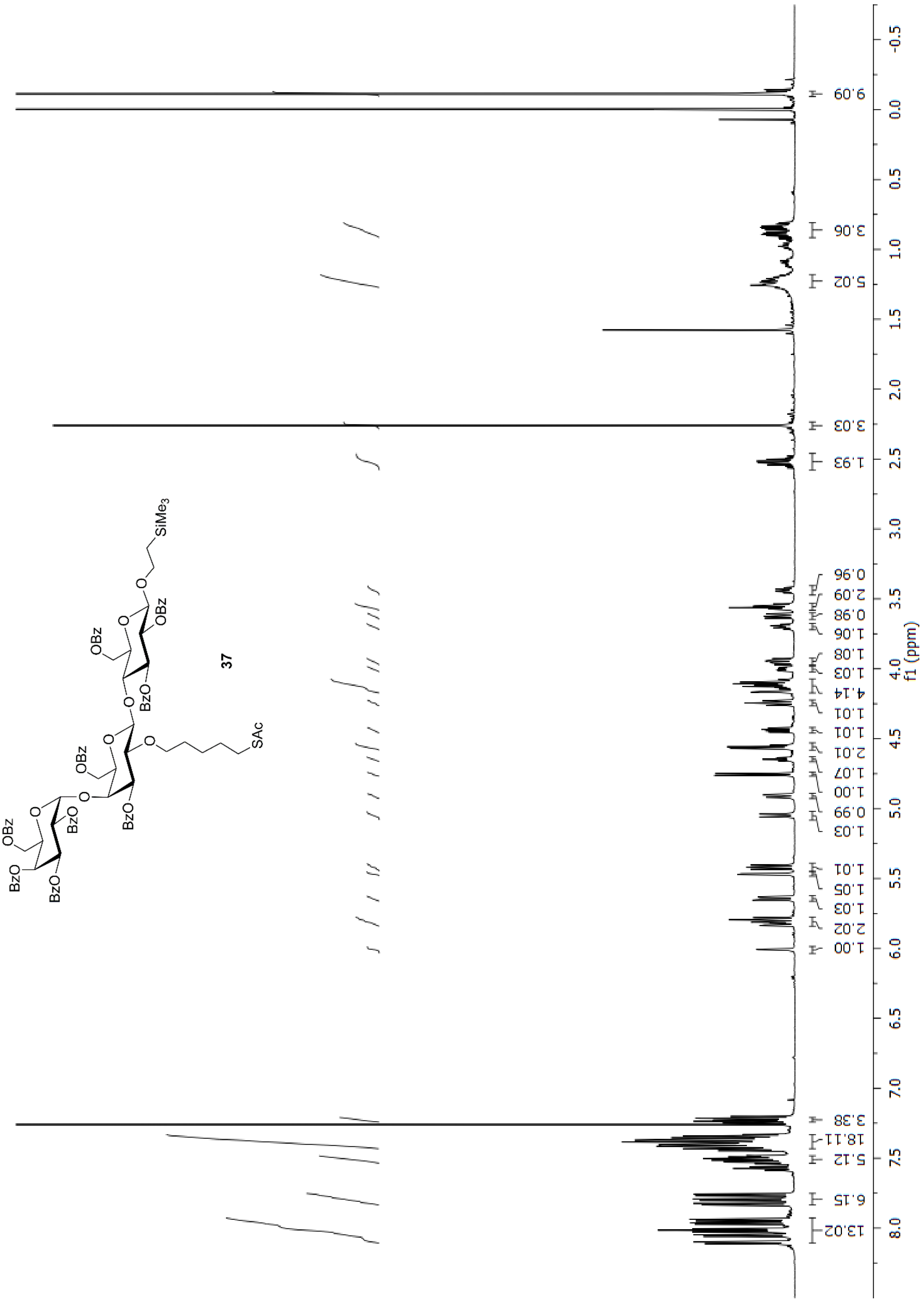
SiMe3
OBz
BzO
SAc
f1 (ppm)
9.09
3.06
5.02
3.03
1.93
0.96
2.09
0.98
1.06
1.08
1.03
4.14
1.01
1.01
2.01
1.07
1.00
0.99
1.03
1.01
1.05
1.03
2.02
1.00
3.38
18.11
5.12
6.15
13.02

37

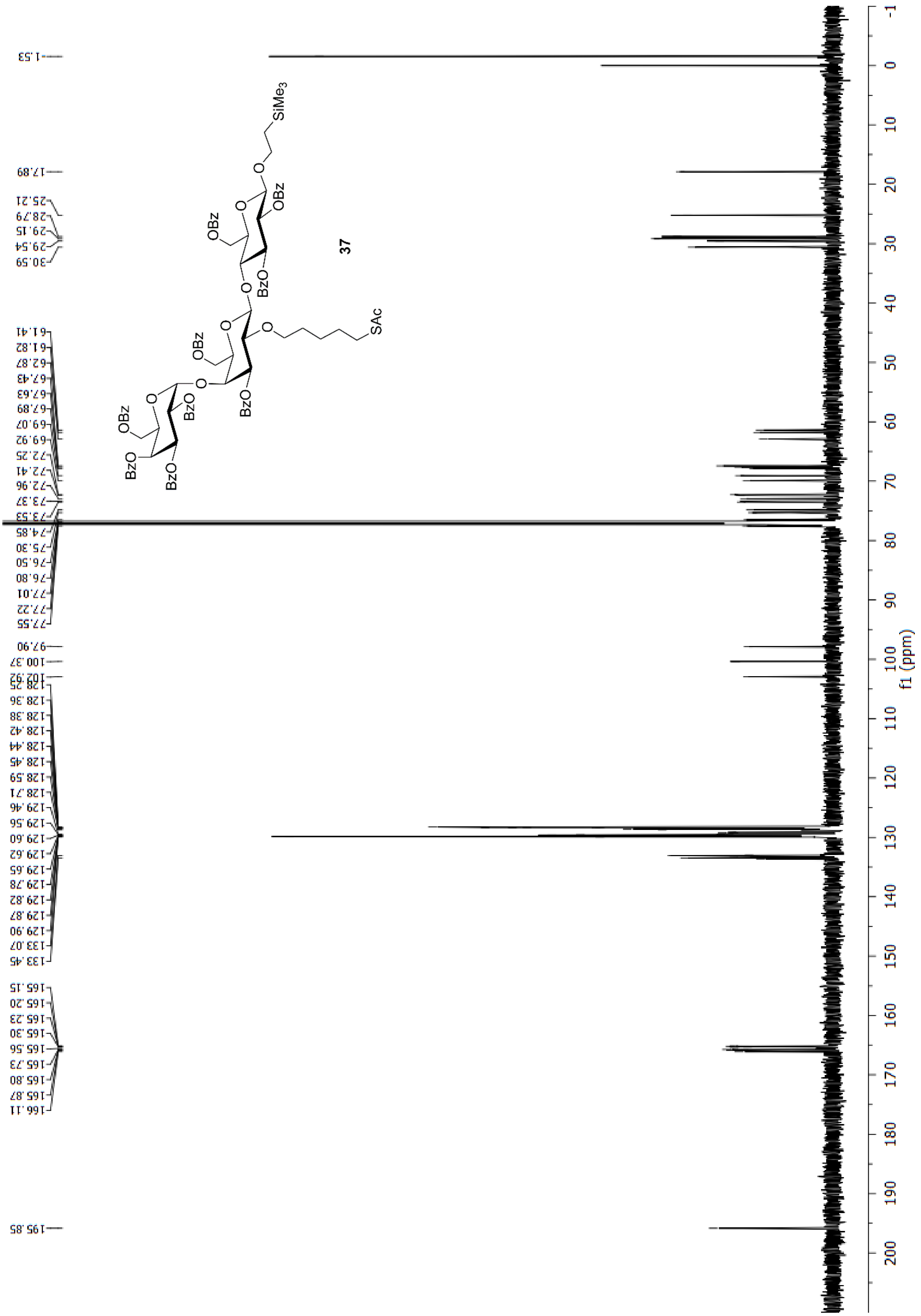
-1.53
17.89
25.21
28.79
29.15
29.54
30.59
61.41
61.82
62.87
67.43
67.63
67.89
69.07
69.92
72.25
72.41
72.96
73.37
73.53
74.85
75.30
76.50
76.80
77.01
77.22
77.55
97.90
100.37
102.92
128.25
128.36
128.38
128.42
128.44
128.45
128.59
128.71
129.46
129.56
129.60
129.62
129.65
129.78
129.82
129.87
129.90
133.07
133.45
165.15
165.20
165.23
165.30
165.56
165.73
165.80
165.87
166.11
195.85
SiMe3
OBz
BzO
SAc
37
f1 (ppm)

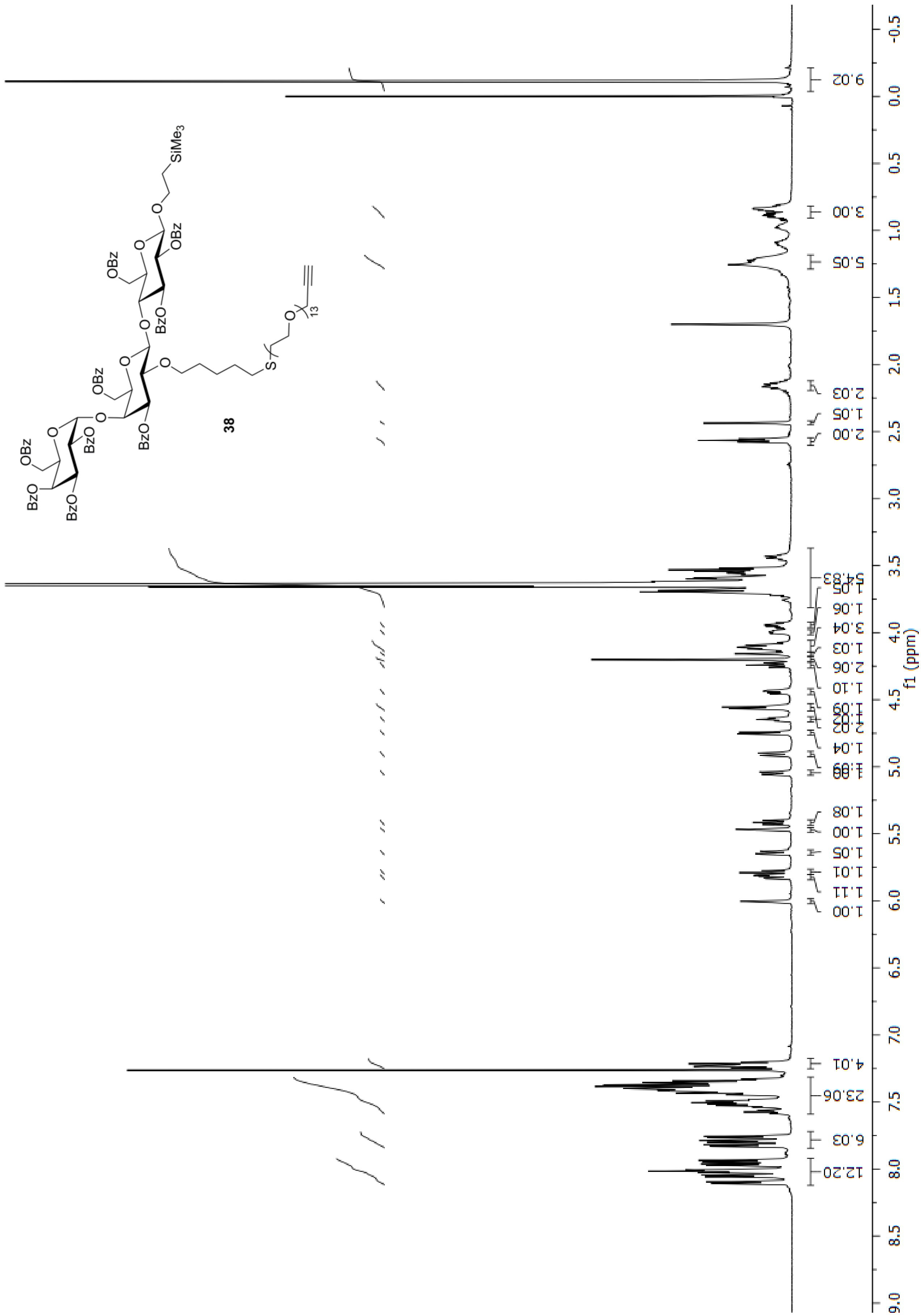
38
SiMe3
OBz
BzO
f1 (ppm)

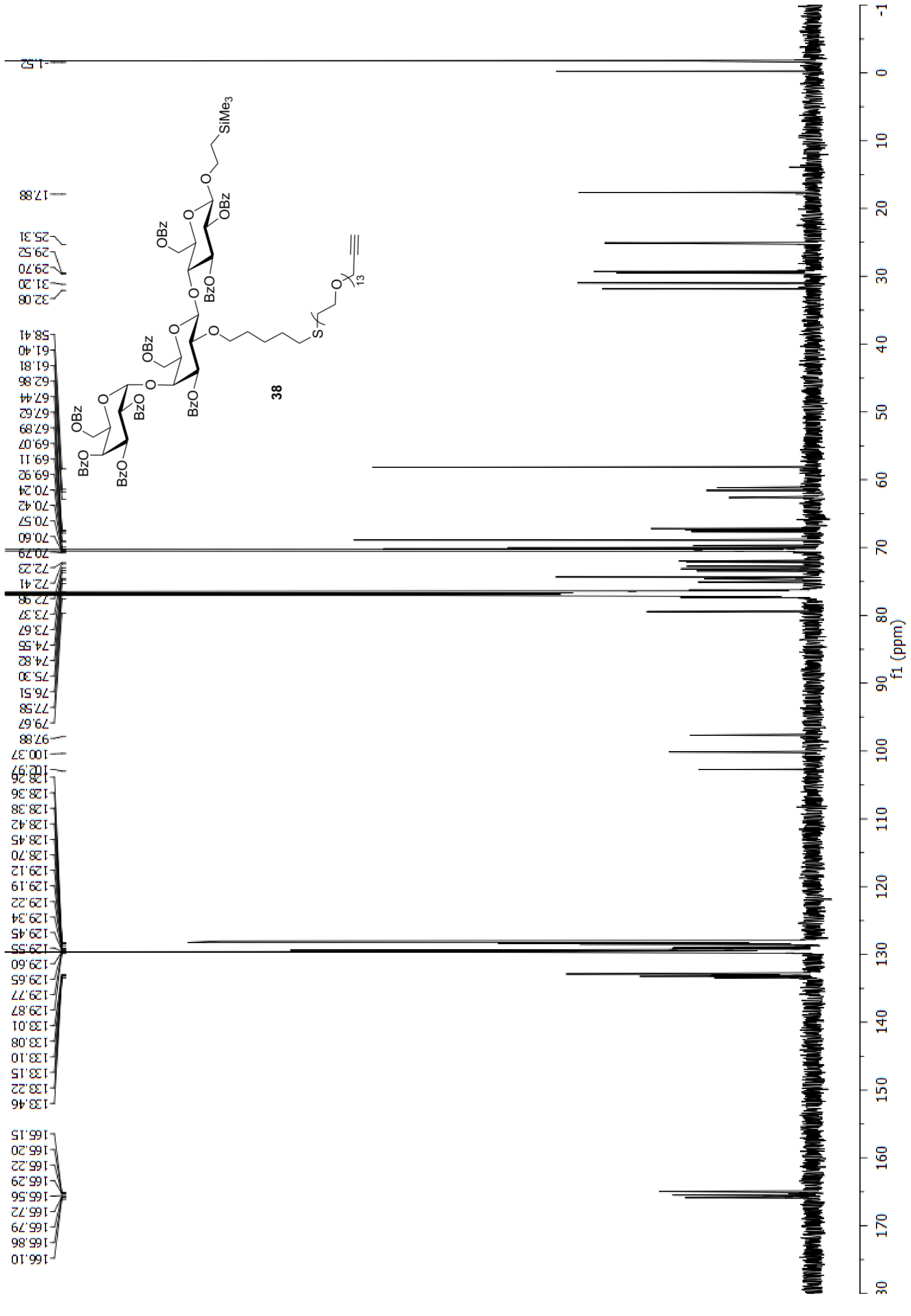
-1.52
17.88
25.31
29.52
29.70
31.20
32.08
58.41
61.40
61.81
62.86
67.44
67.62
67.89
69.07
69.11
69.92
70.24
70.42
70.57
70.60
70.79
72.23
72.41
72.96
73.37
73.67
74.55
74.82
75.30
76.51
77.58
79.67
97.88
100.37
102.97
128.26
128.36
128.38
128.42
128.45
128.70
129.12
129.19
129.22
129.34
129.45
129.55
129.60
129.65
129.77
129.87
133.01
133.08
133.10
133.15
133.22
133.46
165.15
165.20
165.22
165.29
165.56
165.72
165.79
165.86
166.10
SiMe3
OBz
BzO
13
38
f1 (ppm)

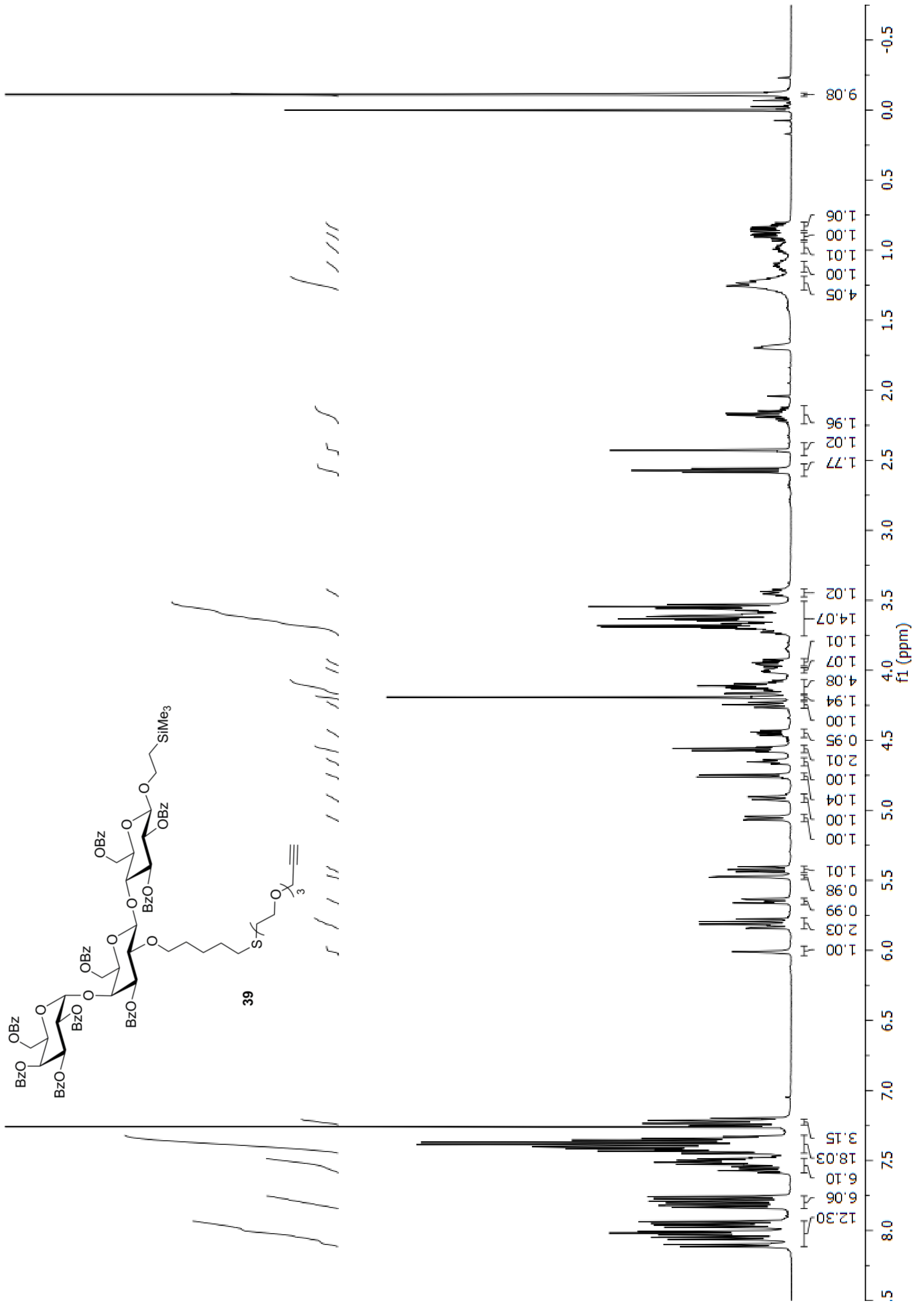
BzO
BzO
OBz
BzO
BzO
OBz
BzO
OBz
OBz
SiMe$_3$
S
O
3
39
f1 (ppm)

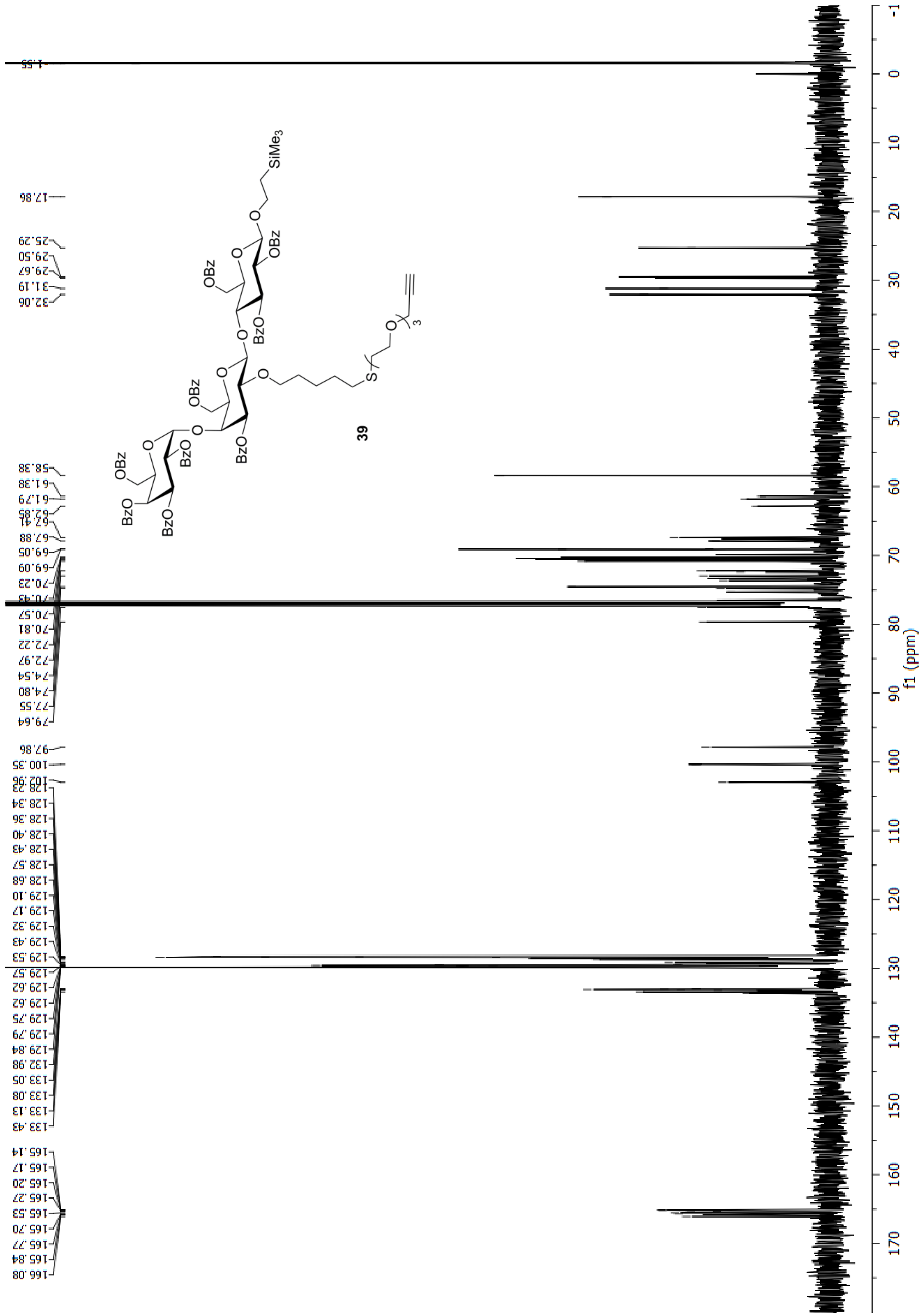
SiMe3
OBz
BzO
S
3
39
f1 (ppm)

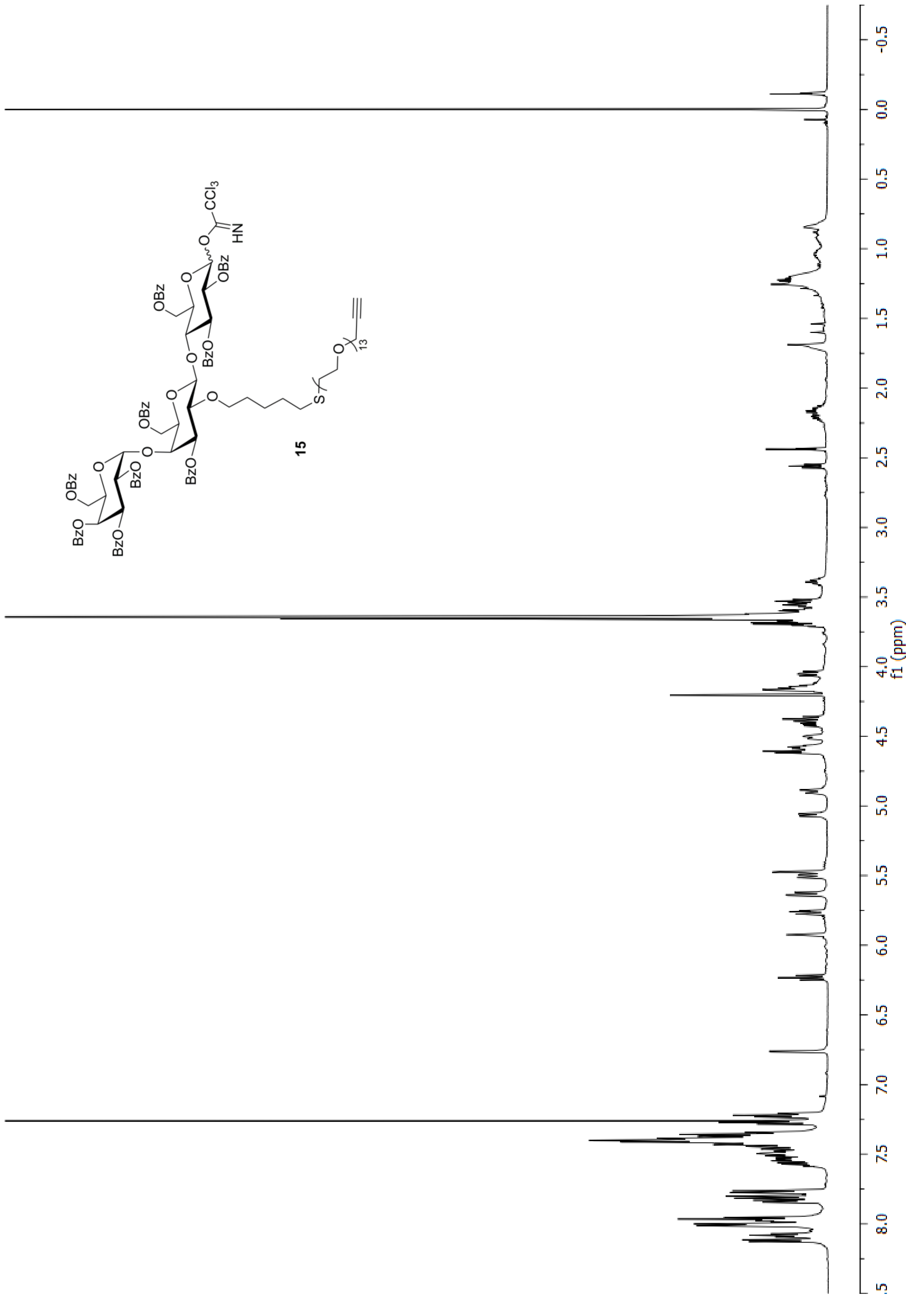

OBz
BzO
BzO
OBz
BzO
BzO
OBz
BzO
OBz
O
HN
CCl3
S
13
15
f1 (ppm)
-0.5
0.0
0.5
1.0
1.5
2.0
2.5
3.0
3.5
4.0
4.5
5.0
5.5
6.0
6.5
7.0
7.5
8.0

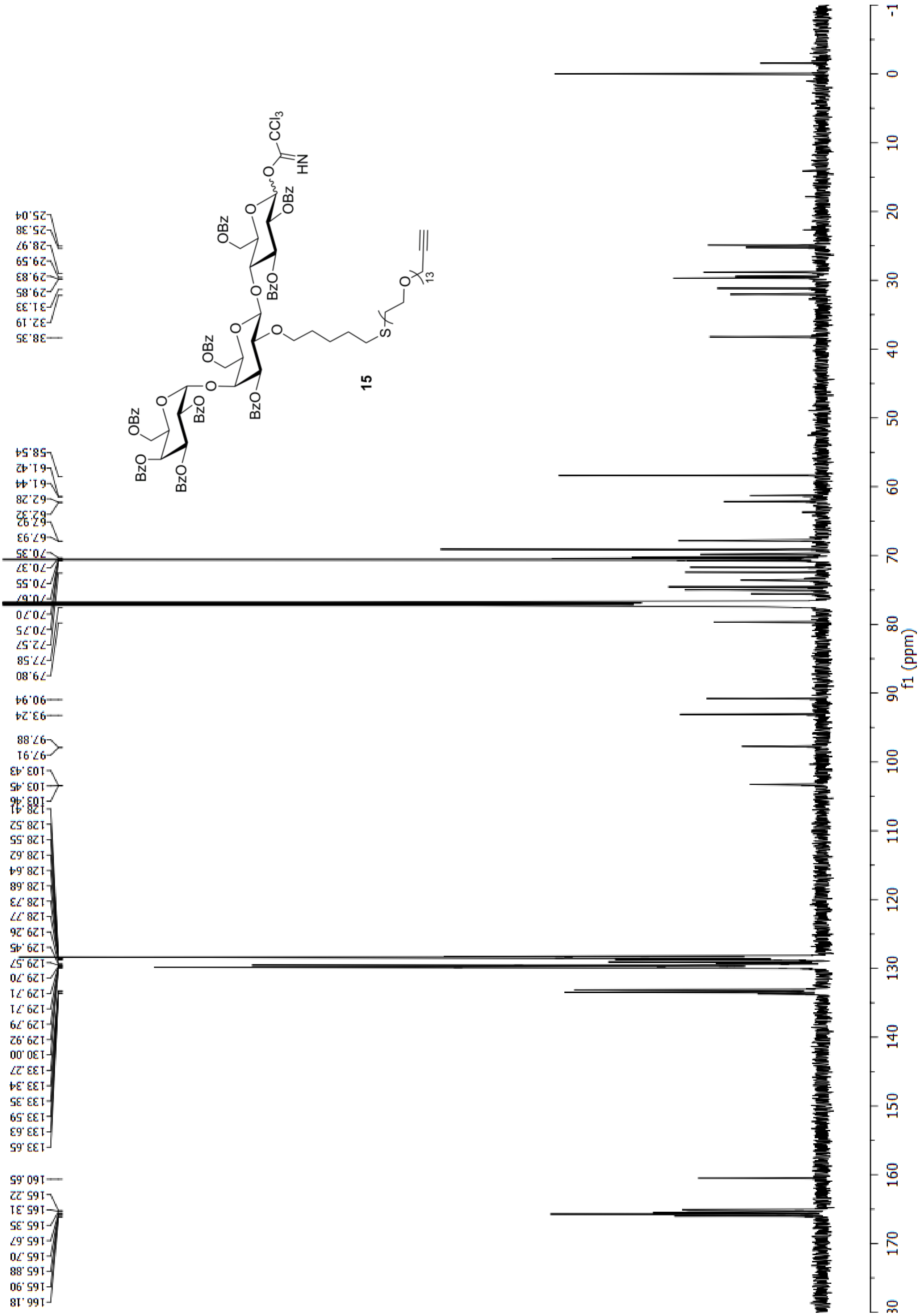

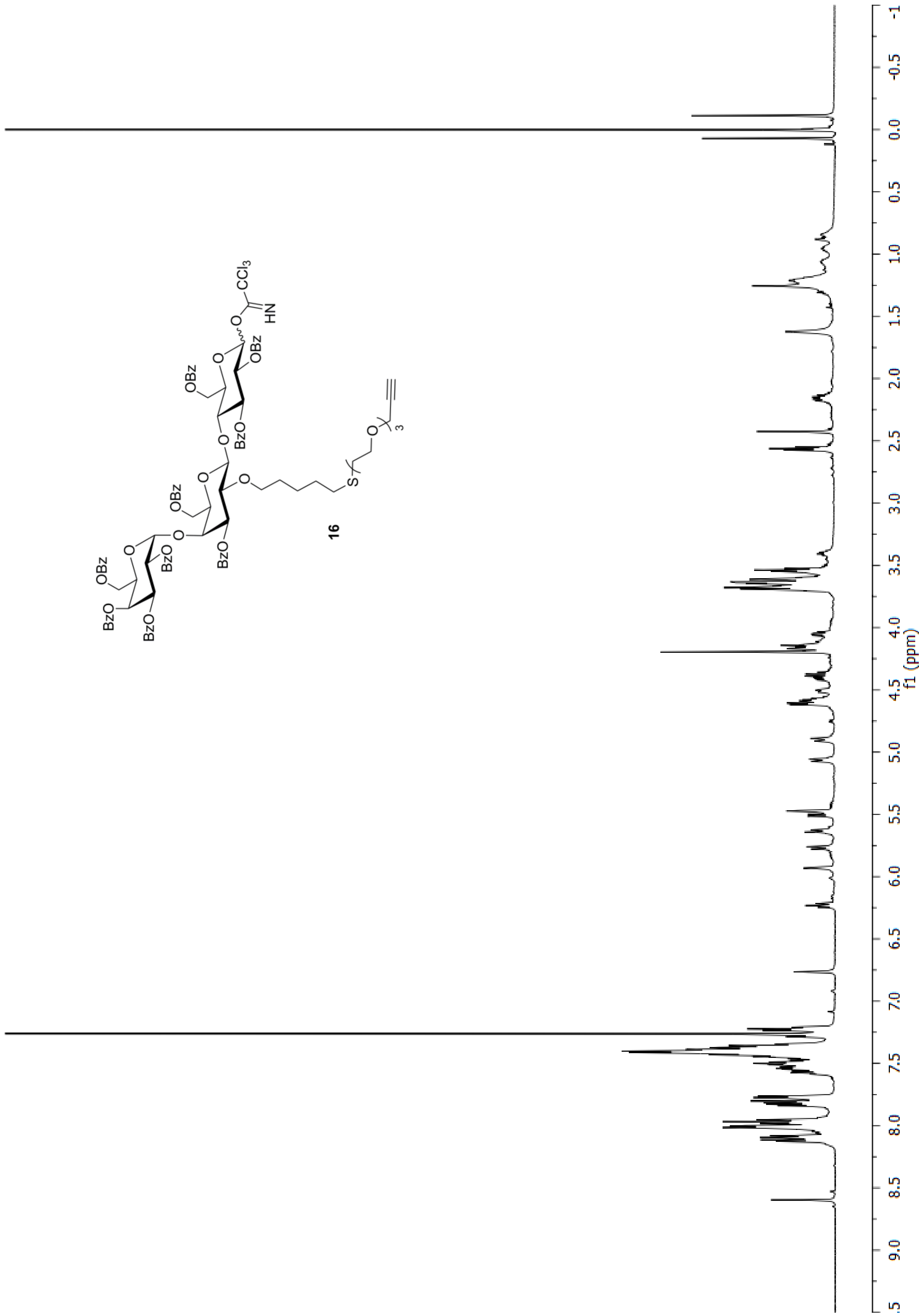
16
OBz
BzO
BzO
BzO
OBz
BzO
OBz
BzO
OBz
O
CCl3
HN
S
3
f1 (ppm)
9.5
9.0
8.5
8.0
7.5
7.0
6.5
6.0
5.5
5.0
4.5
4.0
3.5
3.0
2.5
2.0
1.5
1.0
0.5
0.0
-0.5
-1

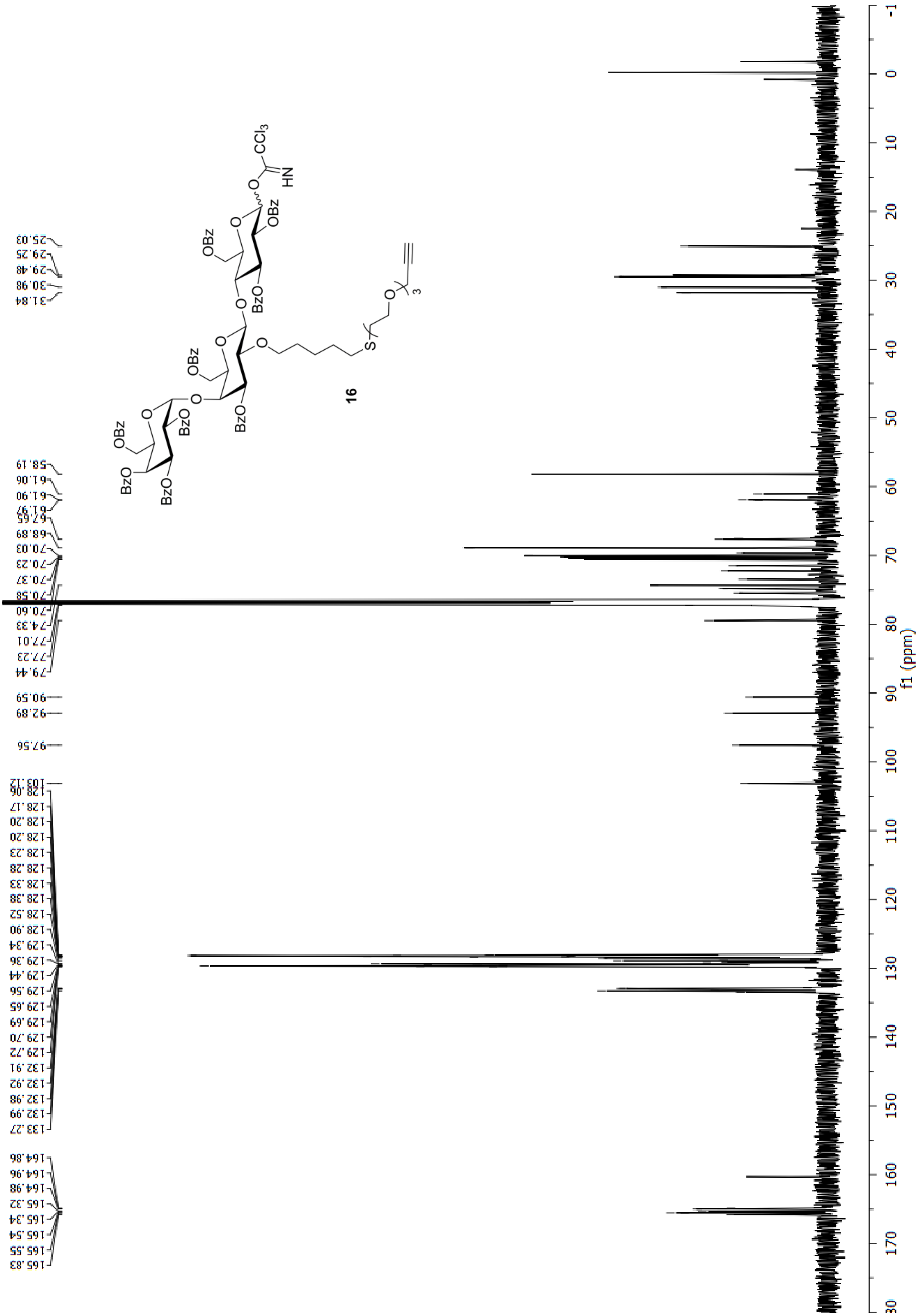
16
f1 (ppm)

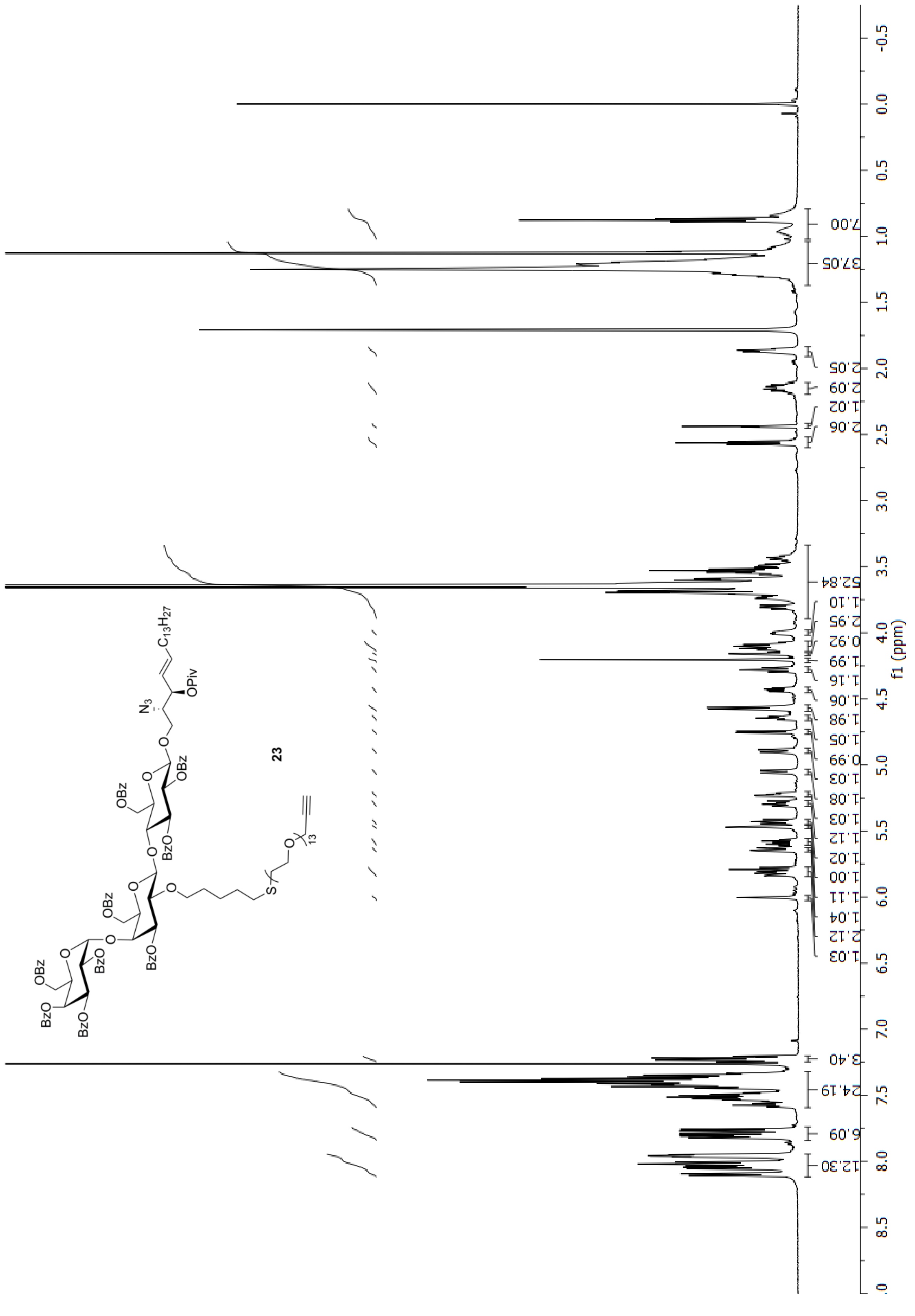
23
f1 (ppm)

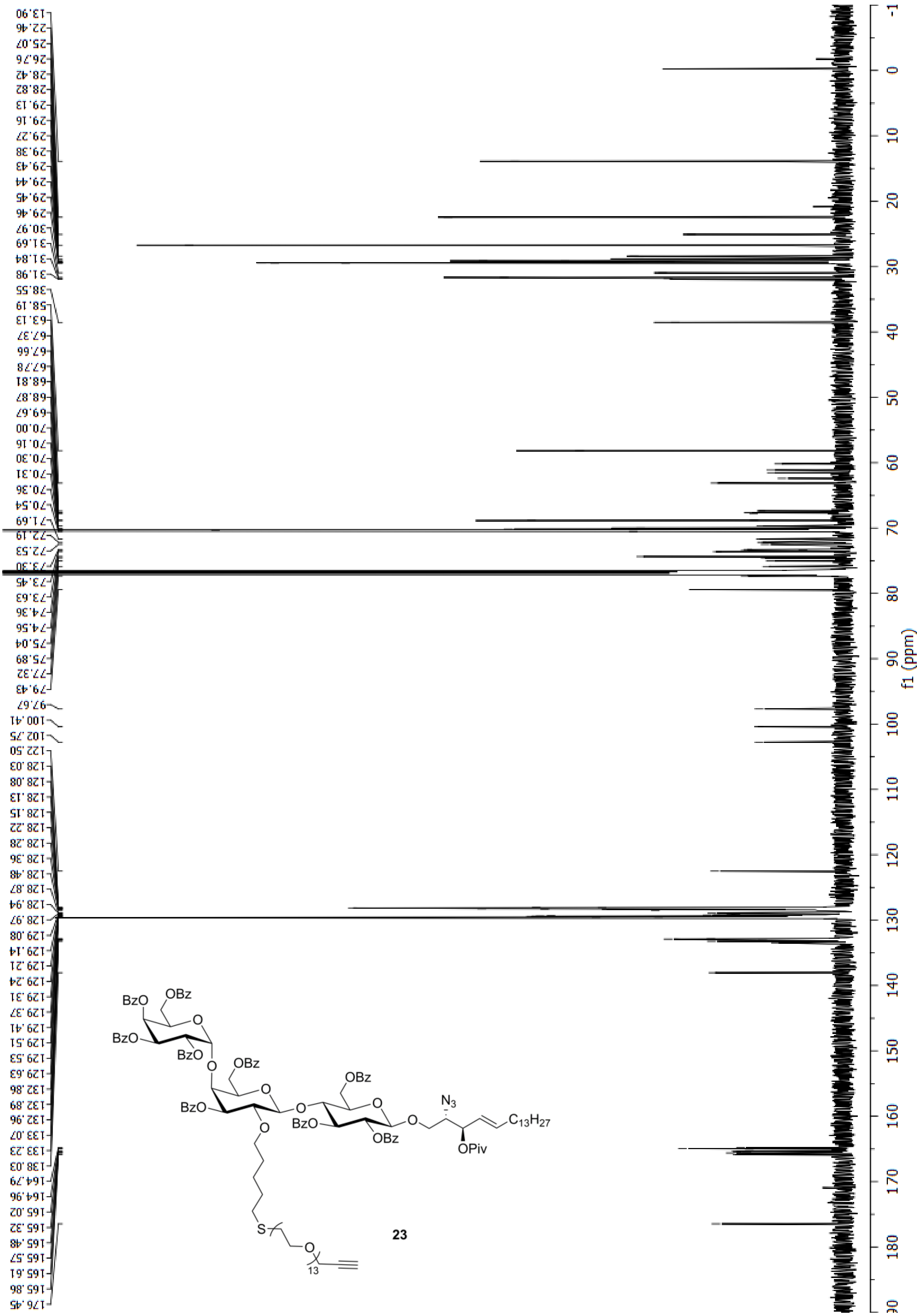
13.90
22.46
25.07
26.76
28.42
28.82
29.13
29.16
29.27
29.38
29.43
29.44
29.45
29.46
30.97
31.69
31.84
31.98
38.55
58.19
63.13
67.37
67.66
67.78
68.81
68.87
69.67
70.00
70.16
70.30
70.31
70.36
70.54
71.69
72.19
72.53
73.30
73.45
73.63
74.36
74.56
75.04
75.89
77.32
79.43
97.67
100.41
102.75
122.50
128.03
128.08
128.13
128.15
128.22
128.28
128.36
128.48
128.87
128.94
128.97
129.08
129.14
129.21
129.24
129.31
129.37
129.41
129.51
129.53
129.63
132.86
132.89
132.96
133.07
133.23
138.03
164.79
164.96
165.02
165.32
165.48
165.57
165.61
165.86
176.45
BzO
OBz
BzO
BzO
OBz
BzO
OBz
BzO
OBz
N3
C13H27
OPiv
S
13
f1 (ppm)

23

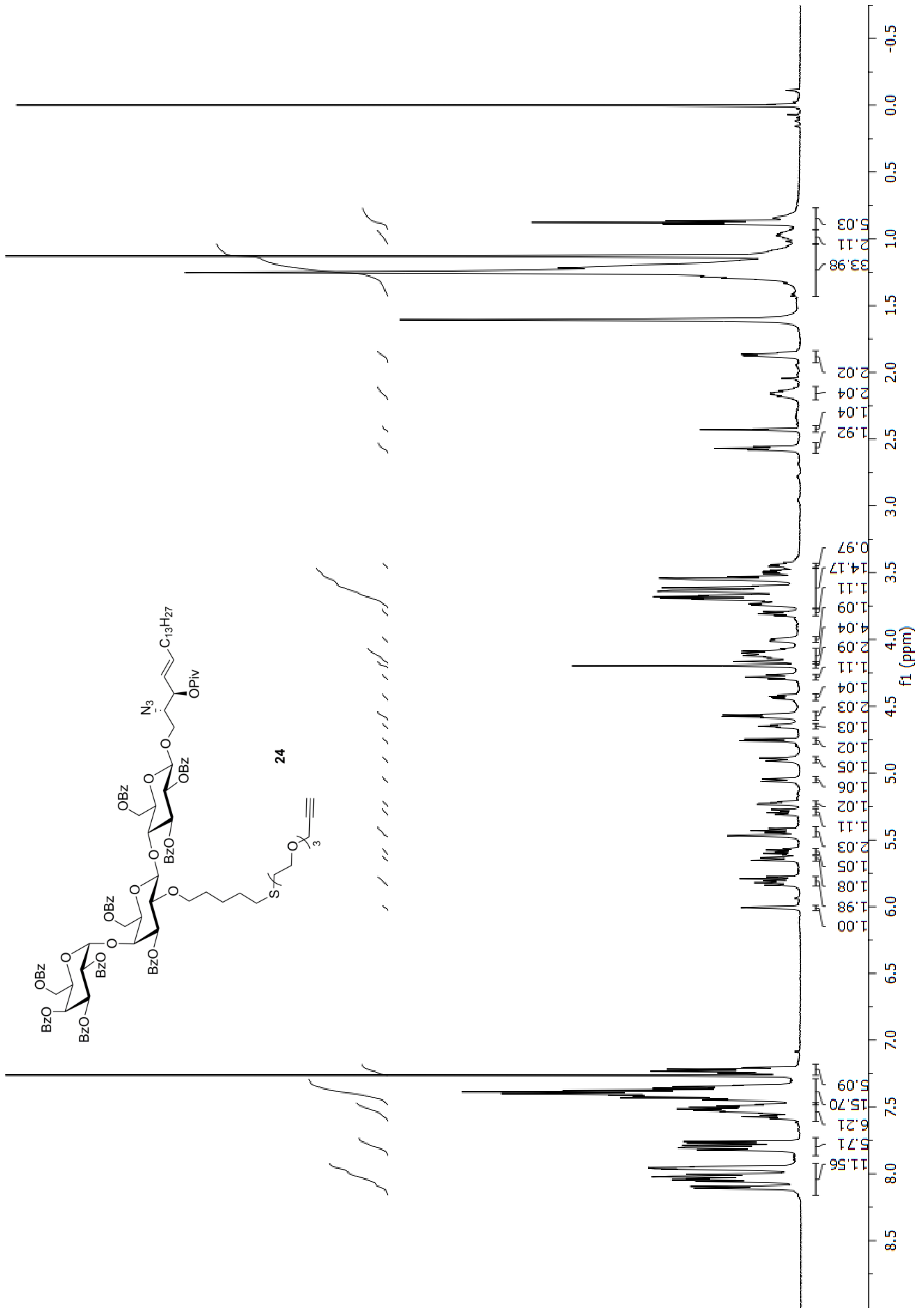
24
f1 (ppm)

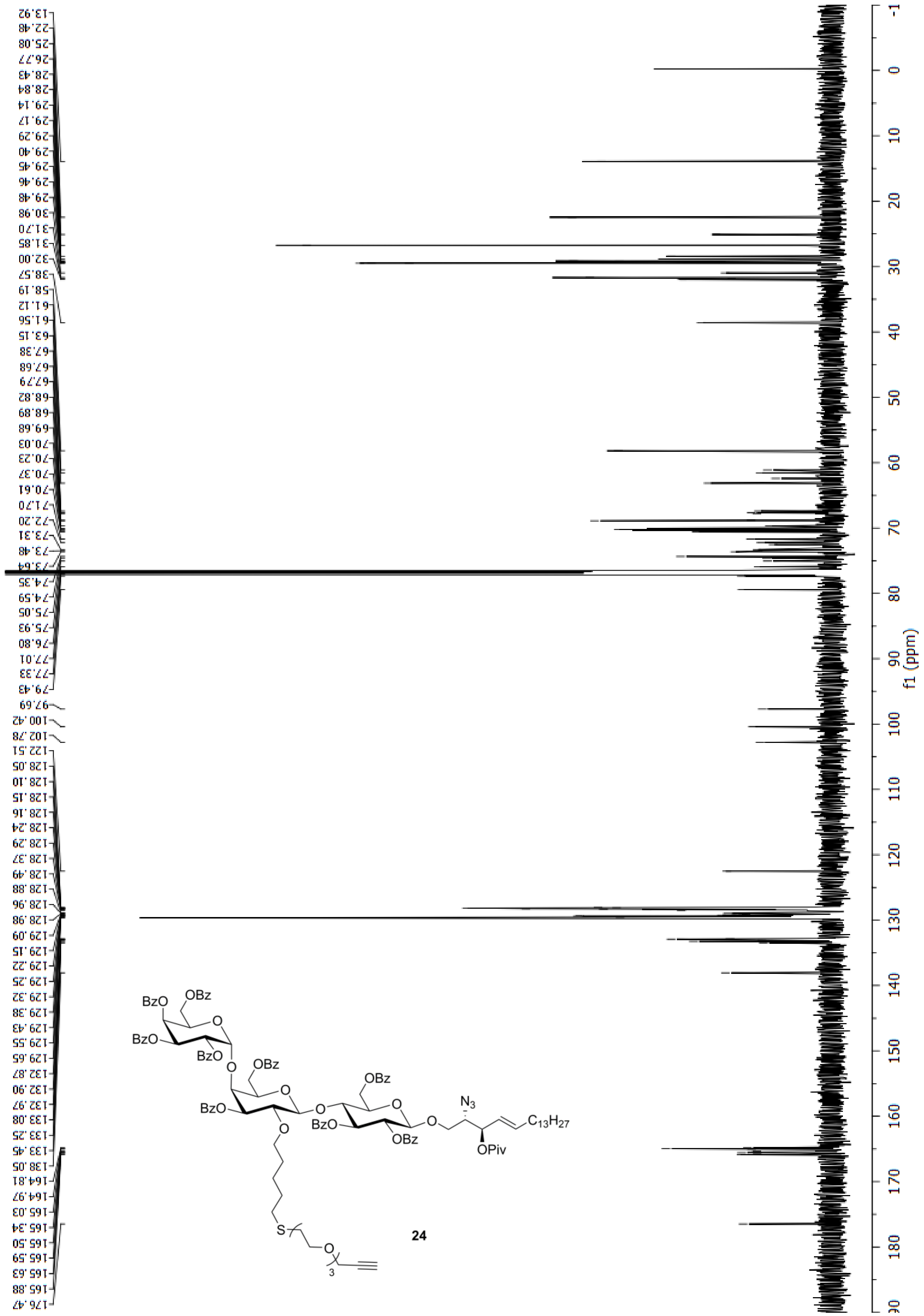
13.92
22.48
25.08
26.77
28.43
28.84
29.14
29.17
29.29
29.40
29.45
29.46
29.48
30.98
31.70
31.85
32.00
38.57
58.19
61.12
61.56
63.15
67.38
67.68
67.79
68.82
68.89
69.68
70.03
70.23
70.37
70.61
71.70
72.20
73.31
73.48
73.64
74.35
74.59
75.05
75.93
76.80
77.01
77.33
79.43
97.69
100.42
102.78
122.51
128.05
128.10
128.15
128.16
128.24
128.29
128.37
128.49
128.88
128.96
128.98
129.09
129.15
129.22
129.25
129.32
129.38
129.43
129.55
129.65
132.87
132.90
132.97
133.08
133.25
133.45
138.05
164.81
164.97
165.03
165.34
165.50
165.59
165.63
165.88
176.47
f1 (ppm)
BzO
OBz
BzO
BzO
OBz
BzO
OBz
BzO
OBz
N3
C13H27
OPiv
S
O
3
24

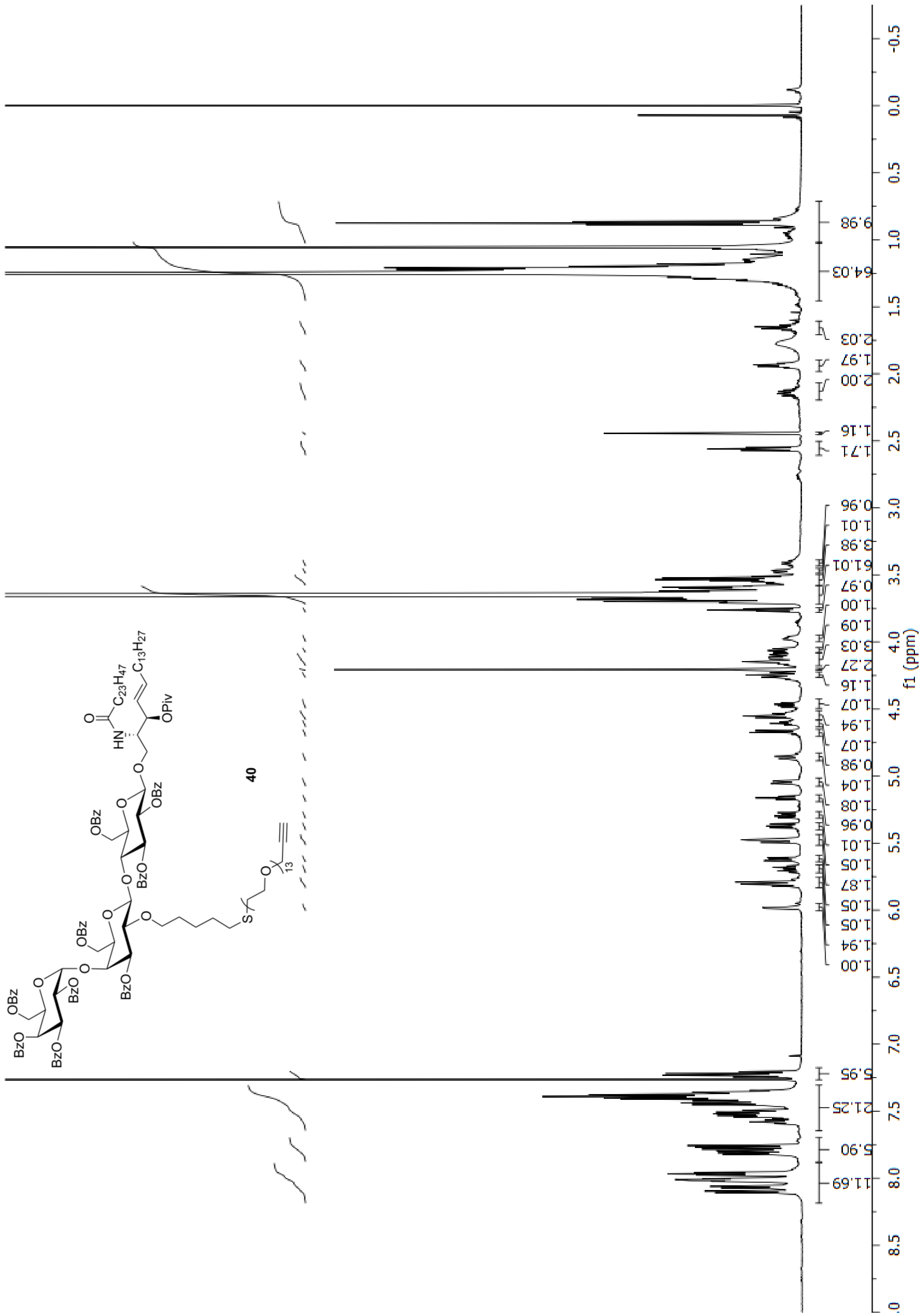
40
f1 (ppm)

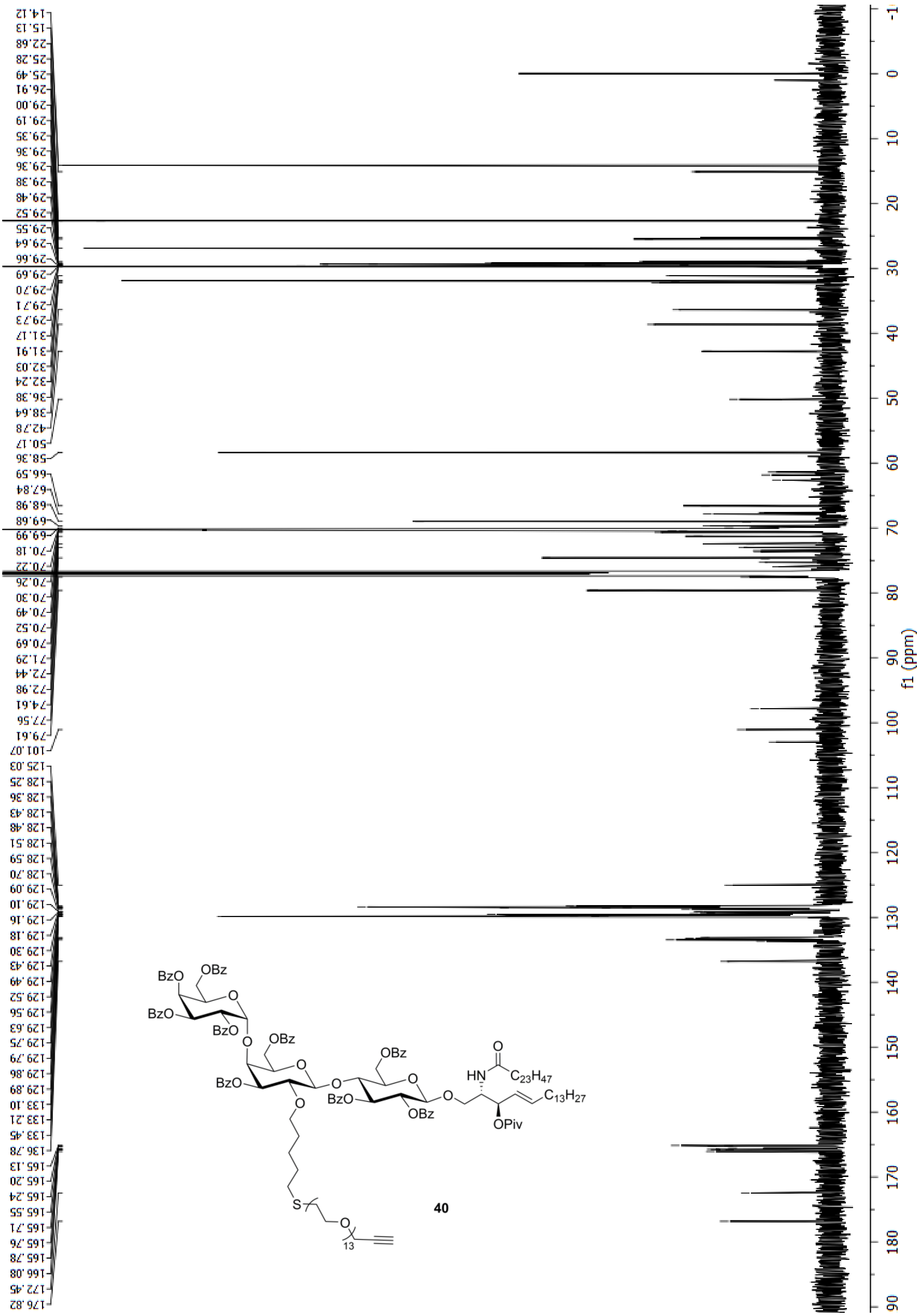
14.12
15.13
22.68
25.28
25.49
26.91
29.00
29.19
29.35
29.36
29.36
29.38
29.48
29.52
29.55
29.64
29.66
29.69
29.70
29.71
29.73
31.17
31.91
32.03
32.24
36.38
38.64
42.78
50.17
58.36
66.59
67.84
68.98
69.68
69.99
70.18
70.22
70.26
70.30
70.49
70.52
70.69
71.29
72.44
72.98
74.61
77.56
79.61
101.07
125.03
128.25
128.36
128.43
128.48
128.51
128.59
128.70
129.09
129.10
129.16
129.18
129.30
129.43
129.49
129.52
129.56
129.63
129.75
129.79
129.86
129.89
133.10
133.21
133.45
136.78
165.13
165.20
165.24
165.55
165.71
165.76
165.78
166.08
172.45
176.82
f1 (ppm)
BzO
OBz
BzO
BzO
OBz
BzO
OBz
BzO
OBz
HN
$C_{23}H_{47}$
$C_{13}H_{27}$
OPiv
S
13
40

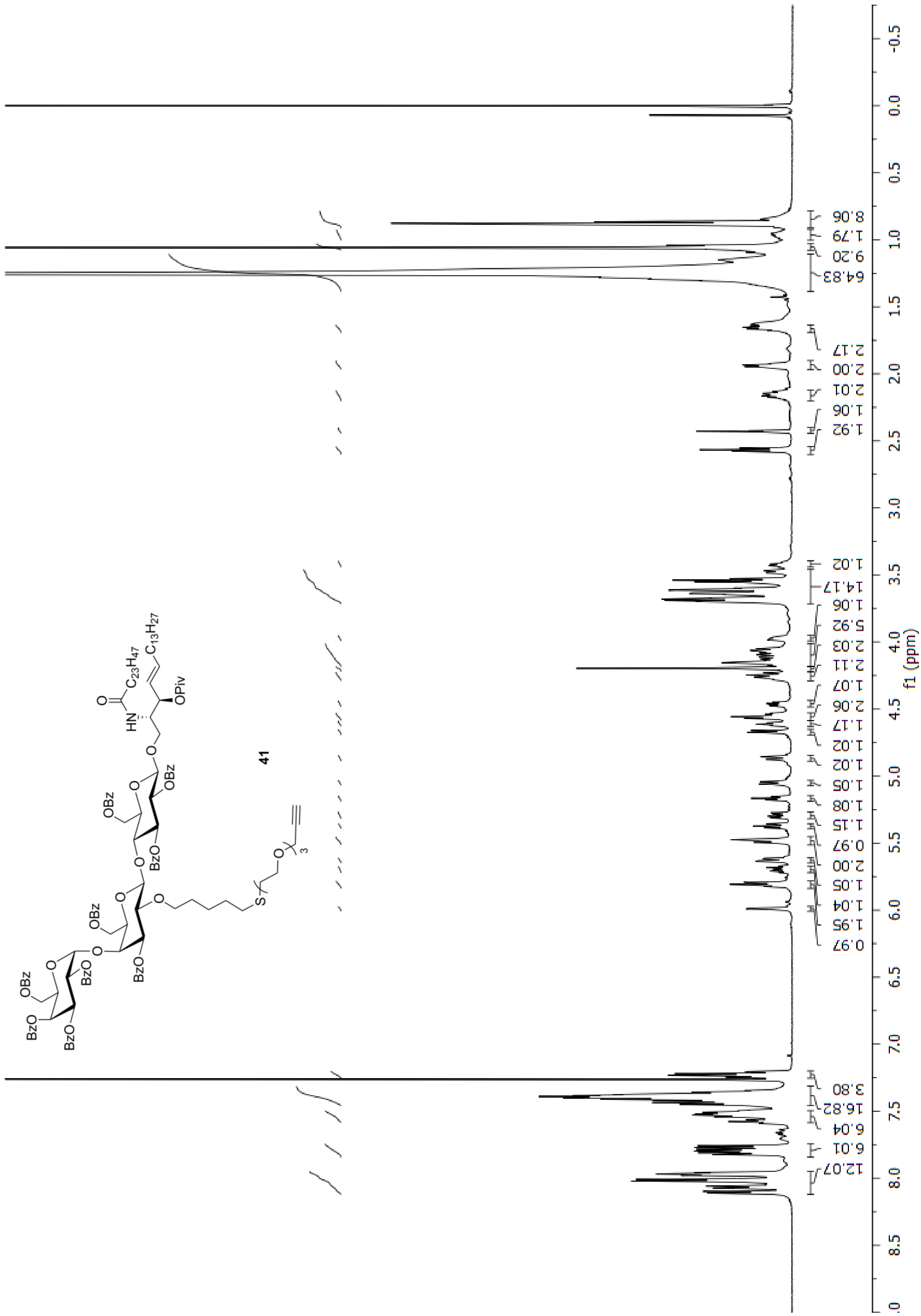

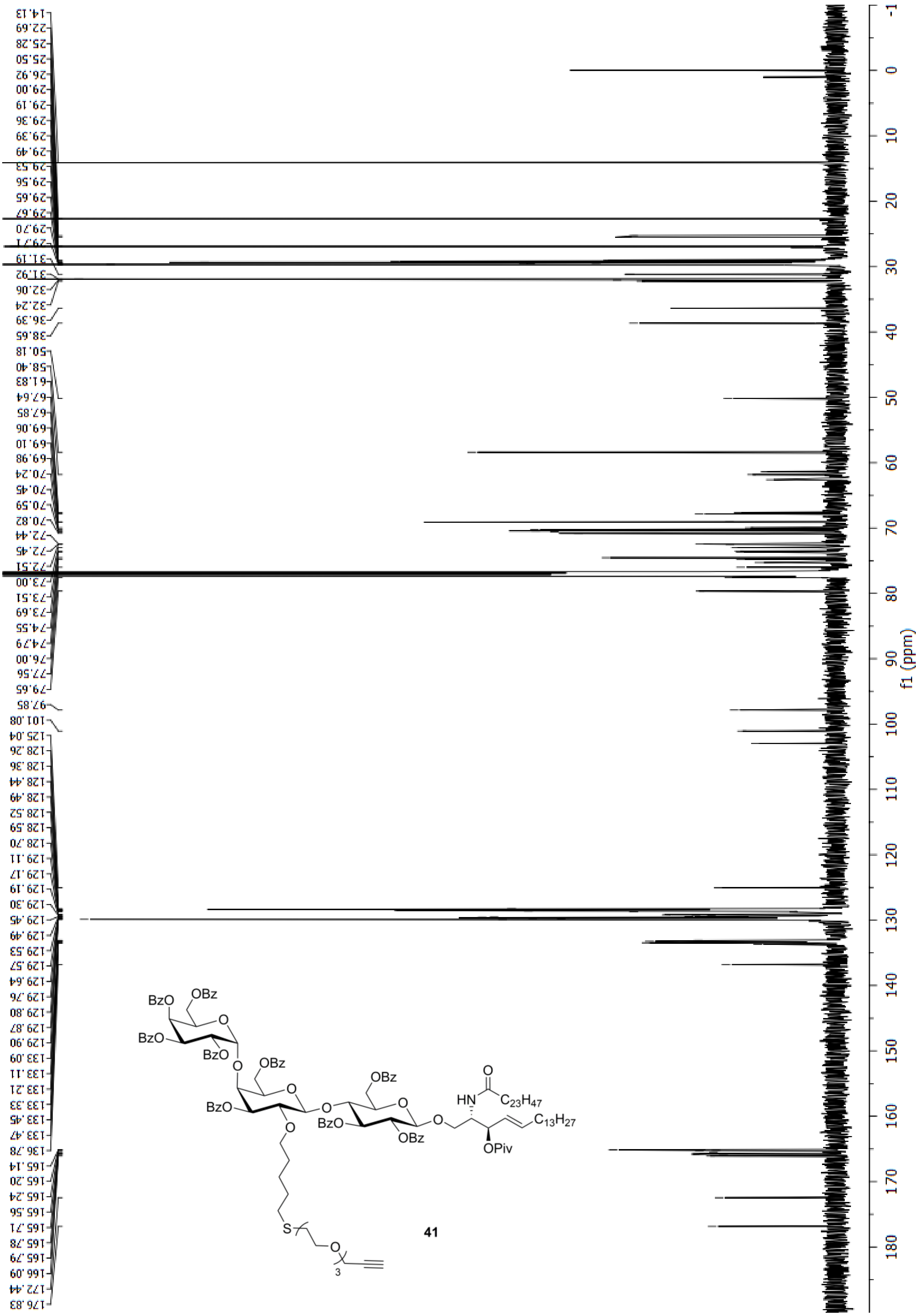
14.13
22.69
25.28
25.50
26.92
29.00
29.19
29.36
29.39
29.49
29.53
29.56
29.65
29.67
29.70
29.71
31.19
31.92
32.06
32.24
36.39
38.65
50.18
58.40
61.83
62.64
62.85
69.06
69.10
69.98
70.24
70.45
70.59
70.82
72.44
72.45
72.51
73.00
73.51
73.69
74.55
74.79
76.00
77.56
79.65
97.85
101.08
125.04
128.26
128.36
128.44
128.49
128.52
128.59
128.70
129.11
129.17
129.19
129.30
129.45
129.49
129.53
129.57
129.64
129.76
129.80
129.87
129.90
133.09
133.11
133.21
133.33
133.45
133.47
136.78
165.14
165.20
165.24
165.56
165.71
165.78
165.79
166.09
172.44
176.83
f1 (ppm)
BzO
OBz
HN
$C_{23}H_{47}$
$C_{13}H_{27}$
OPiv
S
3
41

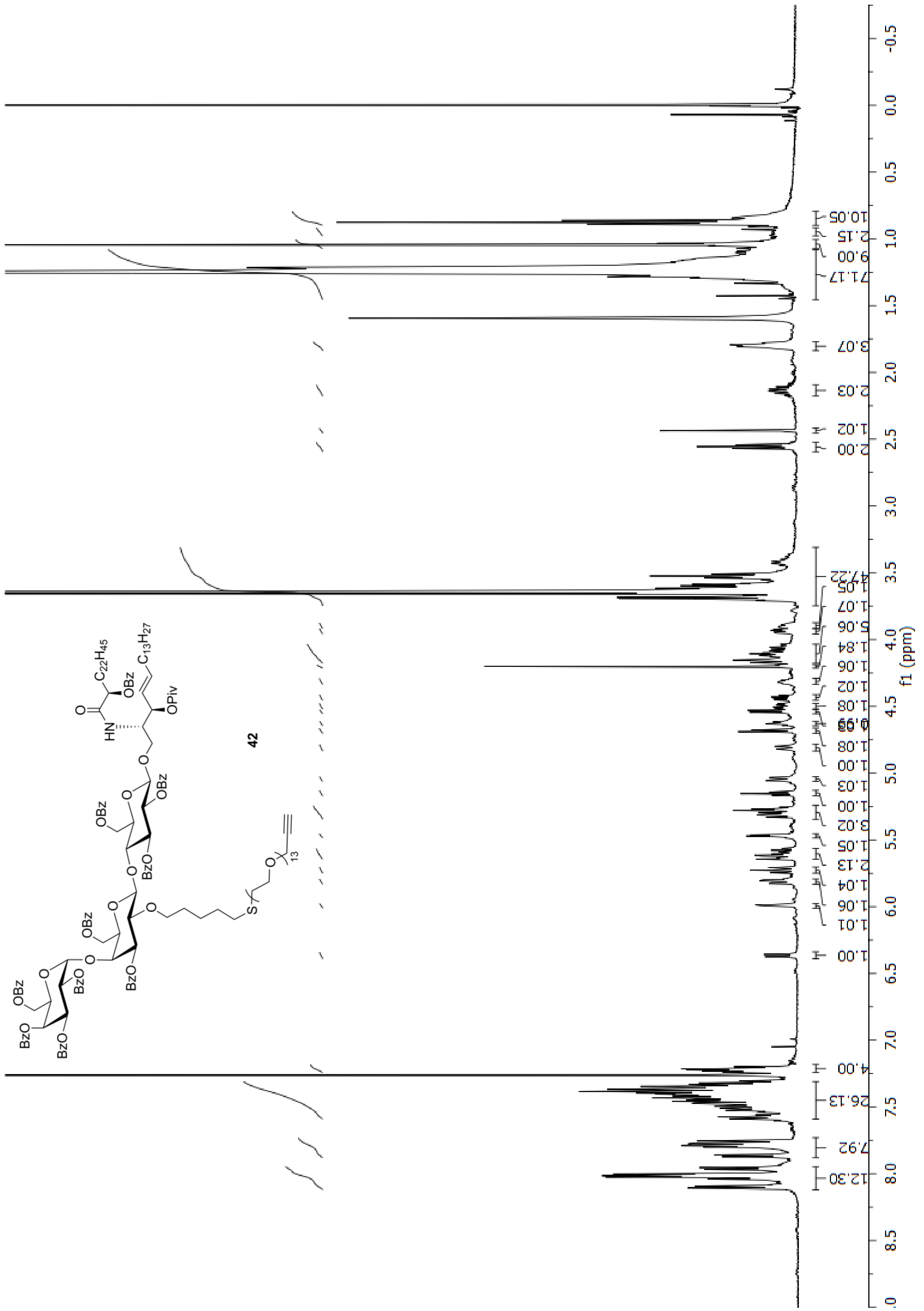

42

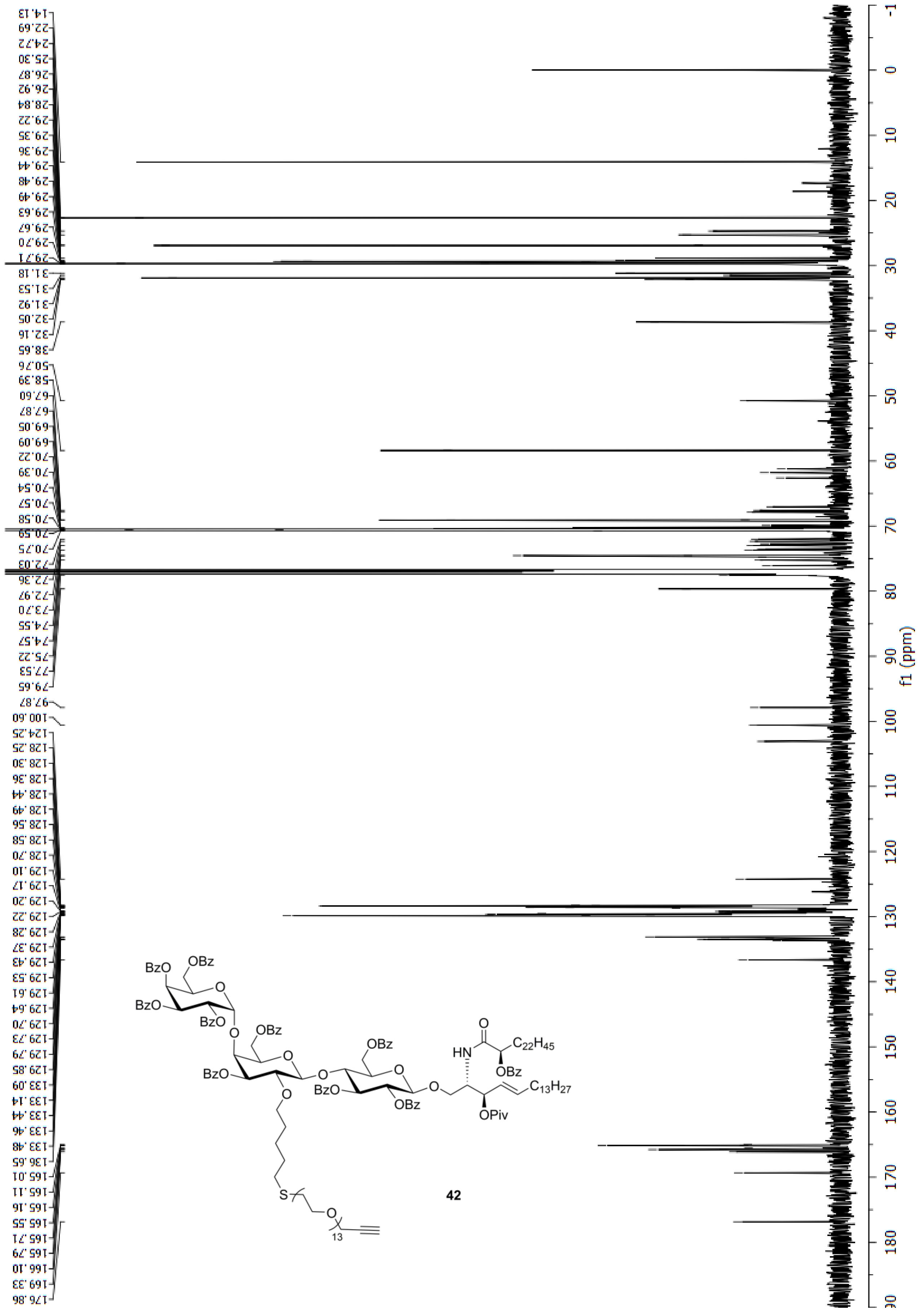
14.13
22.69
24.72
25.30
26.87
26.92
28.84
29.22
29.35
29.36
29.44
29.48
29.49
29.63
29.67
29.70
29.71
31.18
31.53
31.92
32.05
32.16
38.65
50.76
58.39
67.60
67.87
69.05
69.09
70.22
70.39
70.54
70.57
70.58
70.59
70.75
72.03
72.36
72.97
73.70
74.55
74.57
75.22
77.53
79.65
97.87
100.60
124.25
128.25
128.30
128.36
128.44
128.49
128.56
128.58
128.70
129.10
129.17
129.20
129.22
129.28
129.37
129.43
129.53
129.61
129.64
129.70
129.73
129.79
129.85
133.09
133.14
133.44
133.46
133.48
136.65
165.01
165.11
165.16
165.55
165.71
165.79
166.10
169.33
176.86
f1 (ppm)
BzO
OBz
BzO
BzO
OBz
BzO
OBz
BzO
OBz
HN
$C_{22}H_{45}$
OBz
$C_{13}H_{27}$
OPiv
S
13
42

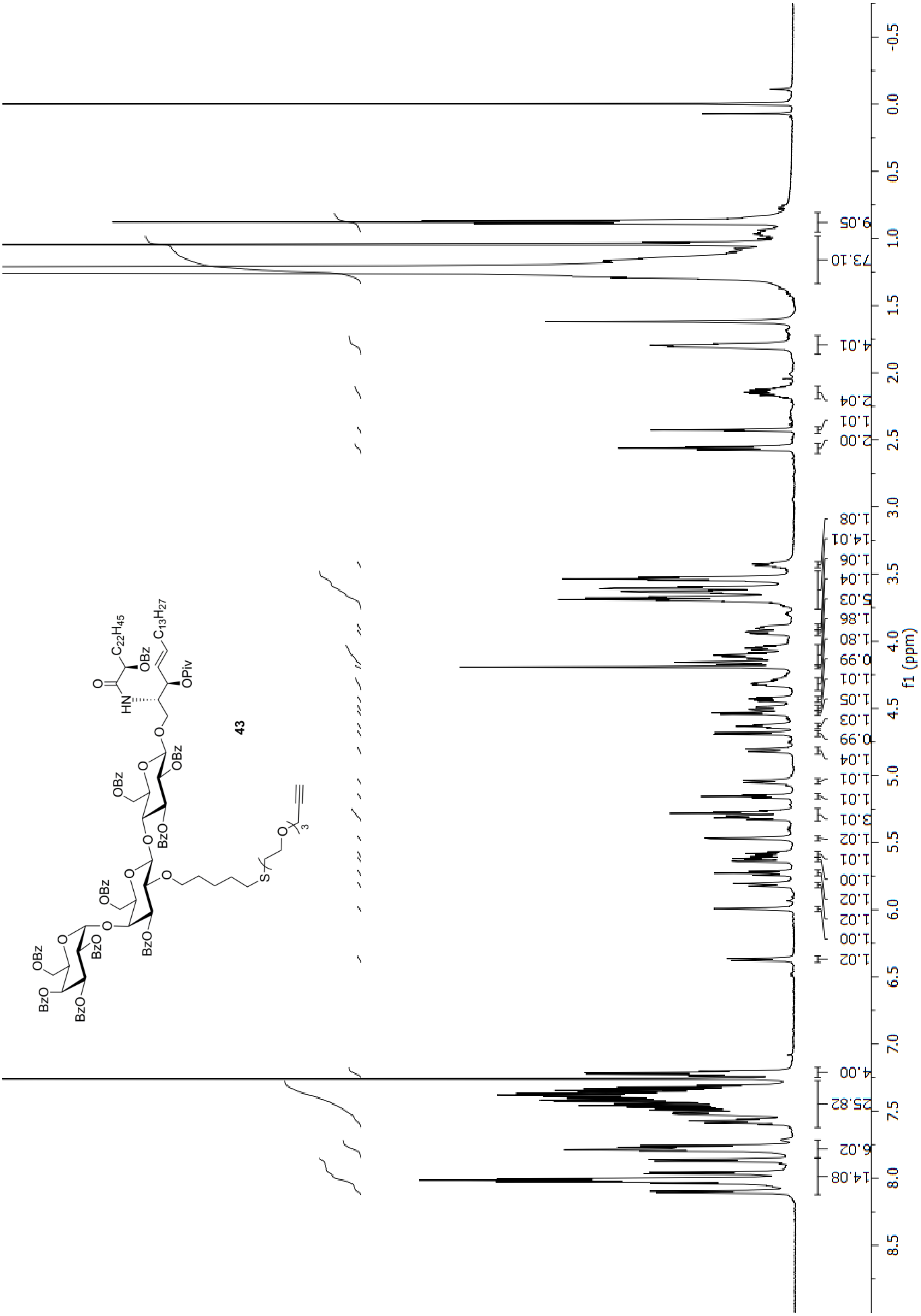
43
f1 (ppm)

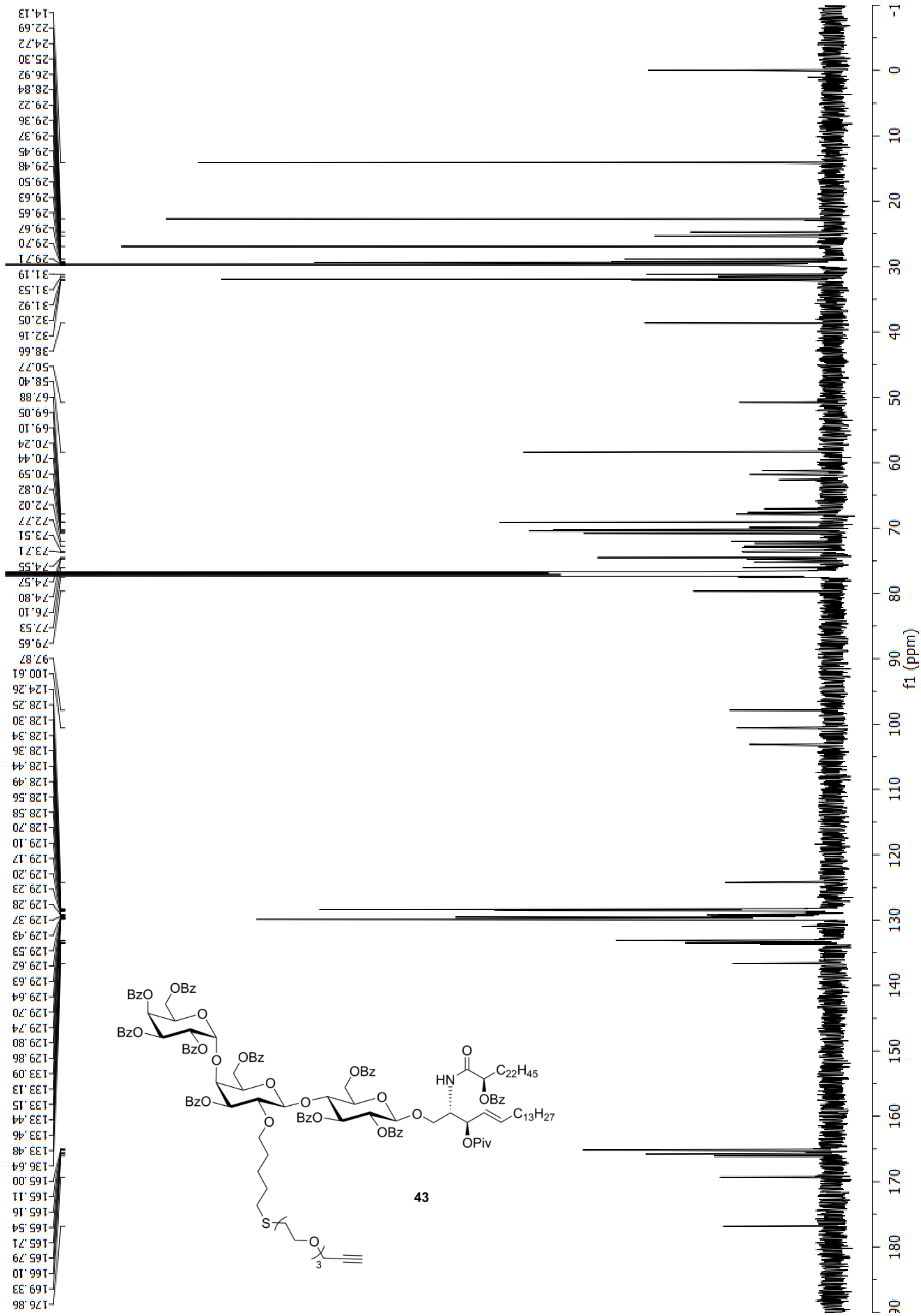
14.13
22.69
24.72
25.30
26.92
28.84
29.22
29.36
29.37
29.45
29.48
29.50
29.63
29.65
29.67
29.70
29.71
31.19
31.53
31.92
32.05
32.16
38.66
50.77
58.40
67.88
69.05
69.10
70.24
70.44
70.59
70.82
72.02
72.27
73.51
73.71
74.55
74.57
74.80
76.10
77.53
79.65
97.87
100.61
124.26
128.25
128.30
128.34
128.36
128.44
128.49
128.56
128.58
128.70
129.10
129.17
129.20
129.23
129.28
129.37
129.43
129.53
129.62
129.63
129.64
129.70
129.74
129.80
129.86
133.09
133.13
133.15
133.44
133.46
133.48
136.64
165.00
165.11
165.16
165.54
165.71
165.79
166.10
169.33
176.86
f1 (ppm)
BzO
OBz
BzO
BzO
OBz
BzO
OBz
BzO
OBz
HN
C22H45
OBz
C13H27
OPiv
S
3
43

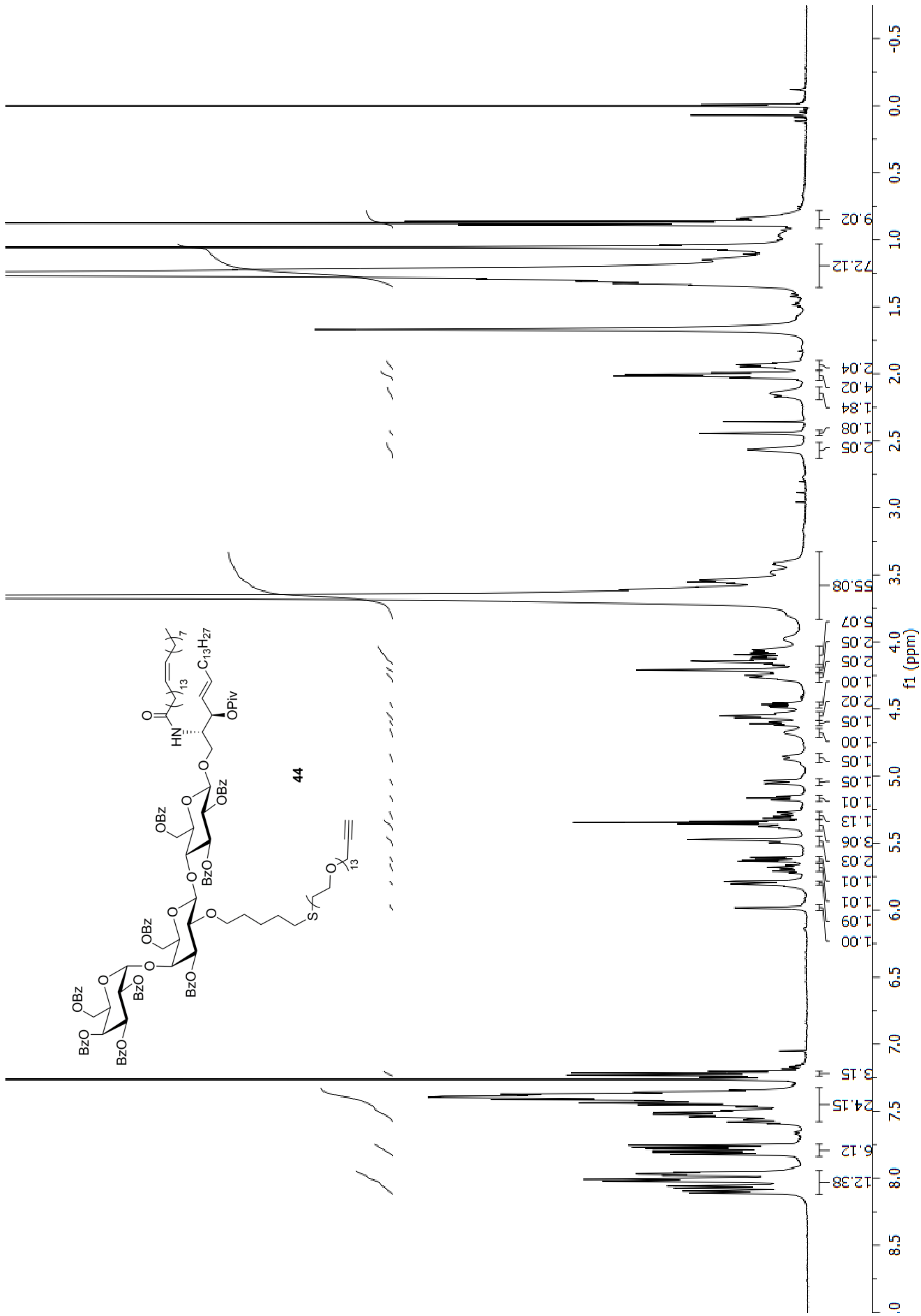
44
f1 (ppm)

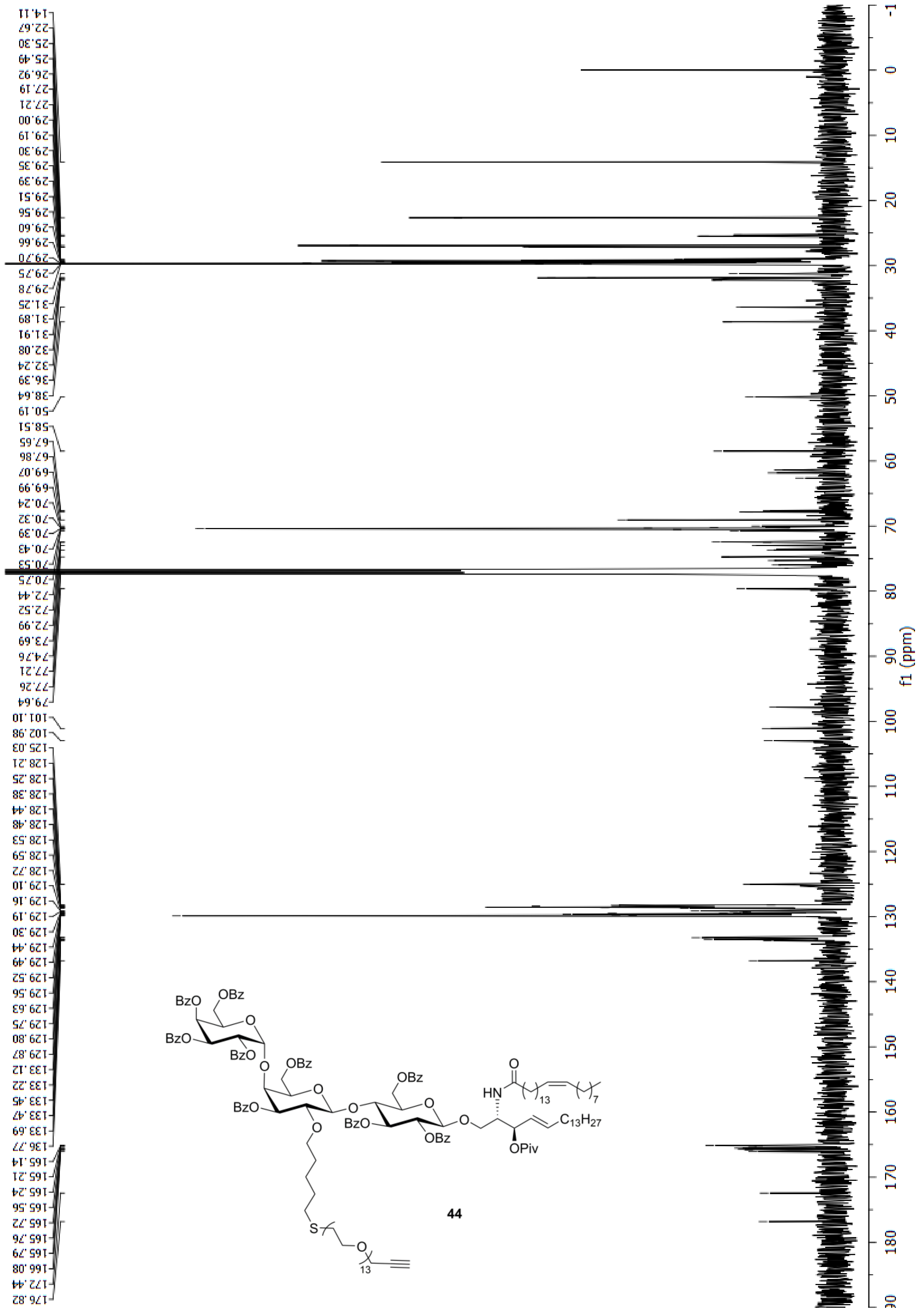
44
f1 (ppm)

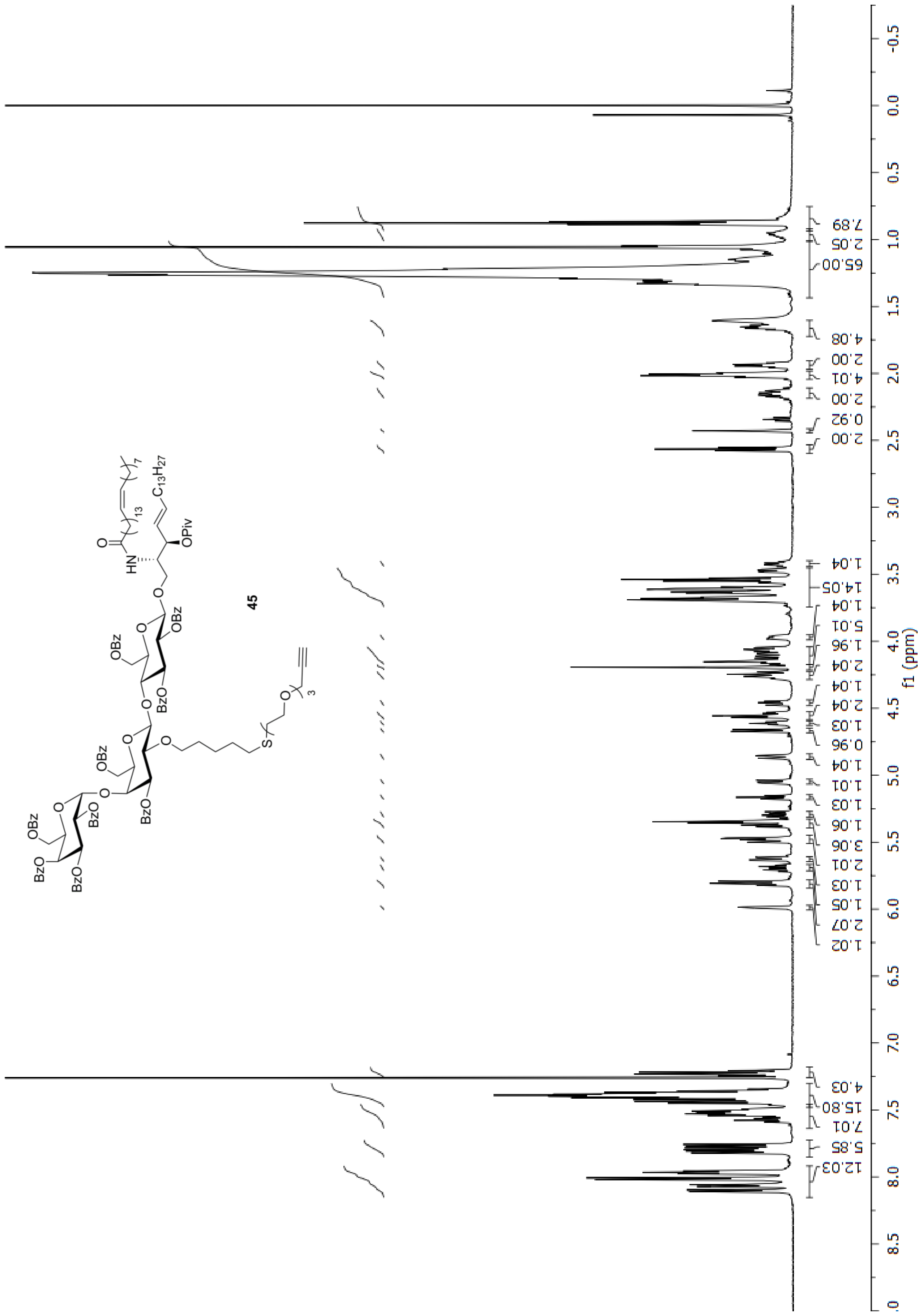
45
f1 (ppm)

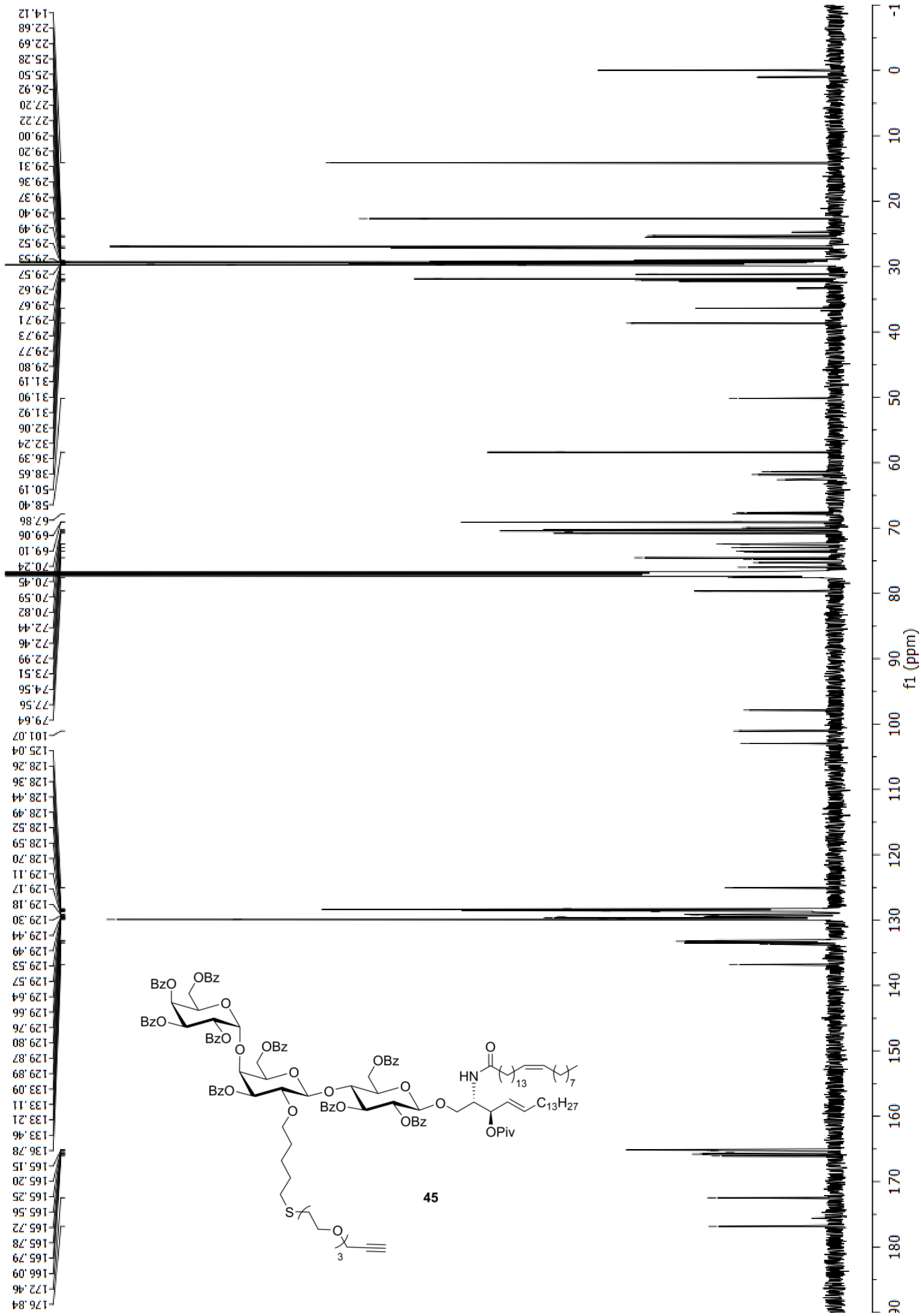
14.12
22.68
22.69
25.28
25.50
26.92
27.20
27.22
29.00
29.20
29.31
29.36
29.37
29.40
29.49
29.52
29.53
29.57
29.62
29.67
29.71
29.73
29.77
29.80
31.19
31.90
31.92
32.06
32.24
36.39
38.65
50.19
58.40
67.86
69.06
69.10
70.24
70.45
70.59
70.82
72.44
72.46
72.99
73.51
74.56
77.56
79.64
101.07
125.04
128.26
128.36
128.44
128.49
128.52
128.59
128.70
129.11
129.17
129.18
129.30
129.44
129.49
129.53
129.57
129.64
129.66
129.76
129.80
129.87
129.89
133.09
133.11
133.21
133.46
136.78
165.15
165.20
165.25
165.56
165.72
165.78
165.79
166.09
172.46
176.84
f1 (ppm)
BzO
OBz
OPiv
HN
$C_{13}H_{27}$

45

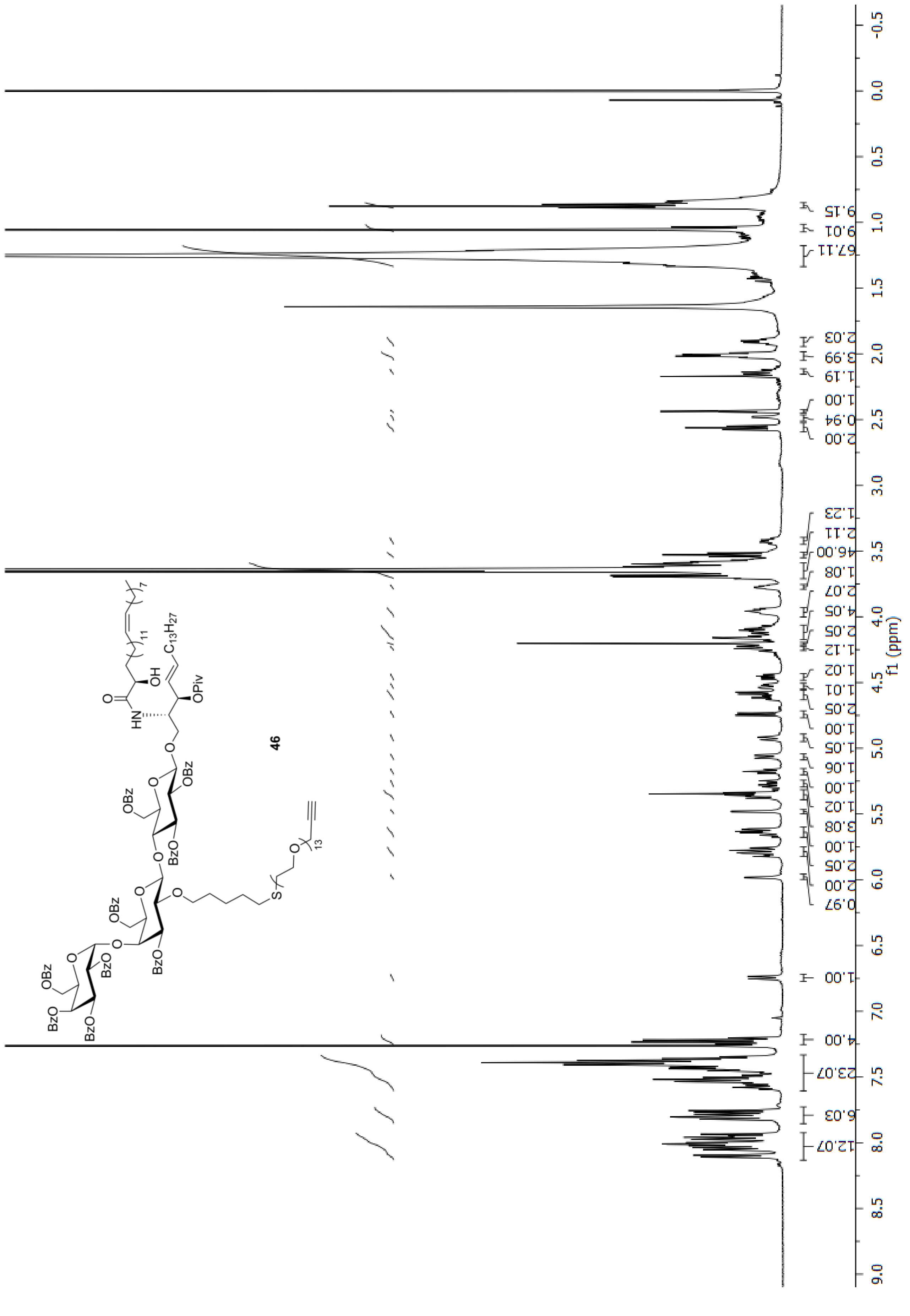
46
BzO
OBz
BzO
BzO
OBz
BzO
BzO
OBz
OBz
O
HN
OH
OPiv
$C_{13}H_{27}$
11
7
S
13
9.15
9.01
67.11
2.03
3.99
1.19
1.00
0.94
2.00
1.23
2.11
46.00
1.08
1.08
2.07
4.05
2.05
1.12
1.02
1.01
2.05
1.00
1.05
1.06
1.00
1.02
3.08
1.00
2.05
2.00
0.97
1.00
4.00
23.07
6.03
12.07
-0.5
0.0
0.5
1.0
1.5
2.0
2.5
3.0
3.5
4.0
4.5
5.0
5.5
6.0
6.5
7.0
7.5
8.0
8.5
9.0
f1 (ppm)

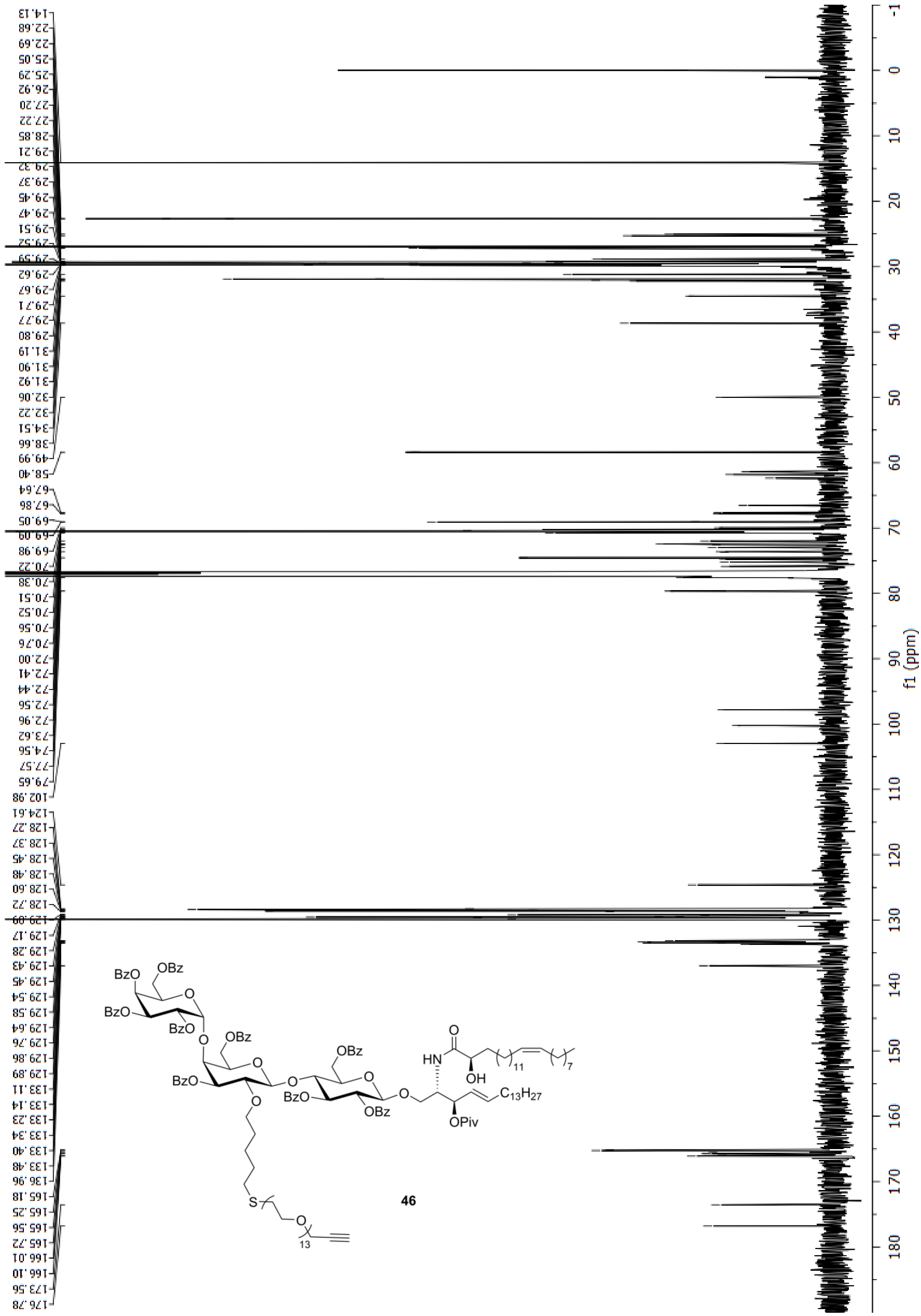
14.13
22.68
22.69
25.05
25.29
26.92
27.20
27.22
28.85
29.21
29.32
29.37
29.45
29.47
29.51
29.52
29.59
29.62
29.67
29.71
29.77
29.80
31.19
31.90
31.92
32.06
32.22
34.51
38.66
49.99
58.40
67.64
67.86
69.05
69.09
69.98
70.22
70.38
70.51
70.52
70.56
70.76
72.00
72.41
72.44
72.56
72.96
73.62
74.56
77.57
79.65
102.98
124.61
128.27
128.37
128.45
128.48
128.60
128.72
129.09
129.17
129.28
129.43
129.45
129.54
129.58
129.64
129.76
129.86
129.89
133.11
133.14
133.23
133.34
133.40
133.48
136.96
165.18
165.25
165.56
165.72
166.01
166.10
173.56
176.78
f1 (ppm)
BzO
OBz
BzO
BzO
O
OBz
BzO
OBz
BzO
OBz
HN
OH
C13H27
OPiv
S
13
46

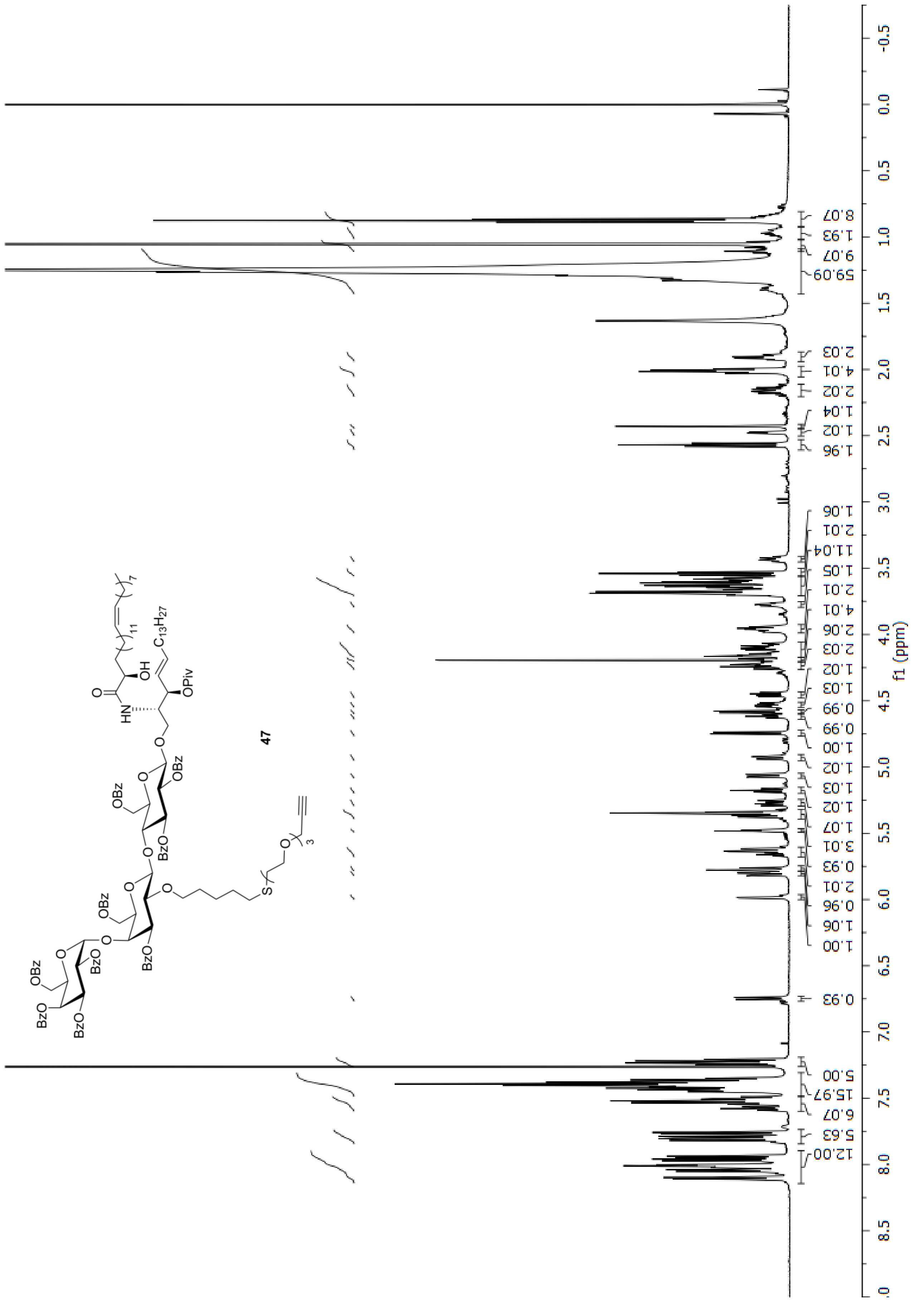
47
f1 (ppm)

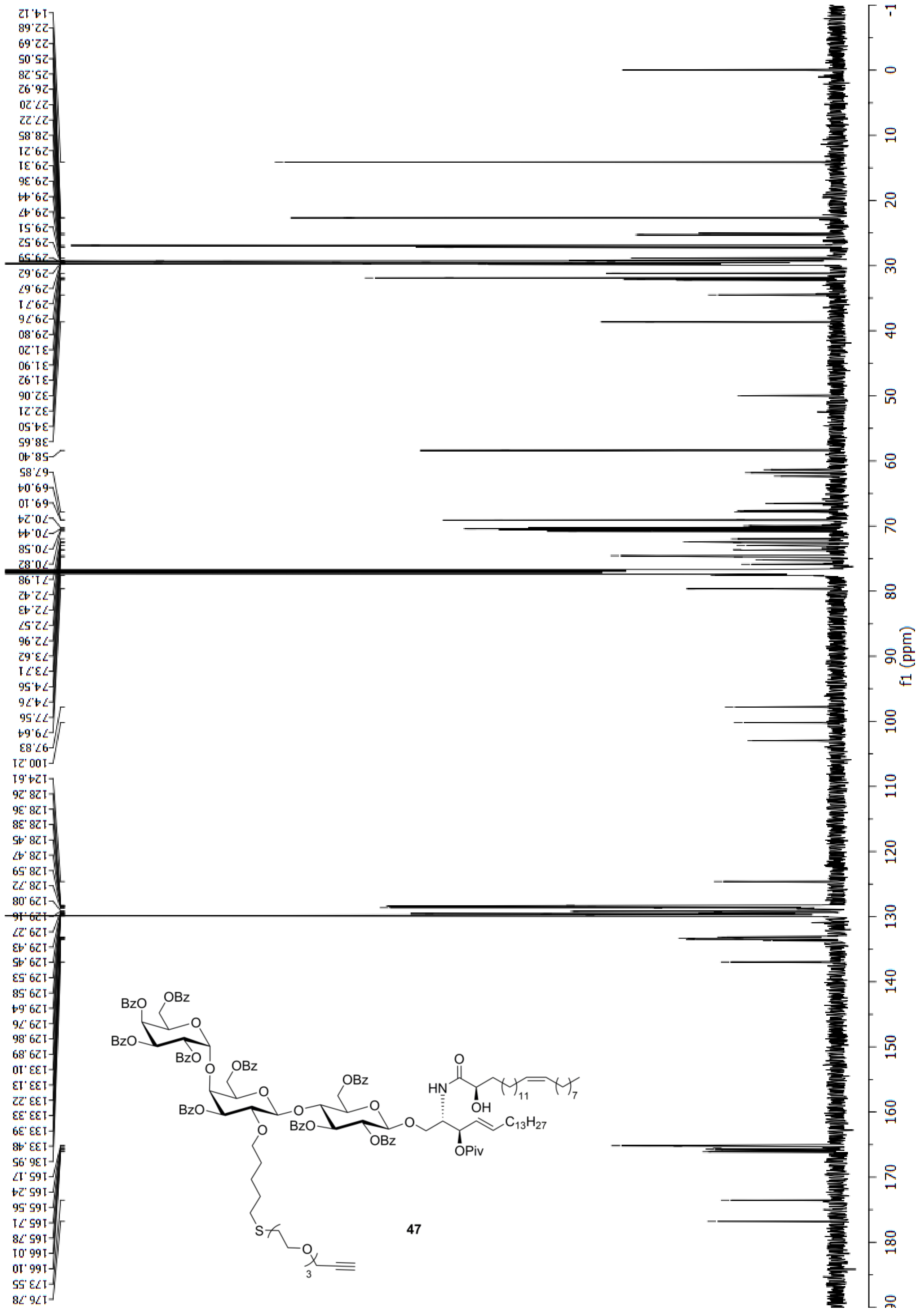
14.12
22.68
22.69
25.05
25.28
26.92
27.20
27.22
28.85
29.21
29.31
29.36
29.44
29.47
29.51
29.52
29.59
29.62
29.67
29.71
29.76
29.80
31.20
31.90
31.92
32.06
32.21
34.50
38.65
58.40
67.85
69.04
69.10
70.24
70.44
70.58
70.82
71.98
72.42
72.43
72.57
72.96
73.62
73.71
74.56
74.76
77.56
79.64
97.83
100.21
124.61
128.26
128.36
128.38
128.45
128.47
128.59
128.72
129.08
129.16
129.27
129.43
129.45
129.53
129.58
129.64
129.76
129.86
129.89
133.10
133.13
133.22
133.33
133.39
133.48
136.95
165.17
165.24
165.56
165.71
165.78
166.01
166.10
173.55
176.78
f1 (ppm)
BzO
OBz
O
BzO
BzO
O
OBz
O
BzO
O
O
OBz
O
BzO
OBz
O
HN
O
OH
11
7
C13H27
OPiv
S
O
3

47

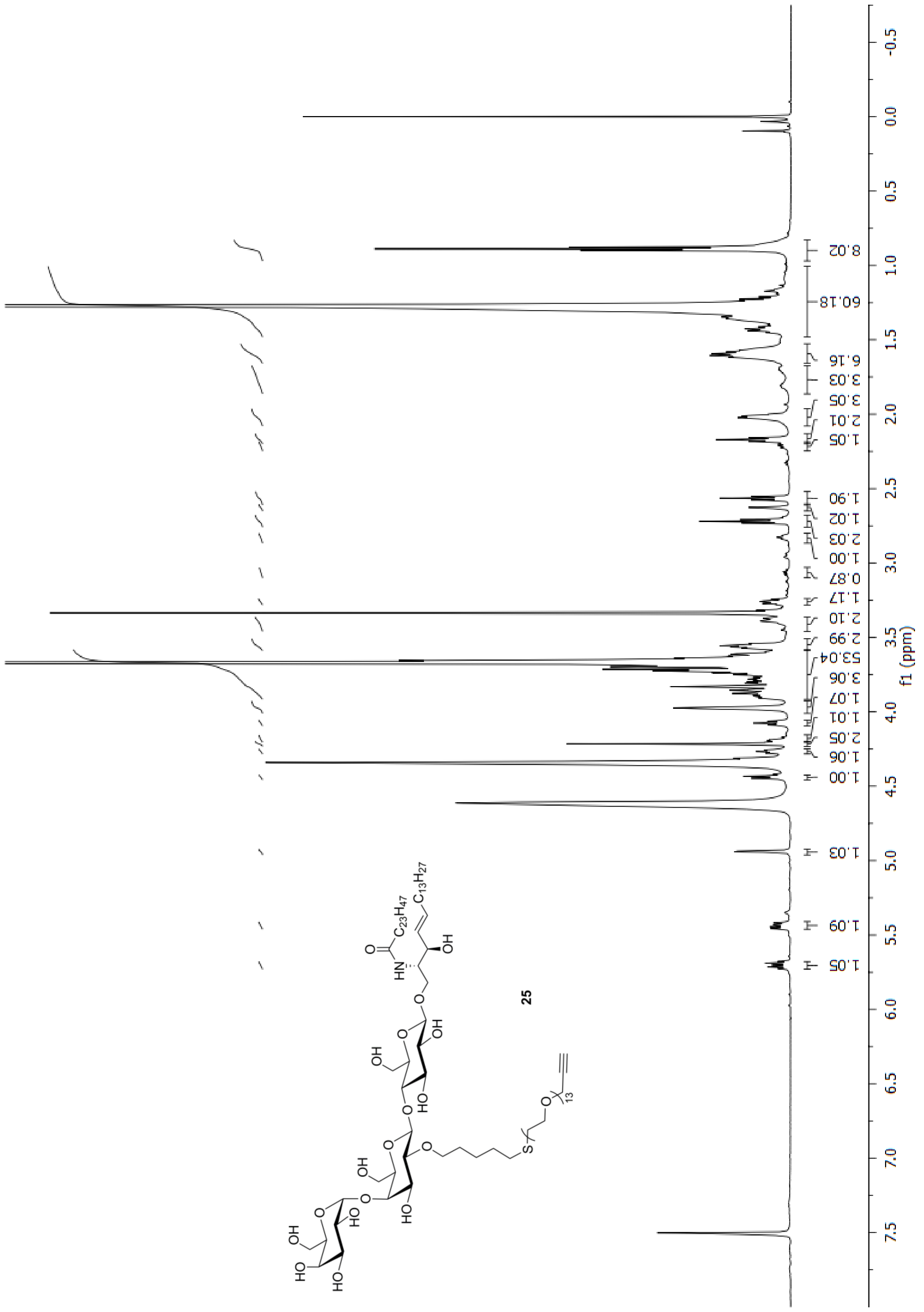
25
f1 (ppm)

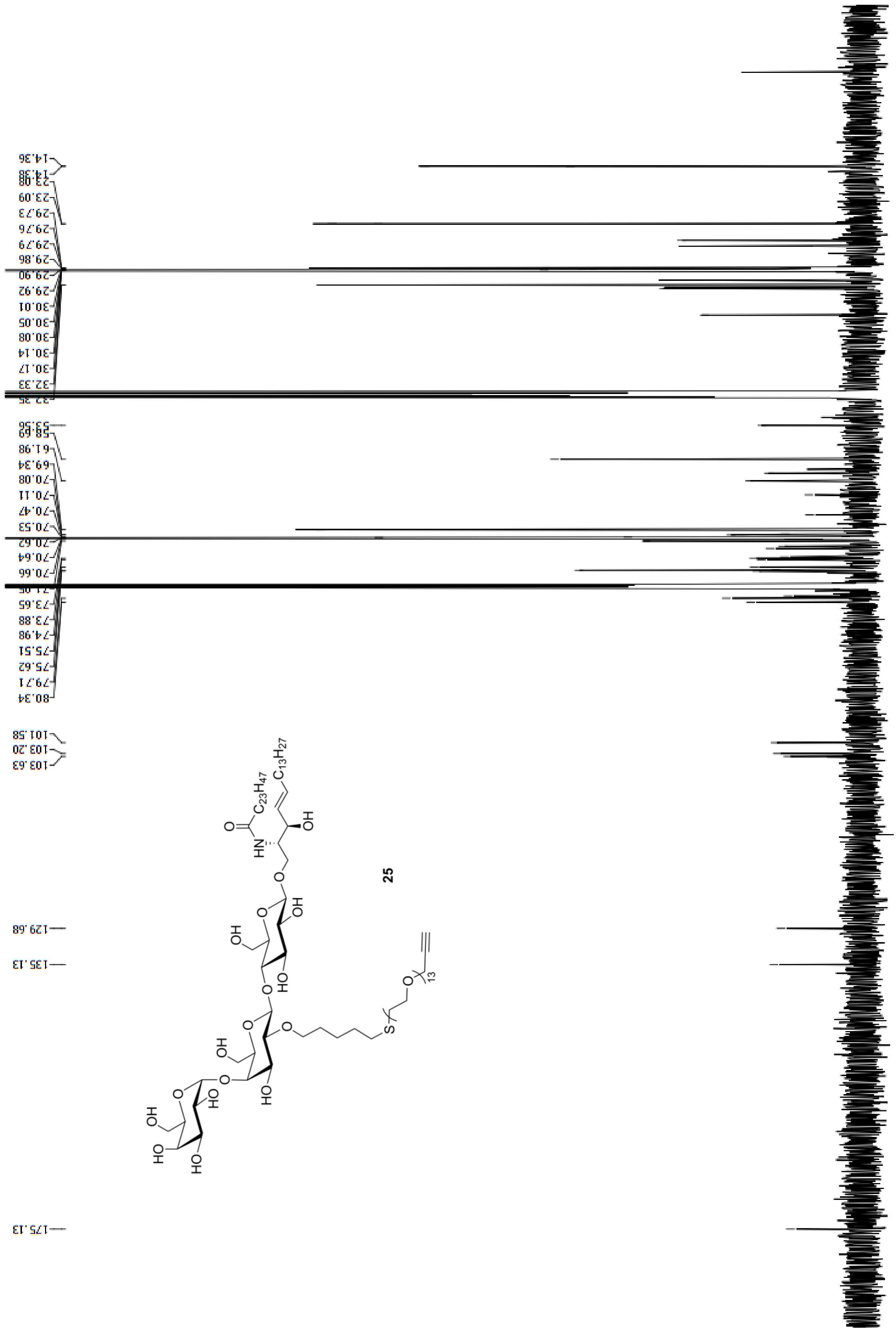
25

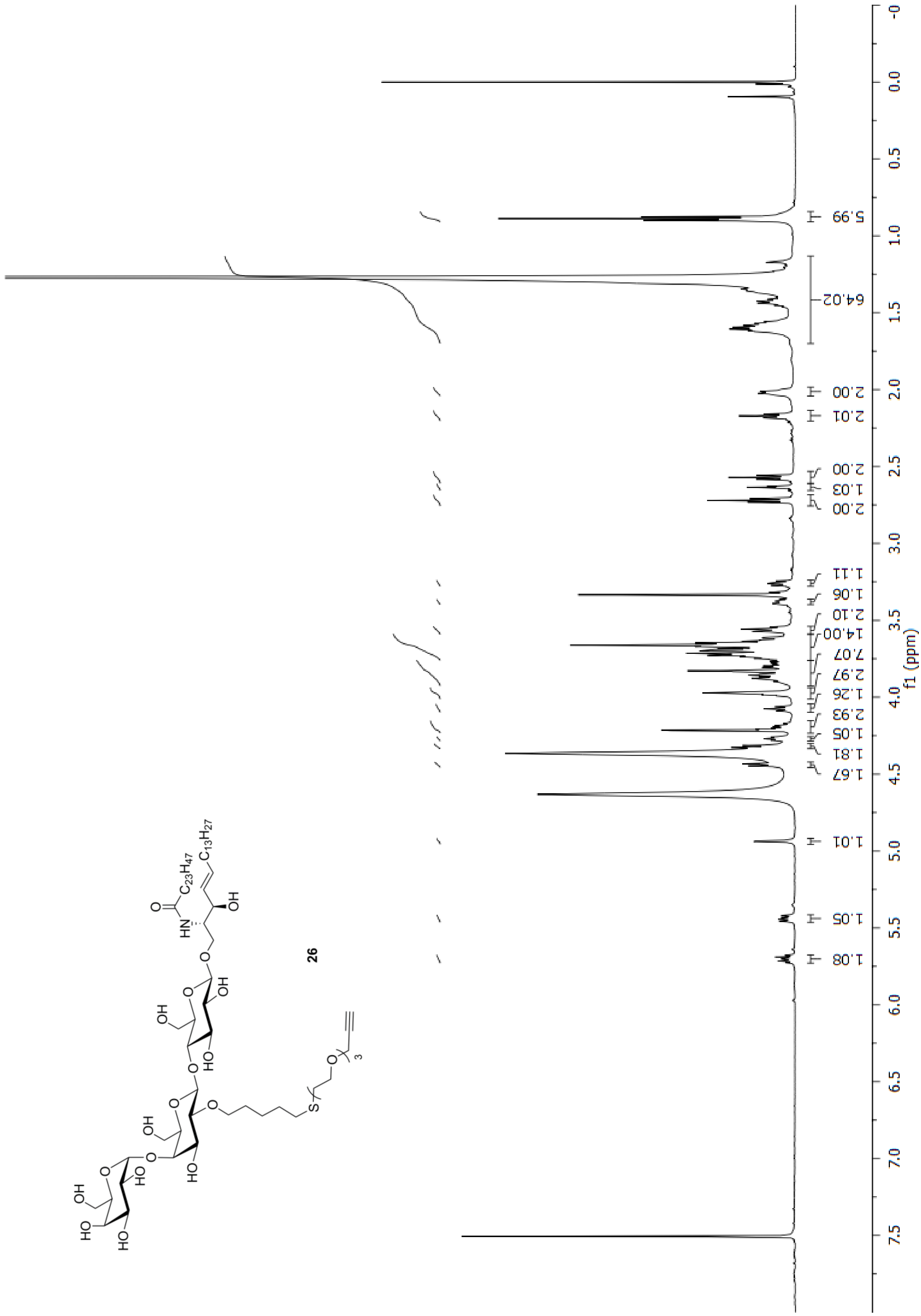
26
f1 (ppm)

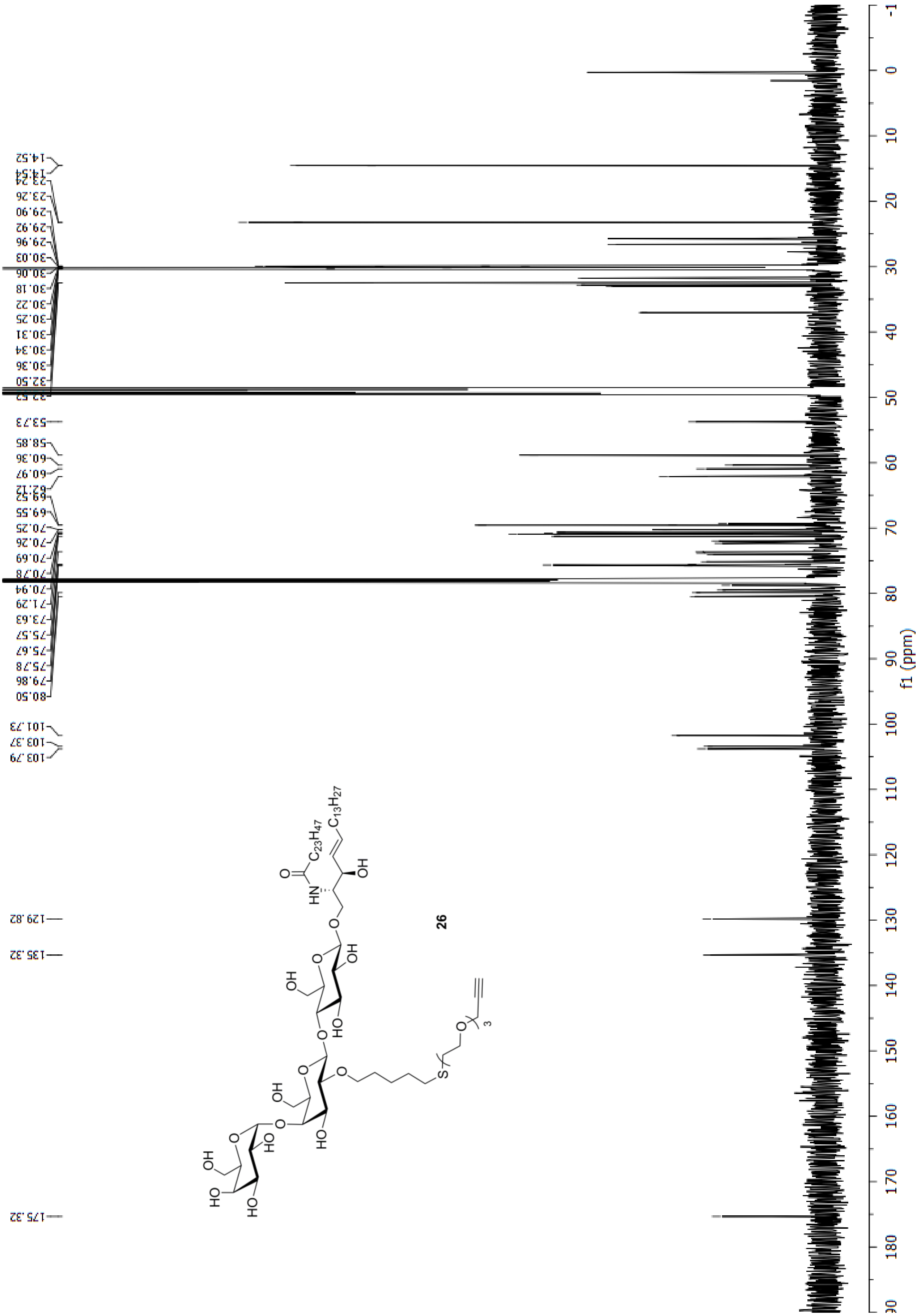
14.52
14.54
23.24
23.26
29.90
29.92
29.96
30.03
30.06
30.18
30.22
30.25
30.31
30.34
30.36
32.50
53.73
58.85
60.36
60.97
62.12
69.52
69.55
70.25
70.26
70.69
70.78
70.94
71.29
73.63
75.57
75.67
75.78
79.86
80.50
101.73
103.37
103.79
129.82
135.32
175.32
f1 (ppm)

26

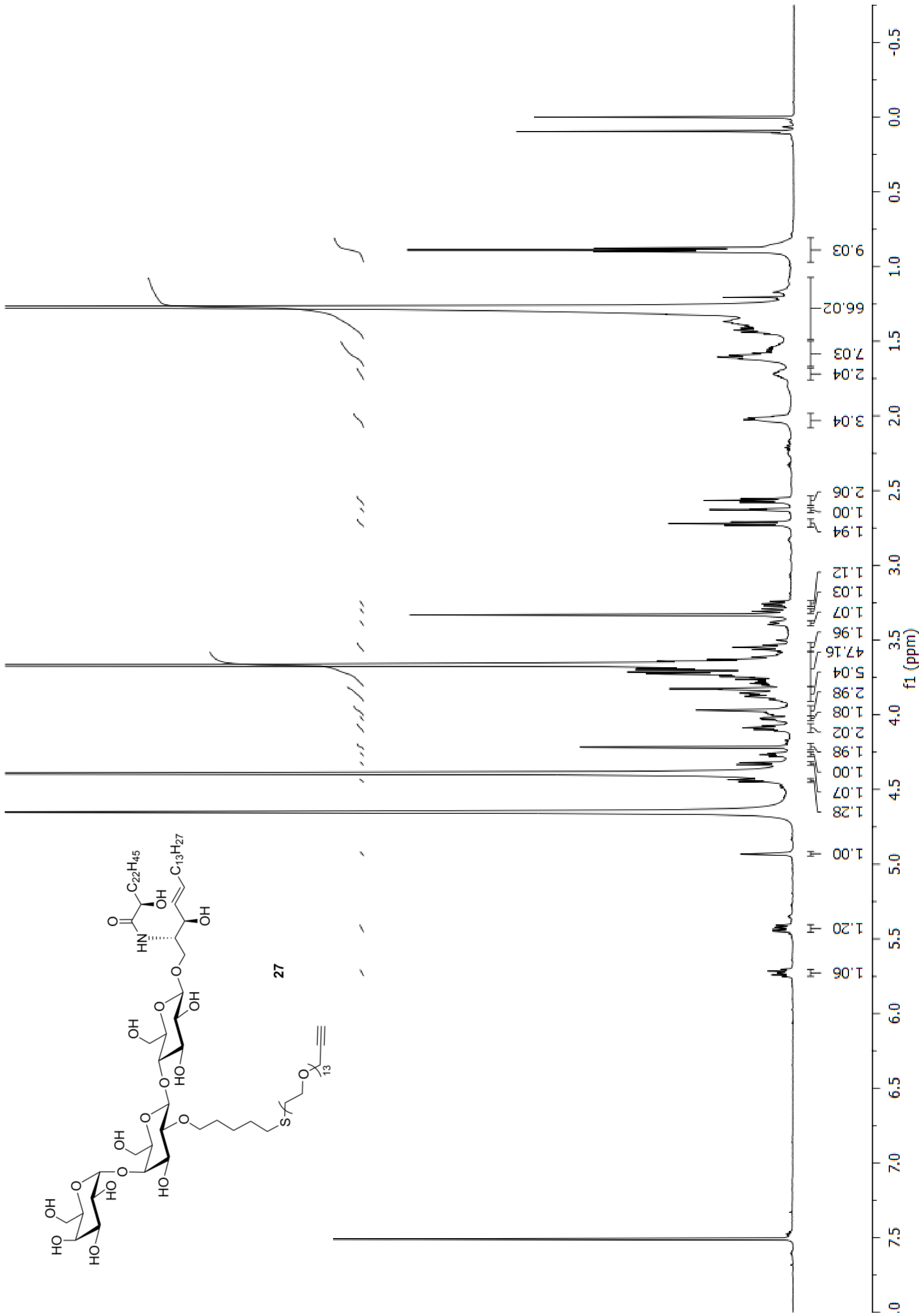

27

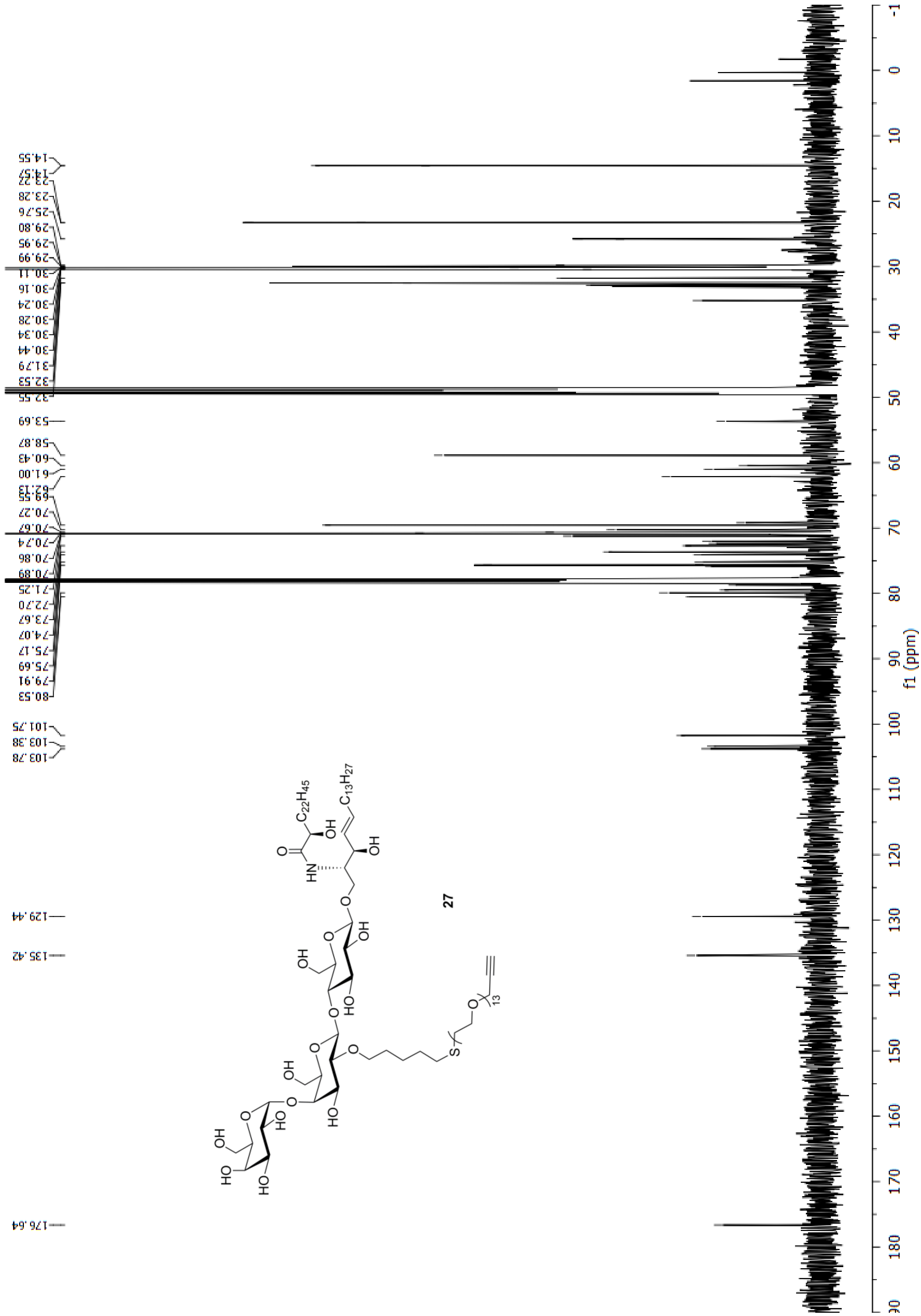
14.55
14.57
23.22
23.28
25.76
29.80
29.95
29.99
30.11
30.16
30.24
30.28
30.34
30.44
31.79
32.53
32.55
53.69
58.87
60.43
61.00
62.13
69.55
70.27
70.67
70.74
70.86
70.89
71.25
72.70
73.67
74.07
75.17
75.69
79.91
80.53
101.75
103.38
103.78
129.44
135.42
176.64
27
f1 (ppm)

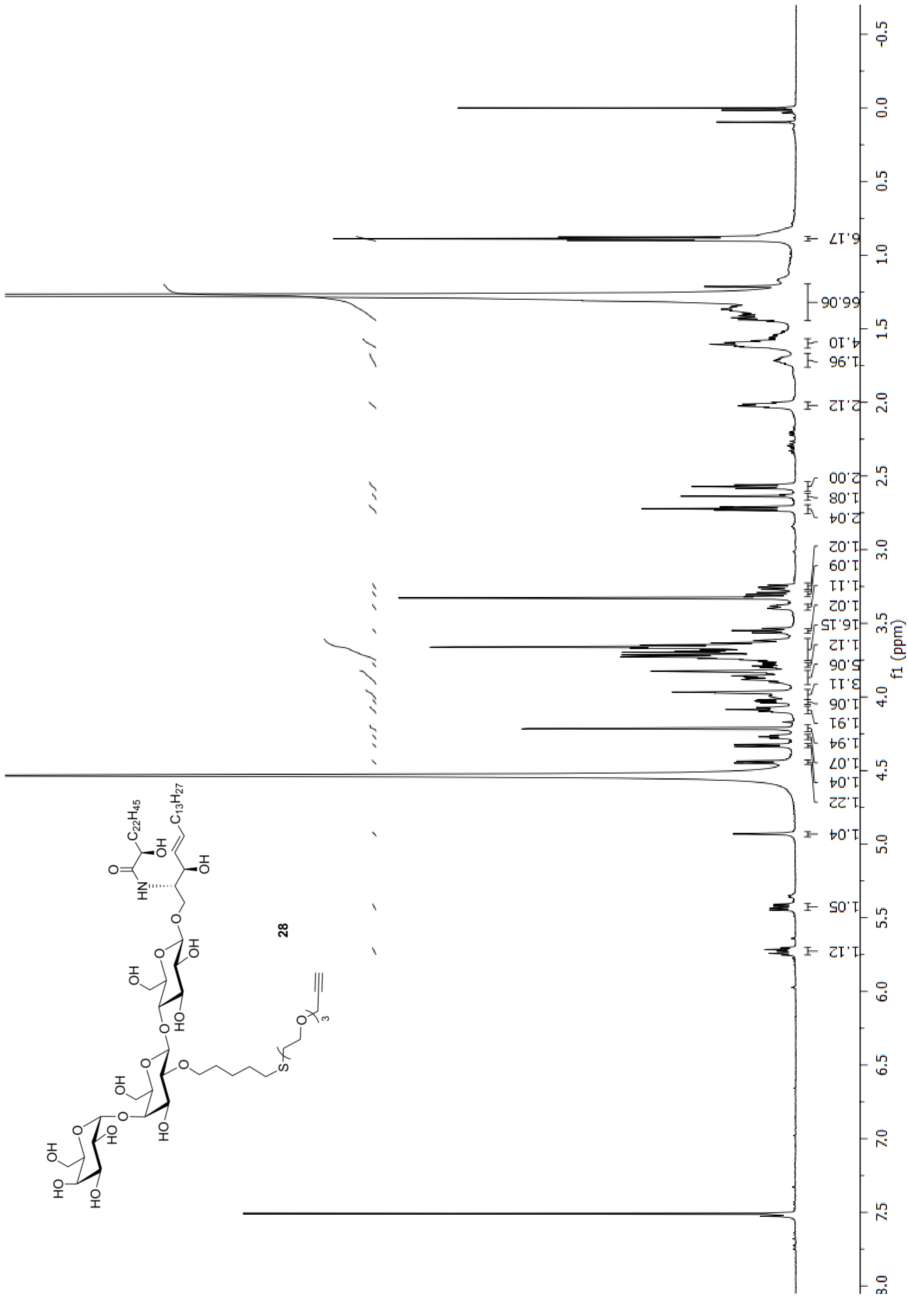
28
f1 (ppm)

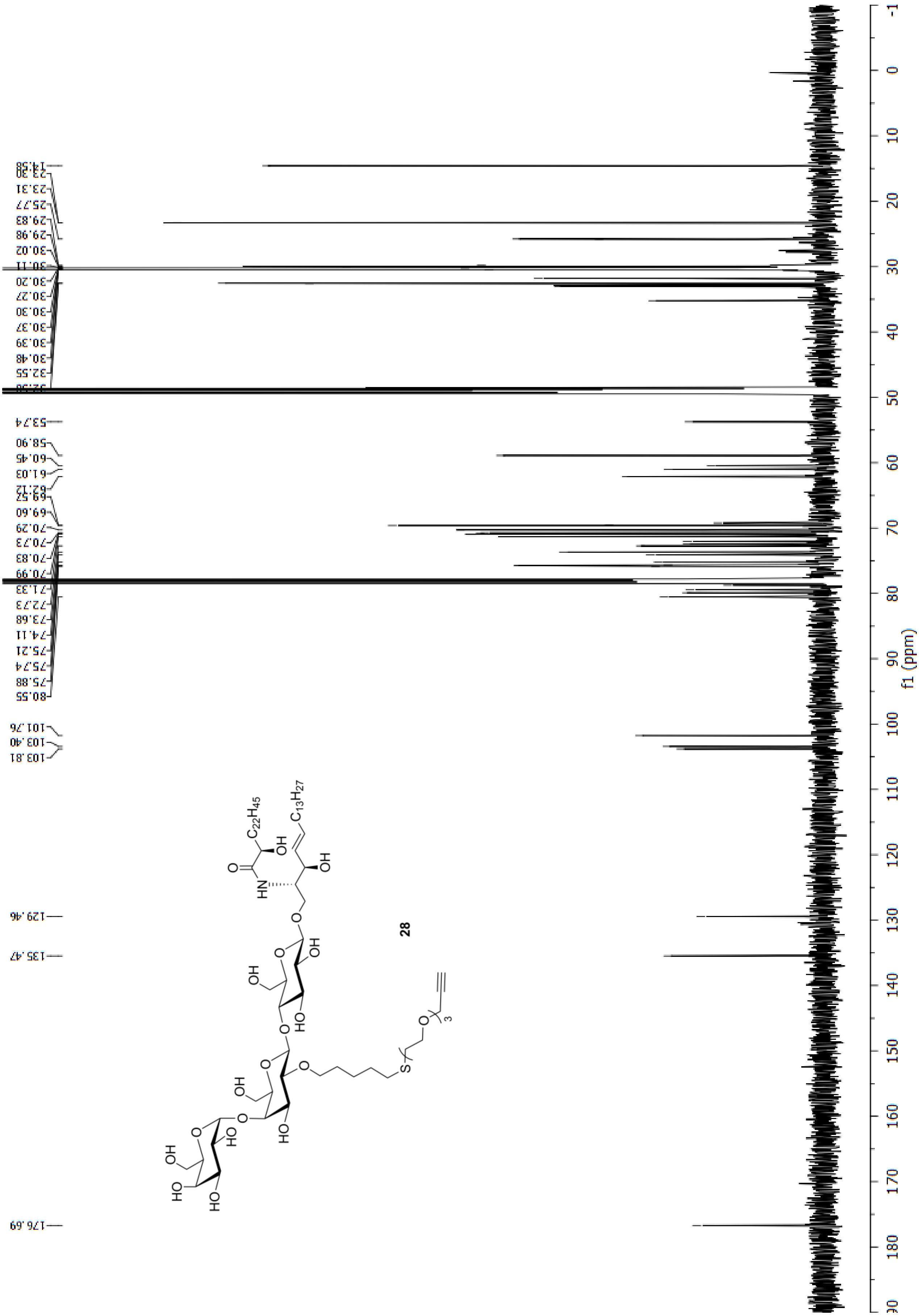

28

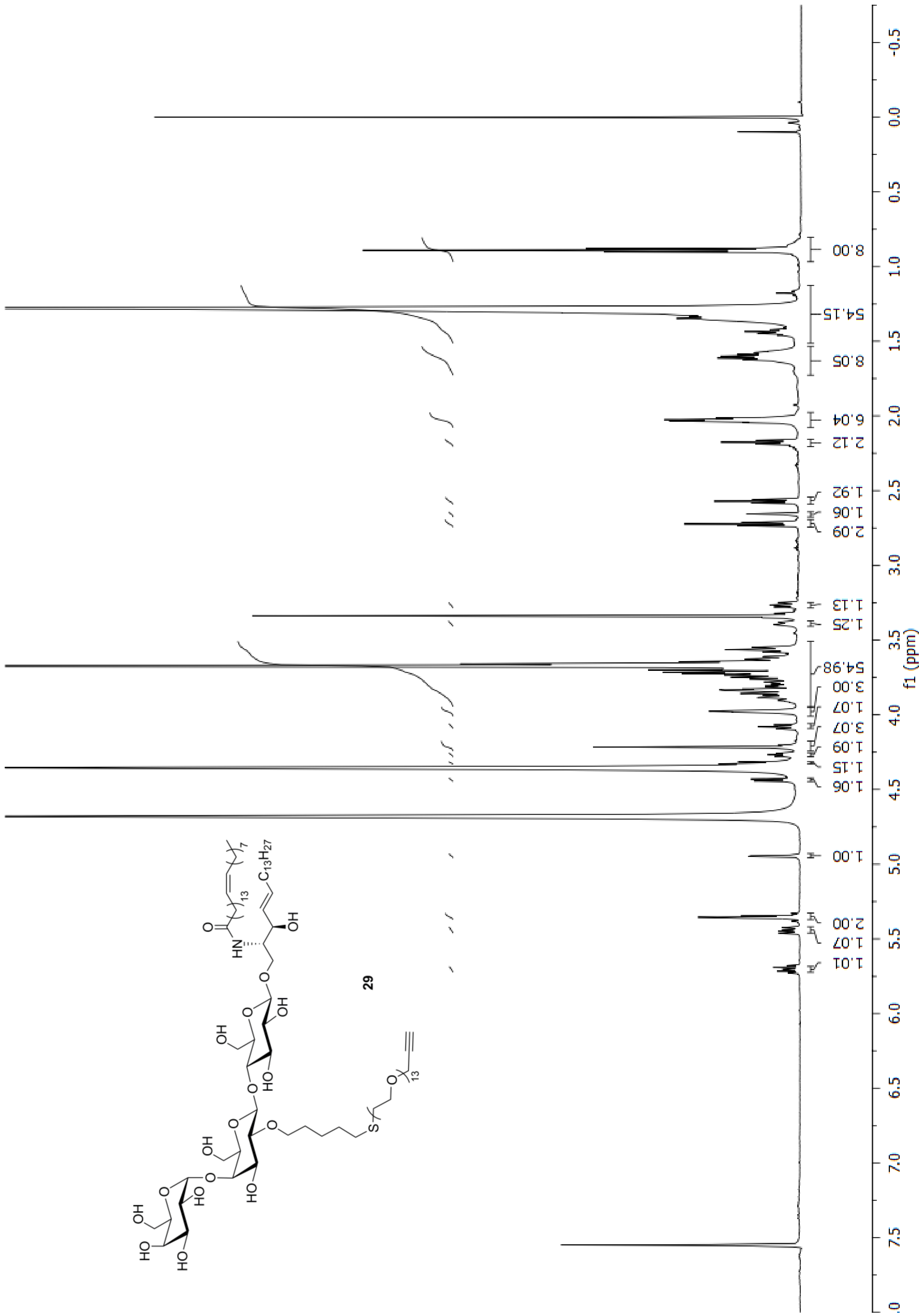
29
f1 (ppm)

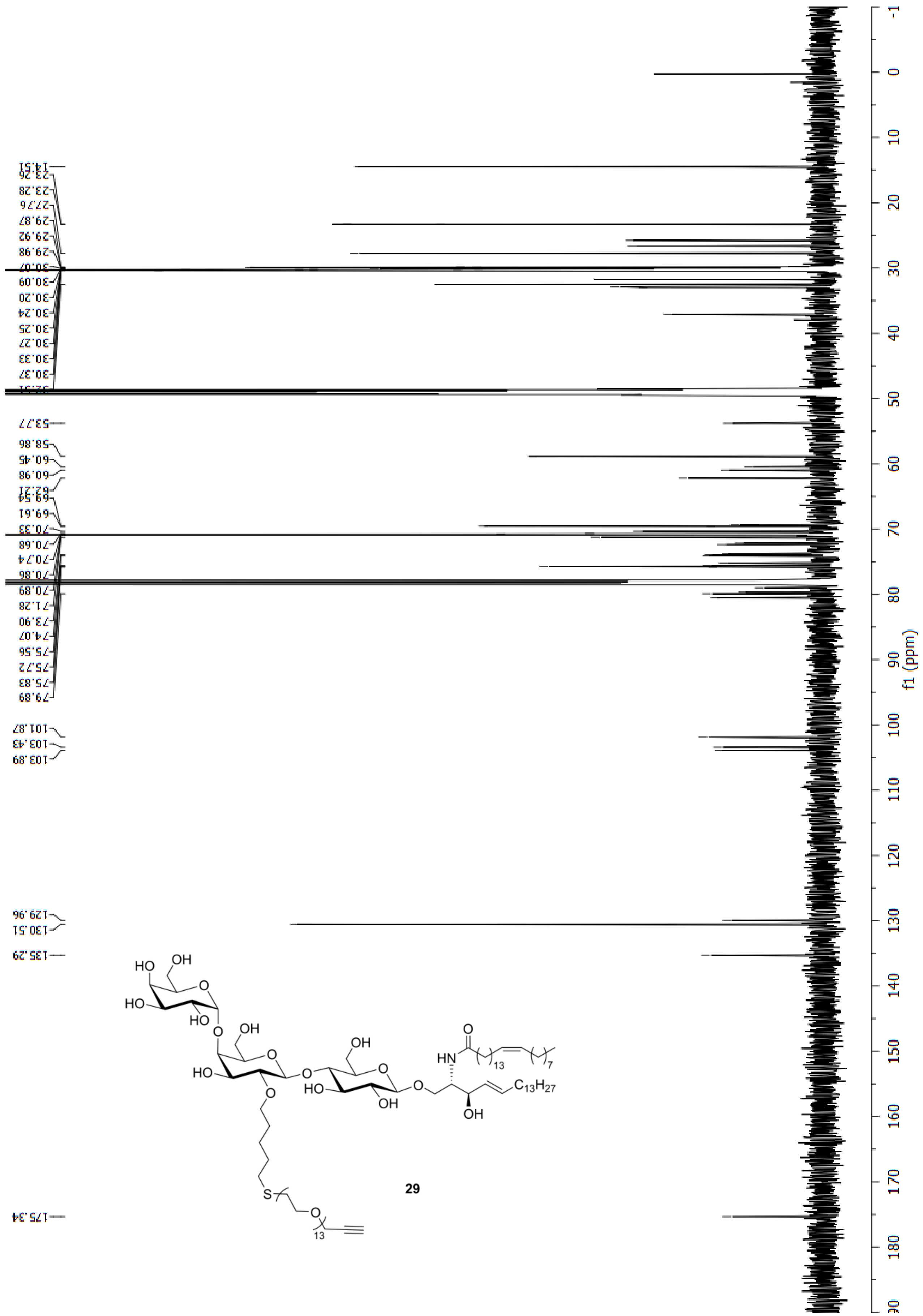
14.51
23.26
23.28
27.76
29.87
29.92
29.98
30.07
30.09
30.20
30.24
30.25
30.27
30.33
30.37
53.77
58.86
60.45
60.98
69.61
70.33
70.68
70.74
70.86
70.89
71.28
73.90
74.07
75.56
75.72
75.83
79.89
101.87
103.43
103.89
129.96
130.51
135.29
175.34
f1 (ppm)
HO
OH
HN
O
$C_{13}H_{27}$
S
13
7
29

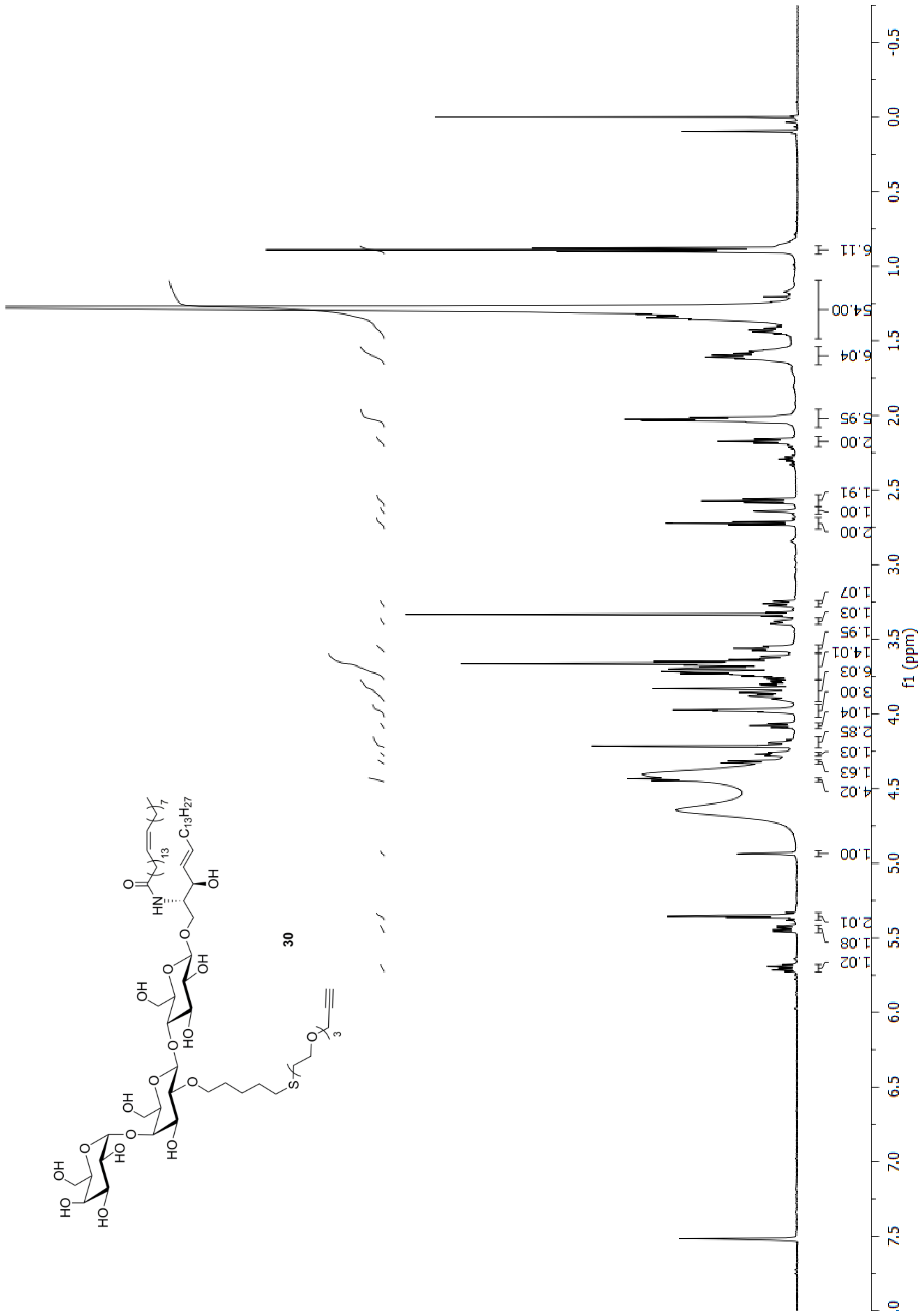

30

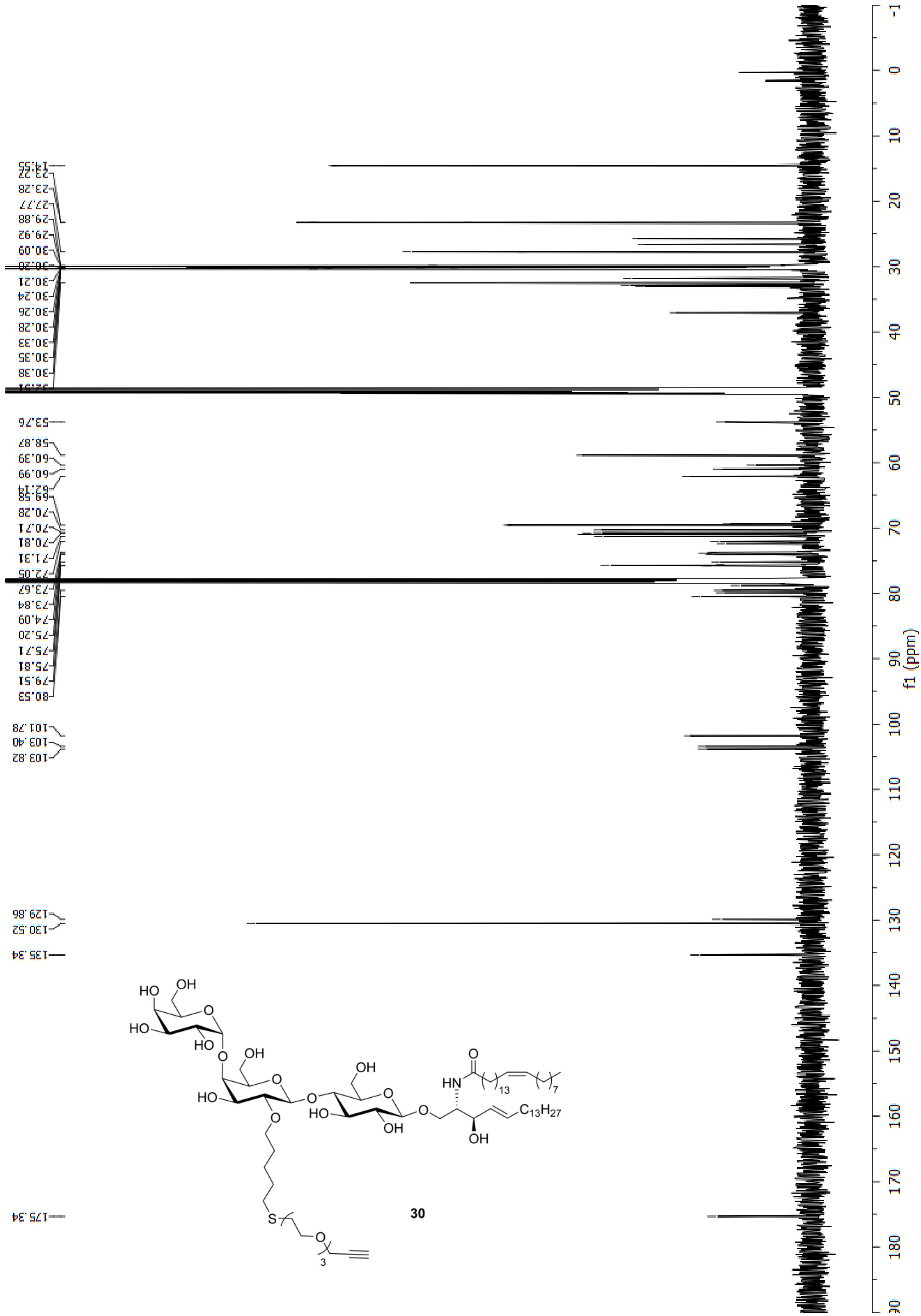
f1 (ppm)
HO
OH
HN
$C_{13}H_{27}$
S
30

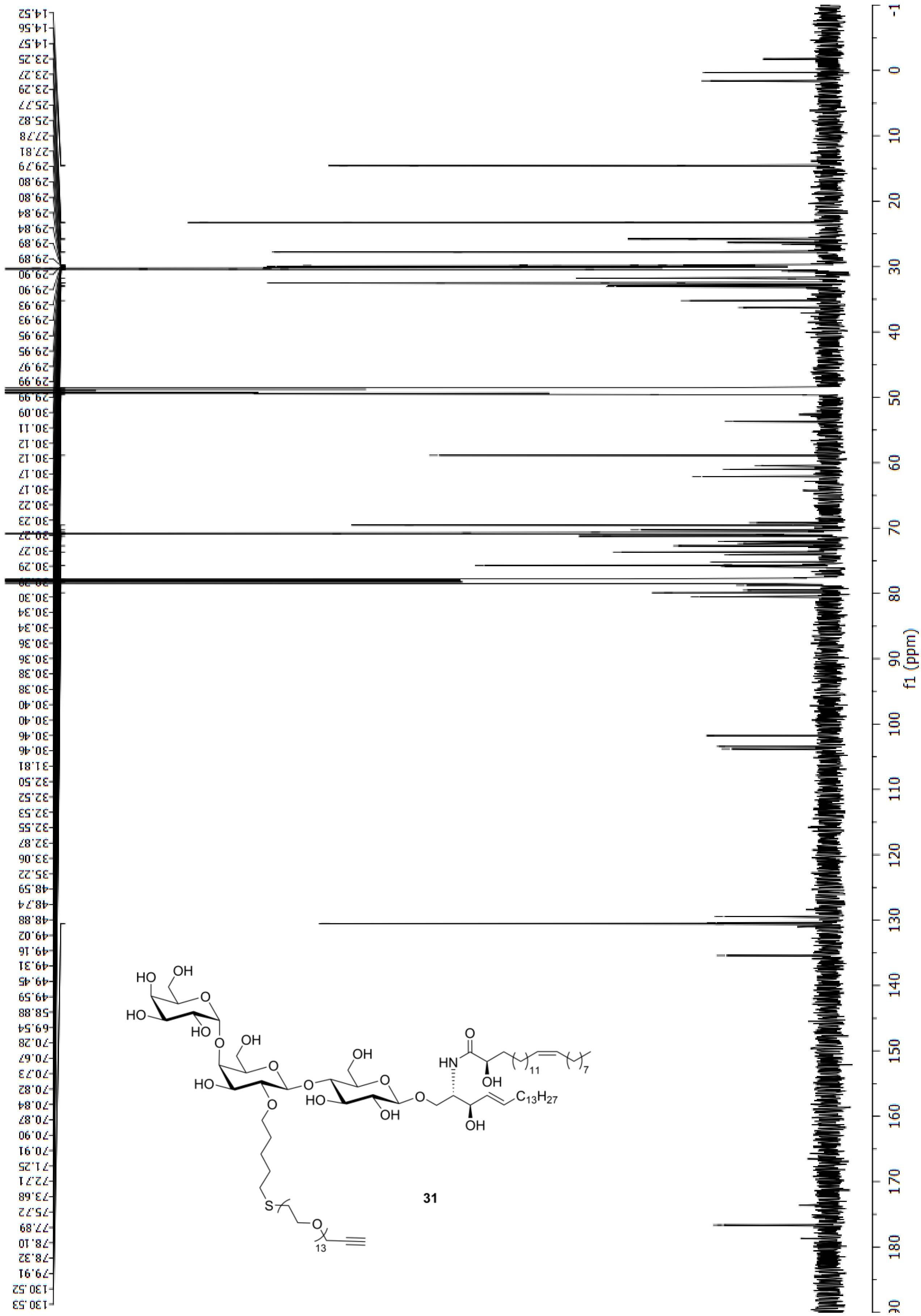
14.52
14.56
14.57
23.25
23.27
23.29
25.77
25.82
27.78
27.81
29.79
29.80
29.80
29.84
29.84
29.89
29.89
29.90
29.90
29.93
29.93
29.95
29.95
29.97
29.99
29.99
30.09
30.11
30.12
30.12
30.17
30.17
30.22
30.23
30.27
30.29
30.30
30.34
30.34
30.36
30.36
30.38
30.38
30.40
30.40
30.46
30.46
31.81
32.50
32.52
32.53
32.55
32.87
33.06
35.22
48.59
48.74
48.88
49.02
49.16
49.31
49.45
49.59
58.88
69.54
70.28
70.67
70.73
70.82
70.84
70.87
70.90
70.91
71.25
72.71
73.68
75.72
77.89
78.10
78.32
79.91
130.52
130.53
f1 (ppm)
HO
OH
HN
O
OH
C13H27
S
13

31

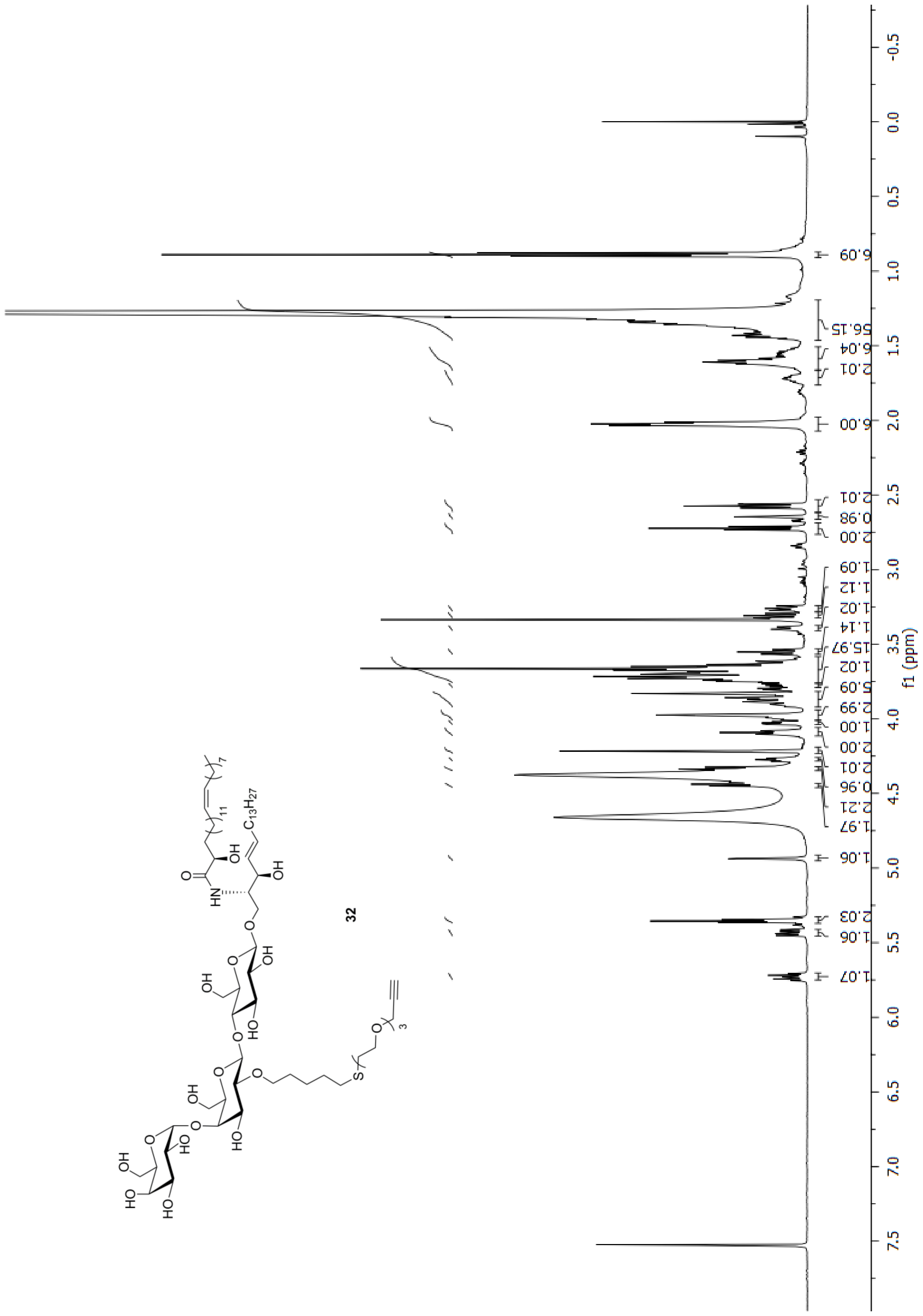
32
f1 (ppm)

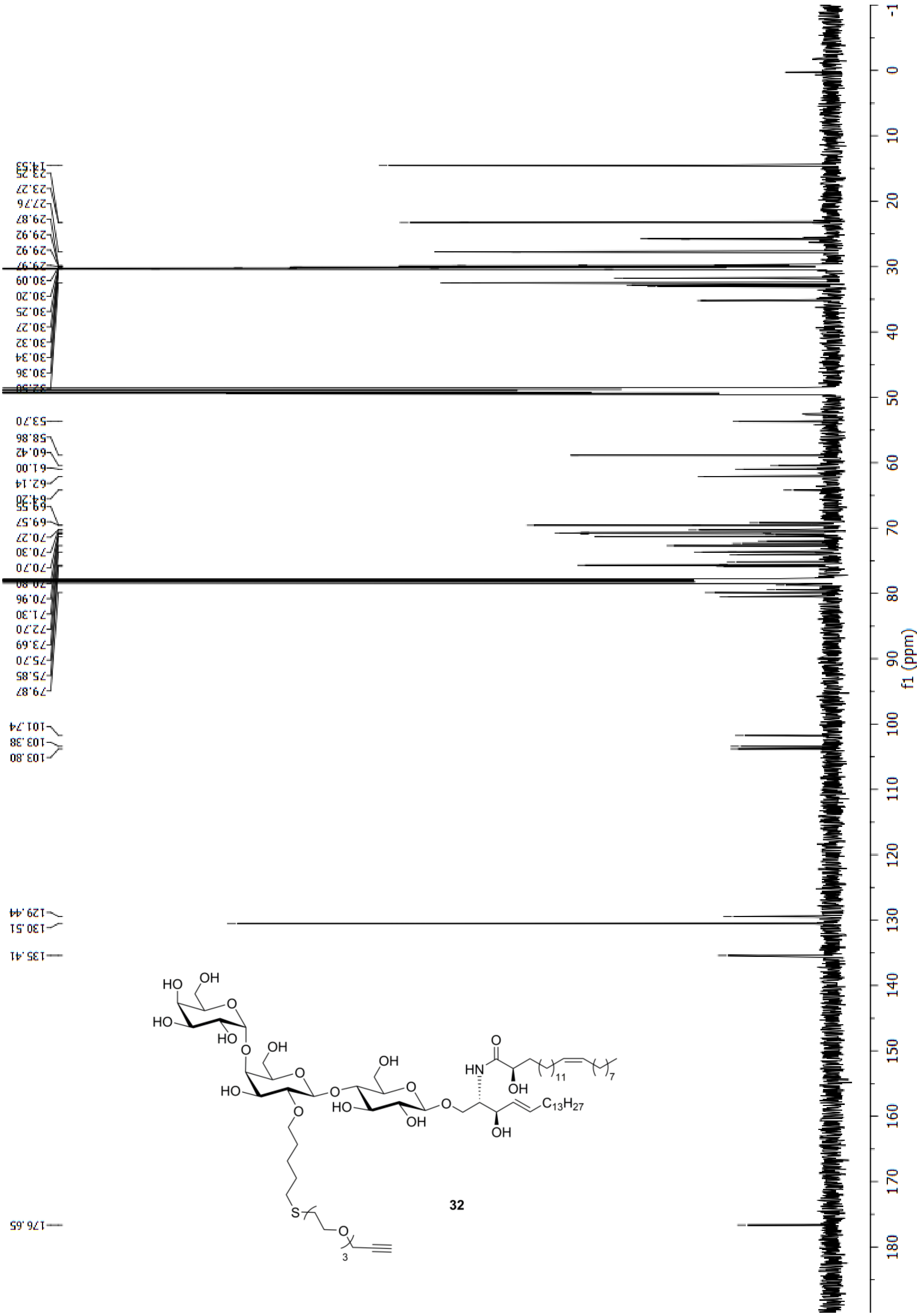
14.53
23.27
27.76
29.87
29.92
29.92
29.97
30.09
30.20
30.25
30.27
30.32
30.34
30.36
53.70
58.86
60.42
61.00
62.14
69.55
69.57
70.27
70.30
70.70
70.96
71.30
72.70
73.69
75.70
75.85
79.87
101.74
103.38
103.80
129.44
130.51
135.41
176.65
f1 (ppm)

32

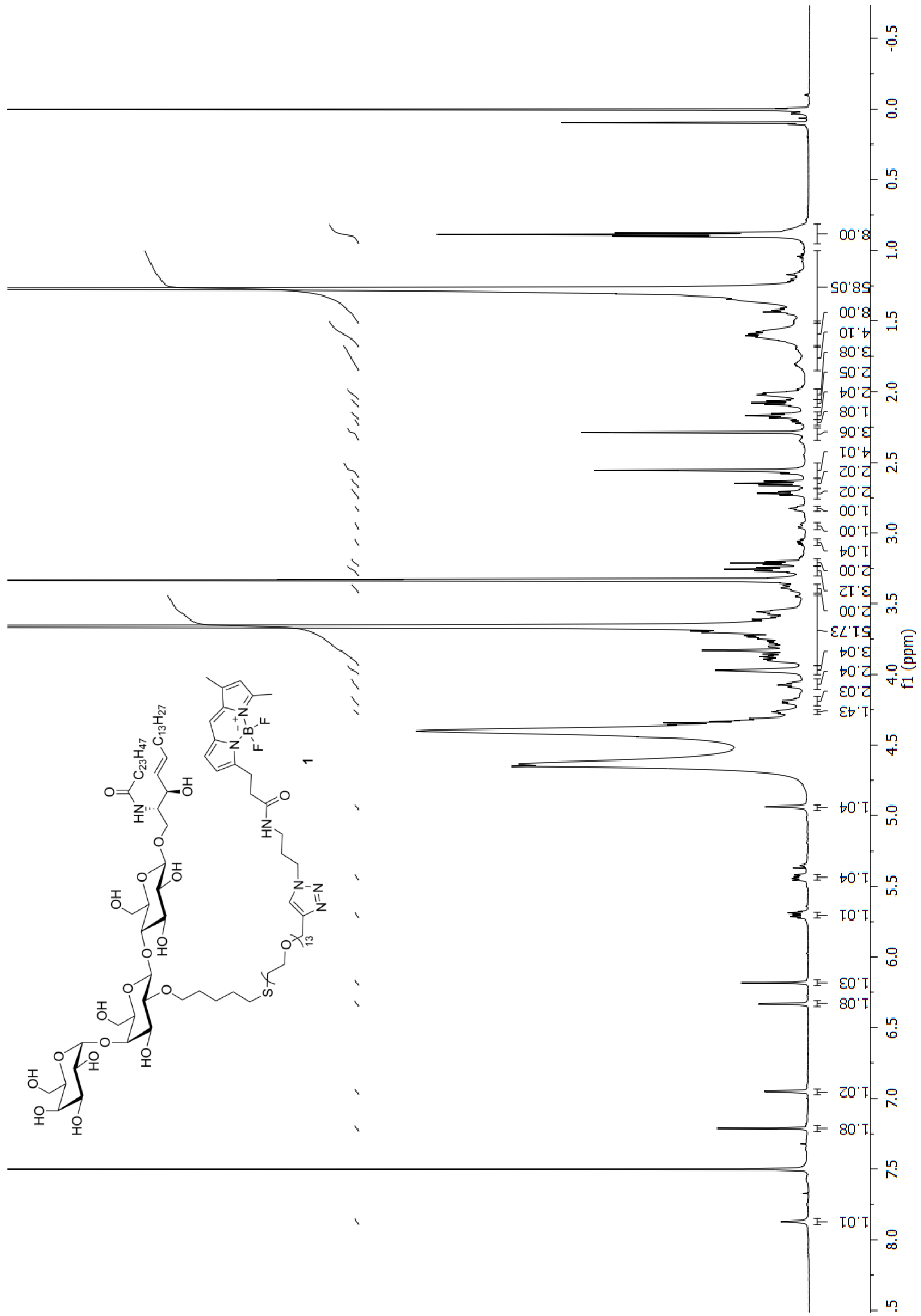
f1 (ppm)
1

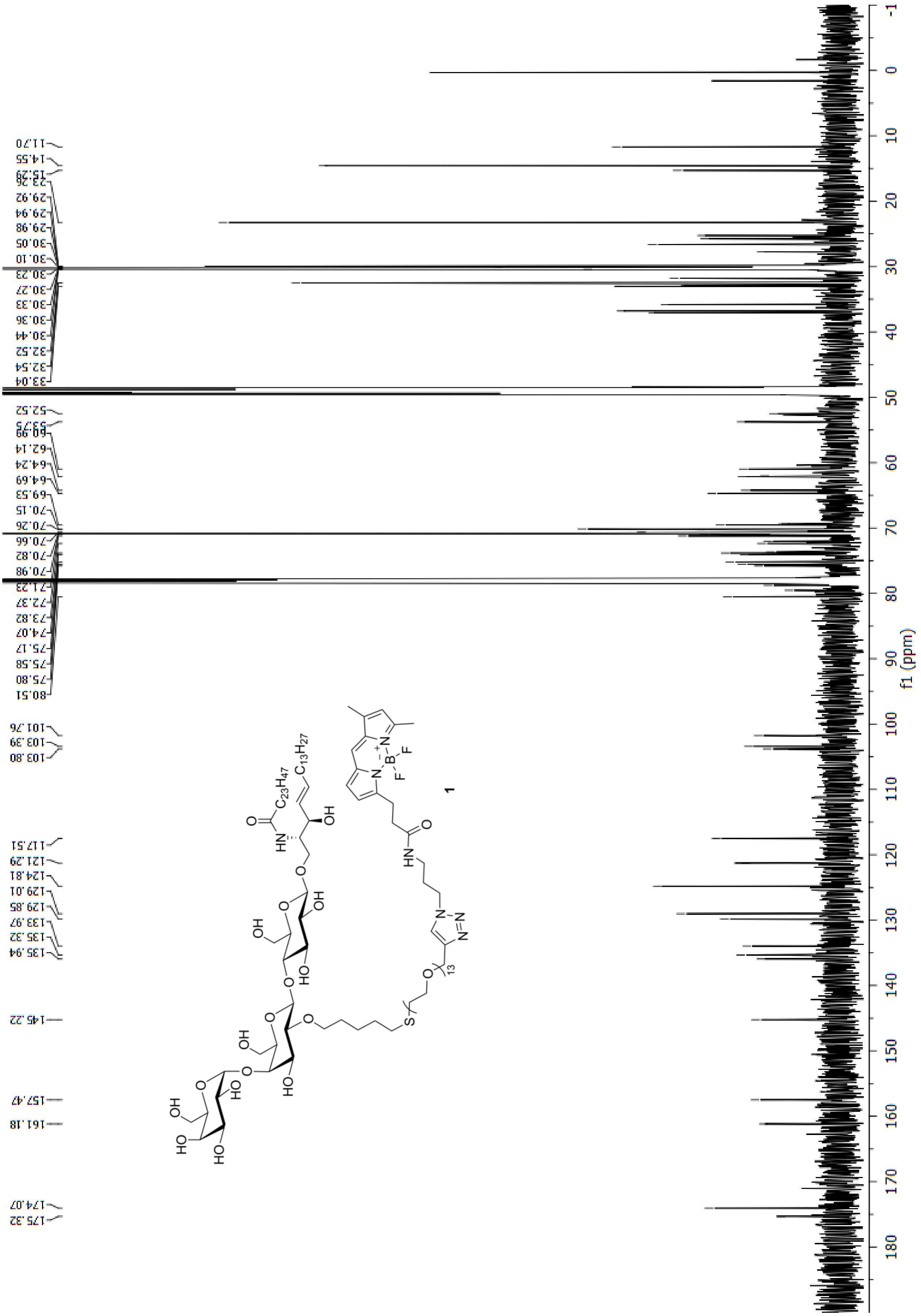
11.70
14.55
15.29
23.36
29.92
29.94
29.98
30.05
30.10
30.23
30.27
30.33
30.36
30.44
32.52
32.54
33.04
52.52
62.14
64.24
64.69
69.53
70.15
70.26
70.66
70.82
70.98
71.23
72.37
73.82
74.07
75.17
75.58
75.80
80.51
101.76
103.39
103.80
117.51
121.29
124.81
129.01
129.85
133.97
135.32
135.94
145.22
157.47
161.18
174.07
175.32
f1 (ppm)
1

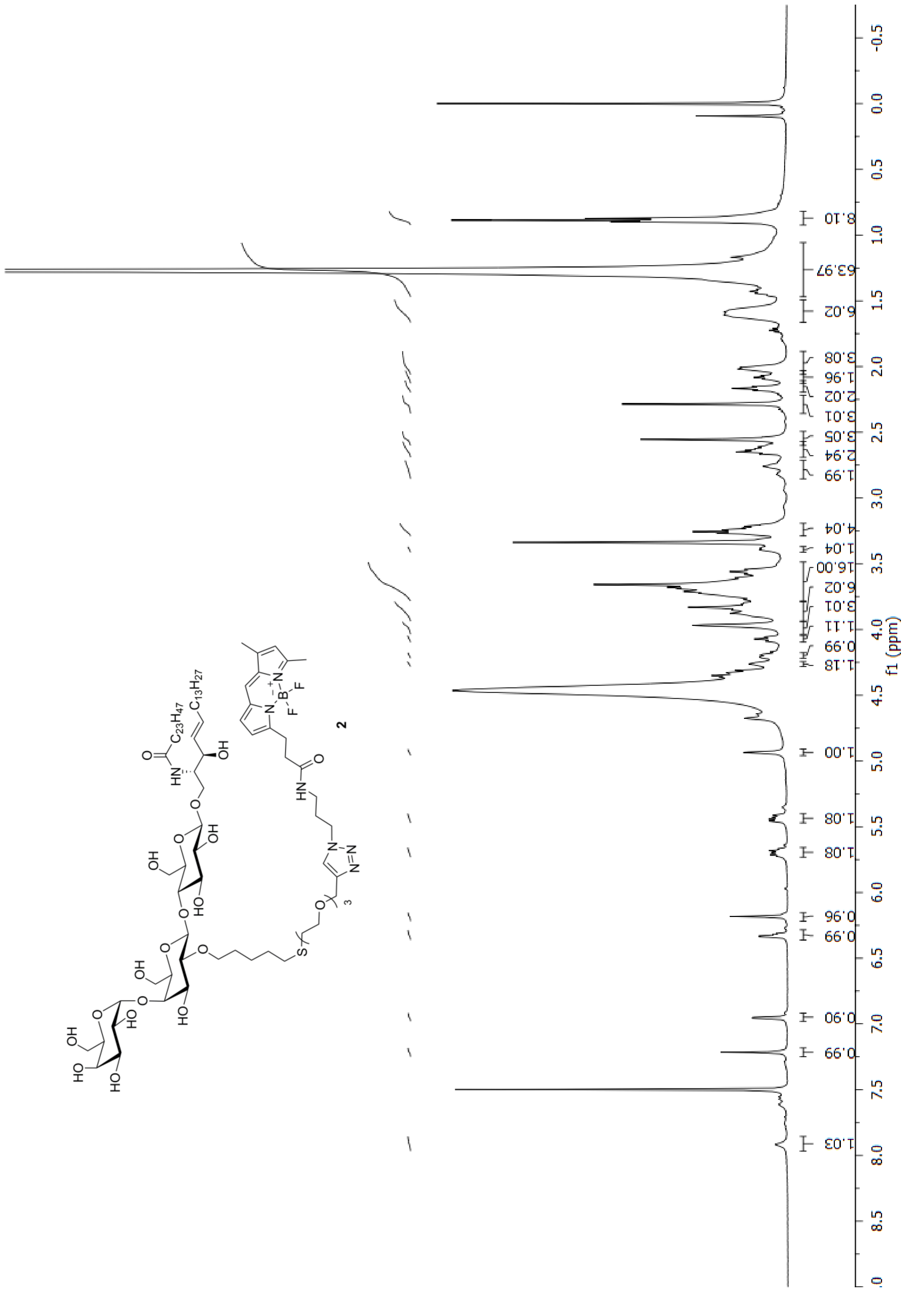
f1 (ppm)
2

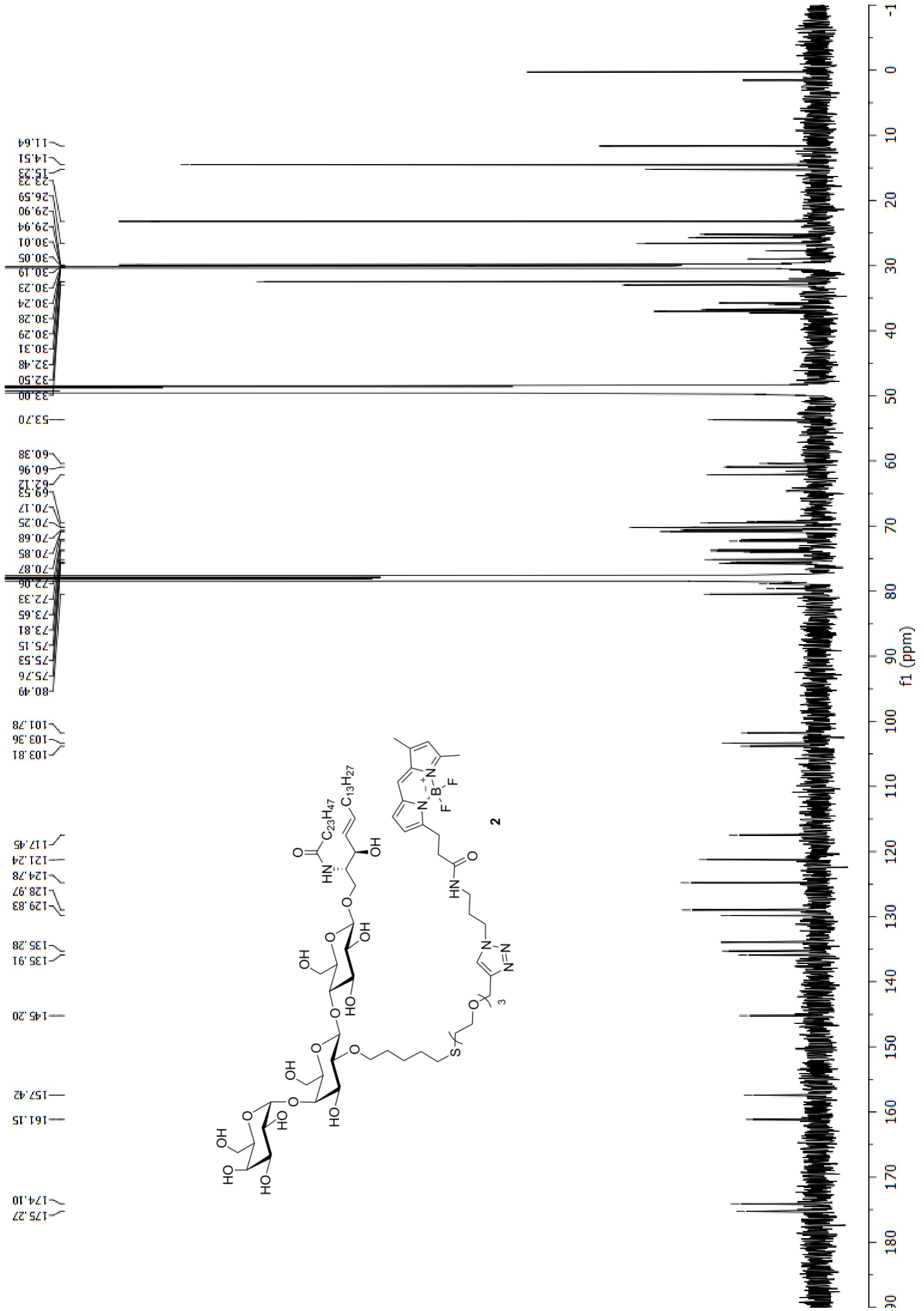

2

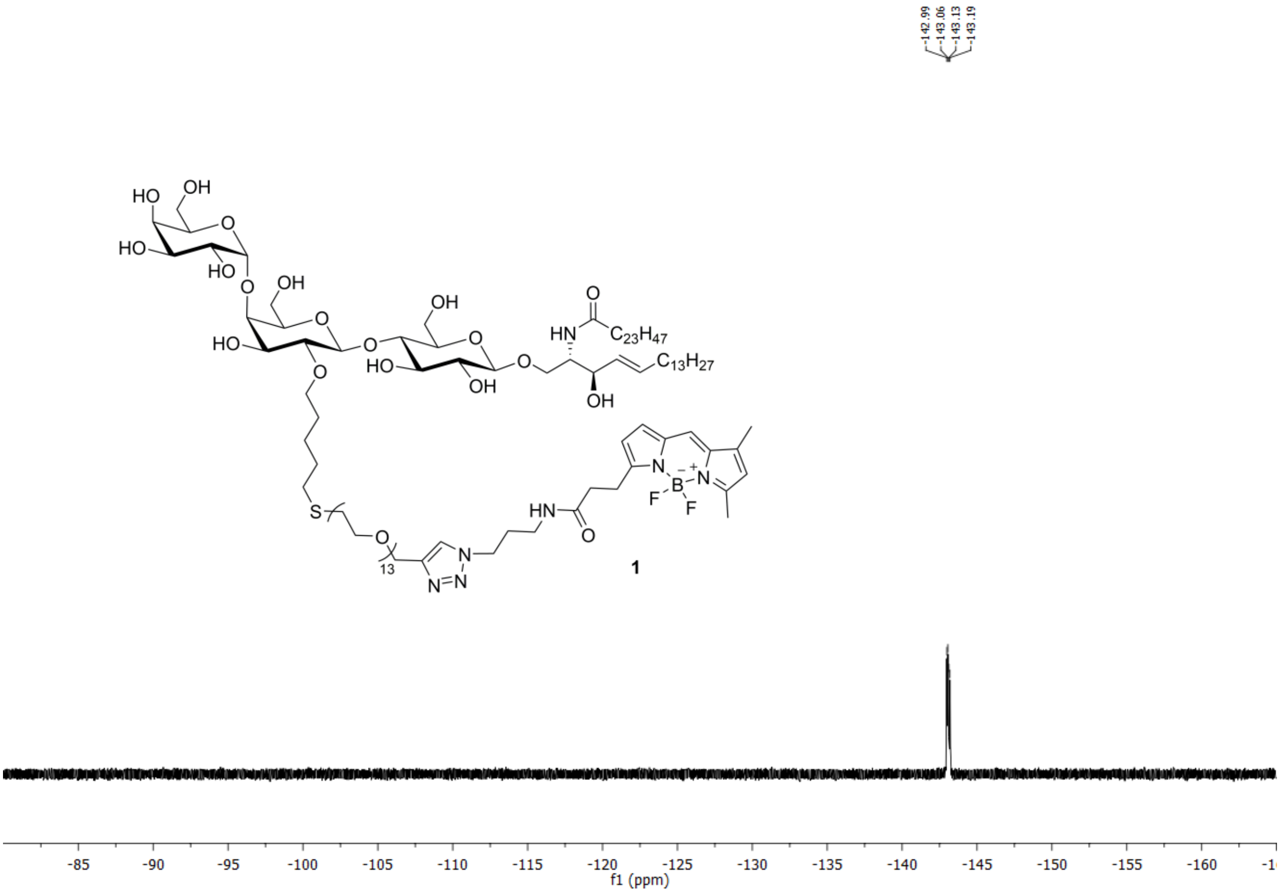
-142.99
-143.06
-143.13
-143.19
1
-85 -90 -95 -100 -105 -110 -115 -120 -125 -130 -135 -140 -145 -150 -155 -160
f1 (ppm)

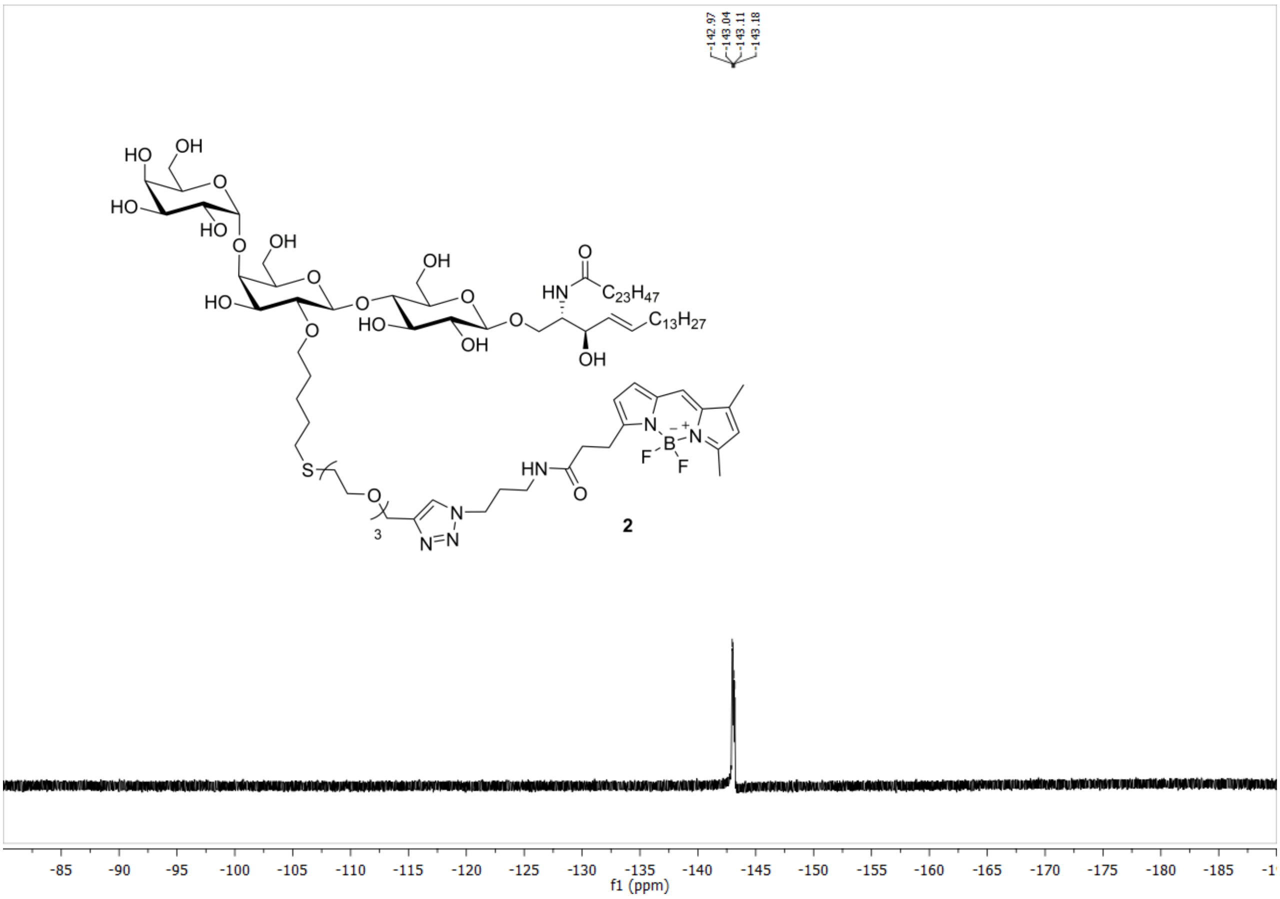
-142.97
-143.04
-143.11
-143.18
2
-85 -90 -95 -100 -105 -110 -115 -120 -125 -130 -135 -140 -145 -150 -155 -160 -165 -170 -175 -180 -185
f1 (ppm)

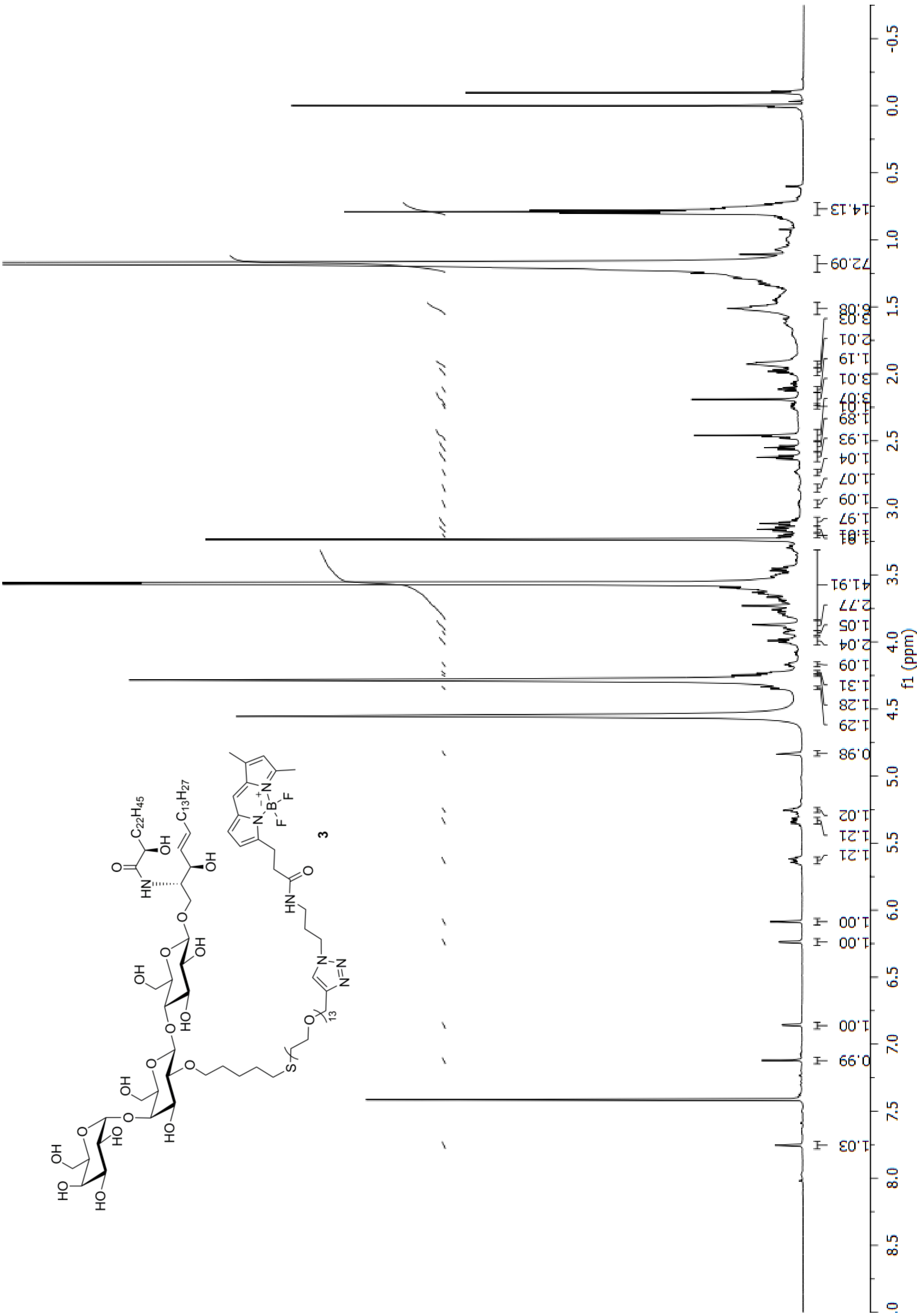
3
f1 (ppm)

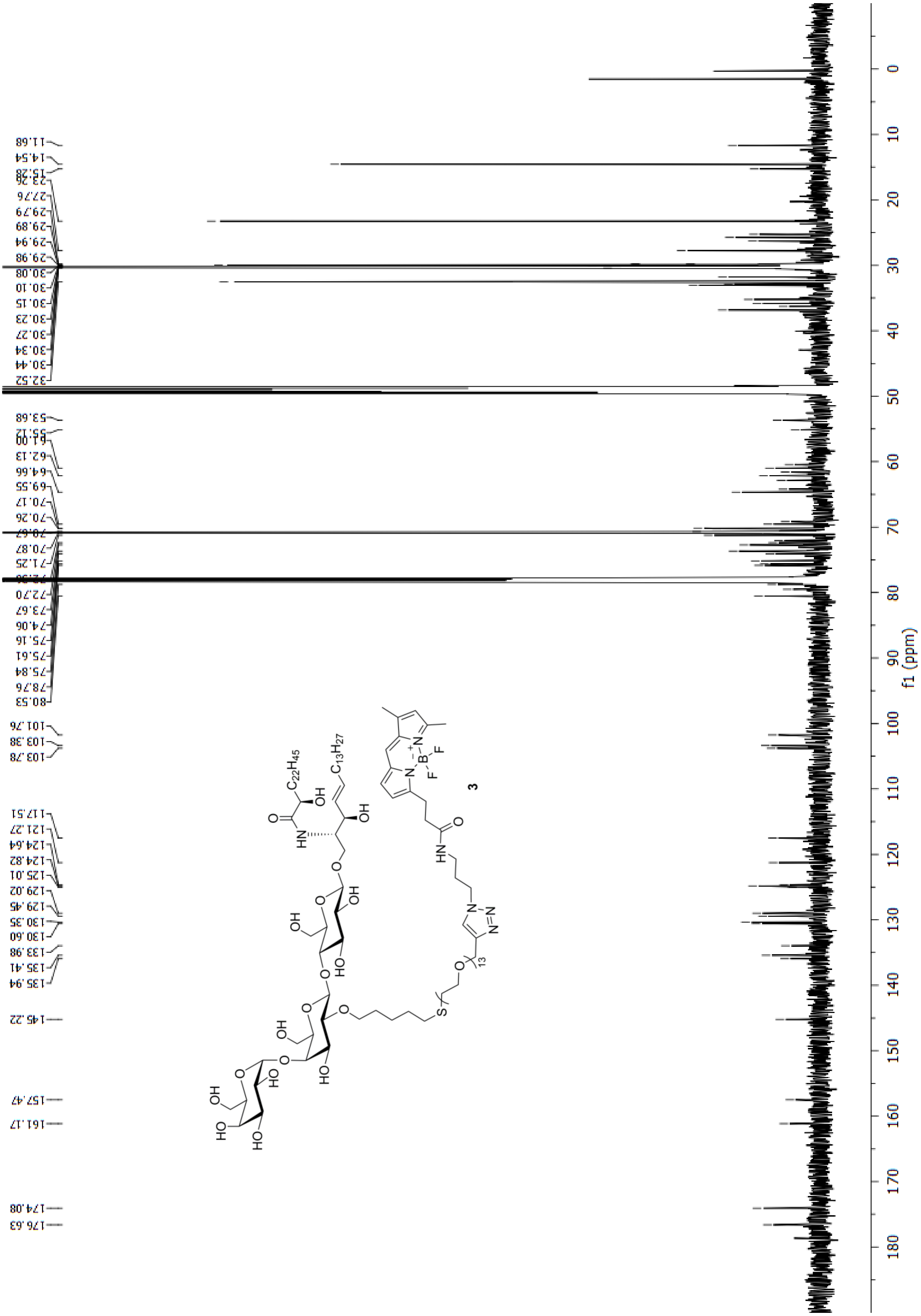
11.68
14.54
15.28
23.26
27.76
29.79
29.89
29.94
29.98
30.08
30.10
30.15
30.23
30.27
30.34
30.44
32.52
53.68
55.12
61.00
62.13
64.66
69.55
70.17
70.26
70.67
70.87
71.25
72.70
73.67
74.06
75.16
75.61
75.84
78.76
80.53
101.76
103.38
103.78
117.51
121.27
124.64
124.82
125.01
129.02
129.45
130.35
130.60
133.98
135.41
135.94
145.22
157.47
161.17
174.08
176.63
f1 (ppm)
$C_{22}H_{45}$
$C_{13}H_{27}$

3

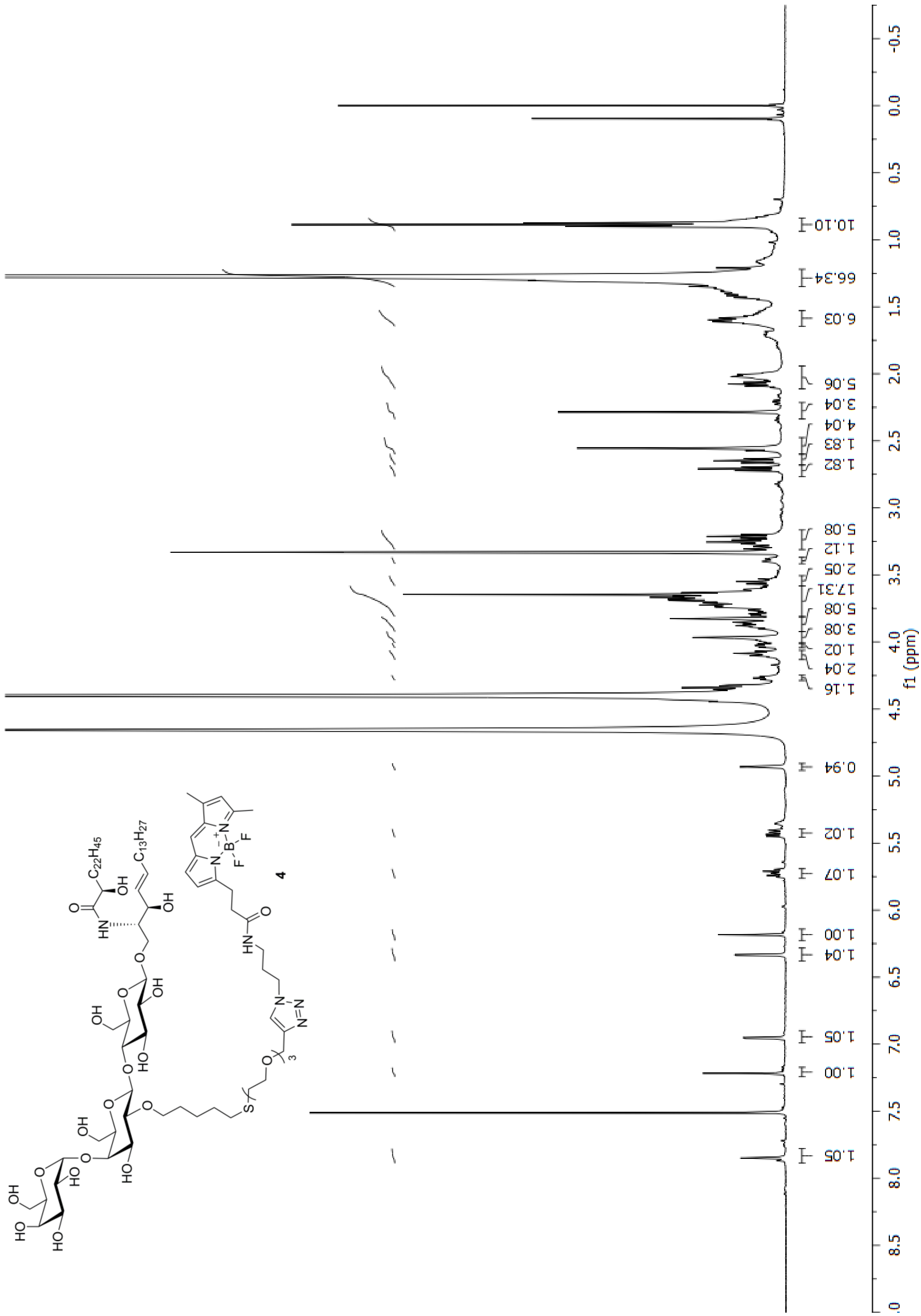
4
f1 (ppm)

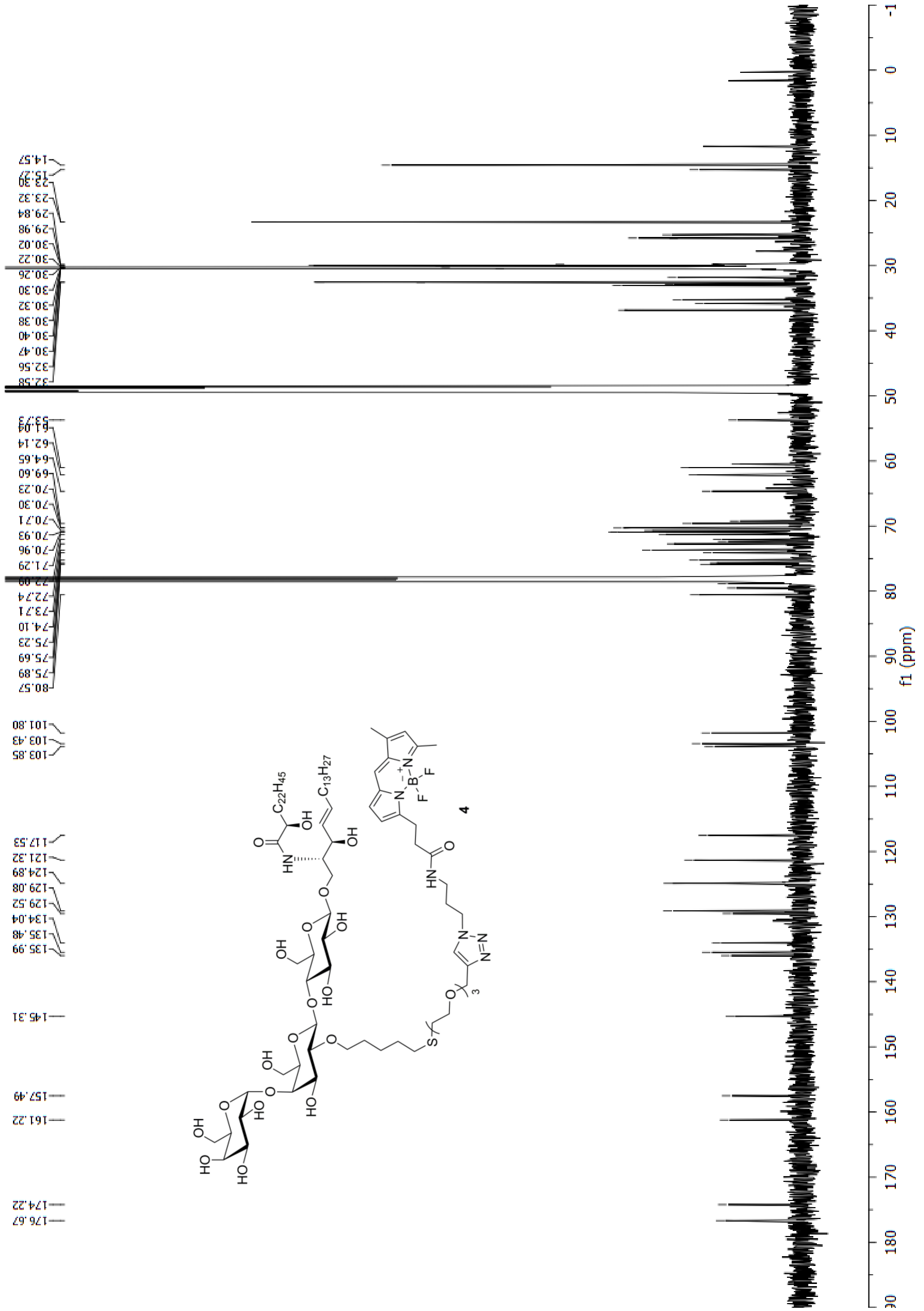
14.57
15.27
23.30
23.32
29.84
29.98
30.02
30.22
30.26
30.30
30.32
30.38
30.40
30.47
32.56
32.58
53.73
61.04
62.14
64.65
69.60
70.23
70.30
70.71
70.93
70.96
71.29
72.09
72.74
73.71
74.10
75.23
75.69
75.89
80.57
101.80
103.43
103.85
117.53
121.32
124.89
129.08
129.52
134.04
135.48
135.99
145.31
157.49
161.22
174.22
176.67
4
f1 (ppm)

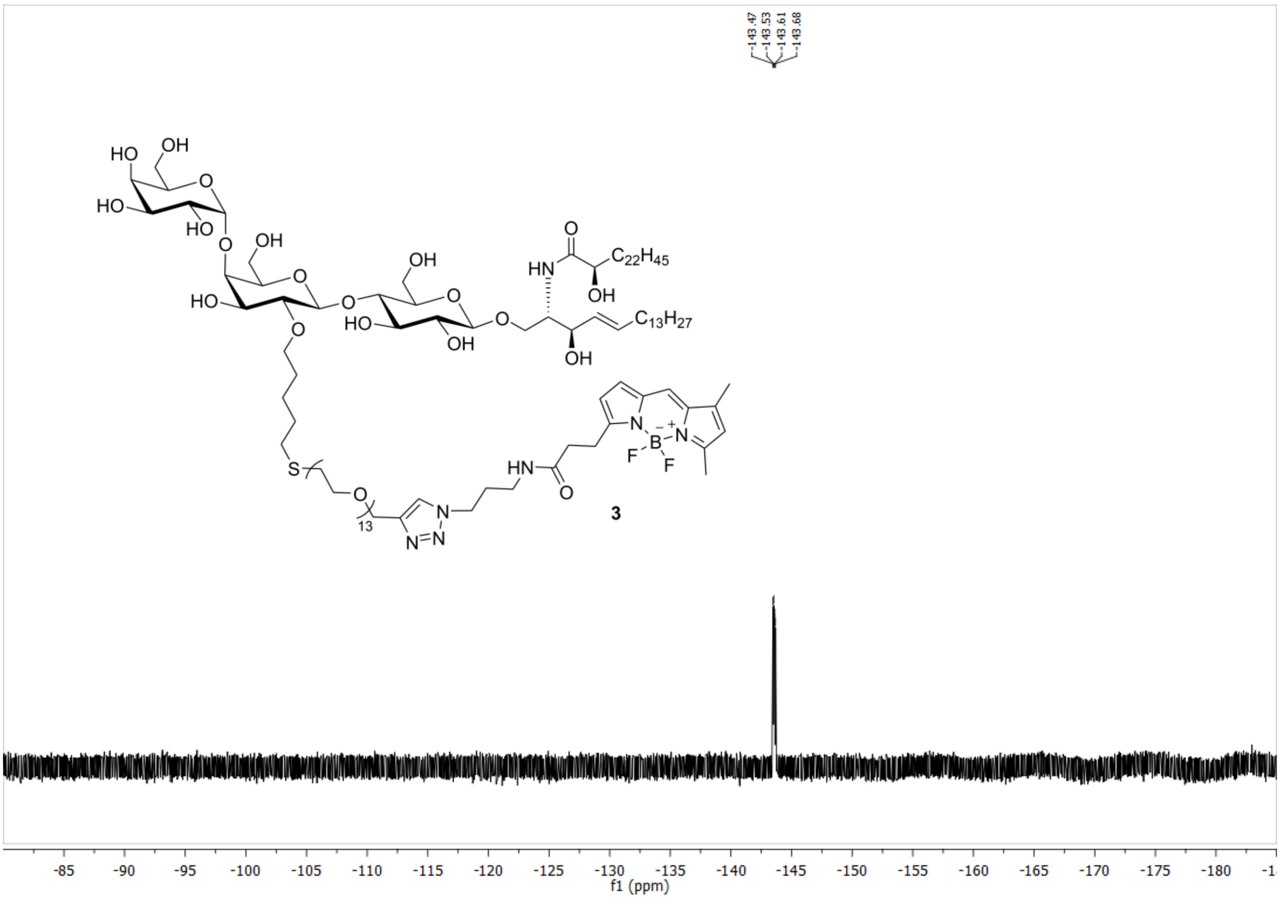
-143.47
-143.53
-143.61
-143.68
3
f1 (ppm)

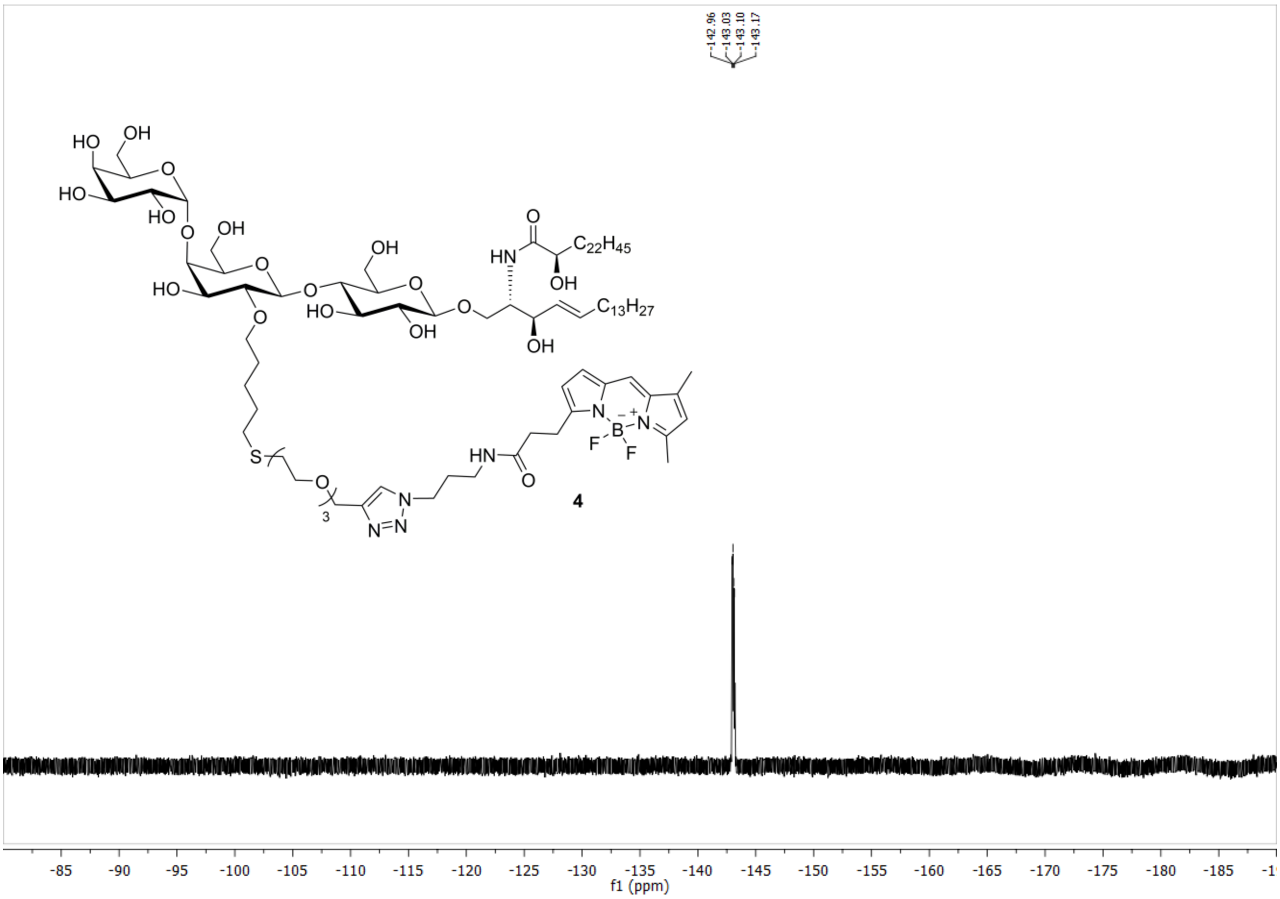
-142.96
-143.03
-143.10
-143.17
4
f1 (ppm)

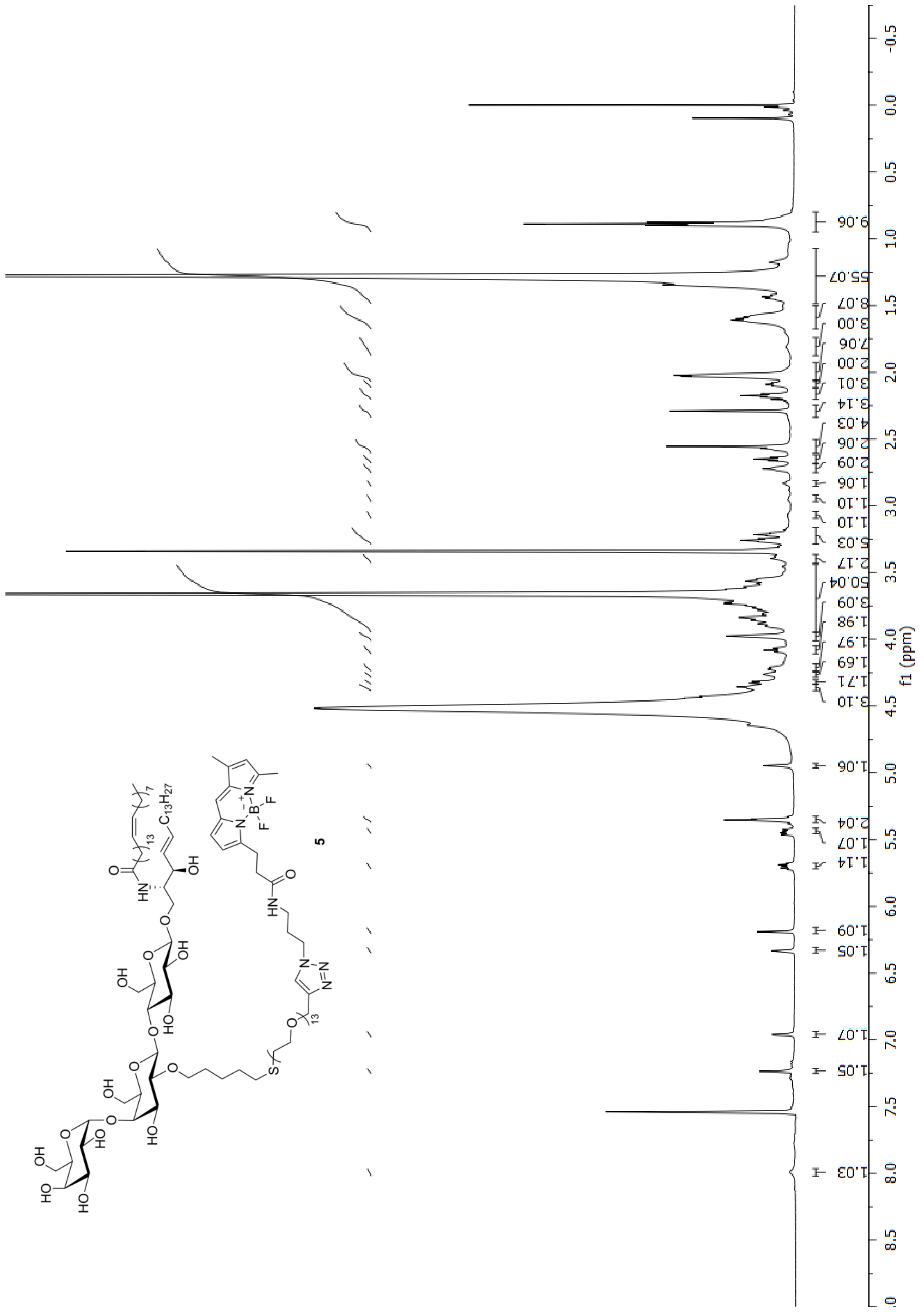

5

f1 (ppm)

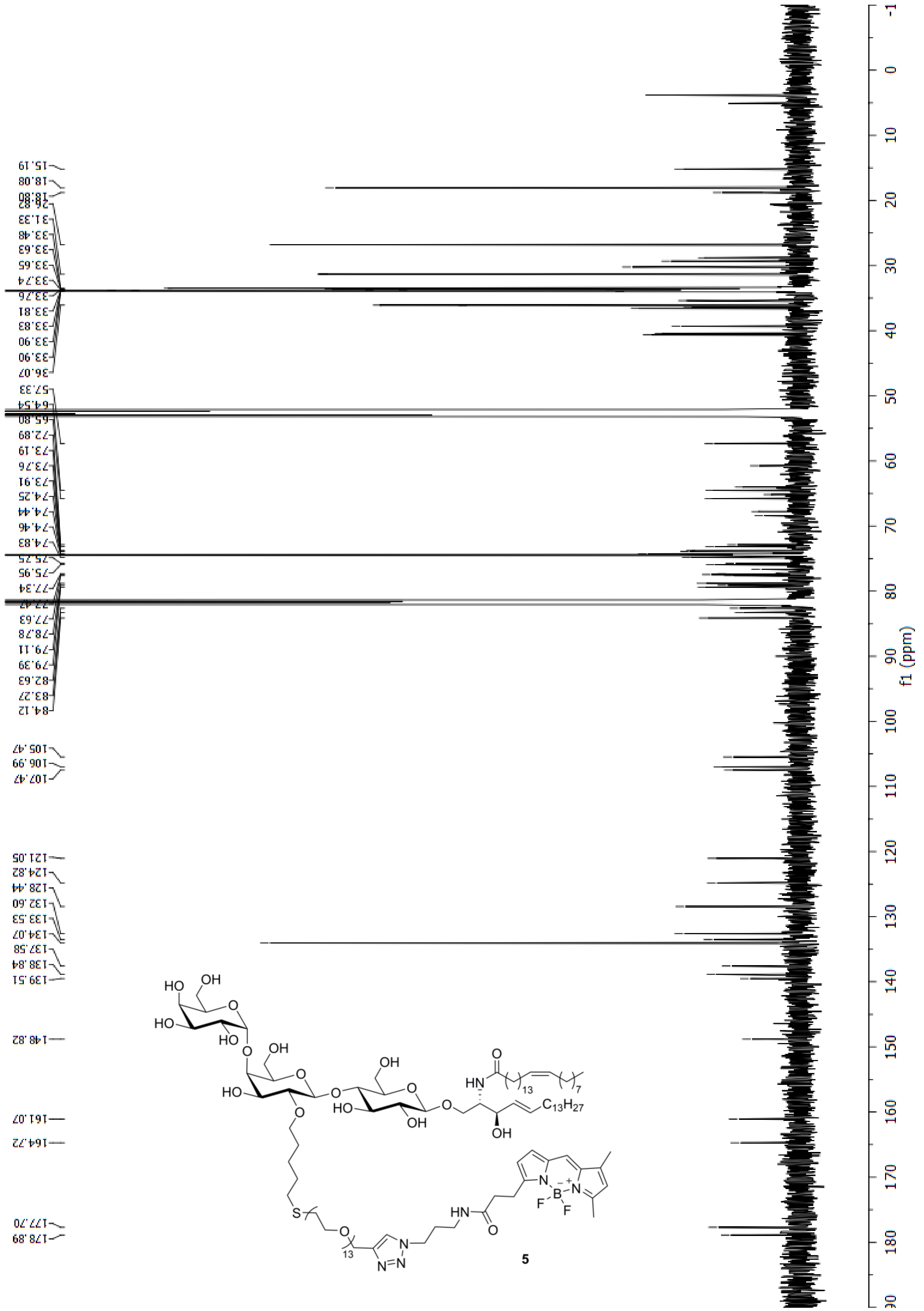

5

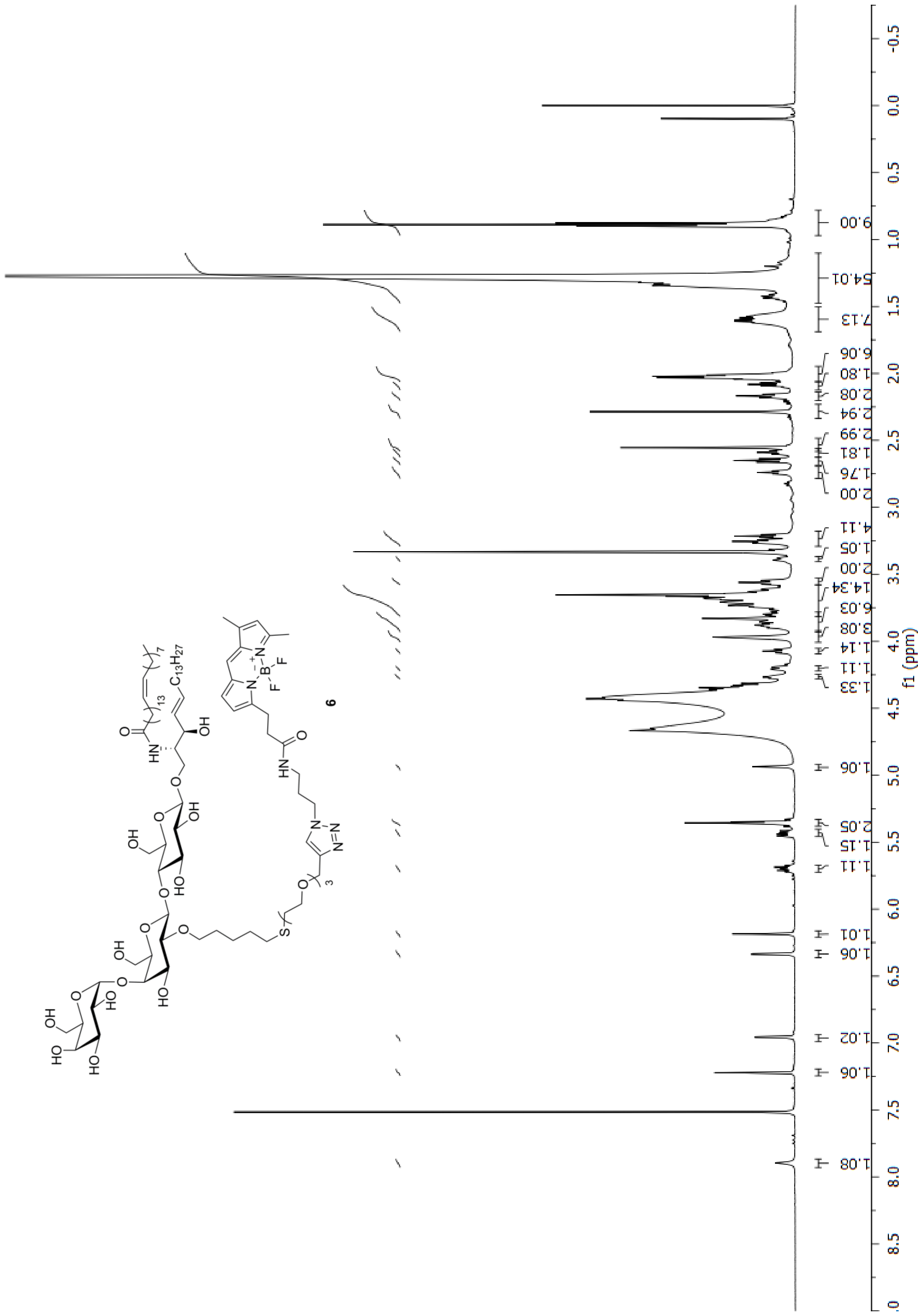
6
f1 (ppm)

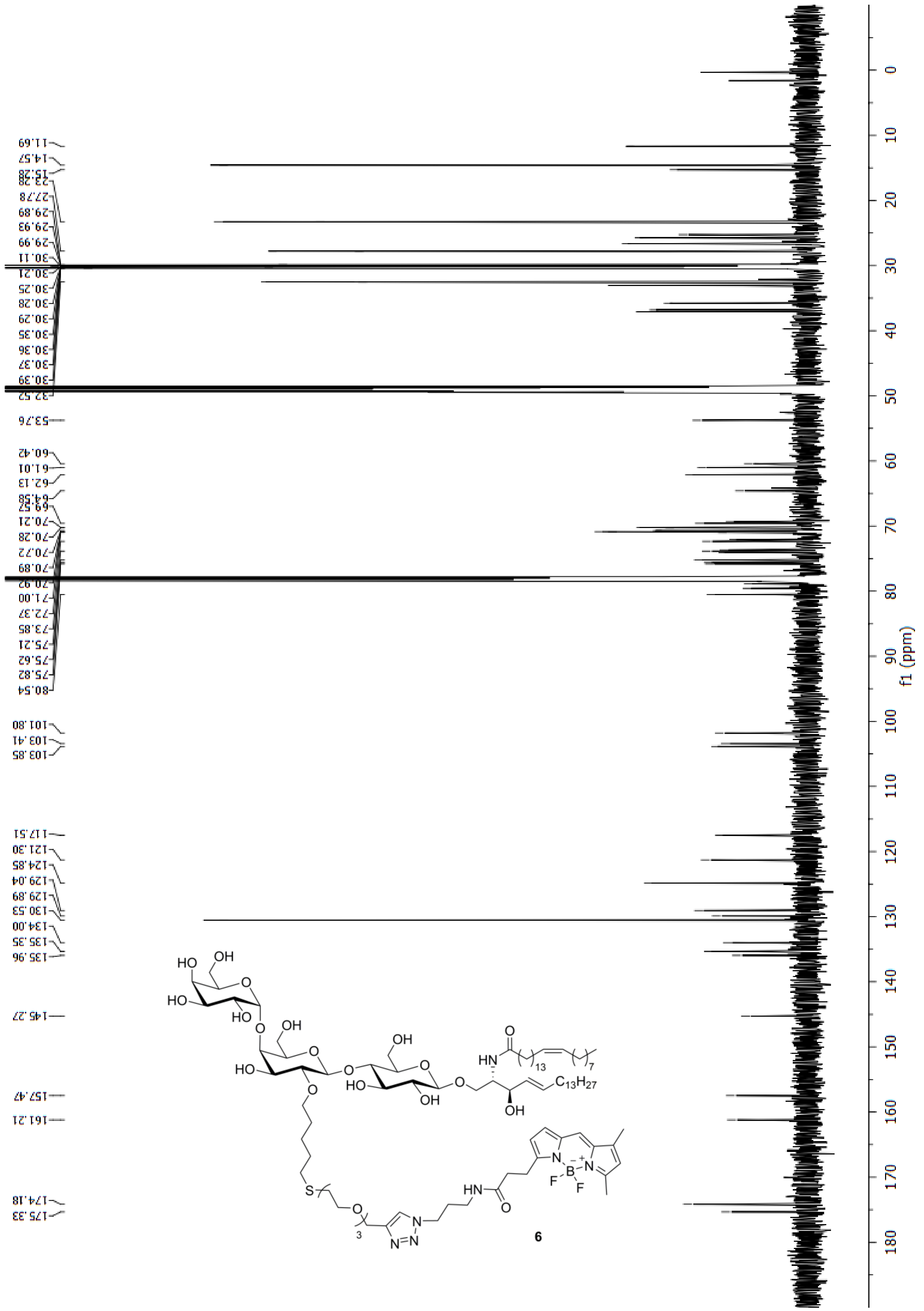
f1 (ppm)
6

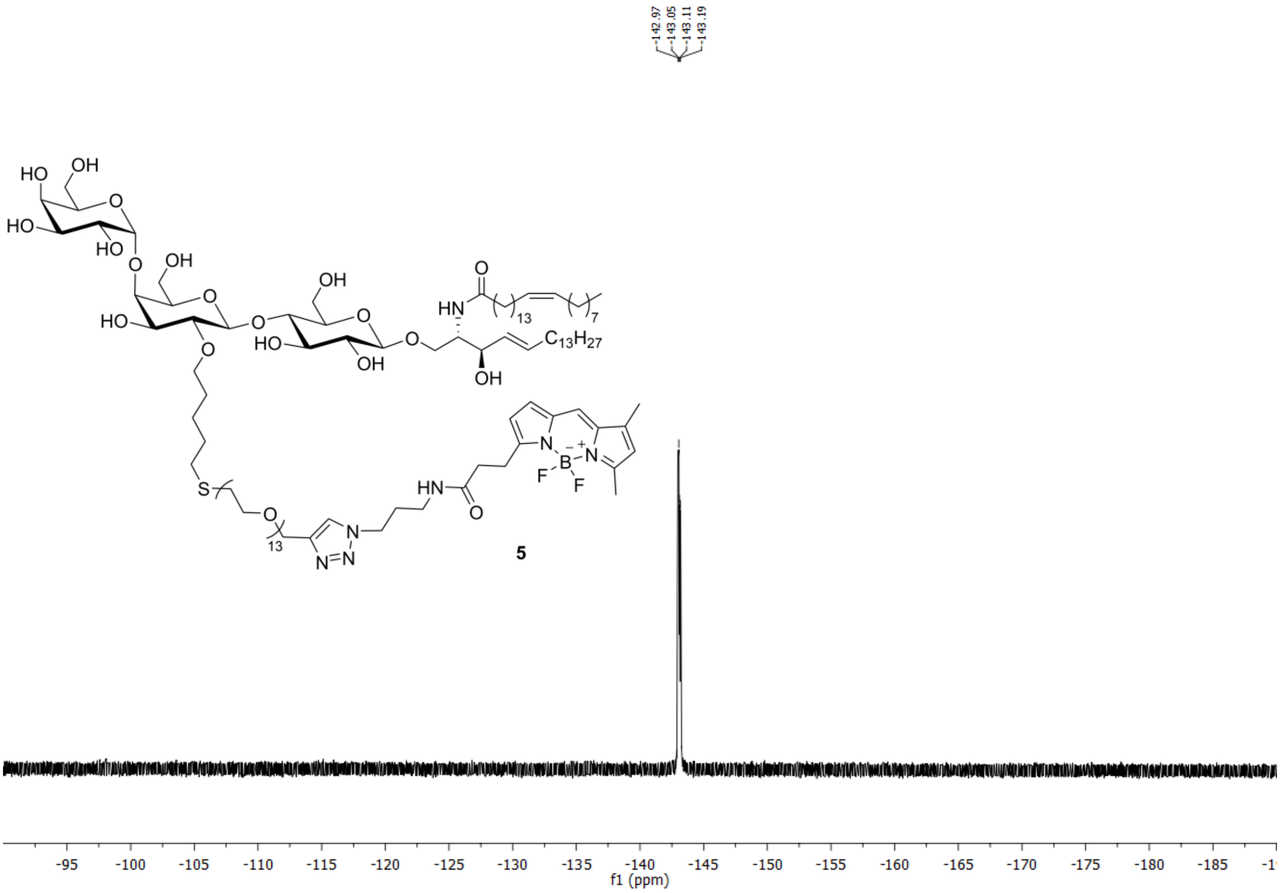
-142.97
-143.05
-143.11
-143.19
-95 -100 -105 -110 -115 -120 -125 -130 -135 -140 -145 -150 -155 -160 -165 -170 -175 -180 -185
f1 (ppm)

5

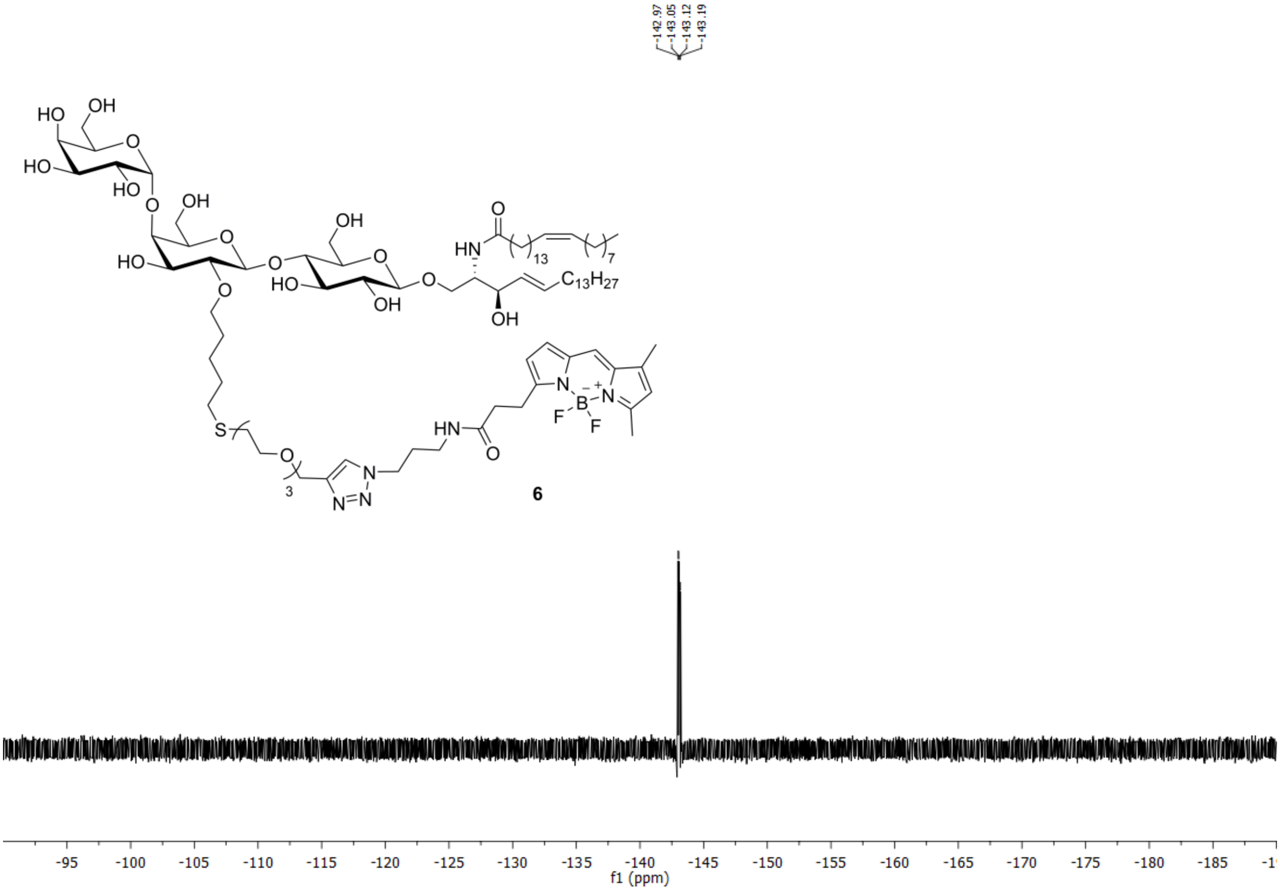
-142.97
-143.05
-143.12
-143.19
-95 -100 -105 -110 -115 -120 -125 -130 -135 -140 -145 -150 -155 -160 -165 -170 -175 -180 -185
f1 (ppm)

6

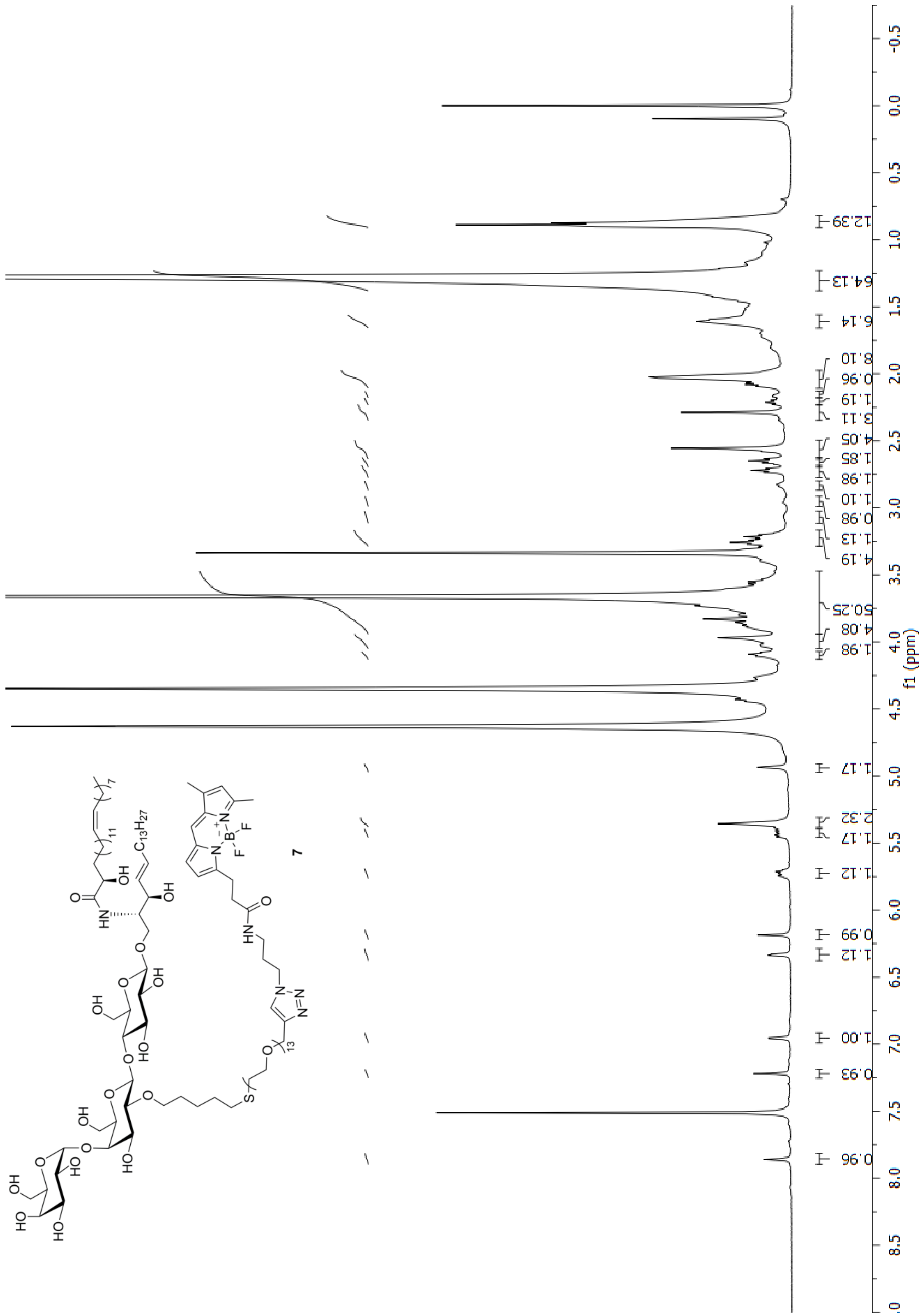
7
f1 (ppm)

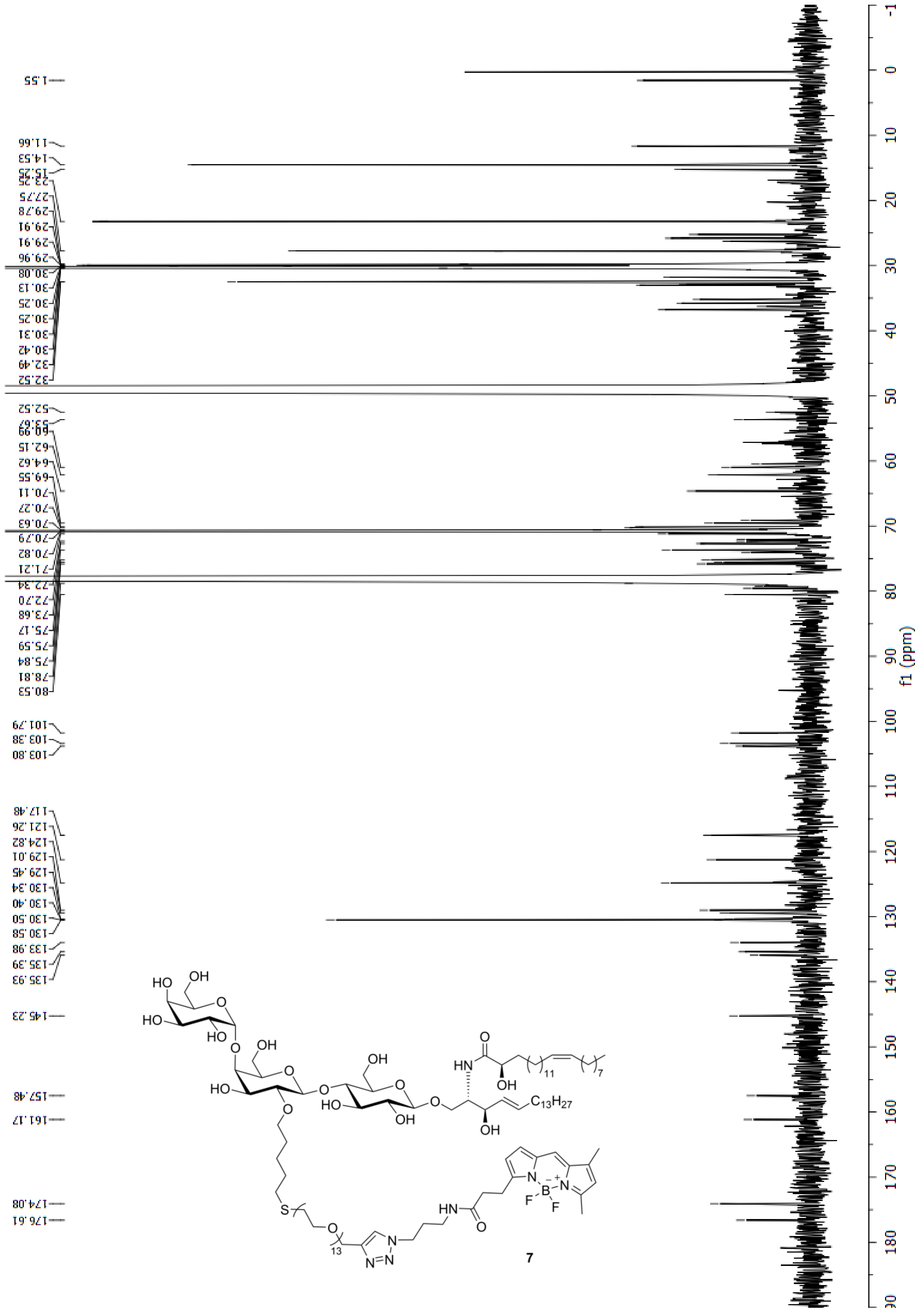

7

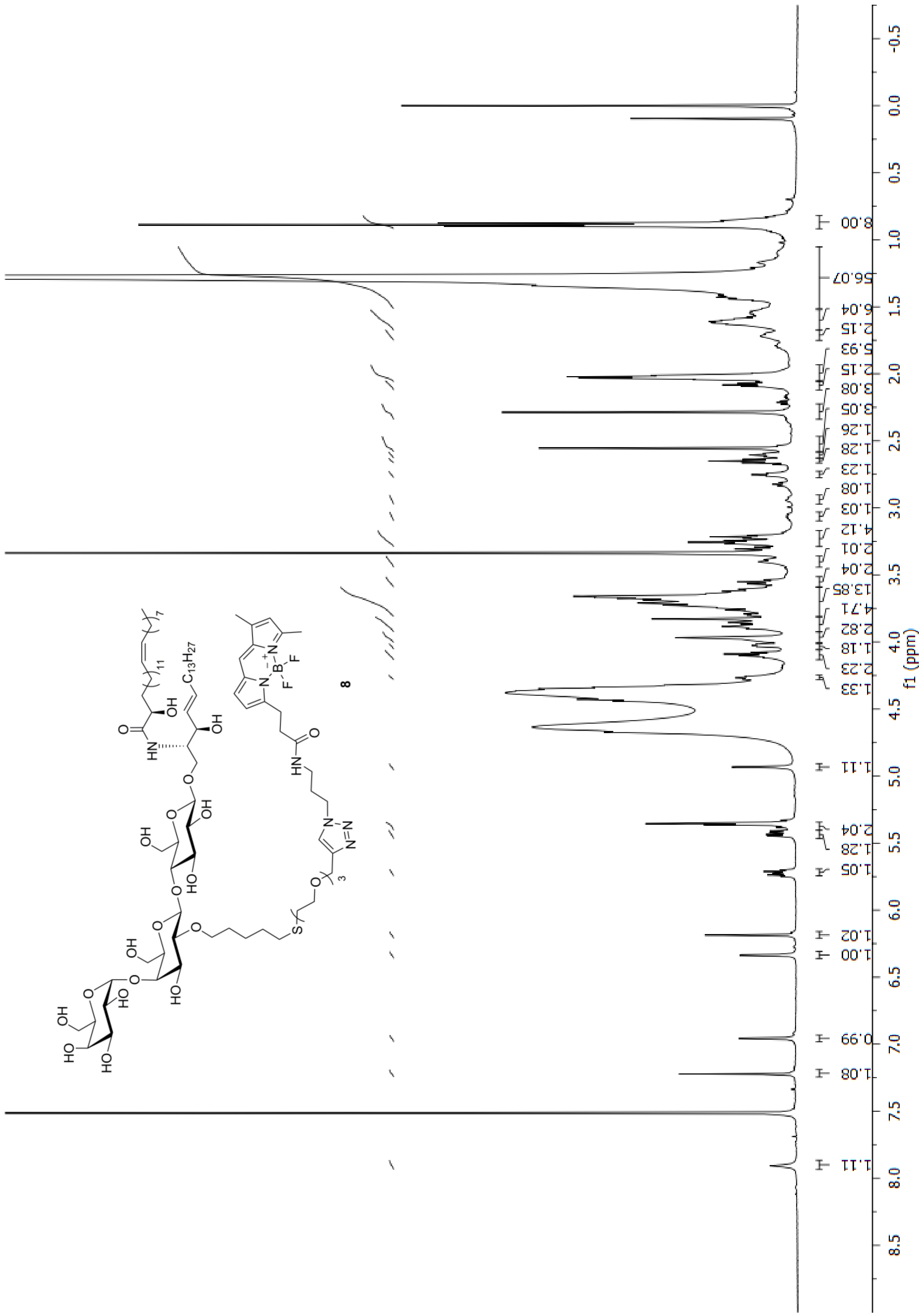
f1 (ppm)
8

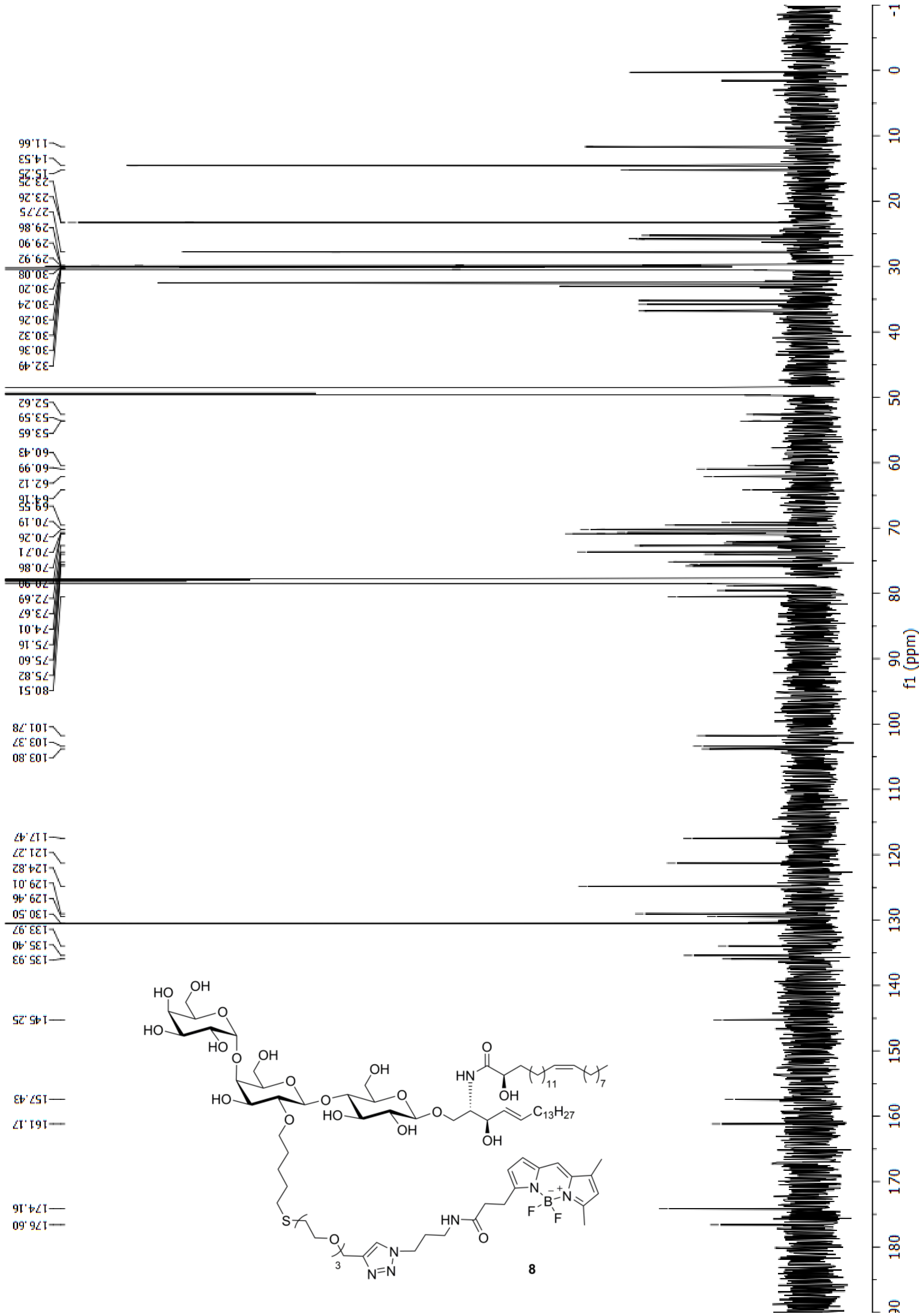

8

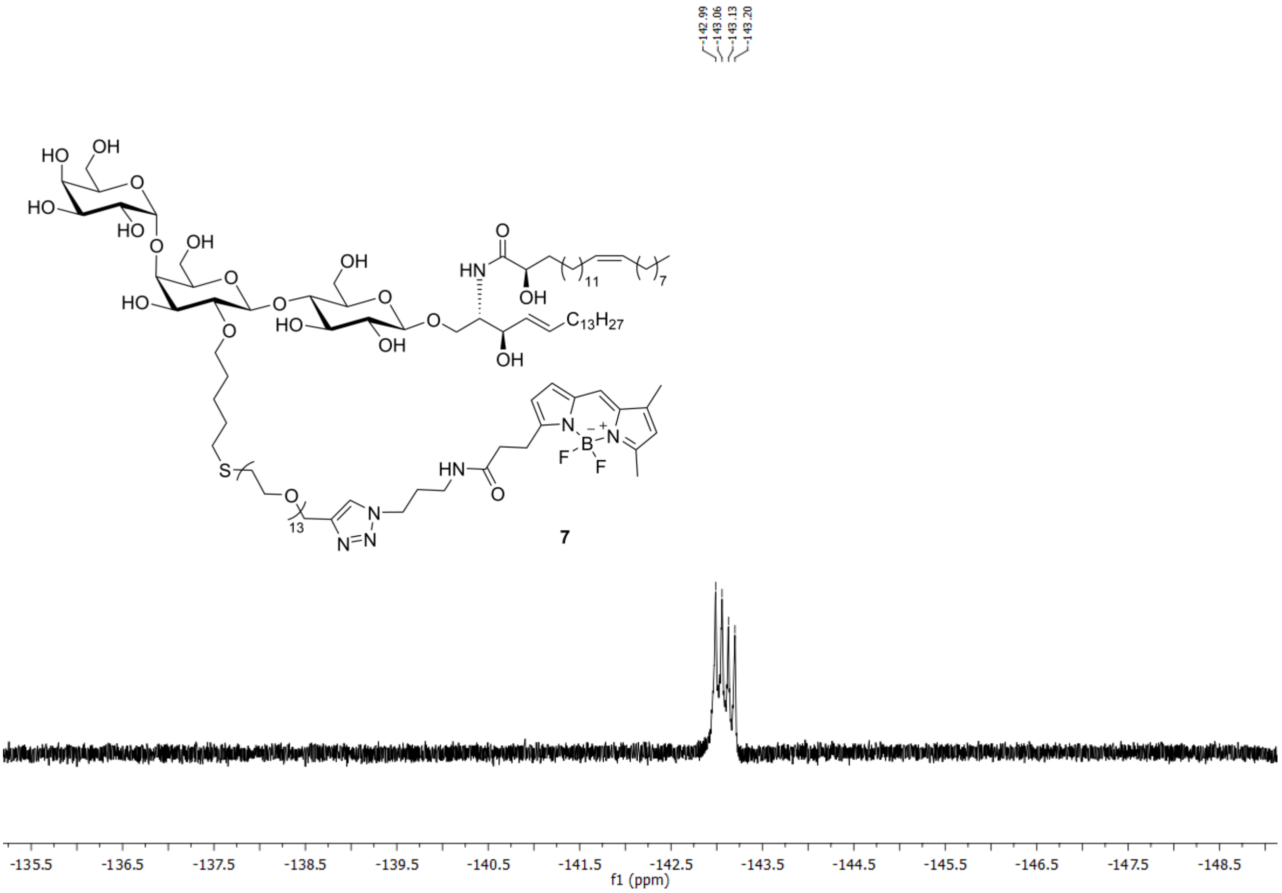
-142.99
-143.06
-143.13
-143.20
HO
OH
O
HO
HO
O
OH
O
HO
O
O
OH
O
HO
OH
O
HN
O
OH
11
7
C13H27
OH
S
O
13
N
N=N
HN
O
N
B
N
F
F
-135.5
-136.5
-137.5
-138.5
-139.5
-140.5
-141.5
-142.5
-143.5
-144.5
-145.5
-146.5
-147.5
-148.5
f1 (ppm)

7

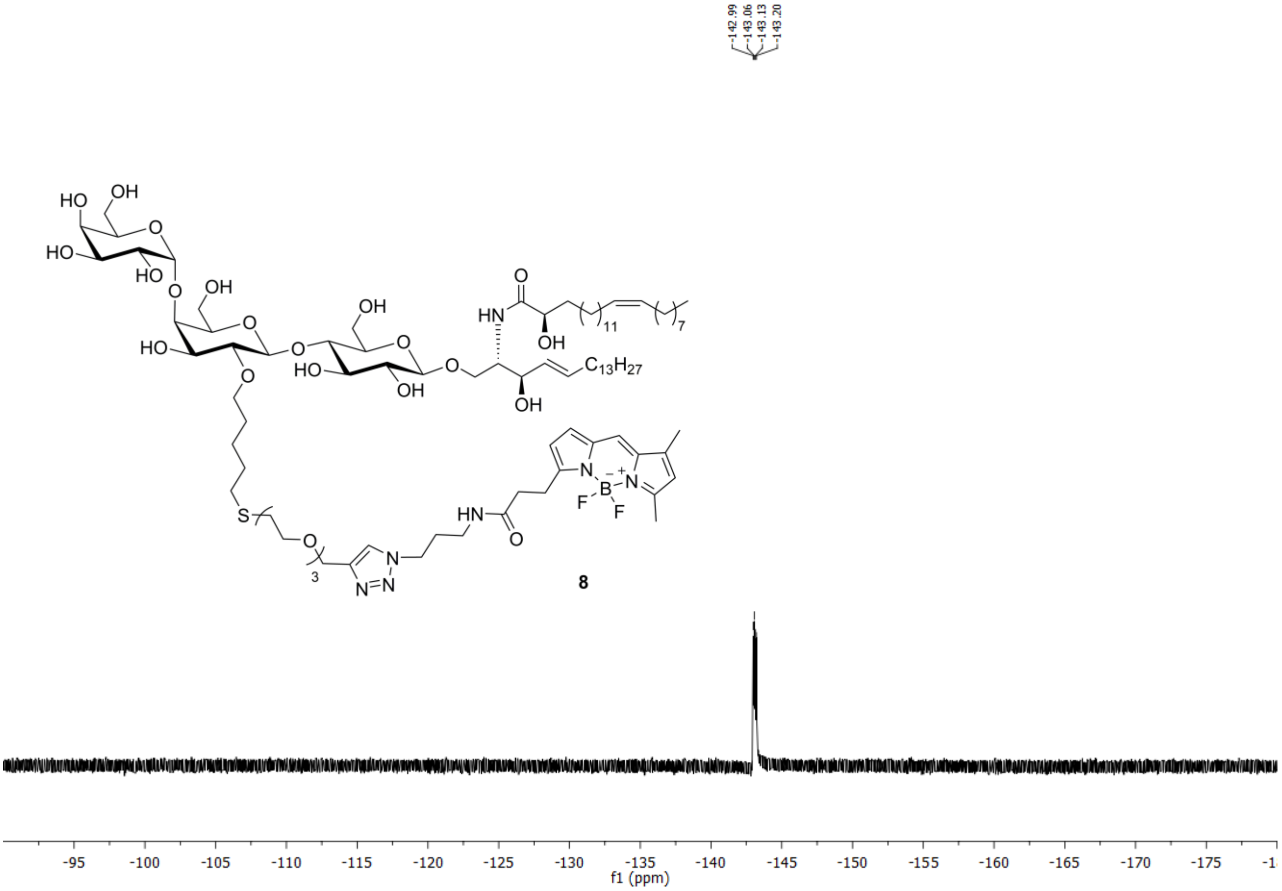
-142.99
-143.06
-143.13
-143.20
HO
OH
O
HO
HO
O
OH
O
HO
O
O
OH
O
HO
OH
O
HN
O
OH
11
7
C13H27
OH
S
O
3
N
N=N
HN
O
N
B
N
F
F
-95
-100
-105
-110
-115
-120
-125
-130
-135
-140
-145
-150
-155
-160
-165
-170
-175
f1 (ppm)

8

Materials

1,2-Dioleoyl-*sn*-glycero-3-phosphocholine (DOPC), sphingomyelin from porcine brain (SM-porc), palmitoyl SM ($C_{16:0}$), stearoyl SM ($C_{18:0}$) and lignoceroyl SM ($C_{24:0}$) were purchased from Avanti Polar Lipids (Alabaster, AL). Arachidoyl SM ($C_{20:0}$) and behenoyl SM ($C_{22:0}$) were from Matreya (State College, PA). Cholesterol (Chol) and sulforhodamine-1,2-dihexanoyl-*sn*-glycero-3-phosphoethanolamine (Texas Red-DHPE) were obtained from Sigma-Aldrich (Taufkirchen, Germany). Dy731-DOPE was synthesized as described previously.[8] Bovine serum albumin (BSA) was purchased from Carl Roth GmbH (Karlsruhe, Germany). STxB-Cy5 was purified as described previously.[9]

Preparation of giant unilamellar vesicles (GUVs)

GUVs were prepared by electroformation. Each lipid composition (total lipid: 1 mg) was dissolved in chloroform/methanol (2:1) and the solution was deposited equally on two indium tin oxide (ITO) cover slides that were preheated to 55 °C. The solvent was removed under reduced pressure for 3 h at 55 °C. A Teflon ring was placed between the two ITO slides and the chamber was filled with 1.7 mL sucrose solution (298 mOsmol/L). An AC field was applied (U = 1.66 V_{pp}, f = 12 Hz) for 3 h at 55 °C. Resulting GUVs were stored at room temperature before use.

Fluorescence microscopy of GUVs

A petri dish was passivated with BSA (8 mg/mL in phosphate buffered saline (PBS): 1.5 mM KH_2PO_4, 8.1 mM Na_2HPO_4, 2.7 mM KCl, 136.9 mM NaCl, pH 7.4) at 4 °C overnight. After removing the BSA solution, the petri dish was filled with 2 mL PBS and washed with 50 mL PBS. 100 µL of the GUV solution were sedimented through a PBS filled 5 mL tip into the petri dish. After 10 min, the GUVs were analyzed with a confocal laser scanning microscope (LSM 710 or 880, Carl Zeiss, Jena, Germany). Both microscopes were equipped with a W Plan-Apochromat 40 × objective and an Ar 488 nm, a DPSS 561 nm and a HeNe 633 nm laser line. Fluorescence intensities were detected in a range of 500-620 nm (BODIPY), 565-620 nm (Texas Red-DHPE), and 640-758 nm (Dy731, Cy5-STxB).

Self-quenching of BODIPY labeled Gb_3

Different amounts of **1** were reconstituted in DOPC GUVs in ultrapure water at room temperature (lipid concentration 16 µM), and the fluorescence intensity at 488 nm was readout before (F) and after (F_0) Triton-X addition.[10]

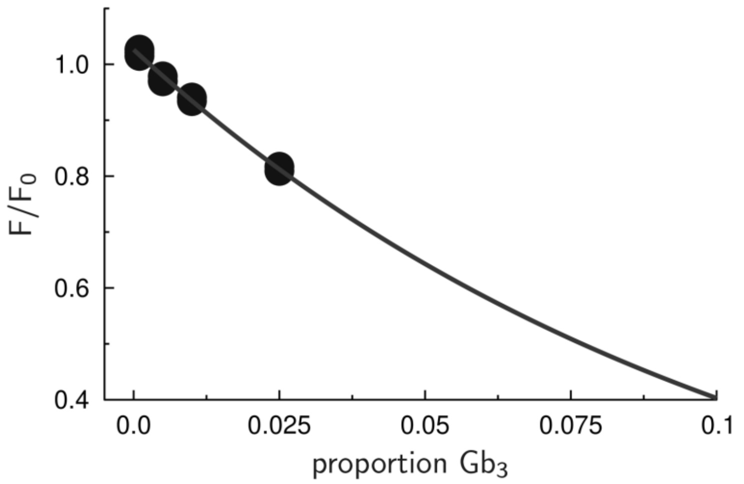

Figure S1. Self-quenching of BODIPY labeled Gb_3 (**1**). The fluorescence intensity of DOPC GUVs with different amounts of Gb_3 was readout before Triton-X addition (*F*) and after (*F_0*).

l_o distribution of different Gb_3 species with a PEG_3 linker (SM-porc)

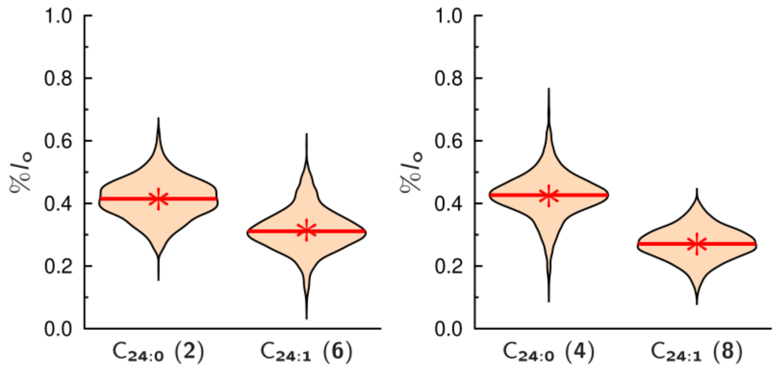

Figure S2. *l_o* distribution of different Gb_3 species with a PEG_3 linker in GUVs composed of DOPC/SM-porc/Chol/Gb_3/Dy731 (39/39/20/1/1). The partition of Gb_3 sphingolipids with a saturated fatty acid ($C_{24:0}$) was compared with those carrying a monounsaturated fatty acid ($C_{24:1}$). The mean values are given as a red star, while the solid red lines show the median value, the number of line scans are given in Table S1.

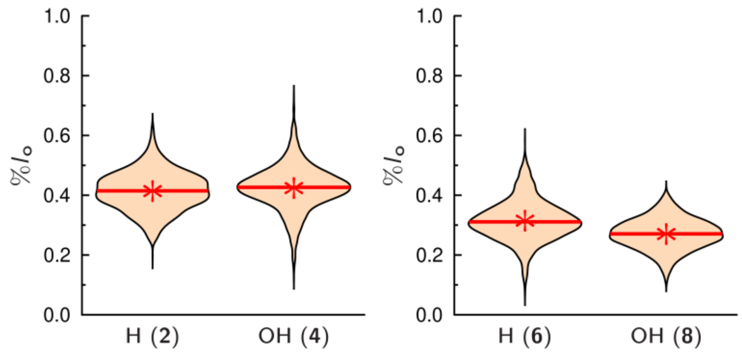

Figure S3. *l_o* distribution of different Gb_3 species with a PEG_3 linker in GUVs composed of DOPC/SM-porc/Chol/Gb_3/Dy731 (39/39/20/1/1). The partition of Gb_3 sphingolipids with no hydroxylation (H) was compared with those carrying an α-hydroxylation (OH). The mean values are given as red stars, while the red solid lines show the median values, the number of line scans are given in Table S1.

l_o distribution of different Gb_3 species with a PEG_3 linker (chemically defined SM)

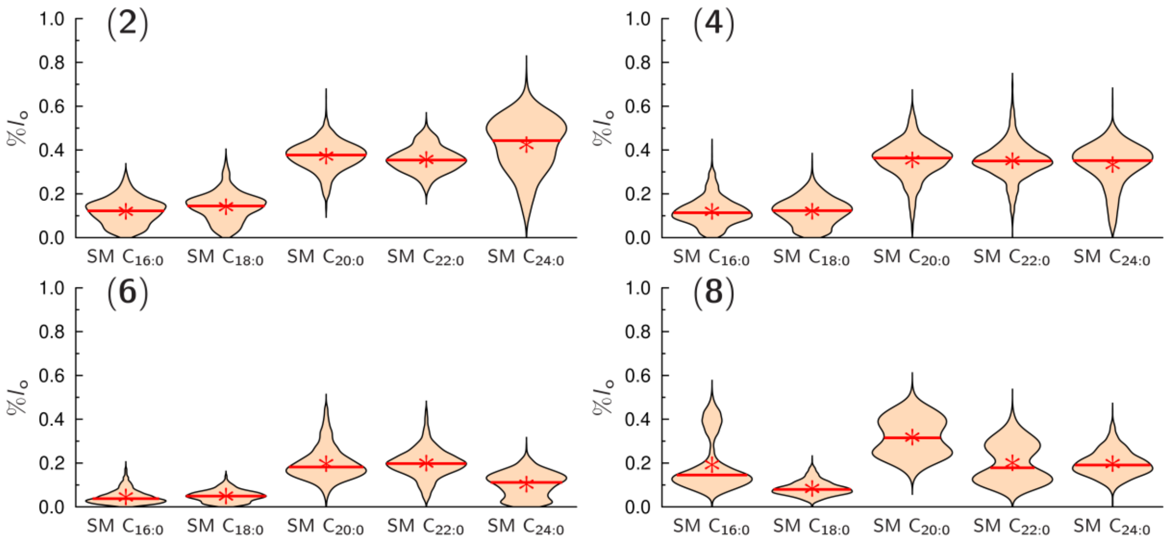

Figure S4. l_o distribution of different Gb_3 species with a PEG_3 linker composed of DOPC/SM/Chol/Gb_3/Dy731 (39/39/20/1/1). The partition of four different Gb_3 sphingolipids in l_o/l_d phase separated GUVs with different sphingomyelin species are shown: SM $C_{16:0}$ (palmitoyl SM), SM $C_{18:0}$ (stearoyl SM), SM $C_{20:0}$ (arachidoyl SM), SM $C_{22:0}$ (behenoyl SM) and SM $C_{24:0}$ (lignoceroyl SM). The mean values are given as red stars and the solid red lines show the median, the number of line scans ins given in Table S1.

Table S1. Mean values of the l_o distributions (%l_o) for the different Gb_3 glycosphingolipids with the PEG_3 linker in GUVs composed of DOPC/SM/Chol/Gb_3/Dy731 (39/39/20/1/1) varying in the SM species: SM $C_{16:0}$ (palmitoyl SM), SM $C_{18:0}$ (stearoyl SM), SM $C_{20:0}$ (arachidoyl SM), SM $C_{22:0}$ (behenoyl SM) and SM $C_{24:0}$ (lignoceroyl SM). The errors are the standard deviation of the mean.

**Gb_3**	**%l_o(SM $C_{16:0}$) (*N*)**	**%l_o(SM $C_{18:0}$) (*N*)**	**%l_o(SM $C_{20:0}$) (*N*)**	**%l_o(SM $C_{22:0}$) (*N*)**	**%l_o(SM $C_{24:0}$) (*N*)**
**2**	0.12 ± 0.06 (1890)	0.14 ± 0.07 (1539)	0.37 ± 0.07 (2188)	0.36 ± 0.06 (2363)	0.42 ± 0.13 (1695)
**4**	0.12 ± 0.07 (2769)	0.12 ± 0.07 (2549)	0.36 ± 0.09 (2907)	0.36 ± 0.09 (2155)	0.33 ± 0.10 (2431)
**6**	0.05 ± 0.04 (2384)	0.05 ± 0.03 (2413)	0.20 ± 0.08 (2227)	0.20 ± 0.07 (2259)	0.10 ± 0.06 (2505)
**8**	0.19 ± 0.12 (3086)	0.09 ± 0.04 (1768)	0.32 ± 0.09 (1988)	0.20 ± 0.09 (2695)	0.20 ± 0.06 (1828)

References

[1] H. Tanaka, R. Takeuchi, M. Jimbo, N. Kuniya, T. Takahashi, *Chem. Eur. J.* **2013**, *19*, 3177.
[2] H. Isobe, K. Cho, N. Solin, D. B. Werz, P. H. Seeberger, E. Nakamura, *Org. Lett.* **2007**, *9*, 4611.
[3] C. Bucher, R. Gilmour, *Angew. Chem., Int. Ed.* **2010**, *49*, 8724.
[4] O. M. Schütte, A. Ries, A. Orth, L. J. Patalag, W. Römer, C. Steinem, D. B. Werz, *Chem. Sci.* **2014**, *5*, 3104.
[5] M. Pawliczek, J. Wallbaum, D. Werz, *Synlett* **2014**, *25*, 1435.
[6] a) M. S. Sandbhor, J. A. Key, I. S. Strelkov, C. W. Cairo, *J. Org. Chem.* **2009**, *74*, 8669; b) E. D. Goddard-Borger, R. V. Stick, *Org. Lett.* **2011**, *13*, 2514; c) E. D. Goddard-Borger, R. V. Stick, *Org. Lett.* **2007**, *9*, 3797; d) K. Sa-Ei, J. Montgomery, *Tetrahedron* **2009**, *65*, 6707; e) T. Murakami, K. Furusawa, *Tetrahedron* **2002**, *58*, 9257.
[7] a) W. R. Roush, J. A. Hunt, *J. Org. Chem.* **1995**, *60*, 798; b) P. Garner, J. M. Park, *Org. Synth.* **1992**, *70*, 18; c) A. Dondoni, D. Perrone, *Org. Synth.* **2000**, *77*, 64; d) P. Garner, *Tetrahedron Lett.* **1984**, *25*, 5855.
[8] L. J. Patalag, J. Sibold, O. M. Schütte, C. Steinem, D. B. Werz, *ChemBioChem* **2017**, *18*, 2171.
[9] K. Sandvig, M. Ryd, O. Garred, E. Schweda, P. K. Holm, B. vanDeurs, *J. Cell Biol.* **1994**, *126*, 53.
[10] R. I. MacDonald, *J. Biol. Chem.* **1990**, *265*, 13533.
